# Expanding the Chemical Space of Cyclic Polyphthalaldehyde via Post‐Functionalization

**DOI:** 10.1002/advs.202522465

**Published:** 2025-12-23

**Authors:** Liting He, Haiyan Zhou, Qiushi Chen, Xing Guo, Han Liu, Xuechen Li

**Affiliations:** ^1^ Department of Chemistry The State Key Laboratory of Synthetic Chemistry The University of Hong Kong Hong Kong SAR P. R. China; ^2^ Laboratory for Synthetic Chemistry and Chemical Biology Limited Hong Kong Science Park Pak Shek Kok Hong Kong SAR P. R. China; ^3^ Materials Innovation Institute for Life Sciences and Energy (MILES) HKU‐SIRI Shenzhen 518000 P. R. China; ^4^ Shanghai‐Hong Kong Joint Laboratory in Chemical Synthesis Shanghai Institute of Organic Chemistry University of Chinese Academy of Sciences Chinese Academy of Sciences 345 Lingling Road Shanghai 200032 P. R. China

**Keywords:** click chemistry, cyclic polymer, post‐functionalization, poly‐*o*‐phthalaldehyde, water soluble

## Abstract

Cyclic polyphthalaldehydes (cPPAs) are hydrophobic cyclic polymers with stimuli‐responsive depolymerization property. The introduction of hydrophilic functionalities to cPPAs is challenging due to the intrinsic limitation of cationic polymerization. Herein, we developed a post‐functionalization approach to achieve water‐soluble cPPA derivatives with high molecular weight. The cPPA modified with cell‐penetrating oligoarginine underwent cellular uptake via endocytosis and acid‐triggered depolymerization in lysosome, which provided new potential for biological application.

## Graphical abstract

In this work, we developed a post‐functionalization approach to achieve water‐soluble cyclic polyphthalaldehyde (cPPA) polymers via click chemistry.

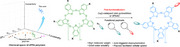

## 1 Introduction

Synthetic polymers are the most important compositions of modern materials. The appealing chemical and physical properties of synthetic polymers are determined by chemical structures, including molecular weight, backbone connections, and side chain functionalities. Analogous to the concept “Chemical Space” from small molecule chemistry [1, 2, 3] which is the sum of both reported and proposed chemical structures, exploring the unoccupied space of each designate polymer type via diversifying monomer structures [4] and side chain modifications [5] will be valuable for realizing new properties and application potentials. However, the availability of those structures is hampered by lacking feasible synthetic approaches.

Polyphthalaldehyde (PPA) is a special type of polyaldehyde with backbone comprised of alternating C and O atoms [6]. The polyacetal structure endows PPA stimuli‐responsive depolymerization properties, where the depolymerization process can be triggered by acid, heat, and mechanical force [7]. PPA can be synthesized in both linear and cyclic forms via different polymerization processes from *ortho*‐phthalaldehyde (OPA) monomers. Linear PPA molecules are prepared through living anionic polymerization followed by end group capping, which have been widely used as responsive materials like photoresists and lithography medium [8, 9, 10, 11, 12]. Functional groups and even hydrophilic functional polymeric blocks can be easily incorporated during either the initiation or the end group capping step, using functionalized initiators or electrophiles respectively [13, 14, 15, 16]. On the contrary, cyclic polyphthalaldehyde (cPPA) with endcap‐free macrocyclic structures is much more challenging for functionalization. The synthesis of cyclic polyphthalaldehyde (cPPA) was pioneered by Aso and Tagami in 1967 [17], while the macrocyclic structure and the unique cationic ring‐expansion polymerization mechanism were recently clarified by Moore [18, 19] and Kohl [20]. Many efforts have been devoted to the synthesis of high molecular weight unsubstituted cPPA molecules with the number average molecular weight (*M*_n_) ranging from less than 100 kDa (DP_n_ ∼ 750) to more than 400 kDa (DP_n_ ∼ 3000) [18, 19, 20, 21, 22]. Meanwhile, the solvent/thermal processing [23, 24], microcapsule formation and payload release [25, 26, 27], and applications based on the triggered depolymerization under heat [28, 29, 30, 31], photo [32, 33], and mechanical [34] stimuli have been studied. Unfortunately, introducing functionalities via polymerization of modified OPA monomers are quite difficult due to the thermodynamic features of the cationic polymerization process, in that the polymer elongation is only marginally favored and sensitive to substitutions. Moreover, the Lewis basic heteroatoms in functional groups may block the cationic polymerization via binding and deactivating Lewis acid catalysts. In 2019, McNeil et al. reported the synthesis of functionalized cPPA molecules from substituted OPA monomers and revealed the relationship between OPA substitutions and ceiling temperature (T_c_) [35]. Introducing electron‐withdrawing groups (e.g., tetrafluoro, ester, and imide) led to increased T_c_ and thermostability, thereby yielding increased molecular weight. In 2022, Ober et al. incorporated complex photoacid generators [36] into OPA monomers and synthesized cPPA molecules with photo‐triggered depolymerization property, where higher molecular weight (up to 5.4 kDa) can only be achieved through copolymerization with bromine‐substituted OPA [37]. Functional groups facilitating water solubility like amine, guanidine, alcohol and carboxylic acid have not been successfully introduced to cPPA polymers, due to the incompatibility between nucleophilic heteroatoms and Lewis acid catalysts. Water solubility is a prerequisite of polymers that to be used in environmental and biology‐relevant senarios [38, 39]. Water‐soluble conjugated polymers have been applied in biosensing and therapy [40, 41], while the cationic dendrimers have been applied in gene and drug delivery [42]. Unfortunately, no water‐soluble cPPA has been achieved, which totally hampers the exploration of the pH‐responsible depolymerization property of cPPA in acidic biological microenvironment [43]. The broad chemical space of cPPA polymers bearing complex hydrophilic functional modifications like sugar and peptides is still waiting for exploration, which requires employing new synthetic strategies.

**FIGURE 1:**
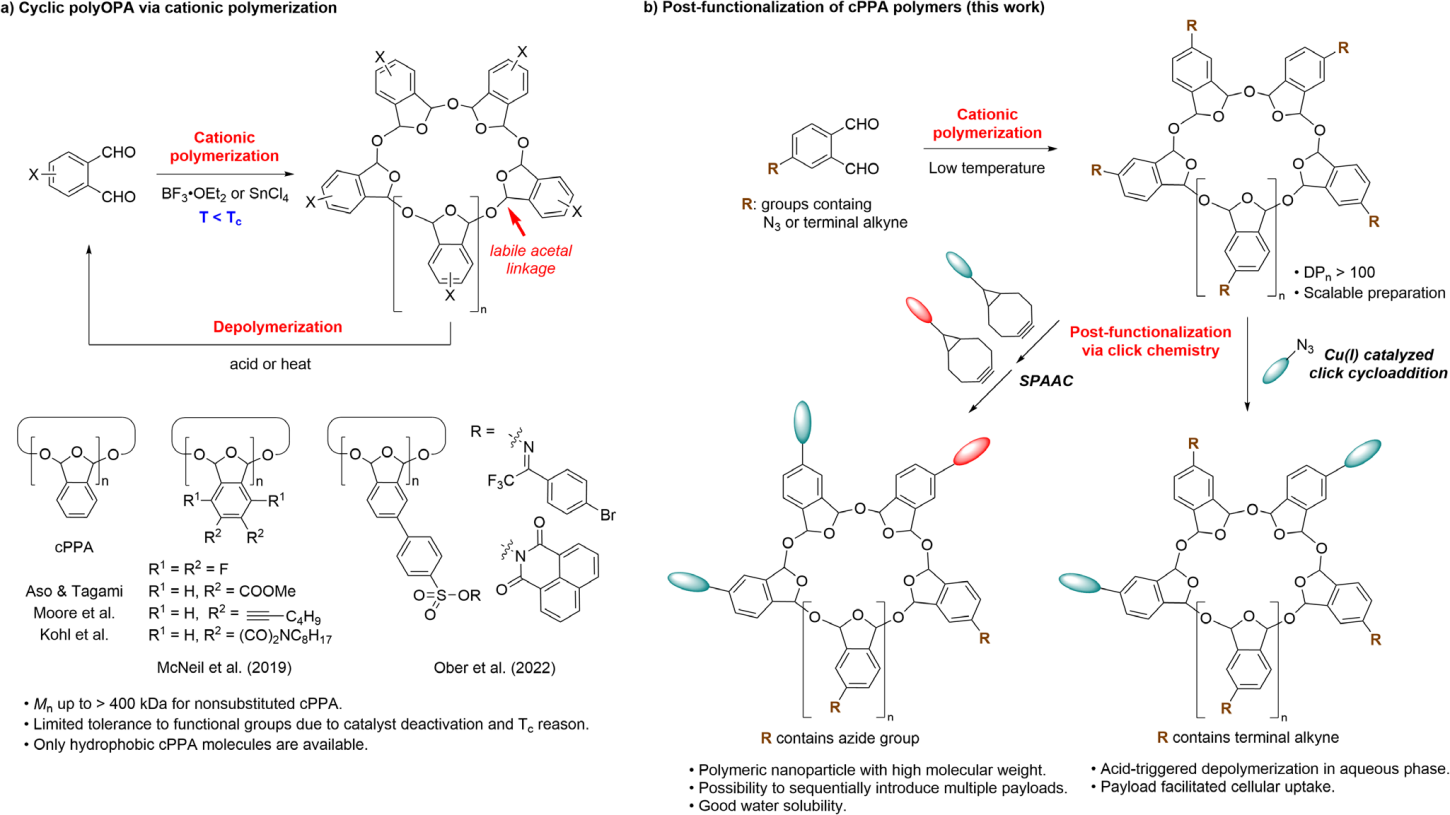
Reported synthesis of cyclic polyphthalaldehyde polymers via cationic polymerization (a) and design of post‐functionalization approach in current study (b).

To overcome the limitation of cationic cyclic polymerization of OPA and expand the chemical space of cPPA, we conceived of a post‐functionalization approach (Figure 1b). Post‐functionalization has been widely applied to the construction of functional polymers [44, 45, 46]. In our cPPA case, we hypothesized that the OPA monomer modified by clickable groups like alkyne and azide could polymerize at low temperature to afford high molecular weight polymers, then complex functional groups could be installed via Cu(I) catalyzed click 1,3‐dipole cycloaddition [47] or strain‐promoted alkyne‐azide cycloaddition (SPAAC) [48] without disruption of the macrocyclic backbone. Herein, we report the synthesis of water‐soluble functional cPPA derivatives with high molecular weight via post‐functionalization, the acid‐triggered depolymerization, and the cellular uptake behavior of water‐soluble cPPA modified with cell‐penetrating peptide.

## 2 Results and Discussion

**FIGURE 2:**
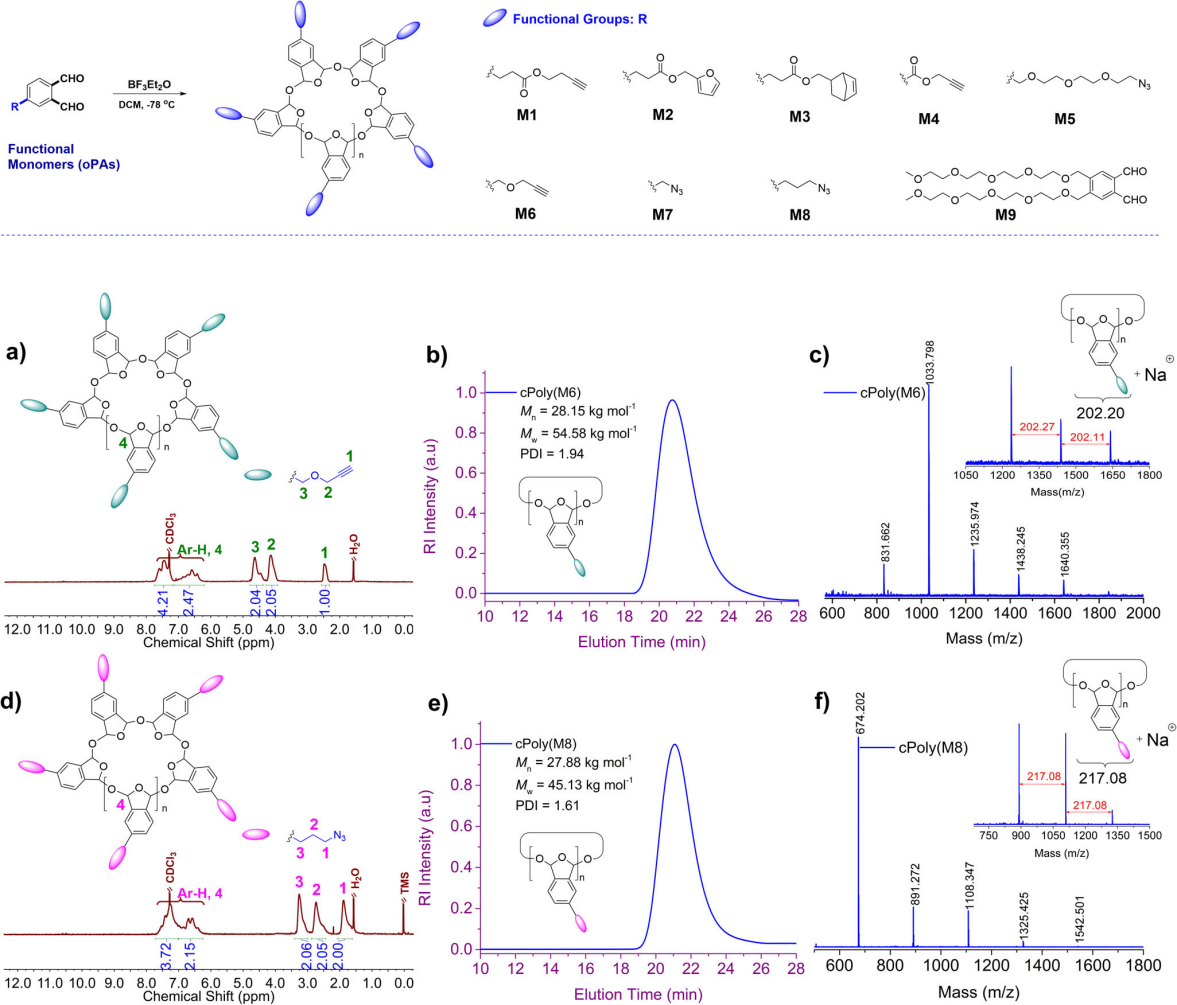
Synthesis and characterizations of cPPAs. a) Synthetic scheme of cPPAs. b,e) ^1^H‐NMR (in CDCl_3_) spectra of **cPoly(M6)** and **cPoly(M8)**. c,f) GPC spectra of **cPoly(M6)** and **cPoly(M8)**. d,g) MALDI‐TOF MS spectra of **cPoly(M6)** and **cPoly(M8)**.

To test our hypothesis, we first investigated the cationic polymerization of monomers **M1** to **M9** (Figure 2a). **M1** to **M8** were modified with clickable groups (i.e., alkyne, azide, norbornene, and furan), while **M9** was directly modified with hydrophilic PEG chains. The monomers were synthesized by adapting reported procedures detailed in the supporting information [49, 50, 51, 52]. All the monomers were subjected to the standard cationic polymerization conditions (BF_3_•OEt_2_ as catalyst, −78°C in CH_2_Cl_2_) [18]. The polymerization reactions were quenched by pyridine, and the products were purified by precipitating into methanol before gel permeation chromatography (GPC) analysis. The results are summarized in Table S1 with their GPC traces presented in Figure 2b,e and Figures S1–S5. Most monomers (**M1**‐**M2** and **M6**‐**M8**) polymerized effectively to form cyclic polymers with weight average molecular weights (*M*_w_) ranging from 1.83 to 106.19 kg·mol^−1^. Monomers **M3** and **M4** generated lower molecular weight products (*M*_w_ < 3.0 kg·mol^−1^), illustrating the sensitivity of cationic polymerization to monomer structures. Monomers **M5** and **M9** failed to afford polymer products possibly due to the competitive coordination of oxygen atoms in PEG to the catalyst. Meanwhile, all the successful polymerizations showed relatively broad polydispersity index (PDI) values ranging from 1.23 to 2.26, which is consistent with the non‐living ring expansion mechanism [19, 20]. Currently, cationic polymerization of OPA remains a challenging process, and despite extensive efforts, we have not yet identified a method that offers improved control over the results.

As representative examples, **cPoly(M6)** bearing propargyl group and **cPoly(M8)** bearing azido group were obtained with *M*_w_ = 54.58 kg·mol^−1^ (DP_n_ ∼ 270) and 45.13 kg·mol^−1^ (DP_n_ ∼ 208), respectively (Figure 2b,e). In the ^1^H‐NMR spectra of **cPoly(M6)** and **cPloy(M8)**, the stronger ─O─CH─O─ proton signal at 6.59 ppm compared to 6.90 ppm indicated *cis*‐configured cyclic acetal was predominantly formed, which aligns well with previous reported preference of *cis*‐rich polymers under cationic polymerization conditions (Figure 2a,d) [53]. Other assigned aliphatic and aromatic proton signals showed integration ratios consistent with theoretical numbers. In the MALDI‐TOF MS spectra of **cPoly(M6)** and **cPoly(M8)** (Figure 2c,f), a series of main peak were separated by an average interval of 202.17 and 217.08, respectively in accordance with the molar mass of substituted phthalaldehyde repeating units. Taking **cPoly(M6)** as an example (Figure 2d), the peak at m/z = 1033.79 is assigned to species with a degree of polymerization of 5. This assignment is based on the calculation: 202.06 × 5 + 23 (Na⁺) = 1033.30, which aligns well with the measured m/z value. These results support the cyclic structure of the polymer, consistent with findings reported in the literature [18]. No high m/z peak was observed due to the depolymerization of cPPAs during the measurement under laser radiation.

**FIGURE 3:**
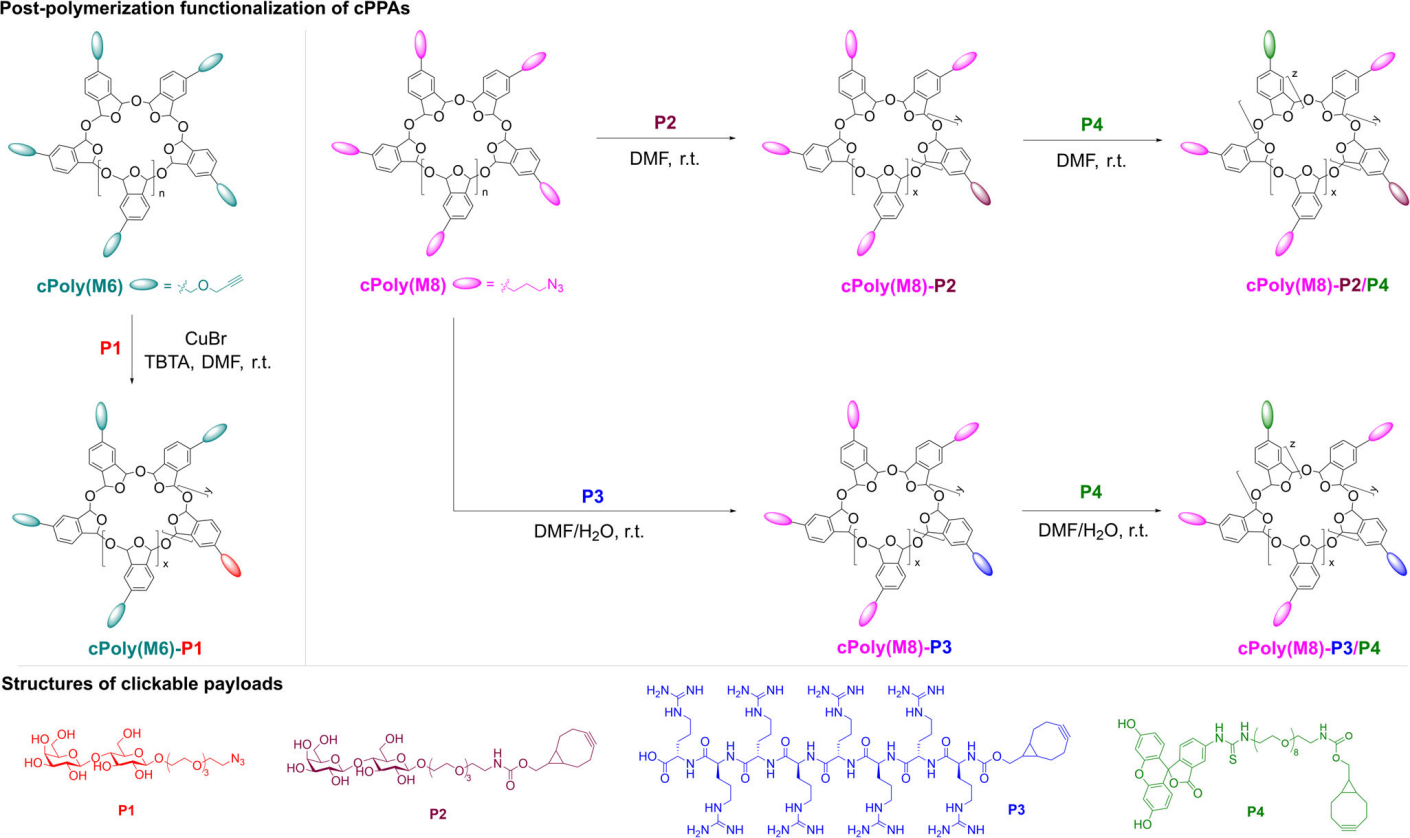
Synthesis of water‐soluble cPPA polymers via Cu(I)‐catalyzed click cycloaddition or SPAAC.

**FIGURE 4:**
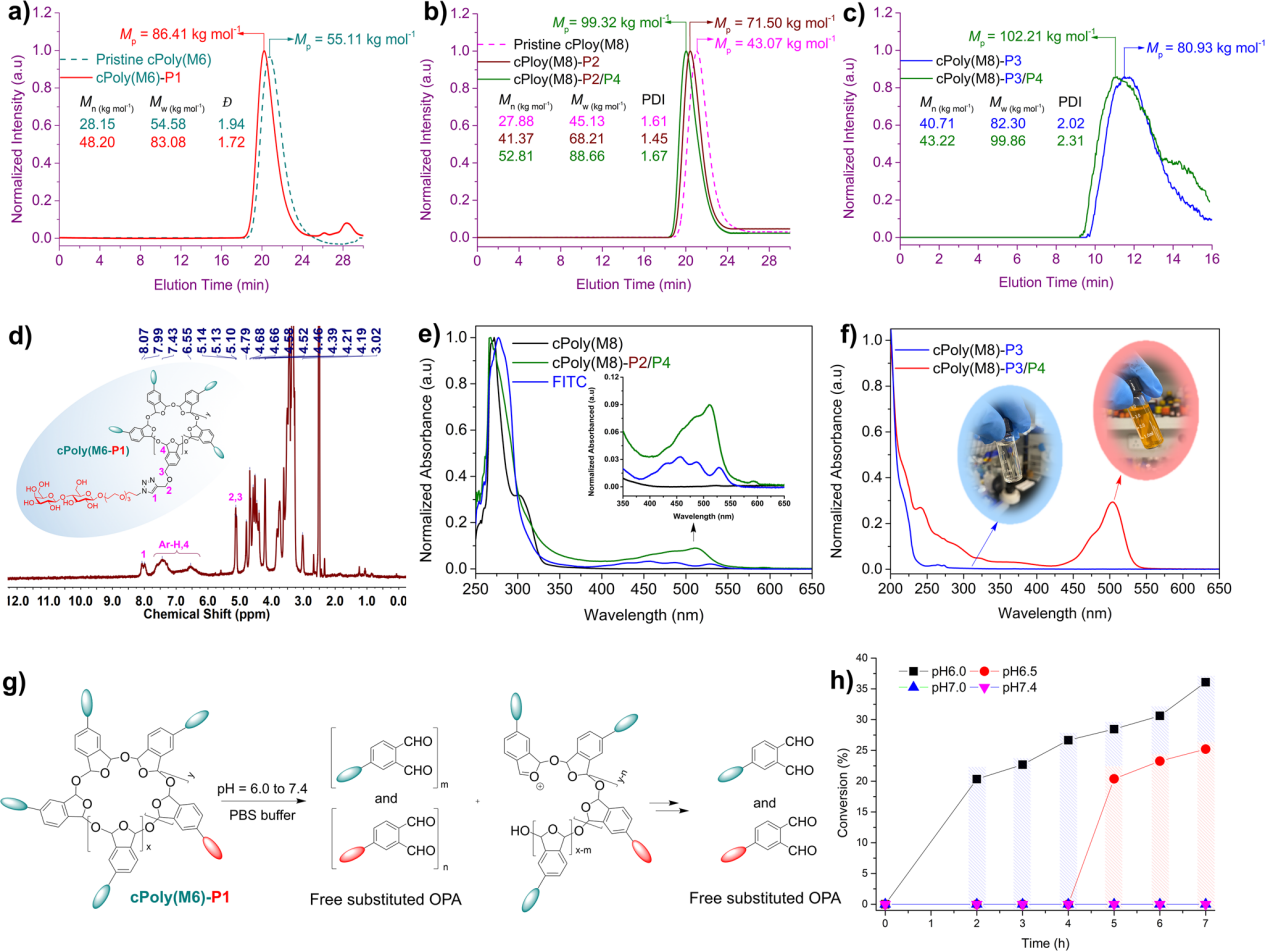
Characterizations of post‐functionalized cPPA polymers. a) GPC curves of **cPoly(M6)** and **cPoly(M6)‐P1**, *M*_w_, *M*_n_, and *M*_p_ determined by GPC in DMF (PS as calibration standard). b) GPC curves of **cPoly(M8)**, **cPoly(M8)‐P2**, and **cPoly(M8)‐P2/P4**, *M*_w_, *M*_n_, and *M*_p_ determined by GPC in DMF (PS as calibration standard). c) GPC curves of **cPoly(M8)‐P3** and **cPoly(M8)‐P3/P4**, *M*_w_, *M*_n_, and *M*_p_ determined by GPC in water (PEG as calibration standard). d) ^1^H‐NMR (in DMSO‐*d*_6_) spectrum of **cPoly(M6)‐P1**; e) UV–vis spectra of **cPoly(M8)**, **cPoly(M8)‐P2/P4** and FITC in DMF with concentration at 3.0 × 10^−6^ mg/mL. f) UV–vis spectra of **cPoly(M8)‐P3** and **cPoly(M8)‐P3/P4** in water with concentration at 7.0 × 10^−6^ mg/mL. Inset photos are **cPoly(M8)‐P3** and **cPoly(M8)‐P3/P4** in water solution, respectively. g) Schematic illustration of acid‐triggered depolymerization of water‐soluble **cPoly(M6)‐P1**. h) Depolymerization outcomes of **cPoly(M6)‐P1** in different 1x PBS buffers: pH = 6.0 (black line), pH = 6.5 (red line), pH = 7.0 (blue line) and pH = 7.4 (magenta line). See Table S3 for details.

Next, we tested the post‐functionalization of **cPoly(M6)** and **cPoly(M8)** (Figure 3). Here we focused on the introduction of hydrophilic functionalities, which is strongly required by the potential biological application but totally incompatible with cationic polymerization. Given the inherent instability of cPPAs, we opted to employ azide‐alkyne click cycloadditions under mild Cu(I) catalysis or Cu‐free conditions that have been widely used in polymer chemistry [47, 48, 54]. Thus, we synthesized lactose‐derived hydrophilic payloads **P1** and **P2** bearing azide and strained bicyclo[6.1.0]nonyne (BCN) groups, respectively. Meanwhile, we prepared another two BCN‐based payloads: **P3** bearing oligoarginine octapeptide which could provide water solubility as well as cell‐penetrating effects [55, 56], and **P4** bearing FITC for fluorescent visualization of modified polymers. In the functionalization of **cPoly(M6)** with **P1** (0.5 equiv based on alkyne group), when CuSO_4_/sodium ascorbate was used as catalyst in DMF/H_2_O medium, no product was obtained due to the depolymerization under acidic conditions generated during the reduction of Cu(II) by ascorbate [57]. A successful click reaction was achieved later using CuBr/tris[(1‐benzyl‐1H‐1,2,3‐triazol‐4‐yl)methyl]amine (TBTA) catalyst in DMF. After 24 h reaction, the product was purified by dialysis (molecular weight cutoff 3000 g·mol^−1^) to remove unconsumed payloads, and fully water‐soluble polymer **cPoly(M6)‐P1** was obtained in solid form after lyophilization. GPC analysis showed the increase of molecular weight from *M*_w_ = 54.58 kg·mol^−1^ to *M*_w_ = 83.08 kg·mol^−1^, indicating installation of ∼52 copies of sugar payloads with ∼218 alkyne left. Meanwhile, new proton signals at 8.07 and 5.13 ppm originating from triazole as well as broad peaks within the range 3.03–5.00 ppm assigned to carbohydrate and PEG spacer in the ^1^H NMR spectrum verified the chemical structure (Figure 4d). In the functionalization of **cPoly(M8)**, the SPAAC under mild Cu‐free conditions was leveraged. Payloads **P2** and **P3** (0.48 equiv based on azido group in each case) were subjected to SPAAC the with **cPoly(M8)** in DMF and DMF/H_2_O, respectively. After 8 h reaction at ambient temperature and dialysis, products **cPoly(M8)‐P2** (*M*_w_ = 68.21 kg·mol^−1^) and **cPoly(M8)‐P3** (*M*_w_ = 82.30 kg·mol^−1^) were obtained. Around 33 and 16 copies of **P2** and **P3** were installed respectively as indicated by GPC analysis (Figure 4b,c), and the unconverted azido groups were evidenced by observing 2069 cm^−1^ peak in FT‐IR measurement (Figure S6). Furthermore, the mild SPAAC conditions and the incomplete azido consumption allowed us to introduce more than one payload to the same polymer, which could increase the structural diversity of cPPA polymers yielding multifunctional constructs. Specifically, the purified **cPoly(M8)‐P2** and **cPoly(M8)‐P3** were further modified by FITC payload **P4** (0.35 equiv based on the total azido group), and the double‐modified cPPA polymers **cPoly(M8)‐P2/P4** (*M*_w_ = 88.66 kg·mol^−1^, 21 copies of **P4**) and **cPoly(M8)‐P3/P4** (*M*_w_ = 99.86 kg·mol^−1^, 18 copies of **P4**) were formed. Introduction of fluorophore led to the appearance of absorption band ranging from 400 to 550 nm (*E*_max_ = 500 nm) in consistent with FITC [58] in the UV–vis absorption spectra (Figure 4e) and clear change of the solution color (Figure 4f). Other examples of post‐functionalized water‐soluble polymers and their analysis results are provided in Figures S8–S10.

cPPA is acid‐responsive under the catalysis of photochemically generated [27, 29, 37] or thermally released acids [28] and gives rise to monomers through an unzipping depolymerization [59]. However, the effect of mild acidic conditions on the depolymerization behavior in aqueous media is totally unrevealed due to the unavailability of water‐soluble cPPA derivatives. With the water‐soluble cPPA polymers in hand, we evaluated the acid‐triggered depolymerization in aqueous medium. Polymer **cPoly(M6)‐P1** was subjected to PBS buffers with variable pH values (i.e., 6.0, 6.5, 7.0, 7.4) at 37°C, and the progress of the depolymerization was monitored by ^1^H NMR analysis. Since released OPA monomers can form cyclic hydrate in aqueous media, identical amount of reaction mixture was taken out at the designated time points and lyophilized to regenerate dialdehyde species and checked by ^1^H NMR in DMSO‐*d*_6_ via measuring the growth of two aldehyde proton signals near 10.50 ppm using 1,3‐dioxane as internal standard (see Supporting Information for details). As illustrated in Figure 4g,h, Table S3 and Figure S11, at pH 6.0, the appearance of aldehyde signals was observed after the first 2 h, and the conversion gradually achieved 36% after 7 h (setting the signal intensity of fully depolymerized sample as 100%). The depolymerization was much slower at pH 6.5, where 20% conversion was observed after 5 h and slightly increased thereafter. At pH 7.0 and 7.4, no depolymerization happened within the time range we monitored. Additionally, we also attempted to monitor the depolymerization of **cPoly(M8)‐P4** in water at pH = 6.0 and 5.5 over different time points using GPC with water as the eluent. However, as shown in Figure S16, the resulting product became insoluble and precipitated out in water after depolymerized, which hindered the water phase GPC analysis. We further investigated the depolymerization of **cPoly(M6)** in a THF/water mixture at pH 6.0 by monitoring the reaction at 2 and 6 h by GPC using THF as the eluent. As presented in Figure S17, we clearly observed the gradual decrease of the molecular weight of **cPoly(M6)** with reaction time increased caused by depolymerization. These results indicated that our functionalized cPPA polymers can survive the normal extracellular environment and undergo acid‐triggered depolymerization in acidic subcellular organelles and tumor microenvironment.

**FIGURE 5:**
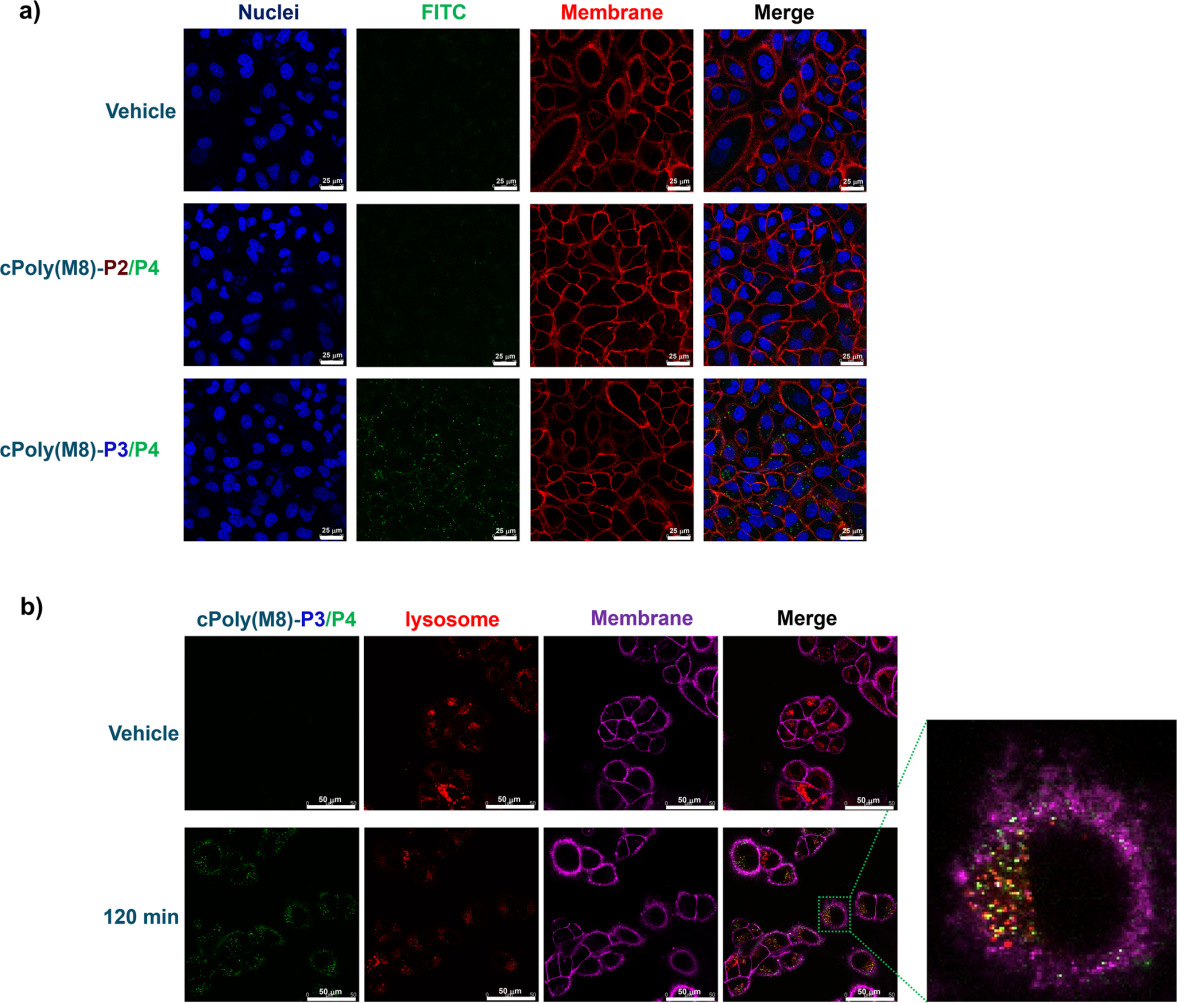
Cellular uptake of water‐soluble cPPA molecules visualized by confocal imaging. a) Confocal images of A549 cells treated with **cPoly(M8)‐P2/P4** (row 2) and **cPoly(M8)‐P3/P4**. (row 3). No cPPA was added in control group (row 1). Scale bars: 25 µm. b) Confocal images of A549 cells treated with **cPoly(M8)‐P3/P4** followed by lysosome staining (LysoTracker Red). Scale bars: 50 µm.

Encouraged by the above results, we investigated the interaction of water‐soluble cPPA polymers with cells. First, we examined the cellular uptake capability of FITC‐modified polymers **cPoly(M8)‐P2/P4** and **cPoly(M8)‐P3/P4**. The A549 lung cancer cells were incubated with the two polymers (50 nm in each case) respectively at 37°C for 2 h. Confocal images of treated cells are displayed in Figure 5a. It was found that the fluorescent signal from FITC was only observed in the cells treated with oligoarginine‐modified **cPoly(M8)‐P3/P4** (Figure 5a, Row 3), while no fluorescence was observed in **cPoly(M8)‐P2/P4** treated cells (Figure 5a, Row 2). This result indicates that the cell‐penetrating effect of (Arg)_8_ tag via electrostatic interaction with negatively charged cell membrane is the determining factor of cellular uptake [60, 61]. The cellular uptake of **cPoly(M8)‐P3/P4** showed concentration and time dependence. Increasing the polymer concentration to 2 µm led to faster uptake and stronger intracellular fluorescent signal at 2 h time point (Figures S18 and S19). No FITC signal was observed on the cell membrane, which indicated that the polymer was uptaken without depolymerization and free OPA release. Meanwhile, we treated A549 and normal lung fibroblast cells (CCD‐19Lu) respectively with OPA‐FITC molecules (equal to the released monomer) at 50 nm in PBS buffer (pH 7.4) for 15 min. In confocal images, only the cell surface was fluorescently labeled in both cases (Figure S21), which is in accordance with the reported cell surface reactivity of free OPA probes [62]. No cytotoxicity of **cPoly(M8)‐P3** was observed within 48 h at the polymer concentrations ranging from 0.11 to 15 µg·mL^−1^ (Figure S20).

To understand the uptake mechanism, after incubating A549 cells with **cPoly(M8)‐P3/P4** at 50 nm concentration for 2 h, lysosomes were stained for Confocal imaging. As illustrated in Figure 5b, colocalization of fluorescent signals from FITC and LysoTracker was clearly observed, indicating that the (Arg)_8_ modified polymer was untaken via endocytosis. We assume that the endocytosis of the polymeric nanoparticle (∼4.3 nm size in water without FITC installation as measured by dynamic light scattering, Figure S7) was induced by the interaction between the positively charged particle surface and negatively charged cell membrane. After entering the early endosome (pH 6.5), depolymerization will start and become much faster in late endosome/lysosome as pH decreased to 4.5–5.5. The released free OPA moieties will then label cellular proteins via OPA‐amine two‐component reaction and retained in the cytoplasma [49, 63].

**FIGURE 6:**
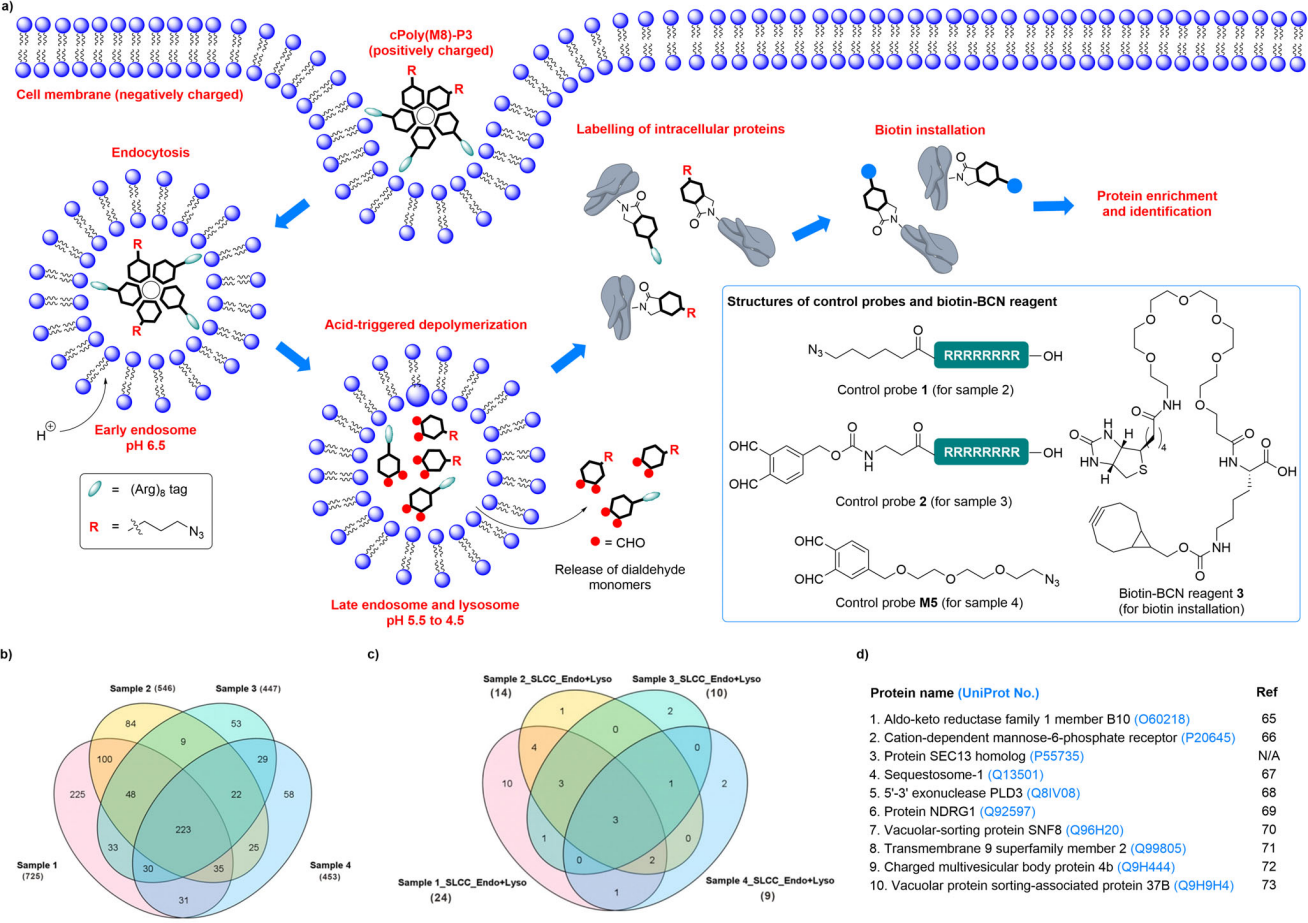
Verification of the endocytosis/depolymerization pathway via identification of labeled intracellular proteins. a) Illustration of the cellular uptake of **cPoly(M8)‐P3**, intracellular depolymerization and protein labeling. b) Venn diagram showing total identified proteins in the supernatant from samples 1–4. c) Venn diagram showing potential endosome and lysosome proteins in the supernatant from samples 1–4. d) List of identified unique endosome/lysosome proteins from sample 1. The Venn diagram was generated using TBtools‐II software [78].

Since the intracellular depolymerization of cPPA in lysosome cannot be monitored directly, we designed an indirect approach by identifying the cellular proteins labeled by the released OPA. Here, we leveraged the partial payload installation during **cPoly(M8)‐P3** synthesis, where the left azido groups could be used for enrichment of labeled proteins. A549 cells were treated with **cPoly(M8)‐P3** (sample 1), (Arg)_8_‐N_3_ **1** (sample 2), (Arg)_8_‐OPA **2** (sample 3), and **M5** (sample 4) respective, where samples 2–4 served as controls. After 48 h incubation at 2 µm concentration, the cell lysates were treated with biotin‐BCN **3**, then the labeled proteins were enriched by streptavidin magnetic beads. The enriched proteins separated by SDS‐PAGE were subjected to in‐gel digestion and LC‐MS/MS for identification (see Supporting Information for details). As illustrated in Figure 6b, 725, 546, 447, and 453 proteins were identified in samples 1–4, respectively. The high protein recovery from samples 2–4 could be attributed to the thiol‐yne reaction of **3** with proteomic Cys residues [64]. The identified proteins were further screened using UniProt data category subcellular location cellular component (SLCC) showing endosome/lysosome presence. As shown in Figure 6c, 10 unique proteins from sample 1 were identified, where the endosome/lysosome location of 9 proteins has been reported (Figure 6d and Table S4) [65, 66, 67, 68, 69, 70, 71, 72, 73]. These findings provide additional evidence for the endocytosis‐depolymerization mechanism. The labeling of other cellular proteins indicates that the some of the released OPA can escape lysosome. Since the particle size as small as < 10 nm is not favored for endocytosis as reported [56, 74, 75], the successful uptake of our cyclic polymer could possibly be attributed to the formation of larger aggregates via the interaction with negatively charged serum proteins from the cell culture media [76, 77].

## 3 Conclusion

In summary, we developed a post‐functionalization approach to synthesize functionalized cyclic polyphthalaldedye (cPPA) polymers with water solubility. The *ortho*‐phthalaldehyde monomers bearing clickable groups like alkyne and azide were polymerized under cationic polymerization conditions, and the high molecular weight products were further modified by functional payloads via click chemistry. This approach allowed us to overcome the intrinsic limitation of cationic polymerization (i.e., sensitivity of T_c_ to substitutions and incompatibility with polar groups containing heteroatoms) and substantially expand the chemical space of cPPA polymers. The capability to achieve both high molecular weight and high structural diversity makes this approach attractive for preparing functionalized cPPA polymers bearing complex payloads (not limited to water‐solubilizing groups) required for different application scenarios.

The generation of water‐soluble cPPA polymers facilitated the study of pH‐responsive depolymerization in aqueous media, and the polymer modified with cell‐penetrating peptides could undergo cellular uptake via endocytosis and acid‐triggered depolymerization in late endosome/lysosome. We believe this study could provide new opportunities for realizing the biological application potentials of cPPA polymers, which is largely unexplored.

## Conflicts of Interest

The authors declare no conflict of interest.

## Author Contributions

L.H. and X.G. did polymer synthesis and characterization. H.Z. carried out biological studies. Q.C. conducted proteomic study. H.L. and X.L. conceived the idea and supervised the overall project. L.H., H.L., and X.L. wrote the manuscript.

## Supporting information




**Supporting File**: advs73514‐sup‐0001‐SuppMat.pdf.

## Data Availability

The data that support the findings of this study are available in the supplementary material of this article.
