# Supplementary material for: Expanding the Chemical Space of Cyclic Polyphthalaldehyde via Post‐Functionalization
Source: Adv Sci (Weinh). 2025 Dec 23;13(13):e22465. doi: 10.1002/advs.202522465 (PMC12955877; doi:10.1002/advs.202522465)
Supplement: Supplementary file 1 — Supporting File: advs73514‐sup‐0001‐SuppMat.pdf. [file ADVS-13-e22465-s001.pdf]

Expanding the Chemical Space of Cyclic Polyphthalaldehyde via Post-functionalization

Liting He, Haiyan Zhou, Qiushi Chen, Xing Guo, Han Liu, and Xuechen Li**

L. He, H. Zhou, Q. Chen, X. Guo, L. Han, X. Li
Department of Chemistry, the State Key Laboratory of Synthetic Chemistry
The University of Hong Kong
Hong Kong SAR, 999077, P. R. China
E-mail: liuhan@hku.hk, xuechenl@hku.hk

Q. Chen
Laboratory for Synthetic Chemistry and Chemical Biology Limited, Hong Kong Science Park
Pak Shek Kok, Hong Kong SAR, 999077, P. R. China

X. Li
Materials Innovation Institute for Life Sciences and Energy (MILES), HKU-SIRI
Shenzhen 518000, P. R. China; Shanghai-Hong Kong Joint Laboratory in Chemical Synthesis, Shanghai Institute of Organic Chemistry, University of Chinese Academy of Sciences, Chinese Academy of Sciences, 345 Lingling Road, Shanghai 200032, P. R. China

Corresponding author email: liuhan@hku.hk, xuechenl@hku.hk

Table of content

1. General information 3

2. General procedure for the synthesis of building blocks and *o*-PA monomers. 4

2.1 General procedure for building blocks synthesis 4

2.2 General procedure for monomer synthesis 8

2.3 Synthesis of payloads (P1 to P4) and compound 27, 28, 30 and 32 17

3. Synthesis of cyclic polyphthalaldehydes (cPPAs) 24

4. Synthesis of Water-soluble cPPAs 27

4.1 Synthesis of **cPoly(M6)-P1** 27

4.2 Synthesis of **cPoly(M8)-P2**, **cPoly(M8)-P2/P3**, **cPoly(M8)-P4** and **cPoly(M8)-P3/P4** 27

5. Depolymerization of cPoly(M6)-P1 in different PBS buffers 28

6. Cellular uptake study of cPoly(M8)-P2/P3 and cPoly(M8)-P3/P4 38

6.1 Cell culture 38

6.2 Cell viability assay 38

6.3 Cellular uptake assay 38

6.4 *o*-PA-FITC for cell labeliing 39

7. Pulldown experiment 41

7.1 Processing of treated cell lysate for sodium dodecyl-sulfate polyacrylamide gel electrophoresis (SDS-PAGE) and subsequent in-gel digestion 41

7.2 Liquid chromatography-mass spectrometry (LC-MS) for proteomics and mass spectrometry (MS) data analysis 42

8. UPLC-Chromatogram and MS-Spectrum 48

9. ^{1}H- and ^{13}C-NMR Spectra of Small Molecular 62

1. General information

All commercially available amino acids and coupling reagents (purchased from Aldrich and CS Bio) were used without further purification. All solvents in reagent grade (RCI) or HPLC grade (DUKSAN) were used without purification. Anhydrous dichloromethane (DCM) and tetrahydrofuran (THF) were freshly distilled from calcium hydride (CaH_2) before use.

^{1}H and ^{13}C nuclear magnetic resonance (NMR) spectra were recorded using a Bruker AVANCE-400 NMR spectrometer or Bruker AVANCE-500 NMR spectrometer; chemical shifts are recorded in parts per million (ppm) using solvent resonance as the internal standard. Gel permeation chromatography (GPC) analyses were performed on three different systems. Most GPC analyses were carried on a TOSOH Bioscience EcoSEC HKC08320 GPC with differential refractive index (RI) detector equipped with a TSKgel GMHHR-M colum and run at a flow rate of 0.35 mL/min at 40°C using HPLC grade DMF, the instrument was calibrated using linear monodisperse PS standards. GPC analyses of water-soluble polymers were carried on an Agilent 1260 GPC analysis system with differential refractive index (RI) detector equipped with two TSKgel column and run at flow rate of 1 ml/min at 30 °C using monomer disperse poly(ethylene glycol) (PEG) as calibration standard. Spectrophotometer Lambda 750 (PerkinElmer) was used for UV-vis measurements. FTIR spectra were recorded using Fourier-transform infrared (FTIR) spectrometer, PerkinElmer. Dynamic light scattering (DLS) was taken by Zetasizer Nano ZS90 from Malvern Instruments (UK). MALDI-TOF mass spectra were recorded with a Bruker Daltonics UltrafleXtreme MALDI TOF at the Mass Spectrometry Facility, Department of Chemistry, The University of Hong Kong. The instrument was operated in reflection mode and positive ions were detected. The ions were accelerated under a potential of 20 kV, and PEG 1500 as external calibration. Polymer samples and salt (NaI) were prepared at 10 mg/mL in THF, respectively, and took 10μL of each mixed together, then took 1 μL spotted on the MALDI sample plate and dried under air. Then, 1 μL of 2,5-dihydroxybenzoic acid (DHB, at 10 mg/mL in THF) was spotted on top of the polymer/salt mixture and dried under air. Preparative HPLC was performed on a Waters system, using a Vydac 218TPTM C18

column (10 μm, 30 × 250 mm) at a flow rate of 20 mL/min. Mobile phases of HPLC used are as followed: Solvent A: 0.1% TFA (v/v) in acetonitrile; Solvent B: 0.1% TFA (*v*/*v*) in water. Waters UPLC H-class system equipped with an ACQUITY UPLC photodiode array detector and a Waters SQ Detector 2 mass spectrometer using a Waters ACQUITY BEH C18 column (1.7 μm, 130 Å, 2.1 × 50 mm) at a flow rate of 0.4 mL/min. Mass spectra of samples were determined by using electrospray ionization (ESI) in positive mode.

2. General procedure for the synthesis of building blocks and OPA monomers.

2.1 General procedure for building blocks synthesis

Building block compounds such as Compounds 4 and 5 were synthesized following a modification of the procedure described in the literature, as shown in Scheme S1.[1,2,3]

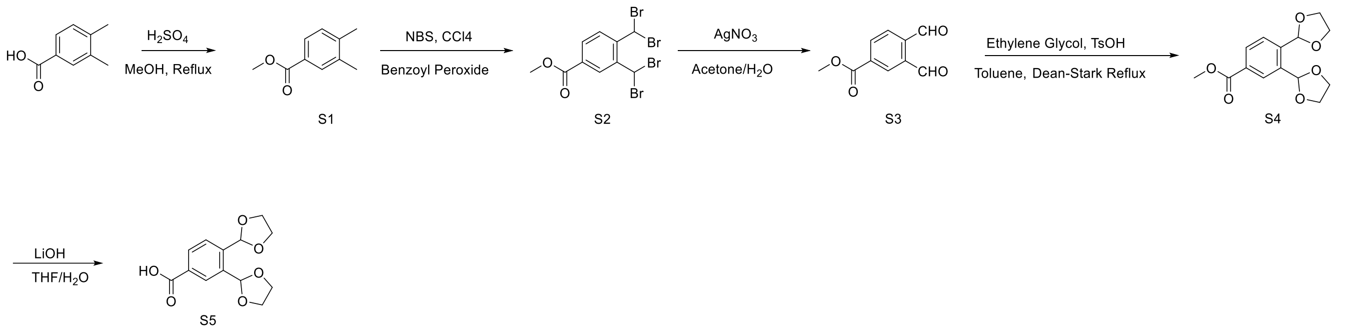

Scheme S1. The synthetic route of building block compounds.

Synthesis of compound S1

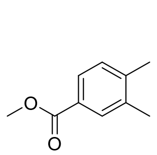

A solution of 3,4-dimethylbenzoic acid (30 g, 199.7 mmol) in 120 mL of methanol (MeOH) was prepared, then concentrated sulfuric acid (H_2SO_4) (2.0 mL, 0.01 eq.) was added to the solution. The mixture was then placed in an oil bath and heated to reflux (boiling) for 8 hours. After the reflux period, the mixture was cooled to room temperature. The solvent was removed under vacuum to concentrate on the solution. The resulting residue was dissolved in 500 mL of ethyl acetate (EA). The solution was then washed

sequentially with sodium bicarbonate ($NaHCO_3$) solution, brine (saturated sodium chloride solution), and dried with anhydrous sodium sulfate (Na_2SO_4). The organic solution was concentrated under vacuum to obtain Compound S1 (32.5 g), which was used directly for the next step in the synthesis without any further purification.

Synthesis of compound S2

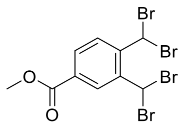

Compound S1 (32.5 g, 197.8 mmol) was dissolved in 600 mL of carbon tetrachloride (CCl_4). *N*-bromosuccinimide (NBS) (144.1 g, 813.9 mmol, 4.1 eq.) and benzoyl peroxide (4.08 g, 16.7 mmol, 0.085 eq.) were added to the solution. The reaction mixture was refluxed for 20 hours. Solids were filtered off and washed with diethyl ether (Et_2O) (2×400 mL). The organic layer solution was evaporated to yield crude Compound S2 as a yellow solid (93.8 g), which was used directly for the next step in the synthesis without any further purification.

Synthesis of compound S3

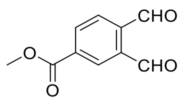

Compound S2 (93.8 g, 195.4 mmol) was dissolved in a 3.1 L mixture solution of acetone and water (*v*/*v*, 8/1), then silver nitrate ($AgNO_3$) (140 g, 822.8 mmol, 4.2 eq.) was added. The mixture was vigorously stirred at room temperature for 36 hours. The mixture was then filtered to remove some solids, and the remaining solution was concentrated to remove acetone. The concentrated solution was extracted with EA (3×900 mL). The combined organic layers were washed with water and brine and dried with Na_2SO_4. The organic solution was then concentrated to give a yellow solid product. The product was triturated in a mixture of EA and hexane (*v*/*v*, 1/50) five times. After drying, Compound S3 was obtained as a yellow solid powder (32.6 g), which was used directly for the next step in the synthesis without further

purification. The overall yield from Compound 1 to Compound S3 was 85%. It should be noted that the product on the TLC plate showed a band in EA/hexane (*v/v*, 1/3).^{1}H NMR (400 MHz, Chloroform-d) δ 10.65 (s, 1H), 10.52 (s, 1H), 8.64 (s, 1H), 8.43 (d, J = 9.5 Hz, 1H), 8.08 (d, J = 7.9 Hz, 1H), 4.03 (s, 3H). ^{13}C NMR (101 MHz, Chloroform-d) δ 191.66, 191.58, 165.04, 139.16, 136.43, 134.80, 134.48, 132.76, 130.78, 52.93.

Synthesis of compound S4

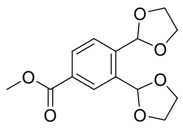

Compound S3 (2.2 g, 10.92 mmol) was dissolved in 250 mL of anhydrous toluene, *p*-toluenesulfonic acid (32 mg, 0.185 mmol) and ethylene glycol (6.16 mL, 109.20 mmol) were added. The mixture was refluxed using a Dean-Stark apparatus for 4 hours. After the reaction was cooled down to room temperature, triethylamine (Et_3N) (0.8 mL, 5.7 mmol) was added to quench the reaction. The mixture was then evaporated under vacuum, and the resulting residue was dissolved in EA. The organic layers were washed with saturated $NaHCO_3$ solution and brine, then dried with Na_2SO_4. The organic solution was evaporated to remove the solvent, yielding a residue. The residue was purified by flash column chromatography on silica gel using a mixture of EA and hexane (*v*/*v*, 1/3) as the eluent. Compound S4 was obtained as a yellow oil (2.45 g) with a yield of 80%. TLC (EA/hexane=1/3, *v*/*v*), Rf≈0.5. ^{1}H NMR (500 MHz, Chloroform-d) δ 8.32 (s, 1H), 8.07 (d, J = 9.6 Hz, 1H), 7.74 (d, J = 8.1 Hz, 1H), 6.28 (s, 1H), 6.28 (s, 1H), 4.20 – 4.06 (m, 8H), 3.94 (s, 3H). ^{13}C NMR (126 MHz, Chloroform-d) δ 166.62, 140.86, 136.68, 130.73, 130.14, 127.51, 126.28, 100.34, 100.31, 65.41, 65.40, 52.20.

Synthesis of compound S5

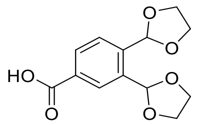

Compound S4 (2.0 g, 7.13 mmol) was dissolved in a mixture of tetrahydrofuran (THF) and water (3/1, v/v) at room temperature. A solution of lithium hydroxide (LiOH) (513 mg, 21.39 mmol) in water was slowly added to the mixture. The mixture was stirred at room temperature overnight. The reaction was then neutralized with 1 N hydrochloric acid (HCl) solution. The reaction mixture was extracted twice with EA. The combined organic layers were washed with brine, dried with Na_2SO_4, and evaporated to yield Compound S5 as a pale-yellow powder (1.73 g) with a yield of 91%. ^{1}H NMR (400 MHz, DMSO-d6) δ 8.13 (s, 1H), 7.98 (d, J = 8.0 Hz, 1H), 7.67 (d, J = 8.0 Hz, 1H), 6.11 (s, 1H), 6.10 (s, 1H), 4.14 – 3.92 (m, 8H). ^{13}C NMR (101 MHz, DMSO-d6) δ 141.02, 136.94, 130.23, 127.57, 126.99, 99.73, 99.63, 65.44, 65.41.

Synthesis of compound S6

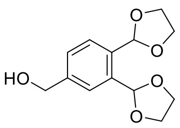

$LiAlH_4$ (0.69 g, 17.82 mmol, 2.5 eq.) was placed in a 100 mL oven-dried round-bottom flask and cooled in an ice bath under argon protection. Anhydrous THF (10 mL) was added slowly to the flask and stirred for 3 minutes. A solution of Compound S4 (2.0 g, 7.13 mmol) in 40 mL of anhydrous THF was added dropwise to the flask, and the mixture was stirred under 0 °C for 4 hours. After the reaction was completed, fresh water (0.69 mL) was added dropwise to quench the reaction, with great care and slowly, and the mixture was allowed to stir for 10 minutes. A solution of 15% sodium hydroxide (NaOH) (0.69 mL) was added to the reaction mixture, and the mixture was allowed to stir for 10 minutes. Then, 2.07 mL of water (3×0.69 mL) was added, and the mixture was diluted with 30 mL of THF and allowed to stir at room temperature for 8 hours. The mixture was dried with Na_2SO_4, filtrated with celite, and evaporated under vacuum to afford Compound S6 as a yellow oil (1.78 g) with a yield of 99%. TLC (EA/hexane=1/3, *v*/*v*) Rf≈0.2. ^{1}H NMR (400 MHz, Chloroform-*d*) δ 7.65 (d, *J* = 2.3 Hz, 1H), 7.63 (s, 1H), 7.40 (d, *J* = 8.0 Hz, 1H), 6.23 (s, 1H), 6.23 (s, 1H), 4.71 (s, 2H), 4.19 – 4.07 (m, 8H). ^{13}C NMR (101 MHz, Chloroform-*d*) δ 141.94, 136.25, 135.39, 127.44, 126.49, 124.53, 100.69, 100.58, 65.36, 65.31, 64.95.

Synthesis of compound S7

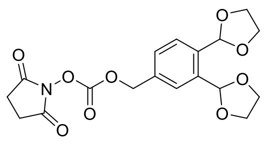

Compound S6 (5.40 g, 21.4 mmol) was dissolved in 100 mL of dry ACN. *N*,*N'*-Diisopropylethylamine (DIPEA) (11 mL, 64.2 mmol) was added to the mixture solution. The mixture was placed in an ice bath and stirred. *N*,*N'*-Disuccinimidyl carbonate (DSC) (6.03 g, 23.5 mmol) was added to the reaction mixture. The reaction was stirred under argon protection for 5 hours in the ice bath. After the reaction was completed, the mixture was directly concentrated under vacuum. The resulting residue was purified by flash column chromatography on silica gel using a mixture of EA and hexane (*v*/*v*, 1/1) as the eluent. Compound S7 was obtained as a white solid (5.40 g) with a yield of 64%. ^{1}H NMR (400 MHz, Chloroform-*d*) δ 7.71 (s, 1H), 7.69 (d, J = 2.2 Hz, 1H), 7.44 (d, J = 8.0 Hz, 1H), 6.26 (s, 1H), 6.26 (s, 1H), 5.35 (s, 2H), 4.20 – 4.04 (m, 9H), 2.85 (s, 4H). ^{13}C NMR (101 MHz, Chloroform-d) δ 168.55, 151.55, 137.25, 136.92, 134.04, 128.92, 126.74, 126.17, 100.48, 100.34, 72.28, 65.37, 65.34, 25.46.

2.2 General procedure for monomer synthesis

Synthesis of M1

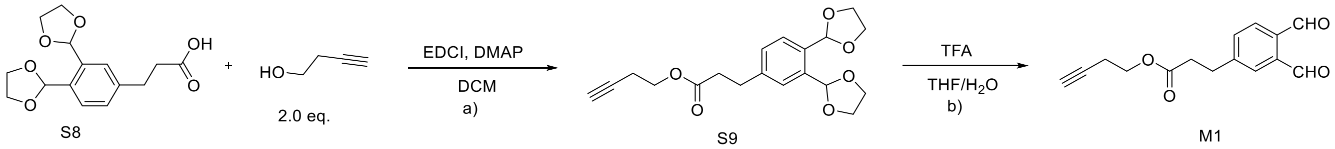

a) Compound S8 was prepared according to the literature reported.[1,4] Compound S8 (1.0 g, 3.4 mmol, 1.0 eq.) was dissolved in 50 mL of anhydrous DCM. *N*-(3-dimethylaminopropyl)-*N'*-ethylcarbodiimide hydrochloride (EDCI) (1.3 g, 6.8 mmol, 2.0 eq.) and 4-dimethylaminopyridine (DMAP) (0.21 g, 1.7 mmol, 0.5 eq.) were added to the solution and stirred for 10 minutes. 3-Butyn-1-ol (0.48 g, 6.8 mmol, 2.0 eq.) was added to the reaction mixture, and the reaction was stirred at room temperature for 16 hours. The mixture was concentrated under vacuum to remove the solvent, yielding a residue. The residue was

purified by flash column chromatography on silica gel using a mixture of EA and hexane (*v*/*v*, 1/3) as the eluent. Compound S9 was obtained as a yellow solid (0.85 g) with a yield of 72%. ^{1}H NMR (400 MHz, Chloroform-d) δ 7.58 (d, J = 7.9 Hz, 1H), 7.50 (s, 1H), 7.25 (d, J = 7.9 Hz, 1H), 6.21 (s, 1H), 6.21 (s, 1H), 4.23 – 4.03 (m, 10H), 3.00 (t, J = 7.8 Hz, 2H), 2.67 (t, J = 7.8 Hz, 2H), 2.53 (m, 2H), 2.02 (t, J = 2.7 Hz, 1H). ^{13}C NMR (101 MHz, Chloroform-d) δ 172.51, 141.40, 136.11, 134.17, 129.02, 126.45, 125.99, 100.72, 100.67, 69.94, 65.32, 65.30, 62.14, 35.59, 30.72, 18.95.

b) A 15 mL solution of trifluoroacetic acid (TFA)/H_2O/THF (1/1/1, *v*/*v*/*v*) was prepared. A 10 mL solution of Compound S9 (0.85 g, 2.4 mmol) in THF was added dropwise to the solution using a dropping funnel. The mixture was stirred at room temperature for 5 hours. The mixture was then diluted with water and extracted three times with EA. The combined organic layers were washed with brine and saturated $NaHCO_3$ solution and dried with Na_2SO_4. The organic solution was concentrated under vacuum, yielding a residue. The residue was purified by flash column chromatography on silica gel using a mixture of EA and hexane (*v*/*v*, 1/3) as the eluent. M1 was obtained as a yellow oil (0.58 g) with a yield of 92%. ^{1}H NMR (500 MHz, Chloroform-d) δ 10.59 (s, 1H), 10.50 (s, 1H), 7.94 (d, J = 7.8 Hz, 1H), 7.85 (s, 1H), 7.66 (d, J = 7.8 Hz, 1H), 4.21 (t, J = 6.7 Hz, 2H), 3.14 (t, J = 7.5 Hz, 2H), 2.77 (t, J = 7.5 Hz, 2H), 2.53 (m, J = 6.7, 3.4 Hz, 2H), 2.01 (t, J = 2.7 Hz, 1H). ^{13}C NMR (126 MHz, Chloroform-d) δ 192.31, 191.97, 147.26, 133.68, 131.90, 130.79, 70.02, 62.42, 34.71, 30.65, 18.97.

Synthesis of M2:

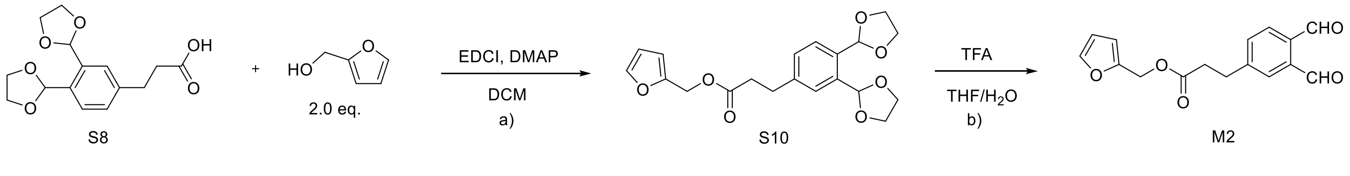

M2 was prepared following the same procedure for M1.

a) Compound S8 (1.0 g, 3.4 mmol, 1.0 eq.), EDCI (1.3 g, 6.8 mmol, 2.0 eq.), DMAP (0.21 g, 1.7 mmol, 0.5 eq.), furfuryl alcohol (0.67 g, 6.8 mmol, 2.0 eq.). After purified by silica gel column (EA/hexane=1/3, *v*/*v*) to afford Compound S10 (0.92 g, 72.3%). ^{1}H NMR (400 MHz, Chloroform-d) δ 7.56 (d, J = 7.9 Hz,

1H), 7.49 (d, J = 7.9 Hz, 1H), 7.44 (s, 1H), 7.22 (d, J = 7.9 Hz, 1H), 6.44 – 6.36 (m, 2H), 6.21 (s, 1H), 6.21 (s, 1H), 5.08 (s, 2H), 4.20 – 4.01 (m, 8H), 2.99 (m, 2H), 2.67 (m, 2H). ^{13}C NMR (101 MHz, Chloroform-d) δ 172.38, 149.43, 143.29, 141.37, 136.14, 134.15, 129.00, 126.44, 125.97, 110.67, 110.57, 100.73, 100.69, 65.31, 58.11, 35.57, 30.67.

b) After acidolysis of Compound S10 and purification by flash column chromatography on silica gel using a mixture of EA and hexane (*v*/*v*, 1/3) as the eluent, M2 was obtained as an oil (0.56 g) with a yield of 80%. ^{1}H NMR (500 MHz, Chloroform-d) δ 10.54 (s, 1H), 10.50 (s, 1H), 7.91 (d, J = 7.8 Hz, 1H), 7.80 (s, 1H), 7.60 (d, J = 7.8, 1H), 7.42 (d, J = 7.8Hz, 1H), 6.42 – 6.35 (m, 2H), 5.08 (s, 2H), 3.12 (t, J = 7.4 Hz, 2H), 2.76 (t, J = 7.4 Hz, 2H). ^{13}C NMR (126 MHz, Chloroform-d) δ 192.35, 191.97, 171.65, 149.16, 147.18, 143.39, 136.62, 134.70, 133.67, 131.73, 130.97, 110.88, 110.62, 58.25, 34.77, 30.66.

Synthesis of M3:

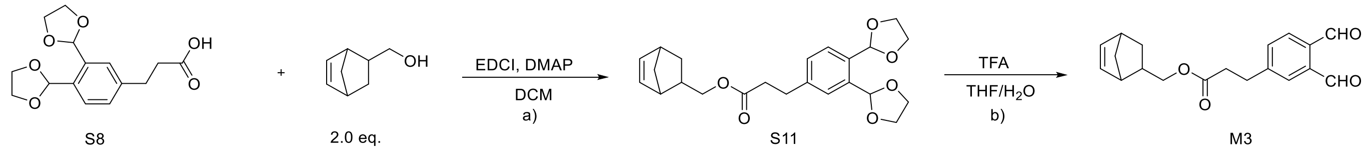

M3 was prepared following the same procedure for M1.

a) Compound S8 (1.0 g, 3.4 mmol, 1.0 eq.), EDCI (1.3 g, 6.8 mmol, 2.0 eq.), DMAP (0.21 g, 1.7 mmol, 0.5 eq.), 5-norbornene-2-methanol (0.84 g, 6.8 mmol, 2.0 eq.). After purified by silica gel column (EA/hexane=1/3, *v*/*v*) to afford Compound S11 (0.85 g, 62.5%). ^{1}H NMR (500 MHz, Chloroform-d) δ 7.58 (d, J = 7.9 Hz, 1H), 7.51 (s, 1H), 7.26 (d, J = 8.0Hz, 1H), 6.21 (s, 1H), 6.21 (s, 1H), 6.17 (dd, J = 5.8, 3.1 Hz, 1H), 6.10 (m, 1H), 5.93 (dd, J = 5.8, 2.9 Hz, 1H), 4.15 (m, 4H), 4.06 (m, 4H), 3.87 (dd, J = 10.8, 6.6 Hz, 1H), 3.67 (t, J = 10.0 Hz, 1H), 3.00 (m, 2H), 2.83 (d, J = 9.8 Hz, 2H), 2.71 – 2.61 (m, 3H), 2.37 (m, 1H), 1.84 (m, 1H), 1.49 – 1.43 (m, 1H), 1.39 – 1.24 (m, 2H), 0.55 (m, 1H). ^{13}C NMR (126 MHz, Chloroform-d) δ 141.59, 137.58, 136.90, 136.26, 136.11, 134.14, 132.20, 128.99, 128.97, 126.41, 125.96, 100.74, 100.70, 68.65, 67.98, 65.31, 65.29, 49.38, 44.95, 43.85, 43.65, 42.21, 41.59, 37.92, 37.77, 35.78, 30.86, 30.84, 29.58, 28.99.

b) After acidolysis of Compound S11 and purification by flash column chromatography on silica gel using a mixture of EA and hexane (*v*/*v*, 1/3) as the eluent, M3 (0.36 g) was obtained with a yield of 52.7%. ^{1}H NMR (500 MHz, Chloroform-d) δ 10.58 (s, 1H), 10.49 (s, 1H), 7.93 (d, J = 7.8 Hz, 1H), 7.85 (s, 1H), 7.65 (d, J = 7.8 Hz, 1H), 6.17 (dd, J = 5.8, 3.1 Hz, 1H), 6.11 – 6.05 (m, 1H), 5.90 (dd, J = 5.7, 2.9 Hz, 1H), 4.16 (dd, J = 10.9, 6.5 Hz, 1H), 3.98 (dd, J = 10.9, 9.1 Hz, 1H), 3.87 (dd, J = 10.7, 6.7 Hz, 1H), 3.68 (dd, J = 10.8, 9.3 Hz, 1H), 3.13 (m, 2H), 2.86 – 2.78 (m, 2H), 2.74 (m, 2H), 2.63 (m, 1H), 2.39 – 2.31 (m, 1H), 1.83 (m, 1H), 1.69 (m, 1H), 1.50 – 1.41 (m, 1H), 1.38 – 1.20 (m, 3H), 1.13 (m, 1H), 0.54 (m, 1H). ^{13}C NMR (126 MHz, Chloroform-d) δ 192.32, 191.97, 137.76, 137.02, 136.64, 136.08, 134.70, 133.67, 131.98, 131.87, 130.79, 68.94, 68.28, 49.38, 44.93, 43.83, 43.62, 42.19, 41.57, 37.94, 37.78, 34.91, 34.89, 30.79, 30.78, 29.56, 28.97.

Synthesis of M4:

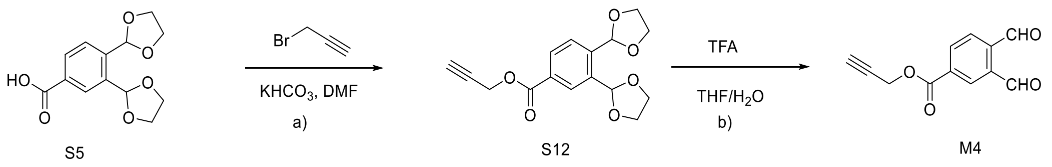

a) Compound S5 (1.0 g, 3.75 mmol, 1.0 eq.) was dissolved in 30 mL of dimethylformamide (DMF). K_2CO_3 (0.75 g, 7.5 mmol, 2.0 eq.) and 3-bromopropyne (1.12 g, 7.5 mmol, 2.0 eq.) were added to the solution under argon protection. The mixture was stirred at room temperature for 18 hours. After completion of the reaction, 50 mL of water was added to the mixture, and the resulting solution was extracted three times with EA (100 mL each). The combined organic layers were washed with brine, dried with Na_2SO_4, and filtered. The organic solution was concentrated under vacuum, yielding a residue. The residue was purified by flash column chromatography on silica gel using a mixture of EA and hexane (*v*/*v*, 1/3) as the eluent. Compound S12 was obtained as a solid (0.98 g) with a yield of 86%. ^{1}H NMR (500 MHz, Chloroform-d) δ 8.33 (s, 1H), 8.09 (d, J = 8.1 Hz, 1H), 7.74 (d, J = 8.1 Hz, 1H), 6.27 (s, 1H), 6.27 (s, 1H), 4.94 (s, 2H), 4.19 – 4.05 (m, 8H), 2.54 (s, 1H). ^{13}C NMR (126 MHz, Chloroform-d) δ 165.32, 141.35, 136.83, 130.34, 129.96, 127.75, 126.35, 100.27, 100.24, 75.10, 65.40, 52.54.

b) M4 was prepared following the same acidolysis procedure for M1. After acidolysis of compound S12 and purified by flash column chromatography on silica gel (EA/hexane=1/3, *v*/*v*) to give M4 (0.61 g, 88.5%). ^{1}H NMR (400 MHz, Chloroform-d) δ 10.65 (s, 1H), 10.52 (s, 1H), 8.66 (s, 1H), 8.46 (d, J = 8.0 Hz, 1H), 8.09 (d, J = 7.9 Hz, 1H), 5.02 (s, 2H), 2.60 (s, 1H). ^{13}C NMR (101 MHz, Chloroform-d) δ 191.58, 191.47, 139.43, 136.47, 134.69, 134.05, 132.95, 130.81, 60.43, 53.39.

Synthesis of M5:

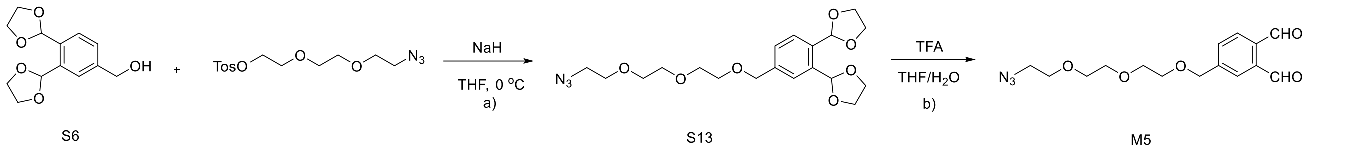

a) Compound S6 (0.32 g, 1.25 mmol) and 5 mL of dry tetrahydrofuran (THF) were added to an oven-dried 100 mL flask, and the system was placed in an ice bath. Sodium hydride (NaH) (0.10 g, 2.5 mmol) was added to the flask under stirring. TosO-PEG$_3$-N$_3$ (0.45 g, 1.38 mmol) was dissolved in 4 mL of dry THF and added to the flask. The reaction was stirred under argon protection for 12 hours. After completion of the reaction, the mixture was filtered through celite and concentrated under vacuum. The residue was purified by flash column chromatography on silica gel using a mixture of EA and hexane (*v*/v, 1/1) as the eluent. Compound S13 was obtained as a colorless oil (0.31 g) with a yield of 60%. ^{1}H NMR (500 MHz, Chloroform-d) δ 7.67 (s, 1H), 7.66 –7.59 (d, 7.9 Hz, 1H), 7.41-7.40 (d, J = 7.9 Hz, 1H), 6.23 (s, 1H), 6.23 (s, 1H), 4.60 (s, 2H), 4.19 – 4.04 (m, 8H), 3.75 – 3.61 (m, 10H), 3.40 (m, 2H). ^{13}C NMR (126 MHz, Chloroform-d) δ 139.29, 136.12, 135.45, 128.23, 126.35, 125.37, 100.70, 72.82, 70.72, 70.05, 69.44, 65.31, 65.30, 50.71.

b) M5 was prepared following the same acidolysis procedure for M1. After acidolysis of compound S13 and purification by flash column chromatography on silica gel using a mixture of EA/hexane (1/1, *v*/*v*) as the eluent. M5 was obtained as a colorless oil (0.23 g) with a yield of 95%. ^{1}H NMR (500 MHz, Chloroform-d) δ 10.56 (s, 1H), 10.52 (s, 1H), 7.98 (s, 1H), 7.97 (d, J = 7.9 Hz, 1H), 7.77 (d, J = 7.9 Hz, 1H), 4.73 (s, 2H), 3.80 – 3.65 (m, 10H), 3.40 (broad, 2H). ^{13}C NMR (126 MHz, Chloroform-d) δ 192.42, 192.40, 192.07, 145.35, 136.52, 135.50, 132.00, 131.49, 129.55, 71.93, 70.75, 70.72, 70.35, 70.09, 50.69.

Synthesis of M6:

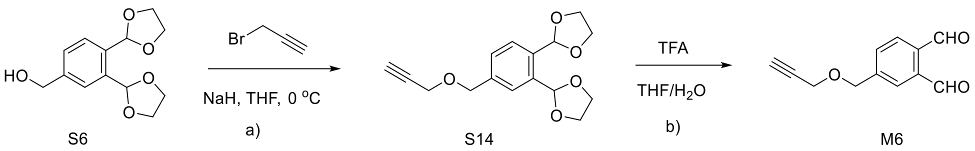

a) Compound S6 (1.65 g, 6.54 mmol) was dissolved in 50 mL of anhydrous THF in a reaction flask and placed in an ice bath for 3 minutes. Sodium hydride (NaH) (0.65 g, 16.35 mmol) was added to the reaction flask and stirred for 10 minutes. 3-bromopropyne (1.94 g, 13.08 mmol, 2.0 eq.) was added to the reaction flask using a syringe under argon protection. The reaction mixture was stirred at 0 °C for 1 hour and then allowed to warm up to room temperature overnight. The reaction mixture was filtered through celite, and the filtrate was concentrated under vacuum. The residue was purified by flash column chromatography on silica gel using a mixture of EA and hexane (*v*/*v*, 1/3) as the eluent. Compound S14 was obtained as an oil (1.55 g) with a yield of 82%. ^{1}H NMR (500 MHz, Chloroform-d) δ 7.66 (s, 1H), 7.65 (d, J = 7.4 Hz, 2H), 7.41 (d, J = 8.0 Hz, 1H), 6.24 (s, 1H), 6.24 (s, 1H), 4.65 (s, 2H), 4.24 – 3.98 (m, 10H), 2.49 (s, 1H). ^{13}C NMR (126 MHz, Chloroform-d) δ 138.28, 136.28, 135.77, 128.51, 126.39, 125.65, 100.67, 100.64, 79.60, 74.72, 71.07, 65.33, 65.30, 57.09.

b) M6 was prepared following the same acidolysis procedure for M1. After acidolysis of compound S14 and purification by flash column chromatography on silica gel using a mixture of EA/hexane (1/3, *v*/*v*) as the eluent. M6 was obtained as a yellow oil (1.05 g) with a yield of 97%. ^{1}H NMR (500 MHz, Chloroform-d) δ 10.57 (s, 1H), 10.54 (s, 1H), 8.00 (s, 1H), 7.99 (d, 7.8 Hz, 1H), 7.79 (d, J = 7.8 Hz, 1H), 4.77 (s, 2H), 4.30 (s, 2H), 2.54 (s, 1H). ^{13}C NMR (126 MHz, Chloroform-d) δ 192.25, 191.98, 144.33, 136.55, 135.70, 132.26, 131.50, 129.83, 75.46, 70.22, 58.05.

Synthesis of M7 and M8:

M7 was prepared through the same method for M8.

As an example, M8 was synthesized as follows:

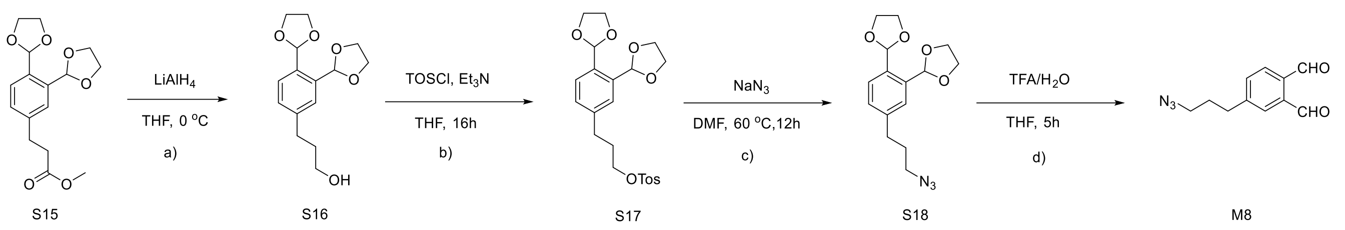

a) Compound S15 was prepared according to synthetic route in literature reported.[43,44] Compound S16 was synthesized using the same procedure as Compound S6. In a 100 mL oven-dried round bottom flask, LiAlH4 (0.63 g, 16.21 mmol, 2.5 eq.) was placed and the flask was then placed in an ice bath at 0 °C under argon protection. Slowly, 10 mL of anhydrous THF was added to the flask and stirred for 3 minutes. A solution of Compound 15 (2.0 g, 6.49 mmol) in 40 mL anhydrous THF was added dropwise to the flask. The resulting mixture was stirred at 0 °C for 4 hours. After the reaction was completed, 0.63 mL of H_2O was added dropwise to quench the reaction slowly and carefully. The mixture was allowed to stir for an additional 10 minutes. Then, a solution of 0.63 mL of 15 % NaOH (aq.) was added to the reaction mixture. After 10 minutes, 1.89 mL (3 × 0.63 mL) of H_2O was added, followed by dilution with 30 mL of THF. The mixture was stirred at room temperature for 8 hours. The resulting mixture was dried using anhydrous Na_2SO_4 and filtered through celite. The filtrate was then evaporated under vacuum to afford Compound S16 as a yellow oil (1.80 g) with a yield of 99%. ^{1}H NMR (400 MHz, $CDCl_3$) δ= 7.57 (d, J = 7.9 Hz, 1H), 7.46 (s, 1H), 7.21 (d, J = 7.9 Hz, 1H), 6.18 (s, 1H), 6.18 (s, 1H), 4.14-4.11 (m, 4H), 4.08 – 3.98 (m, 4H), 3.63 (m, 2H), 2.70 (m, 2H), 1.90 – 1.82 (m, 2H). ^{13}C NMR (400 MHz, $CDCl_3$) δ= 142.817, 135.805, 133.568, 129.038, 126.227, 125.987, 100.693, 65.195, 62.031, 33.942, 31.777, 29.599.

b) Compound S16 (2.0 g, 7.13 mmol) and Et_3N (1.44 g, 14.27 mmol) were combined in a 50 mL anhydrous DCM solution. A 10 mL solution of *p*-toluenesulfonyl chloride (TOSCl, 2.72 g, 14.27 mmol, 2.0 eq.) was then added dropwise using a dropping funnel. The resulting mixture was stirred at room temperature for 18 hours. After the reaction period, the mixture was concentrated under vacuum. The crude product was then purified by flash column chromatography on silica gel using a mixture of EA and hexane (*v*/*v*, 1/2) as the eluent. Compound S17 was obtained as a white oil (2.20 g) with a yield of 71%. ^{1}H NMR (500 MHz, Chloroform-d) δ 7.83 – 7.77 (d, 8.0 Hz, 2H), 7.52 (d, J = 7.9 Hz, 1H), 7.42 (s, 1H), 7.35 (d, J = 8.0 Hz, 2H), 7.09 (d, J = 8.0 Hz, 1H), 6.18 (s, 1H), 6.18 (s, 1H), 4.18 – 4.00 (m, 10H), 2.68

(m, 2H), 2.46 (s, 3H), 1.96 (m, 2H). ^{13}C NMR (126 MHz, Chloroform-d) δ 144.80, 141.42, 136.17, 134.05, 133.05, 129.91, 129.08, 127.90, 126.40, 126.04, 100.68, 100.64, 69.65, 65.32, 65.30, 31.34, 30.31, 21.64.

c) Compound S17 (2.2 g, 5.06 mmol) was dissolved in 50 mL of DMF. NaN_3 (0.668 g, 10.13 mmol) was then added to the solution. The mixture was placed in an oil bath and heated at 60°C for 12 hours. After the reaction was completed, 50 mL of water was added to the reaction mixture. The resulting mixture was then extracted with EA three times. The combined organic layers were washed with water three times. The organic layer was concentrated under vacuum to yield Compound S18 (1.5 g) with a yield of 96.6%. ^{1}H NMR (400 MHz, Chloroform-d) δ 7.59 (d, J = 8.0 Hz, 1H), 7.49 (s, 1H), 7.23 (d, J = 8.0 Hz, 1H), 6.22 (s, 1H), 6.22 (s, 1H), 4.22 – 4.01 (m, 8H), 3.30 (m, 2H), 2.78 – 2.70 (m, 2H), 1.93 (m, 2H). ^{13}C NMR (101 MHz, Chloroform-d) δ 141.87, 136.10, 134.01, 129.15, 126.44, 126.06, 100.74, 100.69, 65.34, 65.32, 50.63, 32.60, 30.30.

d) M8 was prepared according to the same acidolysis procedure for M1. After acidolysis of compound S18 and purification by flash column chromatography on silica gel using a mixture of EA/hexane (*v*/*v*, 1/3) as the eluent. M8 was obtained as a light-yellow oil (1.0 g) with a yield of 94.3%. ^{1}H NMR (400 MHz, Chloroform-d) δ 10.60 (s, 1H), 10.49 (s, 1H), 7.94 (d, J = 7.8 Hz, 1H), 7.82 (s, 1H), 7.62 (d, J = 7.8 Hz, 1H), 3.36 (m, 2H), 2.89 (m, 2H), 2.07 – 1.90 (m, 2H). ^{13}C NMR (101 MHz, Chloroform-d) δ 192.35, 191.97, 147.89, 136.70, 134.64, 133.68, 132.02, 130.70, 50.46, 32.78, 29.91.

Monomer M7:

Compound S6 was a starting material to prepare M7.

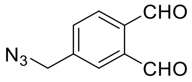

^{1}H NMR (500 MHz, Chloroform-d) δ 10.60 (s, 1H), 10.54 (s, 1H), 8.03 (d, J = 7.8 Hz, 1H), 7.95 (s, 1H), 7.76 (d, J = 7.9 Hz, 1H), 4.58 (s, 2H). ^{13}C NMR (126 MHz, Chloroform-d) δ 191.81, 191.72, 142.02, 136.83, 135.97, 132.67, 132.01, 130.01, 53.78.

Synthesis of M9:

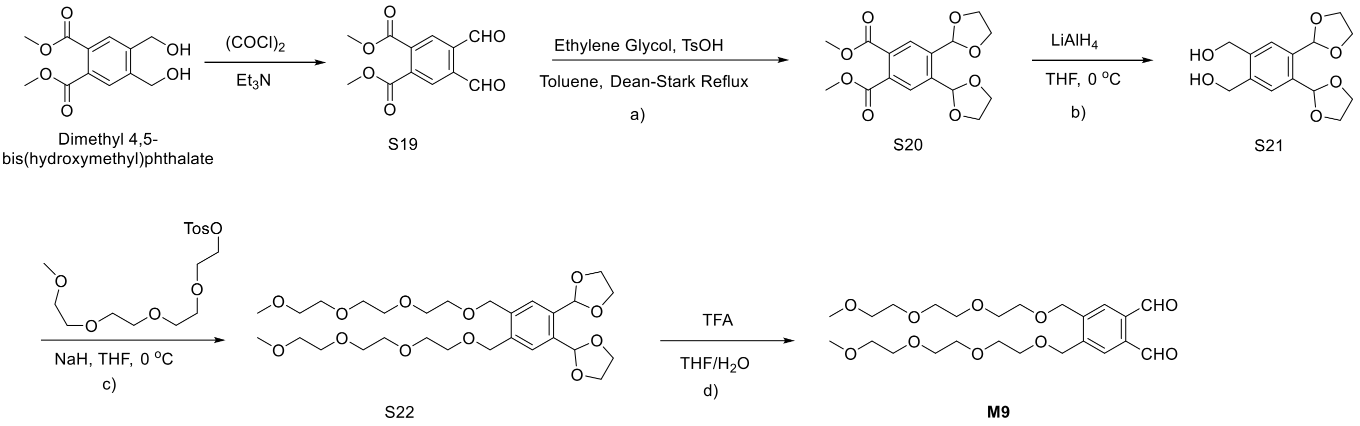

Compound S19 was prepared according to the literature reported.[5] Compound S20 (4.52 g, yield 74%) and compound S21 (3.48 g, yield 96%) were prepared through step a and b, respectively, from dimethyl 4,5-bis(hydroxymethyl)phthalate (6.60g, 26 mmol), which followed the same synthetic procedure for compound S4 and S6, respectively. Additionally, prepare compound S22 (0.46 g, yield 69 %) and M9 (0.37 g, yield 96%) were prepared via step c and d, which followed the same synthetic procedure for compound S13 and M1, respectively.

Compound S20: ^{1}H NMR (400 MHz, Chloroform-d) δ 7.99 (s, 1H), 7.99 (s, 1H), 6.26 (s, 1H), 6.26 (s, 1H), 4.22 – 4.01 (m, 8H), 3.90 (s, 3H), 3.90 (s, 3H). ^{13}C NMR (101 MHz, Chloroform-d) δ 167.63, 139.58, 132.26, 126.90, 99.87, 65.41, 52.68.

Compound S21: ^{1}H NMR (500 MHz, DMSO-d6) δ 7.59 (s, 1H), 7.59 (s, 1H), 6.03 (s, 1H), 6.03 (s, 1H), 5.16 (s, 1H), 5.16 (s, 1H), 4.53 (s, 2H), 4.53 (s, 2H), 4.12 – 3.89 (m, 8H). ^{13}C NMR (126 MHz, DMSO-d6) δ 140.42, 134.51, 124.95, 100.29, 65.21, 60.43.

Compound S22: ^{1}H NMR (500 MHz, Chloroform-d) δ 7.65 (s, 1H), 7.65 (s, 1H), 6.21 (s, 1H), 6.21 (s, 1H), 4.64 (s, 2H), 4.64 (s, 2H), 4.15 – 4.01 (m, 8H), 3.69 – 3.63 (m, 24H), 3.60 (m, 4H), 3.57 – 3.53 (m, 4H), 3.38 (s, 3H), 3.38 (s, 3H). ^{13}C NMR (126 MHz, Chloroform-d) δ 137.34, 135.45, 126.70, 100.70, 71.93, 70.62, 70.61, 70.58, 70.51, 69.54, 65.26, 59.03.

M9: ^{1}H NMR (500 MHz, Chloroform-d) δ 10.55 (s, 1H), 10.54 (s, 1H), 8.08 (s, 1H), 8.08 (s, 1H), 4.72 (s, 2H), 4.72 (s, 2H), 3.72-3.55 (m, 32H), 3.38 (s, 3H), 3.38 (s, 3H). ^{13}C NMR (126 MHz, Chloroform-d) δ 192.35, 142.60, 135.41, 130.64, 71.93, 70.70, 70.62, 70.59, 70.52, 70.32, 69.79, 59.03.

2.3 Synthesis of payloads (P1 to P4) and compound 27, 28, 30 and 32

Synthesis of P1:

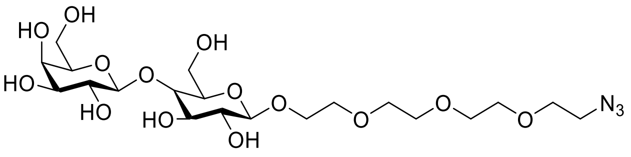

P1

P1 was prepared according to the synthetic route in literature reported.[6]

Synthesis of P2:

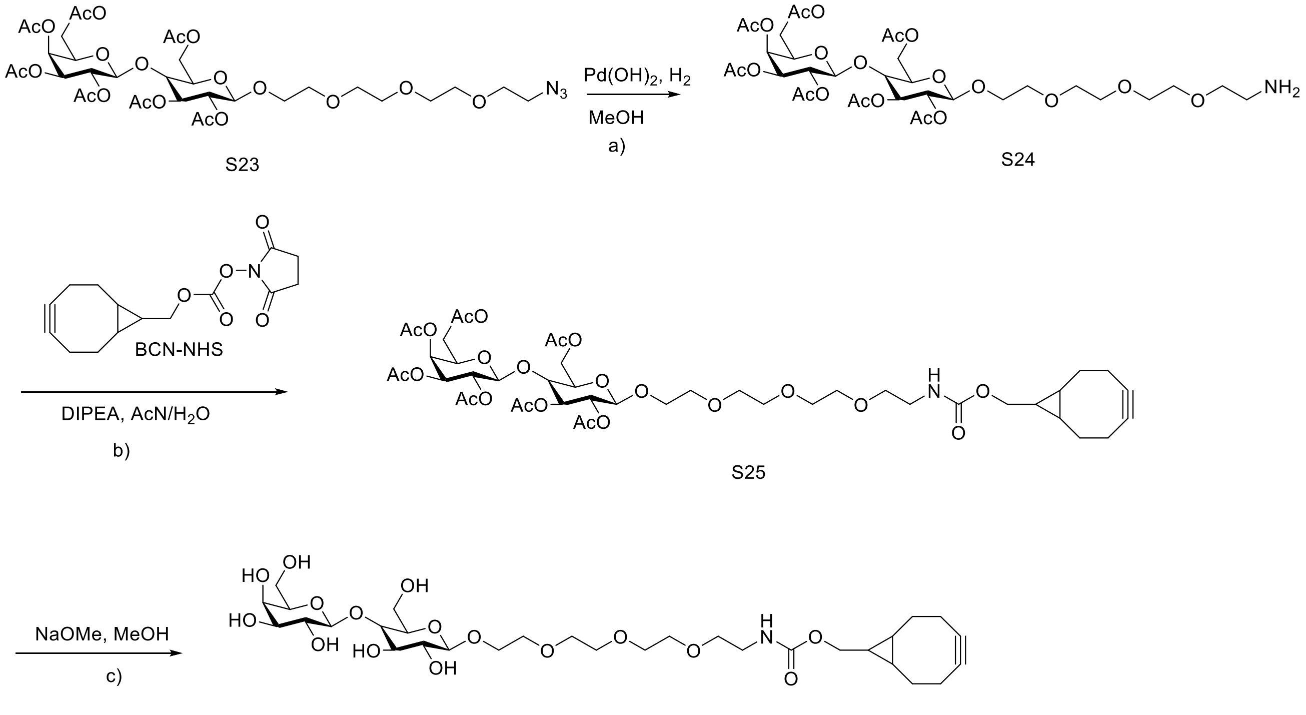

P2

To synthesize P2, the following steps were carried out:

a) In a 50 mL flask, compound S23[6] (0.4 g, 0.48 mmol), $Pd(OH)_2$ (68 mg, 0.48 mmol), and 30 mL of methanol (MeOH) were combined. The flask was then degassed and refilled with hydrogen gas (H_2) three

times. The reaction mixture was stirred under a H_2 atmosphere at room temperature for 2 hours, and the progress of the reaction was monitored by TLC. After the reaction period, the mixture was filtered using celite. The filtrate was then evaporated under vacuum to yield Compound 24 (0.36 g) with a yield of 93%, which was directly used for the next step without undergoing further purification. The UPLC mass analysis of compound S24 is presented in Figure S20.

b) Compound S24 (0.18 g, 0.22 mmol) was dissolved in 6 mL of acetonitrile (ACN)/water (2/1, *v*/*v*), then 0.1 mL *N*,*N*-Diisopropylethylamine (DIPEA) was added. BCN-NHS[7] (64 mg, 0.22 mmol) was dissolved in 1 mL ACN then added to the mixture solution. The mixture was stirred at room temperature for 1 hour. After the reaction was completed, the mixture was concentrated under vacuum, then purified by flash column chromatography on silica gel using EA as the eluent. Compound S25 was obtained as a white solid (0.19 g) with a yield of 88%. The UPLC mass analysis of the purified compound S25 is presented in Figure S21.

Compound 25: ^{1}H NMR (500 MHz, Chloroform-d) δ 5.35 (dd, J = 3.5, 1.1 Hz, 1H), 5.19 (t, J = 9.3 Hz, 2H), 5.11 (dd, J = 10.4, 7.9 Hz, 1H), 4.96 (dd, J = 10.4, 3.5 Hz, 1H), 4.89 (dd, J = 9.6, 7.9 Hz, 1H), 4.57 (d, J = 7.9 Hz, 1H), 4.49 (d, J = 8.0 Hz, 2H), 4.22 – 4.05 (m, 5H), 3.97 (d, J = 6.9 Hz, 1H), 3.95 – 3.85 (m, 2H), 3.80 (t, J = 9.5 Hz, 1H), 3.72 (m, 1H), 3.68 – 3.58 (m, 11H), 3.56 (t, J = 5.2 Hz, 2H), 3.37 (d, J = 5.4 Hz, 2H), 2.40 (dd, J = 13.3, 2.9 Hz, 1H), 2.34 – 2.22 (m, 3H), 2.14 (d, J = 15.4 Hz, 7H), 2.09 – 2.01 (m, 13H), 1.97 (s, 3H), 1.67 – 1.52 (m, 1H), 1.37 (m, 2H), 1.00 – 0.90 (m, 1H), 0.79 – 0.64 (m, 2H). ^{13}C NMR (126 MHz, Chloroform-d) δ 170.37, 170.15, 169.79, 169.66, 169.09, 101.09, 100.62, 98.80, 76.27, 72.83, 72.62, 71.66, 70.98, 70.67, 70.58, 70.52, 70.28, 70.12, 69.11, 69.06, 66.60, 61.99, 60.77, 40.77, 33.29, 29.05, 23.77, 22.84, 21.42, 21.39, 20.87, 20.81, 20.70, 20.64, 20.51, 20.10, 17.79.

c) To a stirring solution of compound S25 (0.19 g, 0.19 mmol) in dry MeOH (10 mL), a 0.1 mL solution of sodium methoxide (NaOMe, 1M in MeOH) was added. The reaction mixture was stirred at room temperature for 2 hours. After the reaction period, the mixture solution was neutralized by adding ion-exchange resin (Dowex 50W×8, 50-100 H) until the pH reached 7. The solution was then filtered, and

the filtrate was dried with Na_2SO_4. The solution was filtered again, and the resulting solution was concentrated under vacuum to afford P2 as a colorless oil (0.12 g) with a yield of 92%. The UPLC mass analysis of P2 is presented in Figure S22.

P2: ^{1}H NMR (500 MHz, Deuterium Oxide) δ 4.43 (d, J = 7.9 Hz, 1H), 4.37 (d, J = 7.8 Hz, 1H), 4.12 (d, J = 8.3 Hz, 1H), 4.03 – 3.83 (m, 4H), 3.81 – 3.43 (m, 24H), 3.33 – 3.20 (m, 3H), 2.32 (d, J = 13.4 Hz, 1H), 1.51 (d, J = 12.0 Hz, 1H), 1.41 – 1.12 (m, 3H), 0.98 – 0.62 (m, 3H). ^{13}C NMR (126 MHz, Deuterium Oxide) δ 158.84, 102.96, 102.11, 100.28, 78.41, 75.35, 74.77, 74.30, 72.80, 72.52, 70.94, 69.68, 69.61, 69.42, 68.73, 68.53, 60.99, 60.08, 40.01, 32.74, 28.57, 23.25, 22.47, 20.74, 20.68, 19.74.

Synthesis of P3:

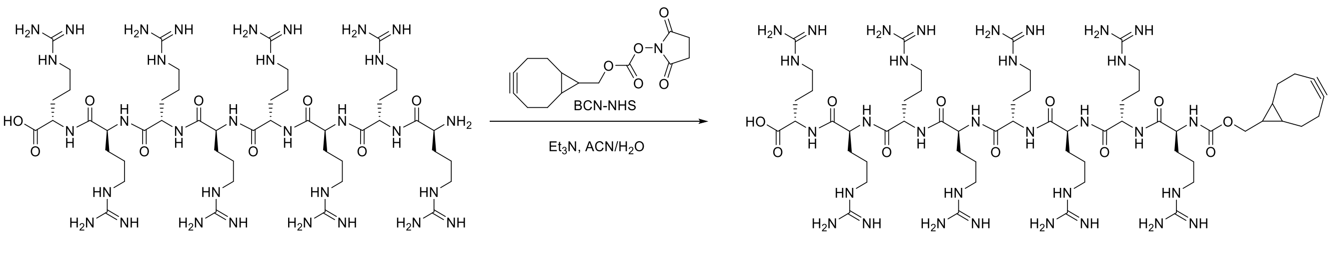

Oligoarginine octapeptide (Arg_8)

P3

To synthesize P3, the following steps were carried out:

Solid-phase peptide synthesis of oligoarginine octapeptide $(Arg)_8$: The 2-chlorotrityl chloride (CTC) resin (GL Biochem, loading: 1.0 mmol/g) (1.0 g, 1.0 mmol) was pre-swelled in 30 mL of anhydrous DCM for 20 minutes in a 50 mL polypropylene syringe with a polyethylene filter at the bottom. The resin was washed with DCM (3 × 20 mL) and DMF (3 × 20 mL). Fmoc-Arg(Pbf)-OH (2.60 g, 4.0 mmol) and DIPEA (1.03 g, 8.0 mmol) as well as 25 mL DCM were added to resin and shaken for 2 hours. The resin was washed with DCM and DMF. A 20 mL solution of 25% 4-methyl piperidine in DMF was added to the resin for 20 minutes to remove the Fmoc protecting group, followed by washing with DCM and DMF. After that, Fmoc-Arg(Pbf)-OH (2.60 g, 4.0 mmol) and HATU (1.52 g, 4.0 mmol) were dissolved in 25 mL of DMF for 2 minutes, and then DIPEA (1.03 g, 8.0 mmol) was added to the solution. The mixture solution was added to the resin and shaken for 2 hours to load the second amino acid. The resin was

washed with DCM and DMF. A solution of 25% 4-methyl piperidine in DMF (20 mL) was added to the resin for 20 minutes to remove the Fmoc protecting group, followed by washing with DCM and DMF. The above loading of second amino acid steps were repeated for the necessary number of arginine units to complete the synthesis of $(Arg)_8$. After the final coupling and deprotection steps, TFA/Triisopropylsilane (Tips)/H_2O (95/2.5/2.5, *v*/*v*/*v*) (10 mL) were added for global deprotection. The crude product was precipitated in Et_2O and purified by HPLC using ACN/H_2O as the eluent (ACN gradient: 2-30%). The UPLC mass analysis of the purified $(Arg)_8$ is presented in Figure S23.

Arg_8 (0.20 g, 0.16 mmol) was dissolved in 4 mL of ACN/H_2O (1/1, *v*/*v*). 0.1 mL of Et_3N was added to the solution. BCN-NHS (43.80 mg, 0.15 mmol) was dissolved in 1 mL of ACN and added to the mixture solution. The mixture was stirred at room temperature for 2 hours. After the reaction was completed, the mixture solution was directly dried under freeze-drying to obtain P3 as a white solid powder. P4 was then ready for the next step. The UPLC mass analysis of P3 is presented in Figure S24.

Synthesis of P4:

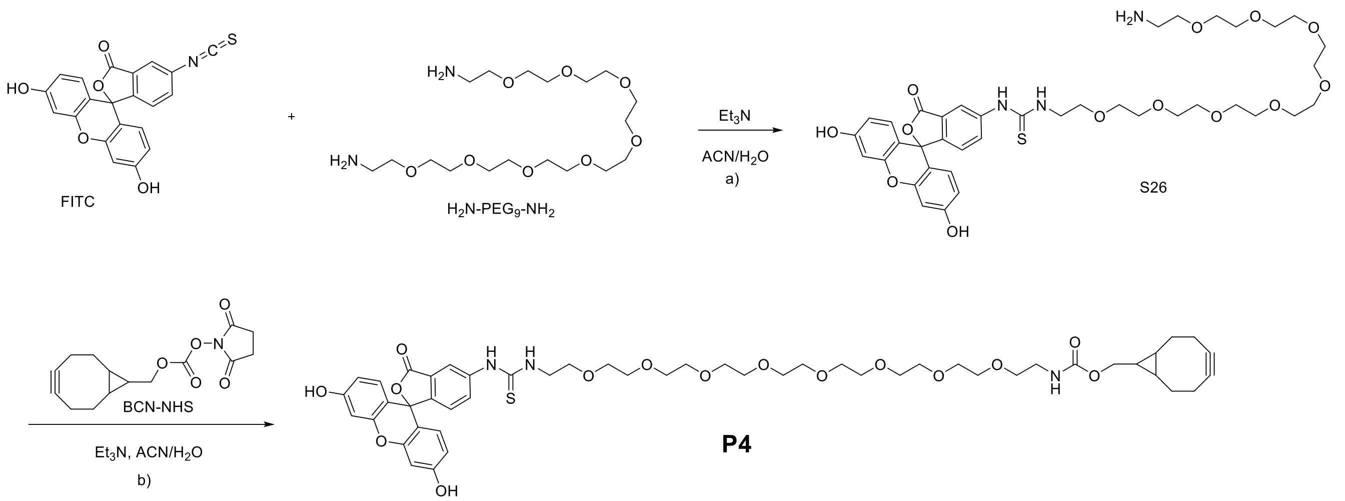

To synthesize P4, the following steps were carried out:

a) H_2N-PEG_9-NH_2 (0.61 g, 1.44 mmol, 3.0 eq.) was dissolved in a mixture of 10 mL ACN/H_2O (1/1, *v*/*v*). Then, Et_3N (0.2 mL) was added to the solution. FITC (0.18 g, 0.48 mmol, 1.0 eq.) dissolved in 5 mL of ACN was added dropwise to the mixture over a period of 0.5 hours. The resulting mixture was stirred at

room temperature for 1 hour. Afterward, the mixture was subjected to HPLC purification using ACN/H_2O as the eluent, with an ACN gradient of 5-95%. The purified product was then freeze-dried to obtain compound 26 (0.30 g) with a yield of 78%. The UPLC mass analysis of compound S26 is presented in Figure S25.

b) Compound S26 (91.2 mg, 0.11 mmol) was dissolved in 6 mL of ACN/H_2O (2/1, *v*/*v*). Et_3N (0.1 mL) was added to the solution, followed by the addition of BCN-NHS (30.5 mg, 0.11 mmol) in 2 mL of ACN. The mixture was stirred at room temperature for 4 hours. The resulting mixture solution was then freeze-dried to obtain P4, which was directly used for the next step without further purification. The UPLC mass analysis of P4 is presented in Figure S26.

Synthesis of 1

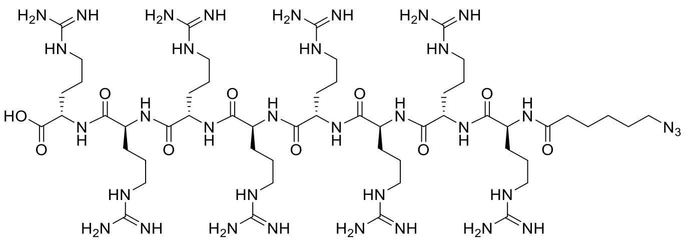

1 ((Arg)$_8$-N_3)

(Arg)$_8$-N_3 **1** was synthesized via SPPS using CTC resin. The synthesis of (Arg)$_8$ followed the same procedure as described for the preparation of P4. N_3-C_5H_{10}-COOH (4.0 eq.) was used to couple with (Arg)$_8$ on the resin. After global deprotection, the crude product was precipitated in Et_2O and purified by HPLC using ACN/H_2O as the eluent (ACN gradient: 2-30%) to afford 1. The UPLC mass analysis of the purified **1** is presented in Figure S27.

Synthesis of 2:

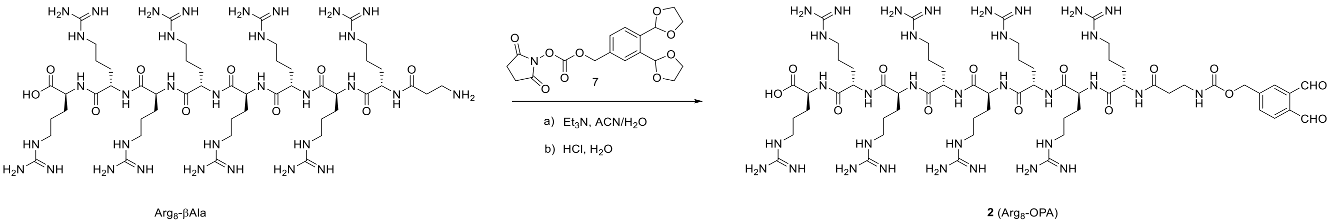

Arg$_8$-βAla

2 (Arg$_8$-OPA)

(Arg)$_8$-βAla was prepared via SPPS by using the CTC resin, which followed the same procedure as described for the preparation of P4. (Arg)$_8$-βAla (0.10 g, 0.07 mmol) was dissolved in 3 mL of ACN/H_2O (1/1, *v*/*v*), then 0.1 mL of Et_3N was added to the solution. Compound S7 (27.5 mg, 0.07 mmol) was dissolved in 1 mL of ACN and added to the mixture solution. The mixture was stirred at room temperature for 2 hours. The mixture solution was then washed with EA three times. 1M HCl (aqueous) was added to the aqueous solution. After the reaction was completed, the mixture was subjected to HPLC purification using ACN/H_2O as the eluent, with an ACN gradient of 2-30%, to afford (Arg)$_8$-OPA **2** (74 mg) with a yield of 70%. The UPLC mass analysis of (Arg)$_8$-βAla and purified 2 are presented in Figure S28 and S29, respectively.

Synthesis of 3:

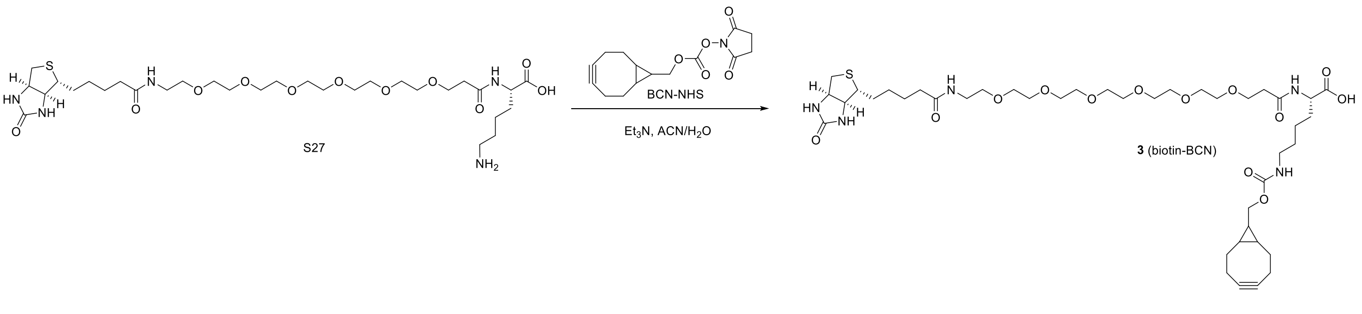

Compound S27 was prepared vis SPPS. The CTC resin (500 mg, 0.5 mmol/g) was pre-swelled in 10 mL of anhydrous DCM for 20 minutes, followed by washing with DCM and DMF. Fmoc-Lys(Boc)-OH (0.5 g, 1.06 mmol) and DIPEA (0.39 mL, 2.12 mmol) were dissolved in 15 mL of DCM and added to the resin, which was then shaken for 2 hours. The resin was washed with DCM and DMF, and then 15 mL of a solution containing 25% 4-methyl piperidine in DMF was added to remove the Fmoc protecting group. The resin was washed again with DCM and DMF. Fmoc-PEG$_6$-COOH (0.61 g, 1.06 mmol) and HATU (0.40 g, 1.05 mmol) were mixed in 10 mL DMF, then DIPEA (0.39 mL, 2.12 mmol) was added. The mixture solution was added to resin and shaken for 2 hours, after repeating above washing and deprotection procedure. Repeat above procedure to preparing the mixture solution of biotin (0.26 g, 1.06 mmol), HATU (0.40 g, 1.05 mmol) and DIPEA (0.39 mL, 2.12 mmol) in 10 mL DMF, then added to resin. After the reaction was completed and followed by washing, FA/Tips/H_2O (95/2.5/2.5, *v*/*v*/*v*) (10

mL) was added to the resin for global deprotection. The resulted solution was poured into Et_2O to form white solid precipitation, which was washed with Et_2O three times. The solid was dried under vacuum to afford compound S27 (0.40 g) as a white solid powder, which was directly used in subsequent steps. The UPLC mass analysis of compound S27 is presented in Figure S30.

Compound S27 (100.0 mg, 0.14 mmol) was dissolved in 2 mL of ACN/H_2O (1/1, *v/v*). 20 μL of Et_3N was added to the solution. BCN-NHS (40.8 mg, 0.14 mmol) was dissolved in 1 mL of ACN and added to the mixture solution. The mixture was stirred at room temperature for 2 hours. After the reaction was completed, the mixture solution was directly dried under freeze-drying conditions to afford biotin-BCN **3** as a white solid powder, which was directly used for the next step. The UPLC mass analysis of 3 is presented in Figure S31.

Synthesis of OPA-FITC:

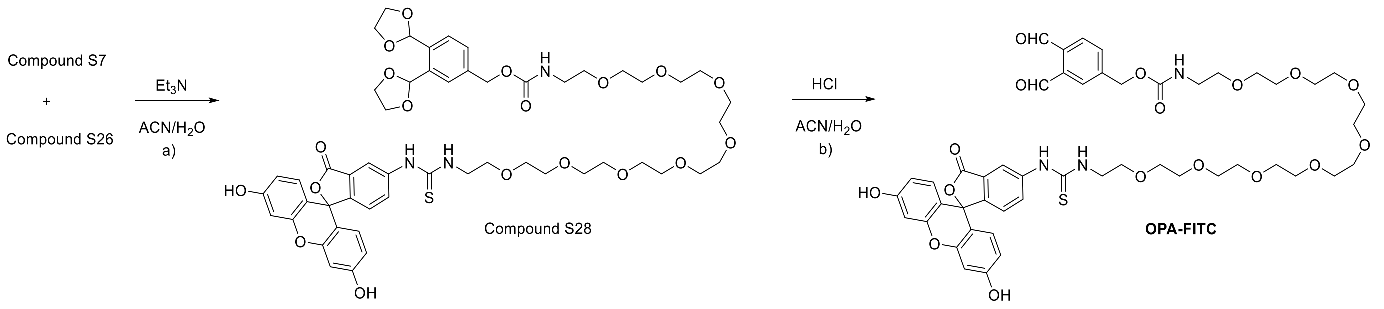

To synthesize OPA-FITC, the following steps were carried out:

a) Compound S26 (97.1 mg, 0.12 mmol) was dissolved in 10 mL of ACN/H_2O (1/1, *v/v*). 100 μL of Et_3N was added to the solution. Compound S7 (50.0 mg, 0.125 mmol) was dissolved in 1 mL of ACN and added to the mixture solution. The mixture was stirred at room temperature for 1 hours. After the reaction was completed, the mixture solution was directly dried under freeze-drying conditions to afford crude compound S28 as a yellow solid, which was directly used for the next step. The UPLC mass analysis of compound S28 is presented in Figure S32.

b) The acidolysis of compound S28 was followed by the same procedure for 2, then the mixture solution was subjected to HPLC purification using ACN/H_2O as the eluent, with an ACN gradient of 20-95%, to

afford OPA-FITC as a yellow solid (77.0 mg) with a yield of 65%. The UPLC mass analysis of OPA-FITC is presented in Figure S33.

3. Synthesis of cyclic polyphthalaldehydes (cPPAs)

Cyclic polyphthalaldehydes were synthesized via polymerization of *ortho*-phthalaldehyde (OPA) derivatives (M1-M9) according to literature.[7] As an example, cPoly(M8) was synthesized as follows. M8 (0.67 g, 3.09 mmol) and DCM (5 mL) were added into an overnight oven dried 25 mL Schlenk flask. The mixture was degassed via three freeze-pump-thaw cycles then cooled to -78 °C in a dry ice/acetone bath. BF_3Et_2O (20 µL, 0.2 mmol) was added, and the reaction was stirred under argon at -78 °C for 2 h. The reaction was quenched by adding pyridine (0.4 mL, 4.8 mmol, pre-dissolved in 1 mL DCM), and allowed to stir at -78 °C for another 2 h. After that, the reaction mixture was added dropwise into 100 mL methanol, a white precipitate immediately formed. The solid was redissolved in DCM and reprecipitated in MeOH for three times, then dried under vacuum to give pure product (0.286 g, yield: 41.7%). The polymerization results are presented in Table S1, and their GPC and ^{1}H-NMR analysis results are shown in Figure S1 to S5. Maldi-tof MS analysis of cPloy(M2) and cPoly(M7) are shown in Figure S2c and S5c respectively, while others are failed to obtain.

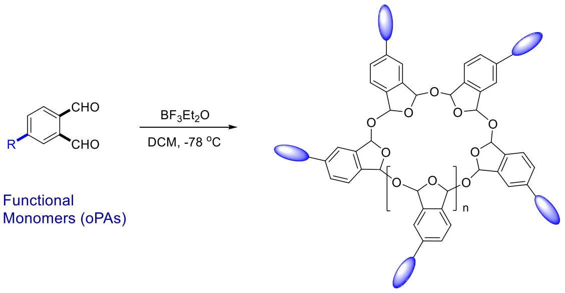

Table S1. The results of Cationic polymerization of oPAs[a].

oPAs	Polymer	M_n (kg·mol^{-1})	M_w (kg·mol^{-1})	PDI	Yield (%)[b]
**M1**	**cPoly(M1)**	59.96	106.19	1.77	60
**M2**	**cPoly(M2)**	15.23	34.24	2.25	51
**M3**	**cPoly(M3)**	1.48	1.83	1.23	33
**M4**	**cPoly(M4)**	2.28	2.86	1.25	43
**M5**	**cPoly(M5)**	---	---	---	---

**M6**	**cPoly(M6)**	28.15	54.58	1.94	55
**M7**	**cPoly(M7)**	18.32	41.41	2.26	46
**M8**	**cPoly(M8)**	27.88	45.13	1.61	42
**M9**	**cPoly(M10)**	---	---	---	---

[a] Catalyst: BF_3Et_2O (20 μL, 0.2 mmol), monomer: (0.67 g, 3.09 mmol), DCM: 5 mL, pyridine: (0.4 mL, 4.8 mmol). [b] Yield was calculated based on the weight of the isolated product. M_n and M_w were determined by GPC in DMF, polydispersity index (PDI=M_w/M_n), PS as the calibration standard.

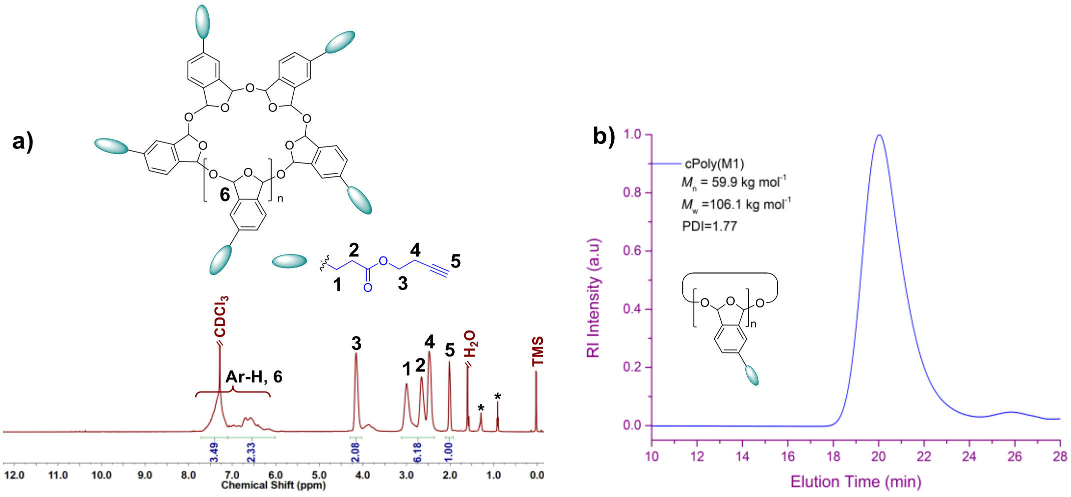

Figure S1: ^{1}H NMR spectrum (a, in $CDCl_3$) and GPC curve (b) of **cPoly(M1)**.

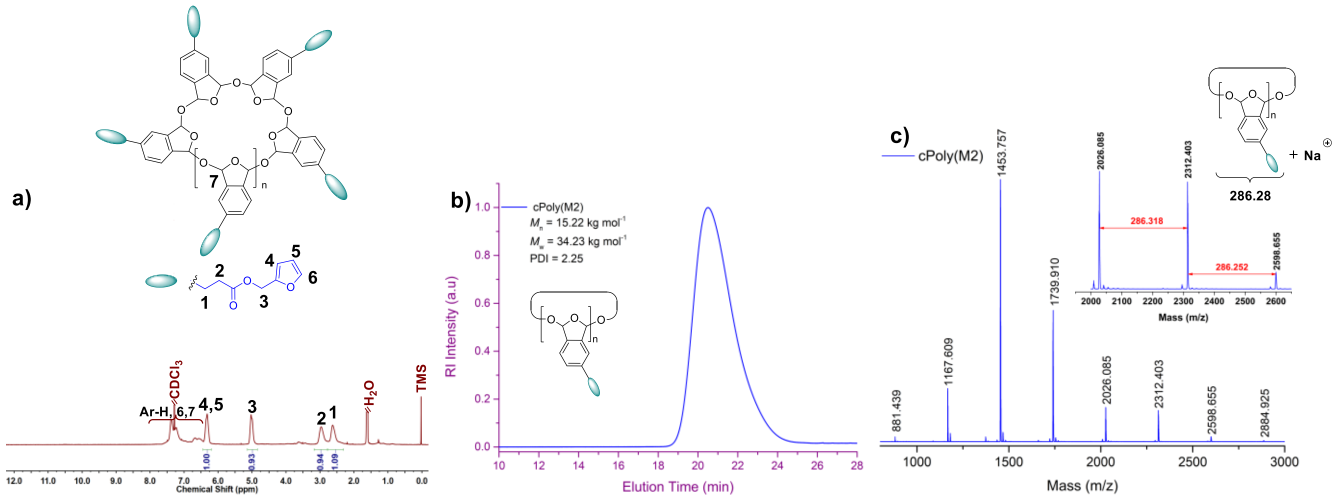

Figure S2: ^{1}H NMR spectrum (a, in $CDCl_3$), GPC curve (b) and Maldi-tof MS (c) of **cPoly(M2)**.

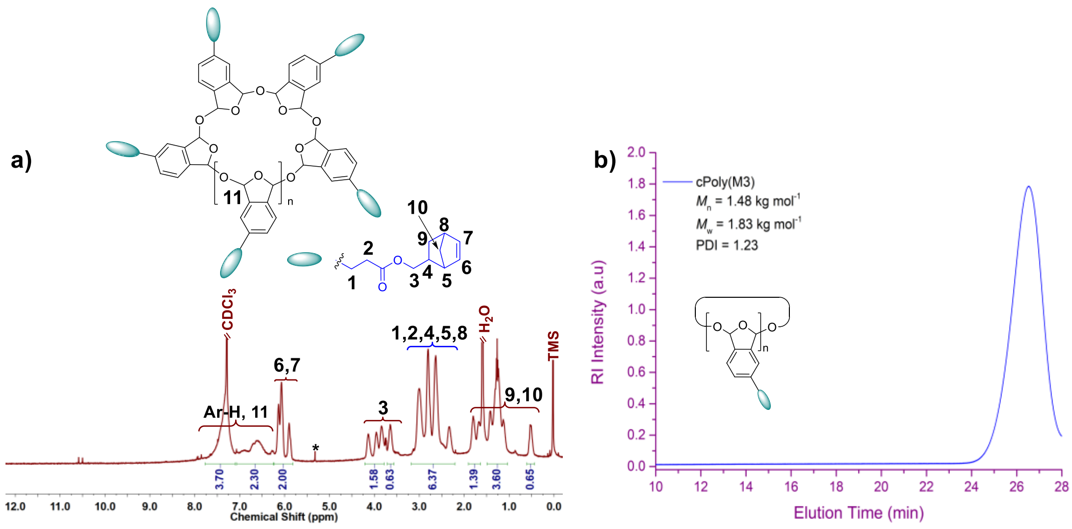

Figure S3: ^{1}H NMR spectrum (a, in $CDCl_3$) and GPC curve (b) of **cPoly(M3)**.

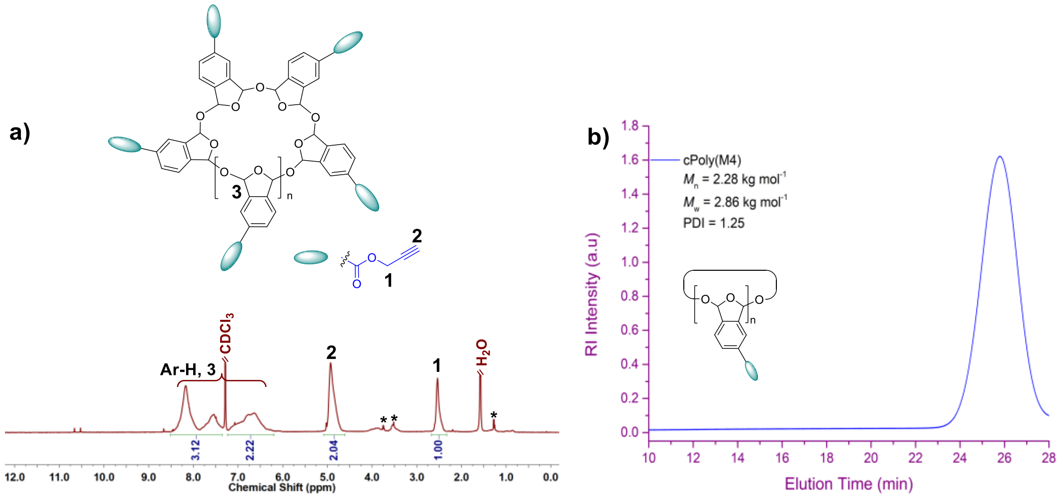

Figure S4: ^{1}H NMR spectrum (a, in $CDCl_3$) and GPC curve (b) of **cPoly(M4)**.

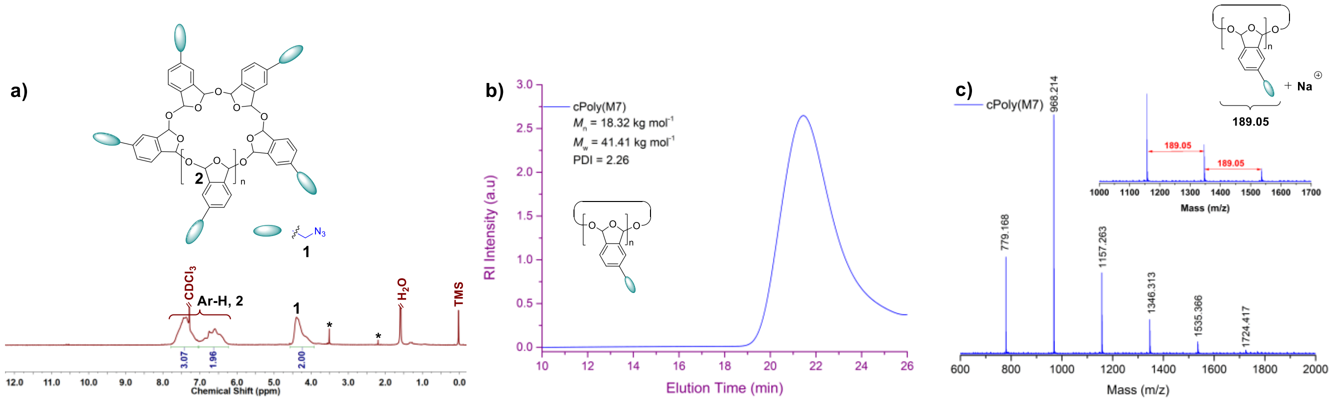

Figure S5: ^{1}H NMR spectrum (a, in $CDCl_3$), GPC curve (b) and Maldi-tof MS (c) of **cPoly(M7)**.

4. Synthesis of Water-soluble cPPAs

4.1 Synthesis of cPoly(M6)-P1

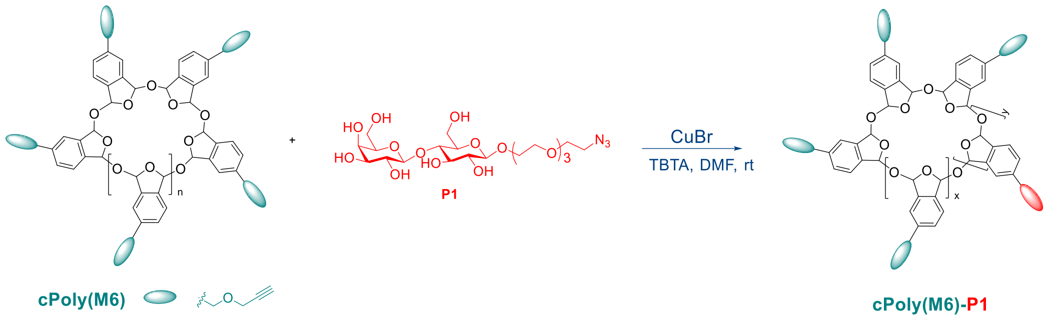

cPloy(M6) (60 mg, 2.06572 x 10^{-3} mmol, equal to 0.575 mmol of alkyne), P1 (161 mg, 0.296 mmol), CuBr (62.8 mg, 0.444 mmol, 1.5 eq. to P1) and TBTA (117 mg, .0222 mmol, 1.5 eq. to P1) were dissolved in 10 mL DMF and stirred at room temperature for 16h. The mixture solution was directly put into dialysis memberane (M_w =3000 g/mol) then placed in H_2O solution (generally change the water very 5 hours) for 5 days to purify. After that, the solution in dialysis memberane was directly freeze drying to give final product. **cPoly(M6)-1** was synthesized following the same procedure.

4.2 Synthesis of cPoly(M8)-P2, cPoly(M8)-P2/P4, cPoly(M8)-P3 and cPoly(M8)-P3/P4

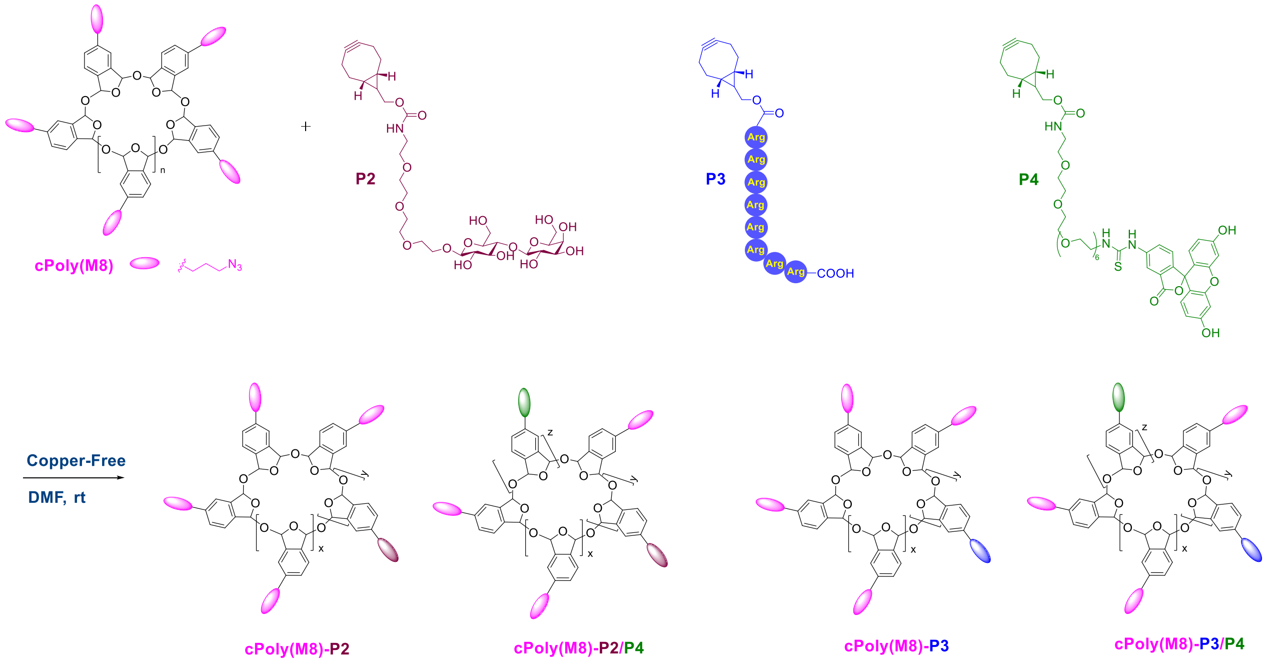

Scheme S2. The structures of payloads and their corresponding water-soluble cPPAs.

Synthesis of cPloy(M8)-P2: **cPloy(M8)** (5 mg, 1.107 x 10^{-4} mmol, equal to 0.023 mmol of $-N_3$) and P2 (7.6 mg, 0.011 mmol, 0.48 eq.) were dissloved in 2 mL DMF, then stirred at room temperature for 8h. The mixture solution was directly put into dialysis memberane (M_w=3000 g/mol) then placed in H_2O solution (generally change the water every 5 hours) for 3 days to give pure product in water solution.

Synthesis of cPloy(M8)-P2/P4: **cPloy(M8)** (5 mg, 1.107 x 10^{-4} mmol, equal to 0.023 mmol of $-N_3$) and P2 (7.6 mg, 0.011 mmol, 0.48 eq.) were dissloved in 2mL DMF , then stirred at room temperature for 6h. After that, P4 (8 mg, 0.008 mmol, 0.35 eq.) was dissloved in 1 mL DMF, then added into mixture solution then stirred at room temperature for 8h. The mixture solution was directly put into dialysis memberane (M_w=3000 g/mol) then placed in H_2O solution (generally change the water every 5 hours) for 3 days to give pure product in water solution.

Synthesis of cPloy(M8)-P3: **cPloy(M8)** (5 mg, 1.107 x 10^{-4} mmol, equal to 0.023 mmol of $-N_3$) was dissolved in 1mL DMF. P3 (16 mg, 0.011 mmol, 0.48 eq.) was dissloved in 1 mL DMF/H_2O (20/1, *v*/*v*) mixture solution then added to polymer solution and stirred at room temperature for 8h. The mixture solution was directly put into dialysis memberane (M_w=3000 g/mol) then placed in H_2O solution (generally change the water every 5 hours) for 3 days to give pure product in water solution.

Synthesis of cPloy(M8)-P3/P4: **cPloy(M8)** (5 mg, 1.107 x 10^{-4} mmol, equal to 0.023 mmol of $-N_3$) was dissolved in 1mL DMF. P3 (16 mg, 0.011 mmol, 0.48 eq.) was dissloved in 1 mL DMF/H_2O (20/1, *v*/*v*) mixture solution then added to polymer solution and stirred at room temperature for 6h. After that, P4 (8 mg, 0.008 mmol, 0.35 eq.) was dissolved in 1mL DMF then added to the mixture solution and stirred at room temperature for 8h. The mixture solution was directly put into dialysis memberane (M_w=3000 g/mol) then placed in H_2O solution (generally change the water every 5 hours) for 3 days to give pure product in water solution.

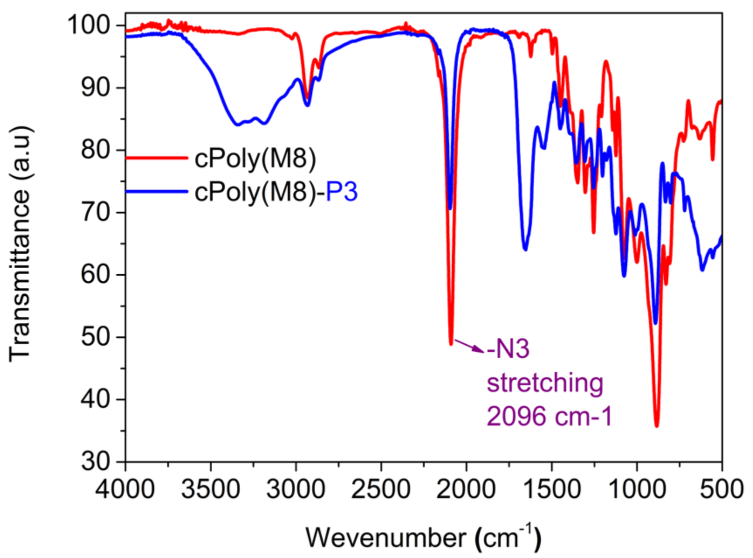

Figure S6. FTIR spectra of **cPoly(M8)** and **cPoly(M8)-P3**.

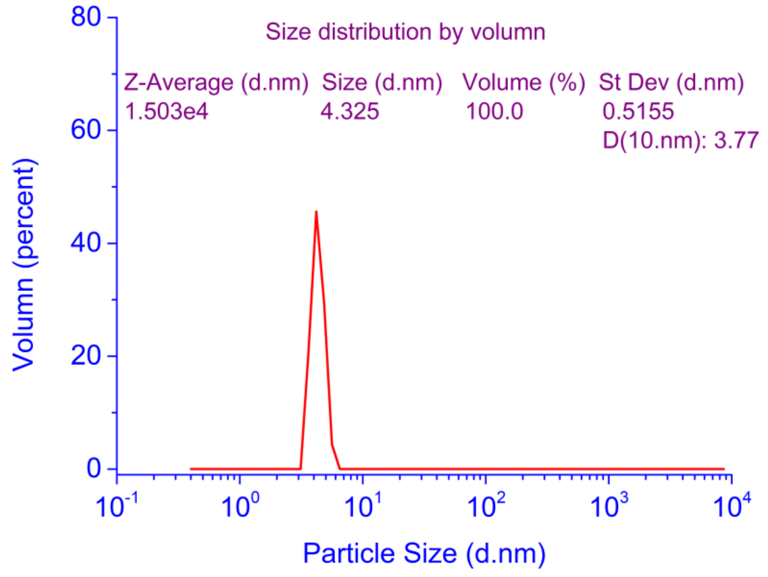

Figure S7. DLS results of **cPoly(M8)-P3** in water solution at the concentration of 5.0 mg/mL.

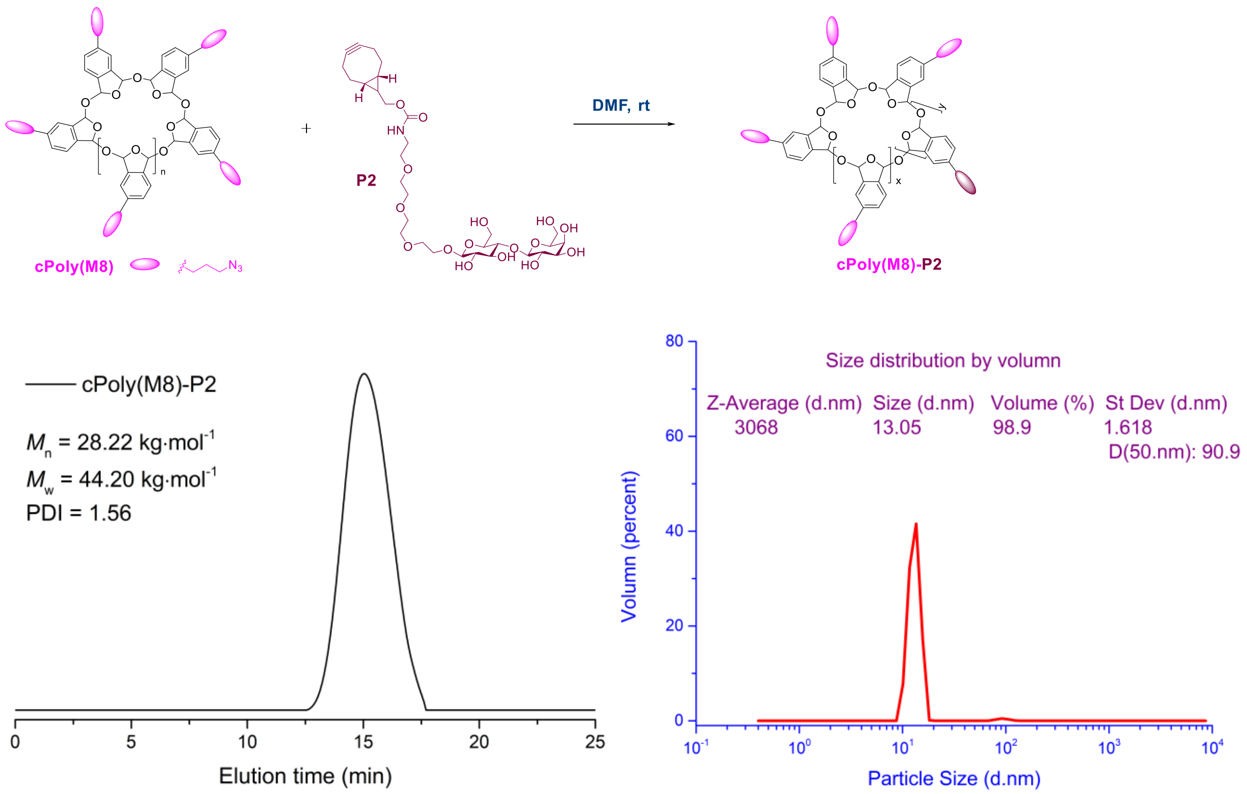

Figure S8. GPC (water as an eluent) and DLS (in water solution at the concentration of 4.6 mg/mL) results of **cPoly(M8)-P2**. **cPoly(M8)**: M_n = 14.51 kg·mol^{-1}, M_w = 26.41 kg·mol^{-1}, PDI: 1.82.

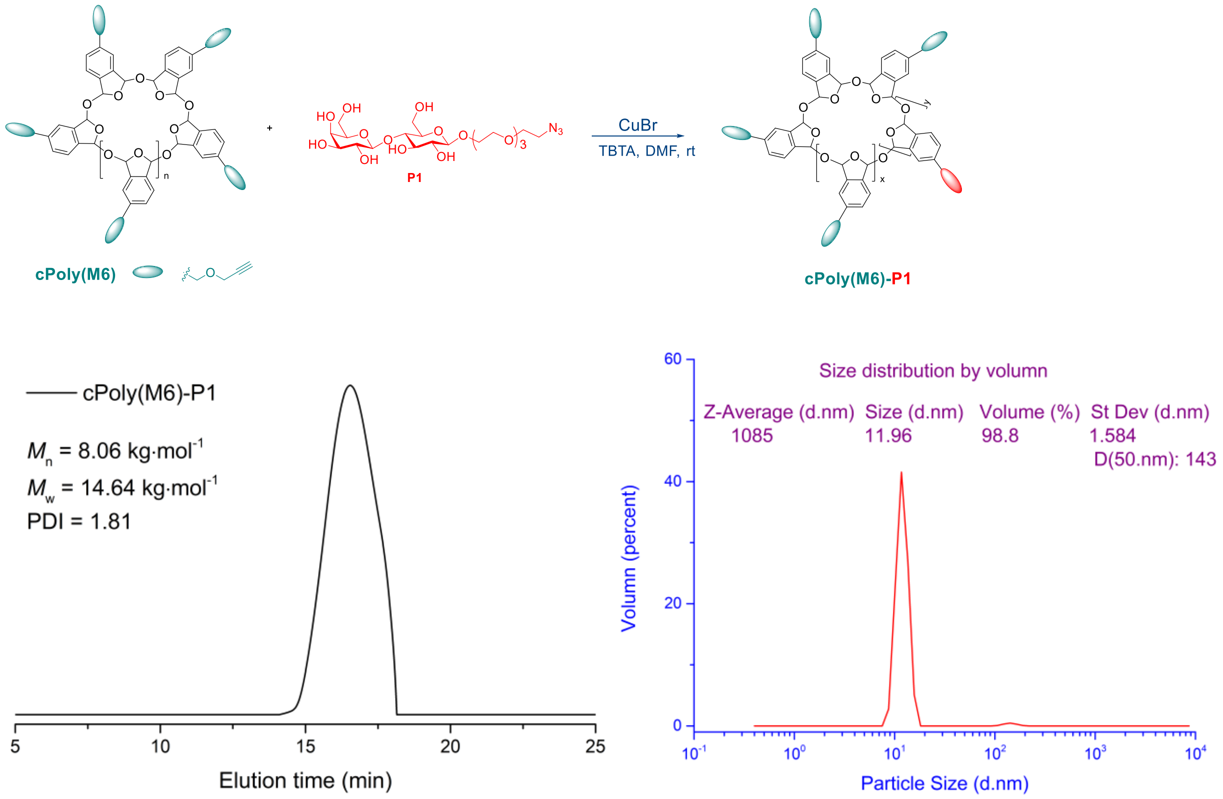

Figure S9. GPC (water as an eluent) and DLS (in water solution at the concentration of 8.0 mg/mL) results of **cPoly(M6)-P1**. **cPoly(M6)**: M_n = 3.75 kg·mol^{-1}, M_w = 8.78 kg·mol^{-1}, PDI: 2.34.

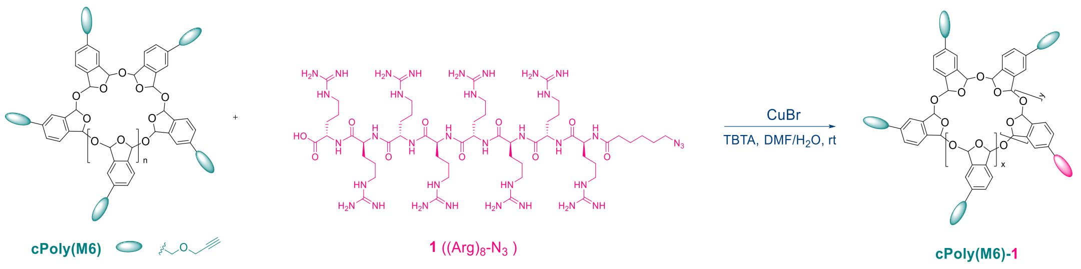

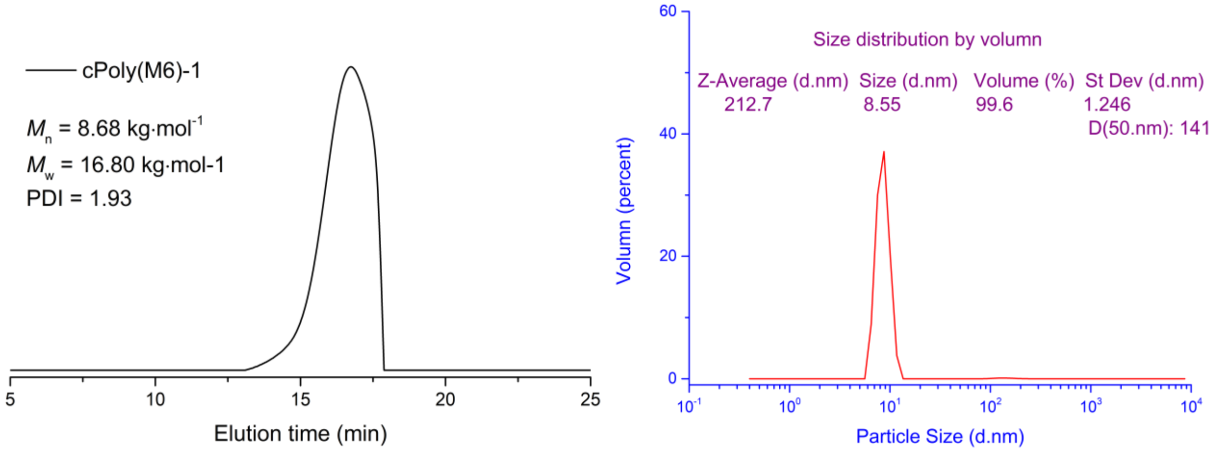

Figure S10. GPC (water as an eluent) and DLS (in water solution at the concentration of 8.0 mg/mL) results of **cPoly(M6)-1**. **cPoly(M6)**: $M_n = 3.75$ kg·mol^{-1}, $M_w = 8.78$ kg·mol^{-1}, PDI: 2.34.

5. Depolymerization of cPoly(M6)-P1 in different PBS buffers

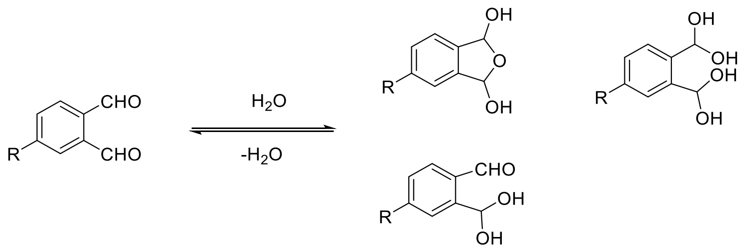

Scheme S3. The reversible reaction of OPA and its intermediates in water condition.

Depolymerization of **cPoly(M6)-P1** study was carried out in different PBS buffers (pH=6.0, pH=6.5, pH=7.0 and pH=7.4, respectively), and the reactions were monitored by ^{1}H NMR, the details of sample preparation are depicted in the following.

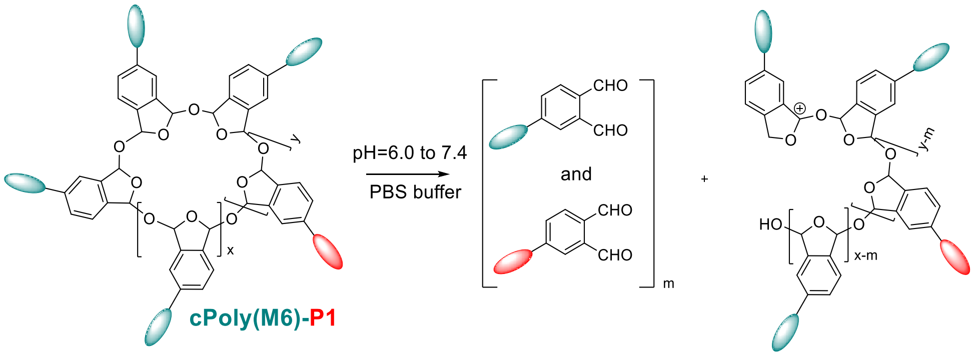

PBS buffer (pH=7.4)

cPoly(M6)-P1 (3.6 mg) was dissolved in 3.6 mL 1x PBS aqueous buffer (pH=7.4), then the sample solution was placed to water bath at 37 °C under stirring. Taking 0.5 mL solution from system every time, and the solution was directly freeze-dried, then the dried sample was dissolved in 0.5 mL DMSO-d_6 (1,3-dioxane as internal standard, concentration at 0.25 μL/mL in DMSO-d_6) to carry out NMR. The period of interval time of 2, 3, 5, 7 and 24 hours were examined.

PBS buffer (pH=7.0)

cPoly(M6)-P1 (3.6 mg) was dissolved in 3.6 mL 1x PBS aqueous buffer (pH=7.0), then the sample solution was placed to water bath at 37 °C under stirring. Taking 0.5 mL solution from system every time, and the solution was directly freeze-dried, then the dried sample was dissolved in 0.5 mL DMSO-d_6 (1,3-dioxane as internal standard, concentration at 0.25 μL/mL in DMSO-d6) to carry out NMR. The period of interval time of 4, 6, 8 and 10 hours were studied.

PBS buffer (pH=6.5)

cPoly(M6)-P1 (3.6 mg) was dissolved in 3.6 mL 1x PBS aqueous buffer (pH=6.5), then the sample solution was placed to water bath at 37 °C under stirring. Taking 0.5 mL solution from system every time, and the solution was directly freeze-dried, then the dried sample was dissolved in 0.5 mL DMSO-d_6 (1,3-dioxane as internal standard, concentration at 0.25 μL/mL in DMSO-d_6) to carry out NMR. The period of interval time of 2, 3, 4, 5, 6 and 7 hours were examined.

PBS buffer (pH=6.0)

cPoly(M6)-P1 (3.6 mg) was dissolved in 3.6 mL 1x PBS aqueous buffer (pH=6.0), then the sample solution was placed to water bath at 37 °C under stirring. Taking 0.5 mL solution from system every time,

and the solution was directly freeze-dried, then the dried sample was dissolved in 0.5 mL DMSO-d_6 (1,3-dioxane as internal standard, concentration at 0.25 µL/mL in DMSO-d_6) to carry out NMR. The period of interval time of 2, 3, 4, 5, 6 and 7 hours were examined.

Table S2. The depolymerization kinetic results of **cPoly(M6)-P1**.

Time/h	OPA/ pH=6.0	OPA/ pH=6.5	OPA/ pH=7.0	OPA/ pH=7.4
2[a]	4.51	0	0	0
3[a]	5.03	0	0	0
4[a]	5.91	0	0	0
5[a]	6.31	4.52	0	0
6[a]	6.79	5.16	0	0
7[a]	8.00	5.59	0	0
Full conversion[b]	22.17	22.17	22.17	22.17

[a] the value of *o*-PA represents the generation of di-formyl group during depolymerization process, data obtained from Figure S14 and S15, refer to 1,3-dioxane as internal standard. [b] Full conversion represents the **cPoly(M6)-P1** was fully depolymerized and converted into monomers, the value was obtained via the addition of HCl to the testing NMR sample solution for 10 mins, see Figure S14(d).

Table S3. The depolymerization coversion of **cPoly(M6)-P1**.

Time/h	pH=6.0	pH=6.5	pH=7.0	pH=7.4
2	20.34	0	0	0
3	22.69	0	0	0
4	26.66	0	0	0
5	28.46	20.39	0	0
6	30.63	23.27	0	0
7	36.08	25.21	0	0

The conversion of cPoly(M6)-P1 was obtained based on the calculation in Table S2.

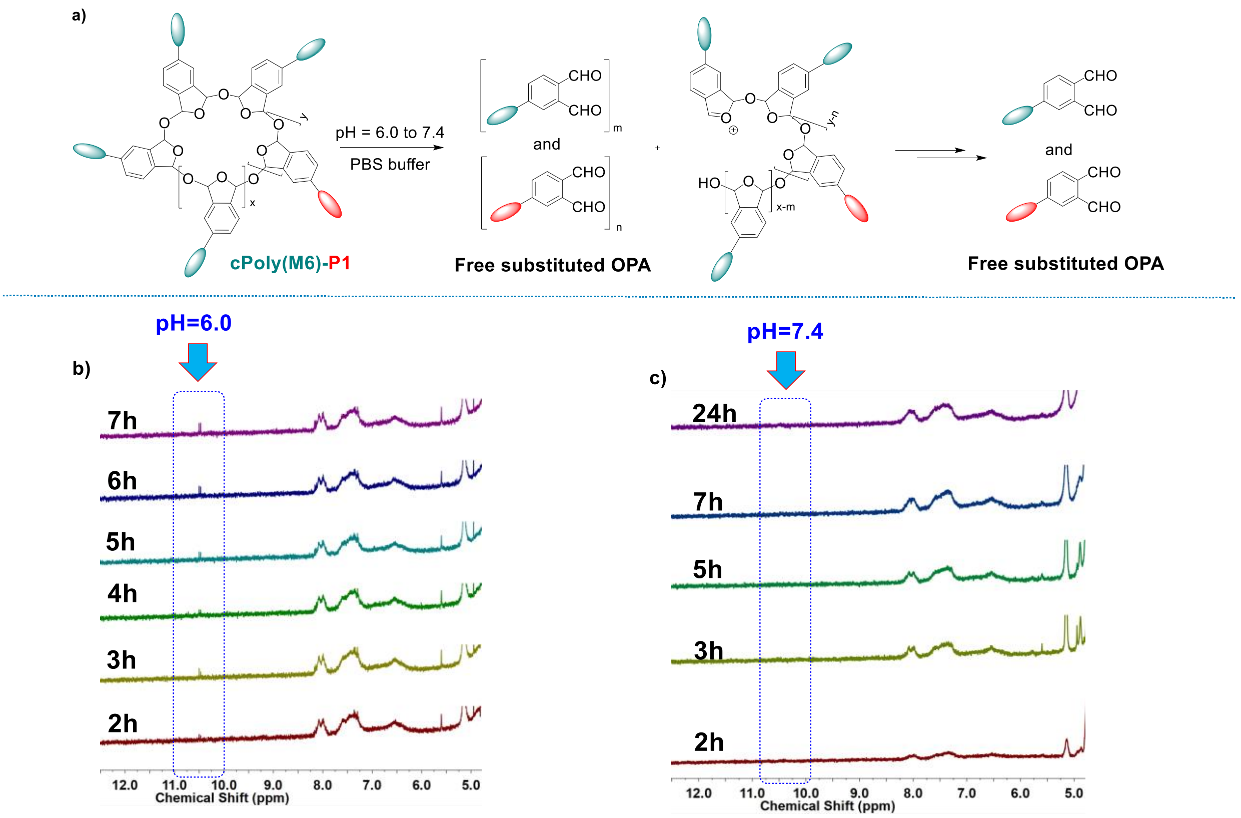

Figure S11. a) Schematic illustration of acid-triggered depolymerization of cPoly(M6)-P1. b & c) ^{1}H-NMR (in DMSO-d_6) spectra of **cPoly(M6)-P1** at different time points at pH = 6.0 and 7.4, respectively.

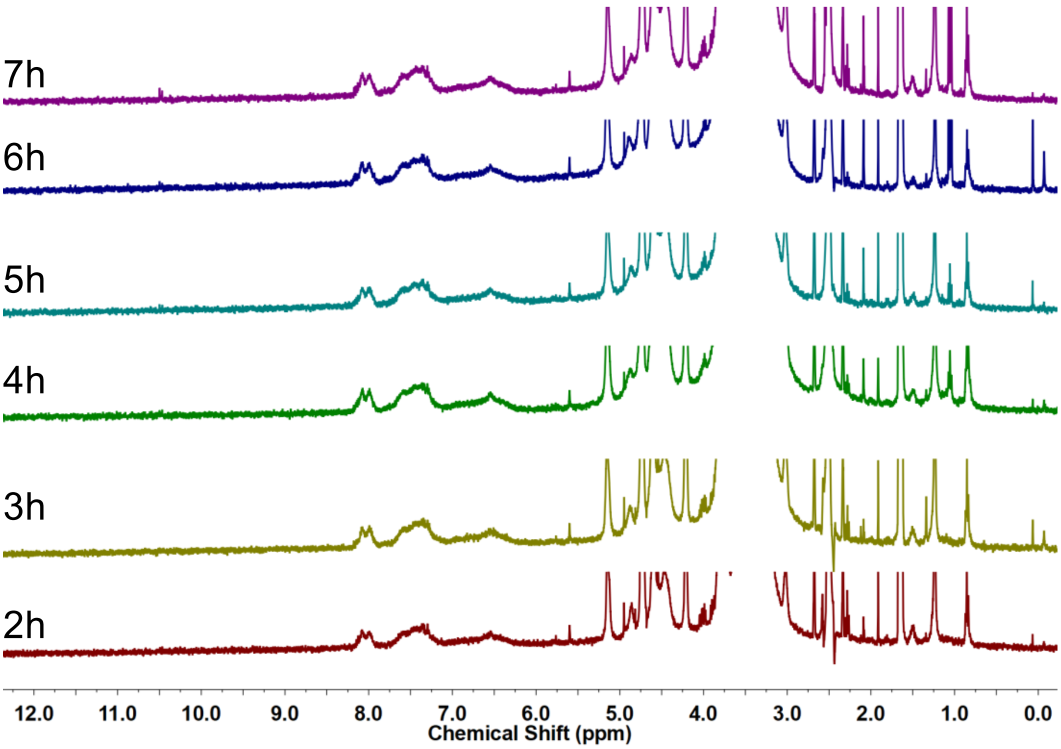

Figure S12. ^{1}H NMR spectra (in DMSO-d_6) of **cPoly(M6)-P1** in pH=6.5 PBS buffer, measured at 2, 3, 4, 5, 6 and 7h respectively, the integration value of di-formyl group see details in Figure S14.

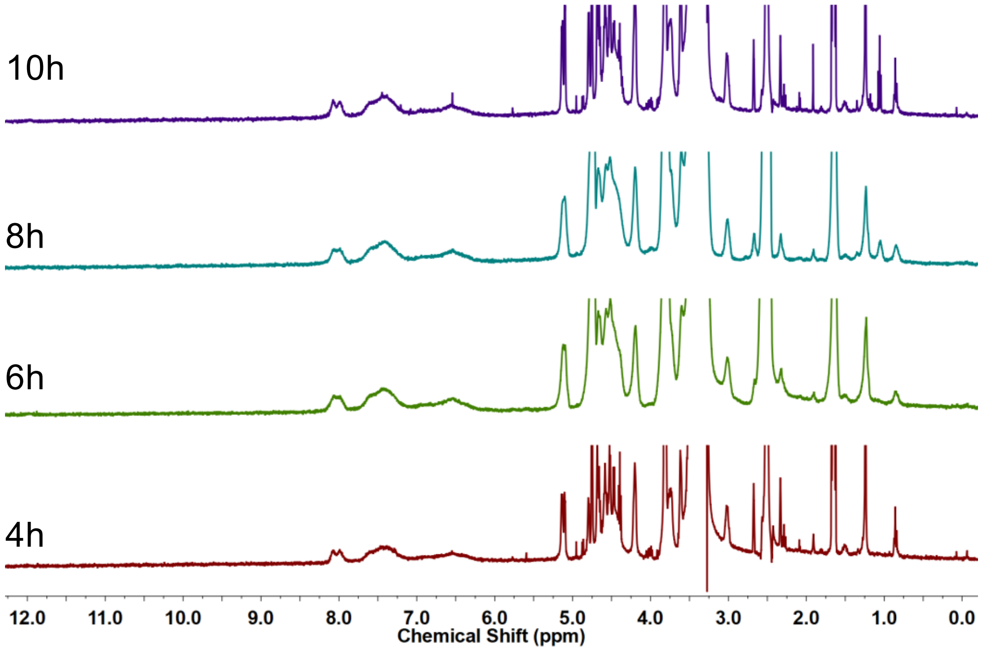

Figure S13. ^{1}H NMR spectra (in DMSO-d_6) of **cPoly(M6)-P1** in pH=7.0 PBS buffer, measured at 4, 6, 8 and 10h respectively.

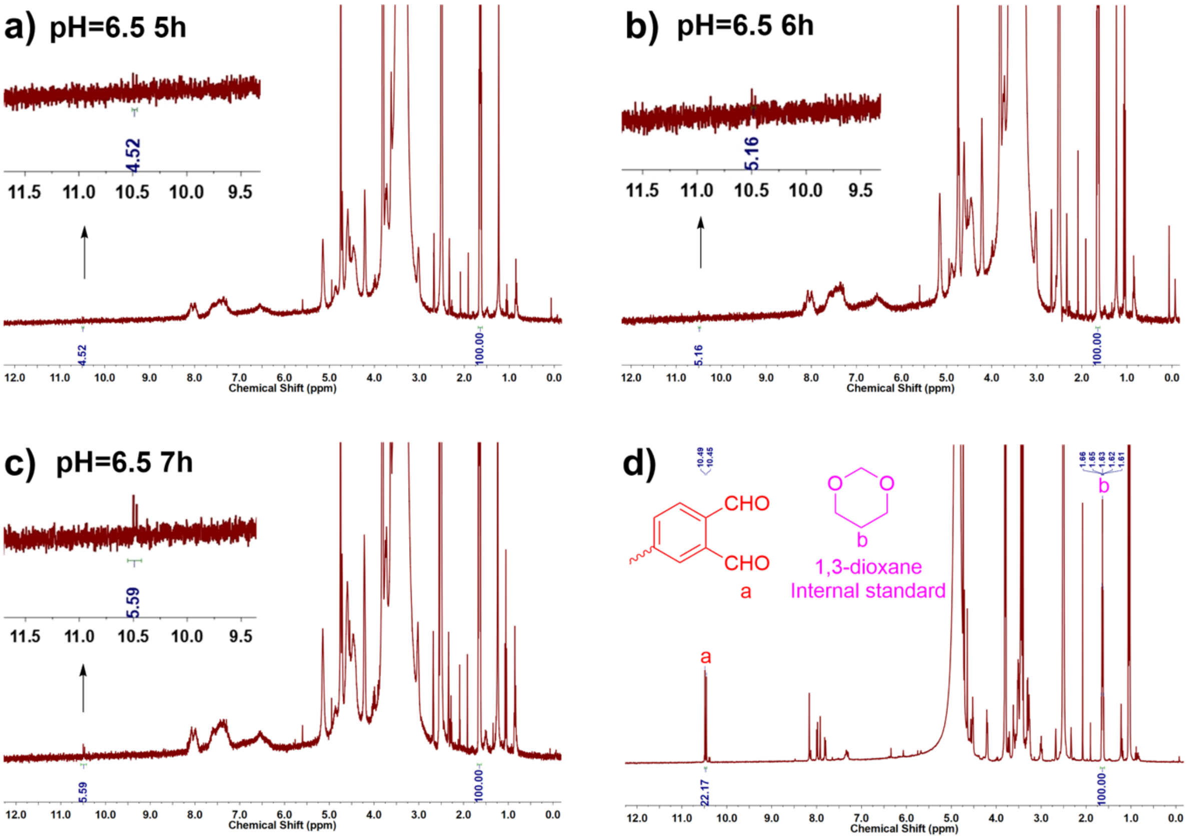

Figure S14: a-c) ^{1}H NMR spectra (in DMSO-d_6) of **cPoly(M6)-P1** in pH=6.5 PBS buffer at 5, 6 and 7h respectively. d) ^{1}H NMR spectrum of **cPoly(M6)-P1**, the sample was measured after the addition of HCl (50 μL, 12 mmol/L) into testing sample for 10 mins.

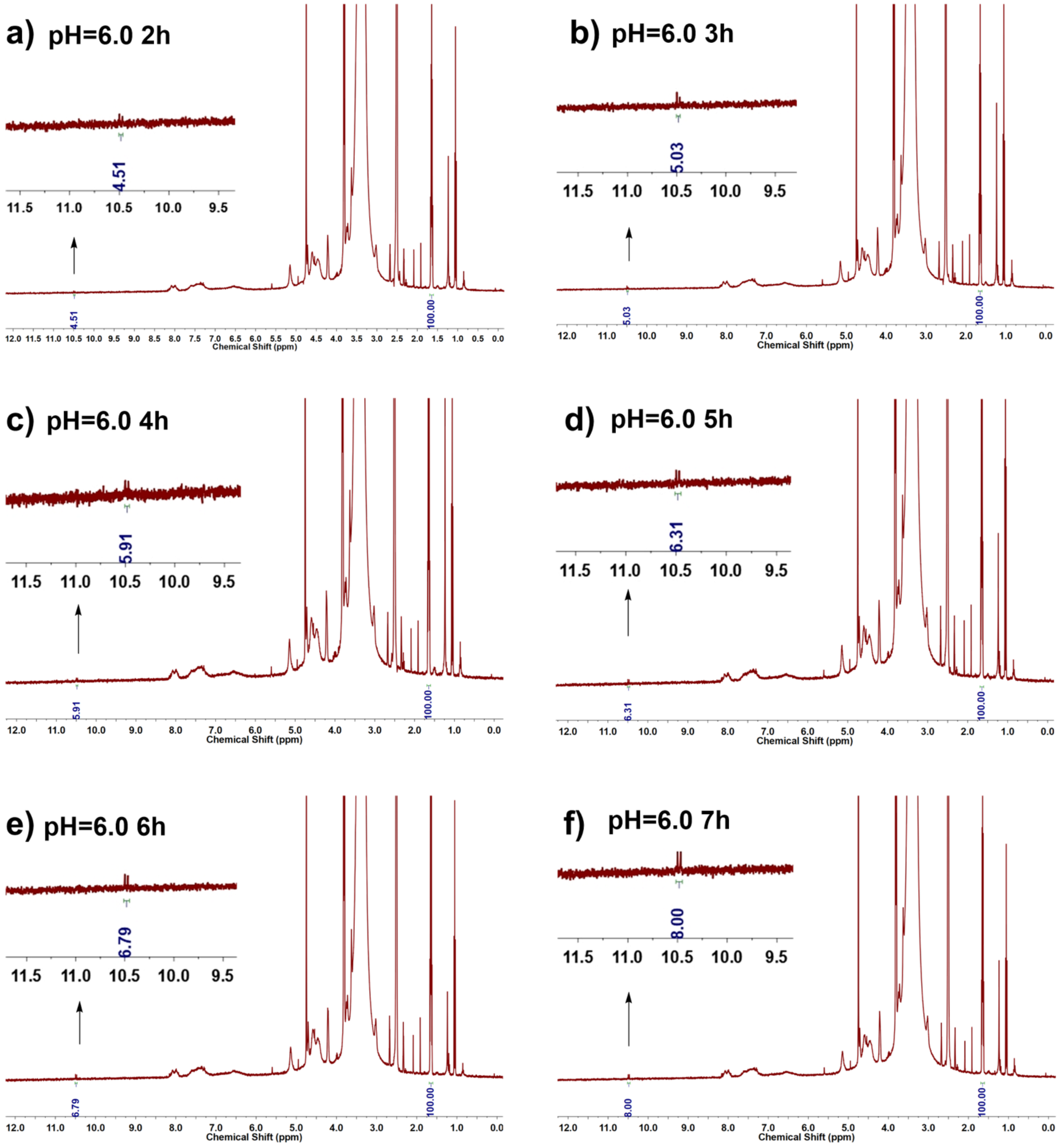

Figure S15. a-f) ^{1}H NMR spectra (in DMSO-d_6) of **cPoly(M6)-P1** in pH=6.0 PBS buffer at 2, 3 ,4, 5, 6 and 7h respectively.

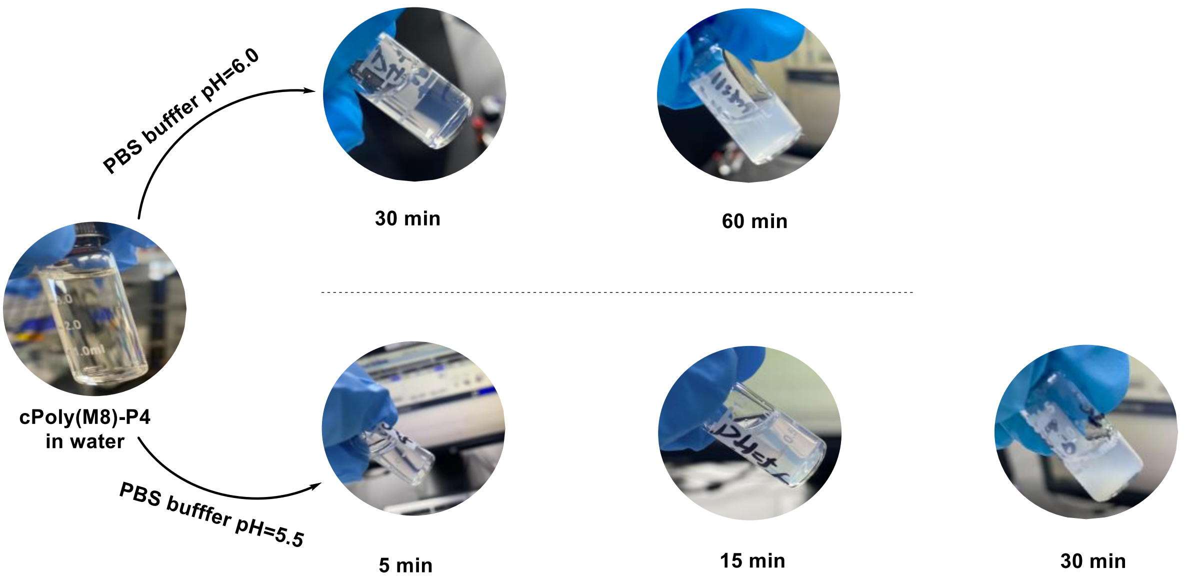

Figure S16. Photo images of **cPoly(M8)-P4** in pH=5.5 and pH=6.0 at different time.

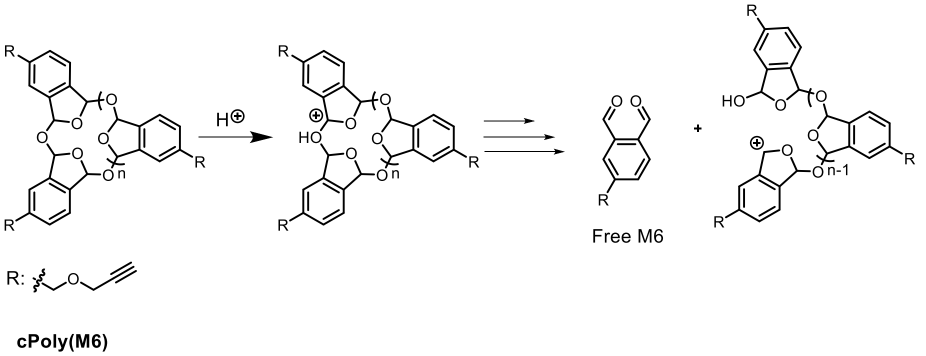

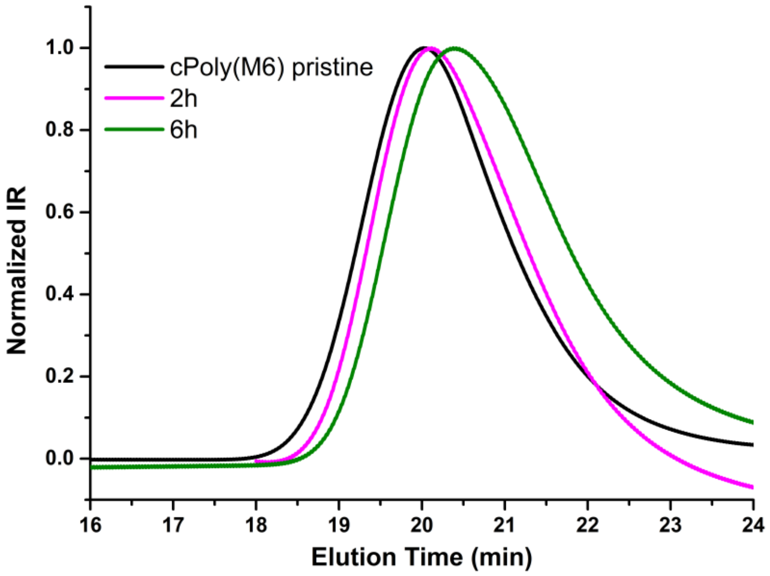

Figure S17. GPC curves of **cPoly(M6)** pristine and **cPoly(M6)** in THF/water mixture solution under pH=6.0 at 2 and 6 hours.

6. Cellular uptake study of cPoly(M8)-P2/P4 and cPoly(M8)-P3/P4

6.1 Cell culture

A549 cell line (Human Fenghui Biotechnology, CL0024) was purchased from Human Fenghui Biotechnology on December 16th 2022 and confirmed to be free of mycoplasma contamination. It was cultured in Ham's F-12K (Kaighn's) medium supplemented with 10% fetal bovine serum (FBS) and 1% penicillin-streptomycin. All cultures were maintained at 37 °C in a 5% CO_2 humidified atmosphere and used within 20 passages.

6.2 Cell viability assay

Cells were seeded in 96-well plate at a density of 5 x 10^3 cells/well for one day before the experiment. Cells were treated with **cPoly(M8)-P3** with different concentrations as specified and further incubated for 24 h and 48 h. cells were then treated with cell counting kit-8 (CCK-8) solution. After 2 h incubation at 37 °C followed by measuring the absorbance at 450 nm on Biotek (Synergy HTX). The data were normalized to the value of cells treated with the vehicle.

6.3 Cellular uptake assay

For the cellular uptake of polymer, A549 cells were seeded on 35 mm glass bottom confocal dishes (BIOFIL) and then treated with 50 nM of **cPoly(M8)-P2/P4** and of **cPoly(M8)-P3/P4**. At scheduled time points, the cells were washed three times with PBS and then stained with 0.2 mg/ml Hoechst 33342 (Thermo Fisher Scientific) and 2.5 mg/ml Wheat Germ Agglutinin (WGA) (Thermo Fisher Scientific) for 15 min at 37 °C before confocal imaging.

For the lysosome staining, the cell was treated with **cPoly(M8)-P3/P4**. At scheduled time points, the cells were washed three times with PBS and then stained with 0.2 mg/ml Hoechst 33342 (Thermo Fisher Scientific), 2.5 mg/ml Wheat Germ Agglutinin (WGA) (Thermo Fisher Scientific) and 50 nM LysoTracker Red DND-99 (Thermo Fisher Scientific) for 15 min at 37 °C before confocal imaging.

6.4 OPA-FITC for cell labelling

A549 cells were seeded on 35 mm glass bottom confocal dishes (BIOFIL) and the medium was discarded and the cells were washed three times with PBS, then treated with 10 uM OPA-FITC for 15 min in PBS and then stained with 0.2 mg/ml Hoechst 33342 (Thermo Fisher Scientific) and 2.5 mg/ml Wheat Germ Agglutinin (WGA) (Thermo Fisher Scientific) for 15 min at 37 °C before confocal imaging (Leica Microsystems).

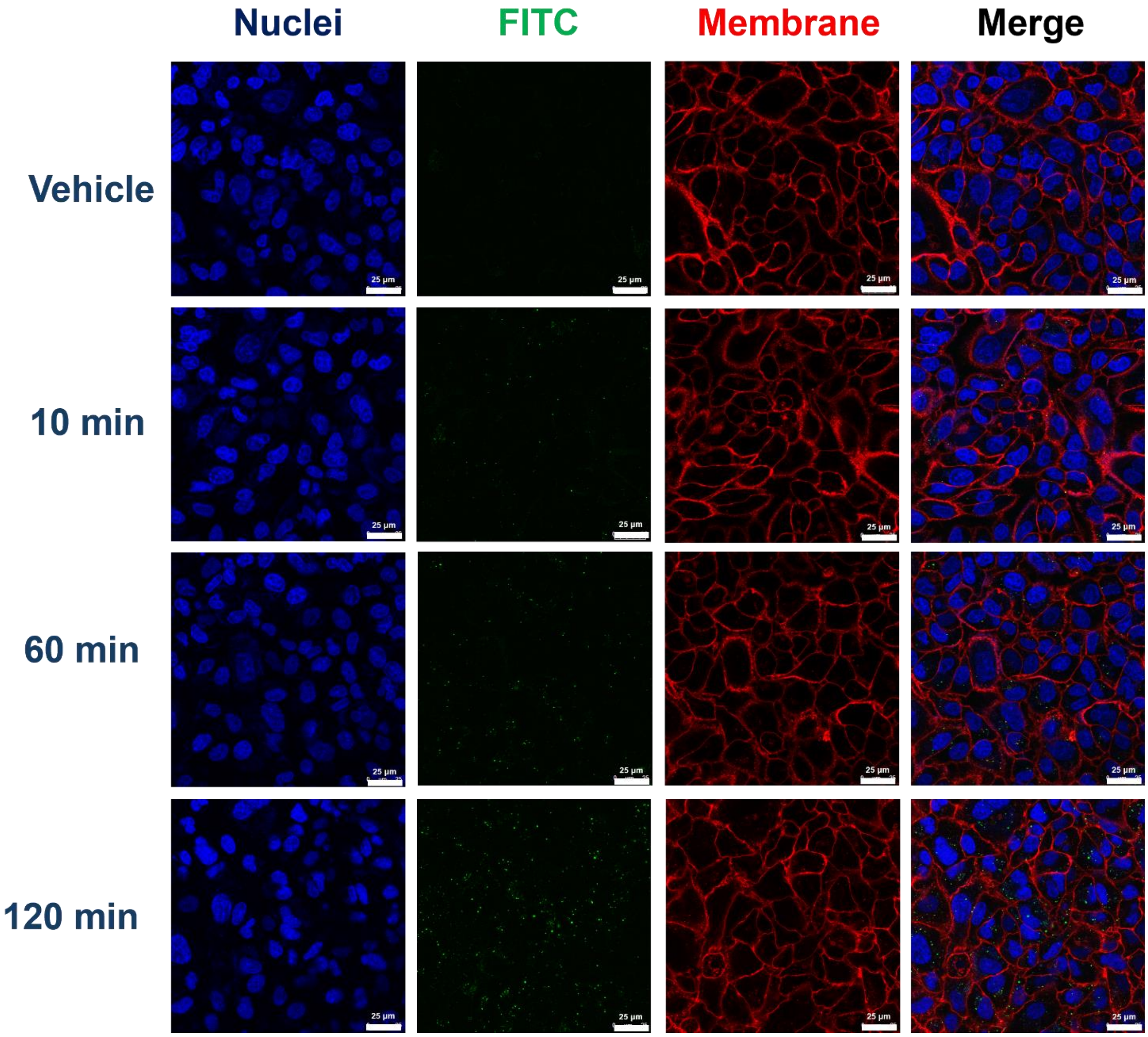

Figure S18. Confocal macroscopy images of **cPoly(M8)-P3/P4** (50 nM) on A549 cells at different time. Scale bars: 25 μm.

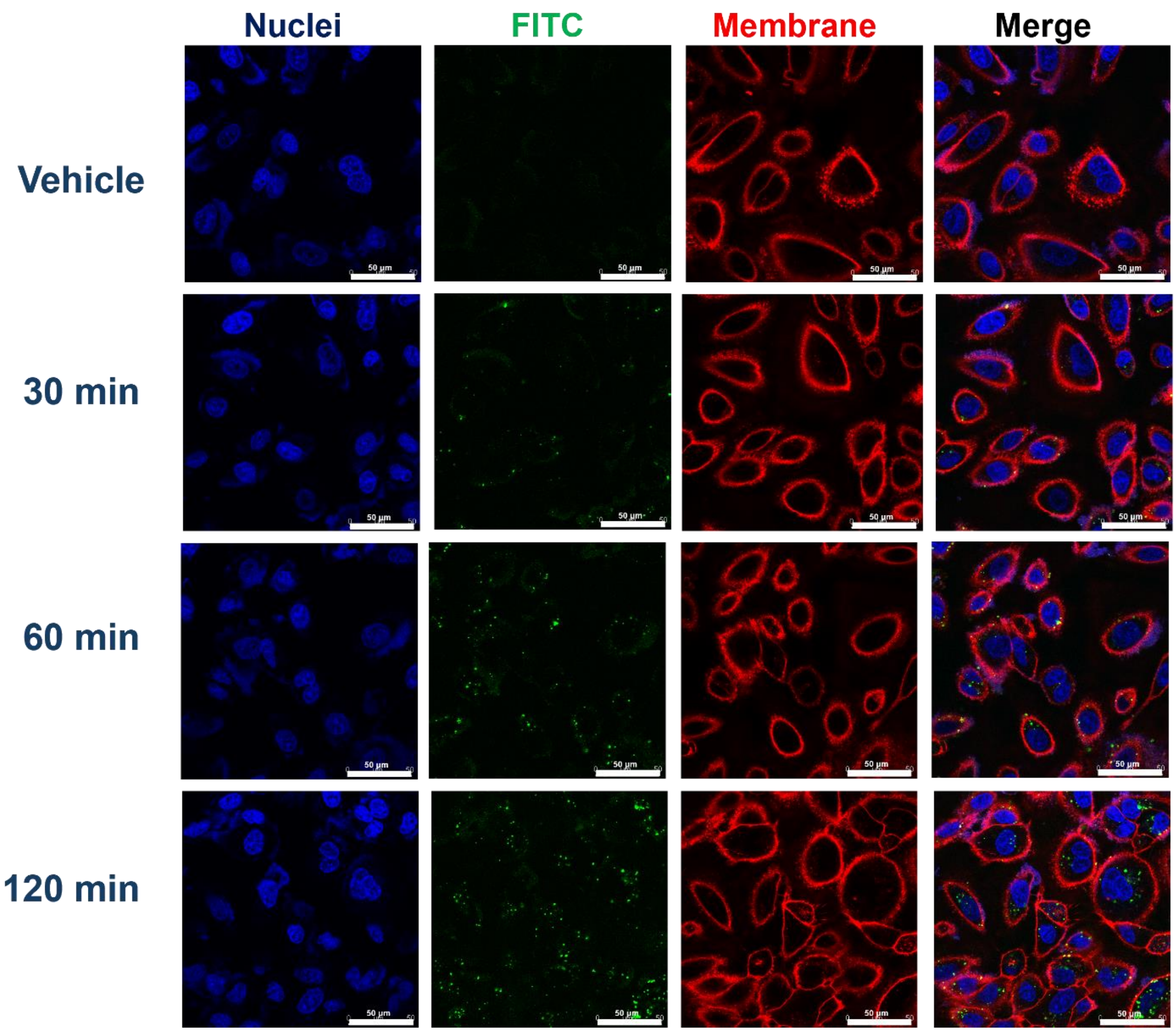

Figure S19. Confocal macroscopy images of **cPoly(M8)-P3/P4** (2 μM) on A549 cells at different time. Scale bars: 50 μm.

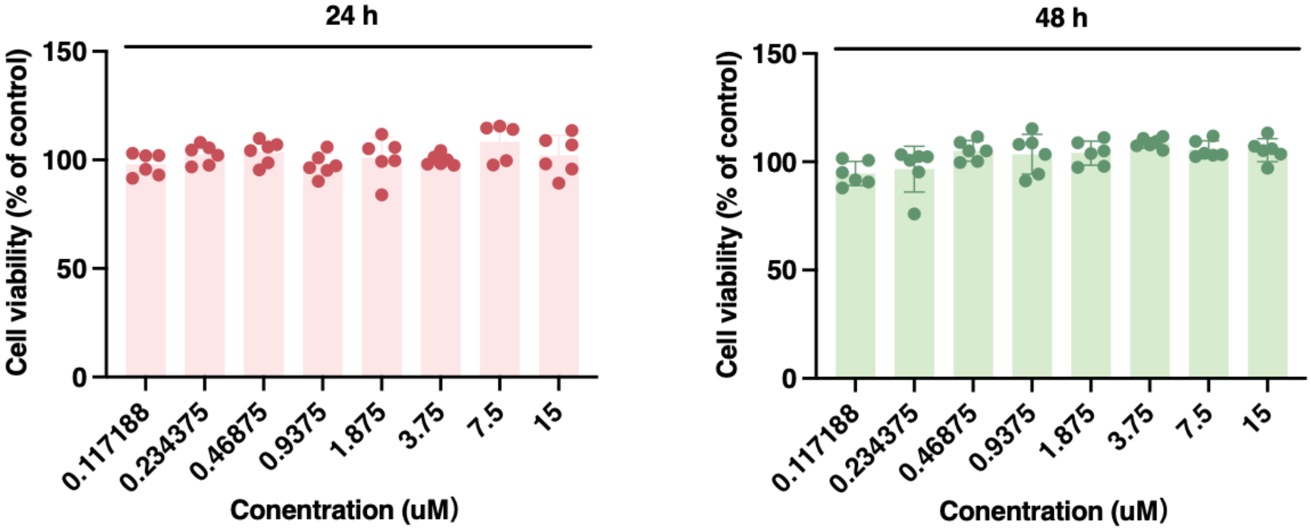

Figure S20. *In vitro* cell viability studies of **cPoly(M8)-P3** in A549 cells within differnt concentrations.

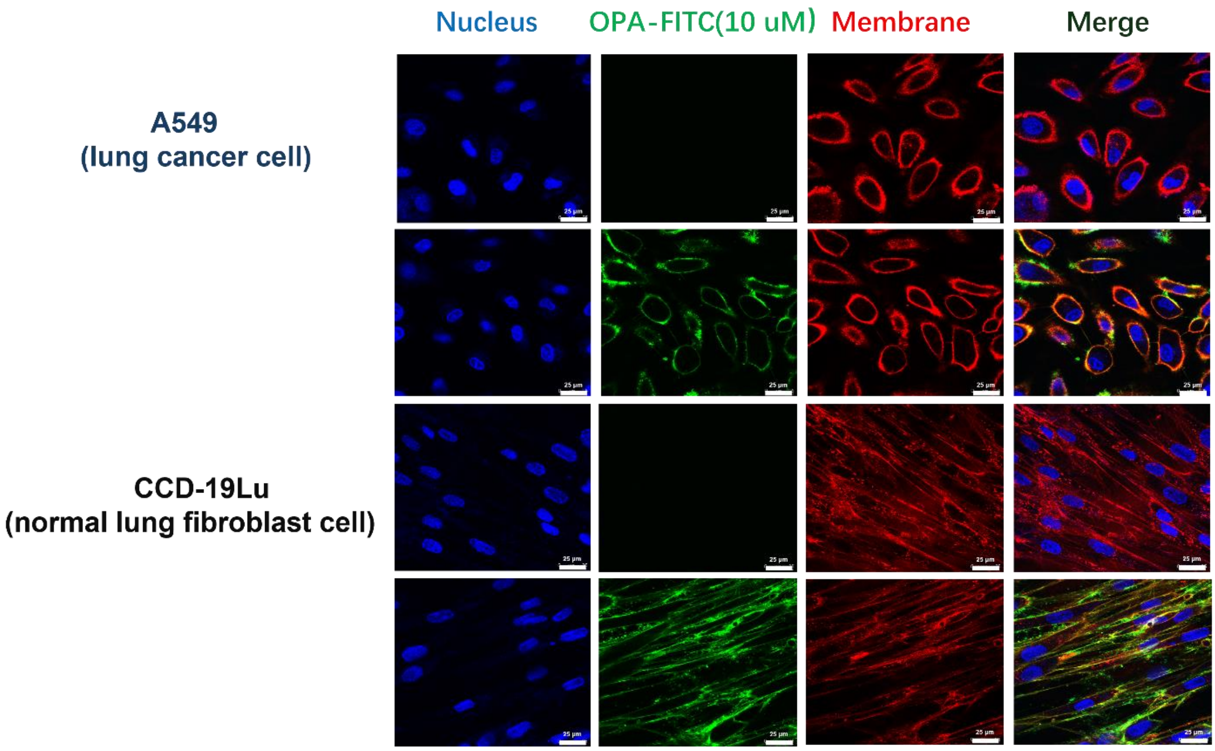

Figure S21. Confocal macroscopy images of OPA-FITC (10 μM) on A549 and CCD-19Lu cells. Scale bars: 25 μm.

7. Pulldown experiment

7.1 Processing of treated cell lysate for sodium dodecyl-sulfate polyacrylamide gel electrophoresis (SDS-PAGE) and subsequent in-gel digestion

The treated A549 cells were washed 5 times using ice-cold 1x phosphate buffered saline (PBS, pH 7.4). After washing, cells were lysed as previously reported.[8] Briefly, cells were suspended in lysis buffer (25 mM Tris, 150 mM NaCl, 5 mM EDTA and 1% CHAPS (v/v), pH 7.4) prior to homogenization and sonication by a VCX800 Vibra-Cell ultrasonic processor (Sonics & Materials, Inc). The lysis buffer was freshly made. Cell lysates were centrifugated at 4 °C for 10 minutes, and the supernatant for each sample was collected. Biotin-BCN **3** was added to each supernatant in the ratio of 1:10 (1 additive to 10, biotin-BCN), then incubated at 4 °C overnight. After incubation, the mixture was dialyzed against 50 mM ammonia bicarbonate (AMBIC) buffer (pH 7.5) at 4 °C for 24 hours, and the buffer was regularly changed. Protein concentration of each sample was determined using NanoDrop™ Lite spectrophotometer (Thermo Scientific). Another dialysis was performed in 1x PBS binding buffer (pH 7.4, 0.05% Tween-

20 was added to reduce non-specific binding) at 4 °C for 48 hours, and the buffer was regularly changed. After the dialysis, the sample was collected and then 250 µL of streptavidin magnetic beads (#S1420S, New England Biolabs) was added to each sample and incubated at 4 °C overnight. After pulldown which was based on the high specificity, high affinity interaction between biotin and streptavidin, all above-mentioned samples were then boiled at 100 °C with sodium dodecyl sulfate (SDS) loading buffer and used for sodium dodecyl sulfate–polyacrylamide gel electrophoresis (SDS-PAGE) (12% gel) analysis, followed by Coomassie blue staining. The stained gel was scanned using BIO-RAD ChemiDoc MP Imaging System and processed using Image Lab Touch Software (version 2.0.0.25). Subsequent in-gel digestion was performed as previously described.[9-11] The gel bands were cut into small pieces (around 1 x 1 mm) and then destained using 100 mM AMBIC/acetonitrile (1:1, vol/vol) twice. The gel bands were then treated with dithiothreitol and subsequently with iodoacetamide. Mass spectrometry (MS) grade trypsin (#90057, Thermo Scientific) was added in the ratio of 1:20 (1 enzyme to 20 protein) and the digestion was incubated at 37 °C overnight. The digested peptides were processed using Pierce™ C18 tips (#87784, Thermo Scientific) and then dried using a Savant™ SpeedVac™ (Thermo Scientific) prior to MS analysis.

7.2 Liquid chromatography-mass spectrometry (LC-MS) for proteomics and mass spectrometry (MS) data analysis

The samples were analyzed using Orbitrap™ Ascend Tribrid™ mass spectrometer (Thermo Scientific) with a Vanquish™ Neo UHPLC System (Thermo Scientific). The instrument was operated in a data-dependent mode. The MS precursor scan was performed using an Orbitrap detector at resolution set to 120,000 in positive mode, automatic gain control (AGC) target was set to be 4e5, and maximum injection time (IT) was 251 ms, MS/MS fragment ions were analyzed using an Orbitrap detector at resolution set to 15,000, the AGC target was set to be 5e4, and maximum IT was 27 ms, the peptide ions with charge states 2-6 were sequentially isolated and analyzed using normalized collision energy (NCE).

The MS raw data were searched by software Byonic (version 5.1) against the human proteome fasta sequences from UniProt human database, and the protein false discovery rate (FDR) cutoff value was set

to be 1%. Different search parameters were allowed for the protein identity check, with carboxyamidomethylation of cysteine as a fixed modification, oxidation of methionine, deamidation of asparagine and glutamine, phosphorylation of serine, threonine and tyrosine as a variable modification. Trypsin was selected as the digestion enzyme, specific cleavage sites (the carboxyl side of lysine and arginine) were enabled, and up to 1 missed cleavage was allowed. The digestion specificity was set to be fully specific. Mass tolerance for precursor ions was set to be 30 ppm, mass tolerance for fragment ions was set to be 30 ppm. Byonic searching results were filtered with PEP 2D score < 0.001, then the remaining proteins were further screened using UniProt data category subcellular location cellular component (SLCC) showing presence in either endosome or lysosome, the result was shown in Venn diagram using software TBtools-II.[12]

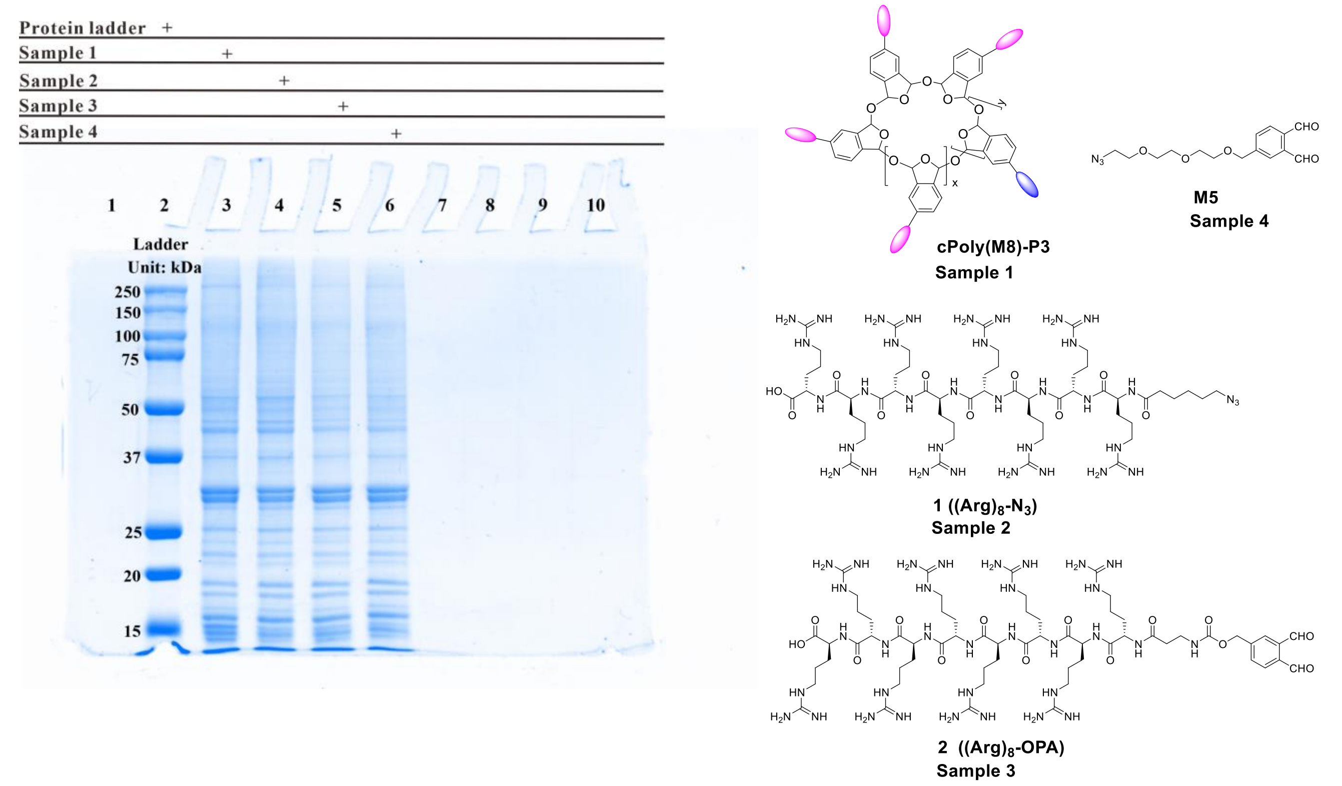

Figure S22. SDS-PAGE result of the 4 pull-down samples, and the structures of 4 samples. lane 1: protein ladder; lane 2: Sample 1; lane 3: Sample 2; lane 4: Sample 3; lane 5: Sample 4. The gel was scanned using BIO-RAD ChemiDoc MP Imaging System and processed using Image Lab Touch Software and Image Lab program\.

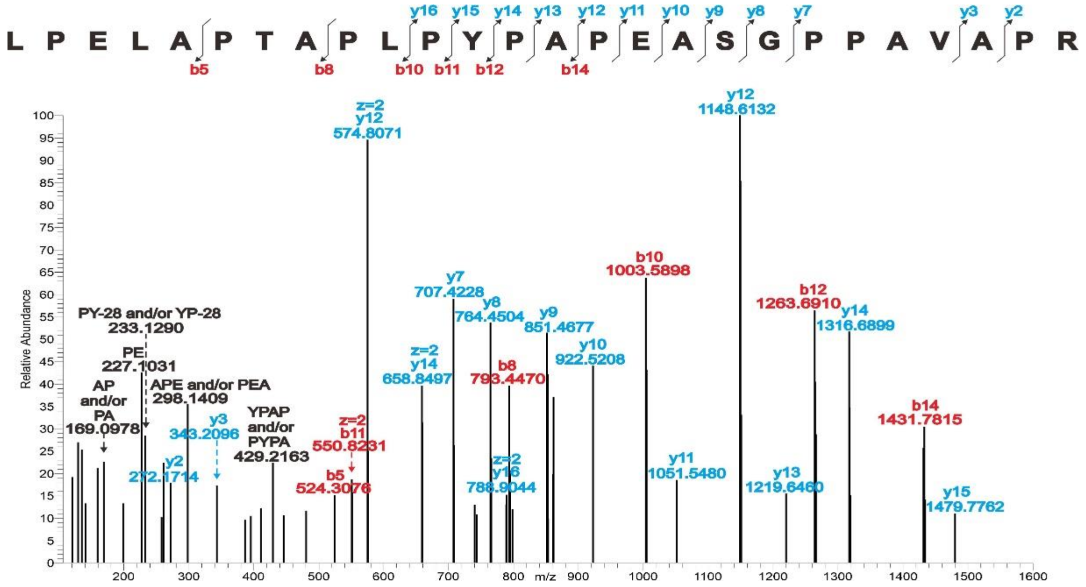

Figure S23. Annotated ESI-Orbitrap MS/MS spectrum of the peptide LPELAPTAPLPYPAPEASGPPAVAPR from vacuolar protein sorting-associated protein 37B (Q9H9H4). This peptide was obtained after trypsin treatment. All ions are $[P + H]^+$ or $[P + 2H]^{2+}$. Double charged ions are annotated as z = 2, and others are monocharged. The number indicated above the peak in the spectrum is the m/z value of the ion that has been detected by the mass spectrometer. To make the annotation clearer, ions are labeled in different colors.

Figure S23 displays an annotated MS/MS spectrum of the peptide LPELAPTAPLPYPAPEASGPPAVAPR from vacuolar protein sorting-associated protein 37B, and this protein has already been reported on endosome.[13] This protein is 1 of the 10 unique proteins in sample 1. As shown in the figure, its 6 peptide 'b' ions (b5, b8, b10-b12, and b14) and 12 peptide 'y' ions (y2, y3, and y7-y16) clearly demonstrate the peptide sequence information, which supports the presence of this protein in sample 1.

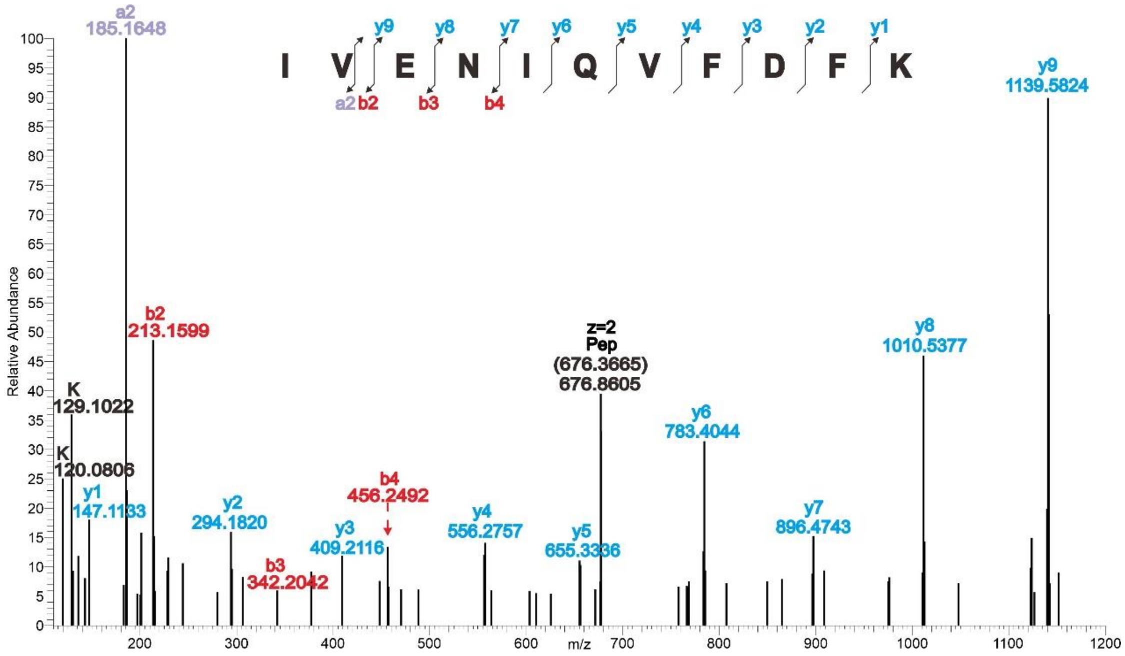

Figure S24. Annotated ESI-Orbitrap MS/MS spectrum of the peptide IVENIQVFDFK from aldo-keto reductase family 1 member B10 (O60218). This peptide was obtained after trypsin treatment. All ions are $[P + H]^+$ or $[P + 2H]^{2+}$. Double charged ions are annotated as z = 2, and others are monocharged. The number indicated above the peak in the spectrum is the m/z value of the ion that has been detected by the mass spectrometer. The number indicated in the brackets is the theoretical m/z value of the ion, and the difference between the detected and the theoretical can be due to the fact that the first monoisotopic peak is missing. To make the annotation clearer, ions are labeled in different colors.

Figure S24 displays an annotated MS/MS spectrum of the peptide IVENIQVFDFK from aldo-keto reductase family 1 member B10, and this protein has already been reported on lysosome.[14] This protein is 1 of the 10 unique proteins in sample 1. As shown in the figure, its 1 peptide ‘a’ ion (a2), its 3 peptide ‘b’ ions (b2-b4) and 9 peptide ‘y’ ions (y1-y9) clearly demonstrate the peptide sequence information, which supports the presence of this protein in sample 1.

Table S4. Detailed information of all unique proteins in each sample

Sample name	No.	UniProt No.	Protein name	Location (lysosome or/and endosome)
Sample 1	1	O60218	Aldo-keto reductase family 1 member B10 (EC 1.1.1.300) (EC 1.1.1.54) (ARL-1) (Aldose reductase-like) (Aldose reductase-related protein) (ARP) (hARP) (Small intestine reductase) (SI reductase)	Lysosome[14]
	2	P20645	Cation-dependent mannose-6-phosphate receptor (CD Man-6-P receptor) (CD-MPR) (46 kDa mannose 6-phosphate receptor) (MPR 46)	(late) endosome[15]
	3	P55735	Protein SEC13 homolog (GATOR complex protein SEC13) (SEC13-like protein 1) (SEC13-related protein)	---
	4	Q13501	Sequestosome-1 (EBI3-associated protein of 60 kDa) (EBIAP) (p60) (Phosphotyrosine-independent ligand for the Lck SH2 domain of 62 kDa) (Ubiquitin-binding protein p62)	late endosome or lysosome[16]
	5	Q8IV08	5'-3' exonuclease PLD3 (EC 3.1.16.1) (Choline phosphatase 3) (HindIII K4L homolog) (Hu-K4) (Phosphatidylcholine-hydrolyzing phospholipase D3) (Phospholipase D3) (PLD 3)	endosome and lysosome[17]
	6	Q92597	Protein NDRG1 (Differentiation-related gene 1 protein) (DRG-1) (N-myc downstream-regulated gene 1 protein) (Nickel-specific induction protein Cap43) (Reducing agents and tunicamycin-responsive protein) (RTP) (Rit42)	Endosome[18]
	7	Q96H20	Vacuolar-sorting protein SNF8 (ELL-associated protein of 30 kDa) (ESCRT-II complex subunit VPS22) (hVps22)	Endosome[19]
	8	Q99805	Transmembrane 9 superfamily member 2 (p76)	Endosome[20]
	9	Q9H444	Charged multivesicular body protein 4b (Chromatin-modifying protein 4b) (CHMP4b) (SNF7 homolog associated with Alix 1) (SNF7-2) (hSnf7-2) (Vacuolar protein sorting-associated protein 32-2) (Vps32-2) (hVps32-2)	Endosome[21]
	10	Q9H9H4	Vacuolar protein sorting-associated protein 37B (hVps37B) (ESCRT-I complex subunit VPS37B)	Endosome[13]
Sample 2	1	P49757	Protein numb homolog (h-Numb) (Protein S171)	Endosome[22]
Sample 3	1	O75351	Vacuolar protein sorting-associated protein 4B (EC 3.6.4.6) (Cell migration-inducing gene 1 protein) (Suppressor of	Endosome[23]

			K(+) transport growth defect 1) (Protein SKD1)	
	2	Q96EE3	Nucleoporin SEH1 (GATOR complex protein SEH1) (Nup107-160 subcomplex subunit SEH1) (SEC13-like protein)	---
Sample 4	1	Q15907	Ras-related protein Rab-11B (EC 3.6.5.2) (GTP-binding protein YPT3)	Endosome[24]
	2	Q5JSH3	WD repeat-containing protein 44 (Rabphilin-11)	Endosome[25]

Note: the location displayed in the above table may not be the only location of the corresponding protein as a protein can have more than 1 location[26]. ---, no suitable reference.

8. UPLC-Chromatogram and MS-Spectrum

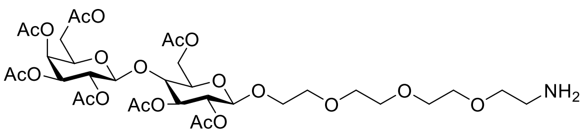

S24 Chemical Formula: $C_{34}H_{53}NO_{21}$
Exact Mass: 811.31
Molecular Weight: 811.78

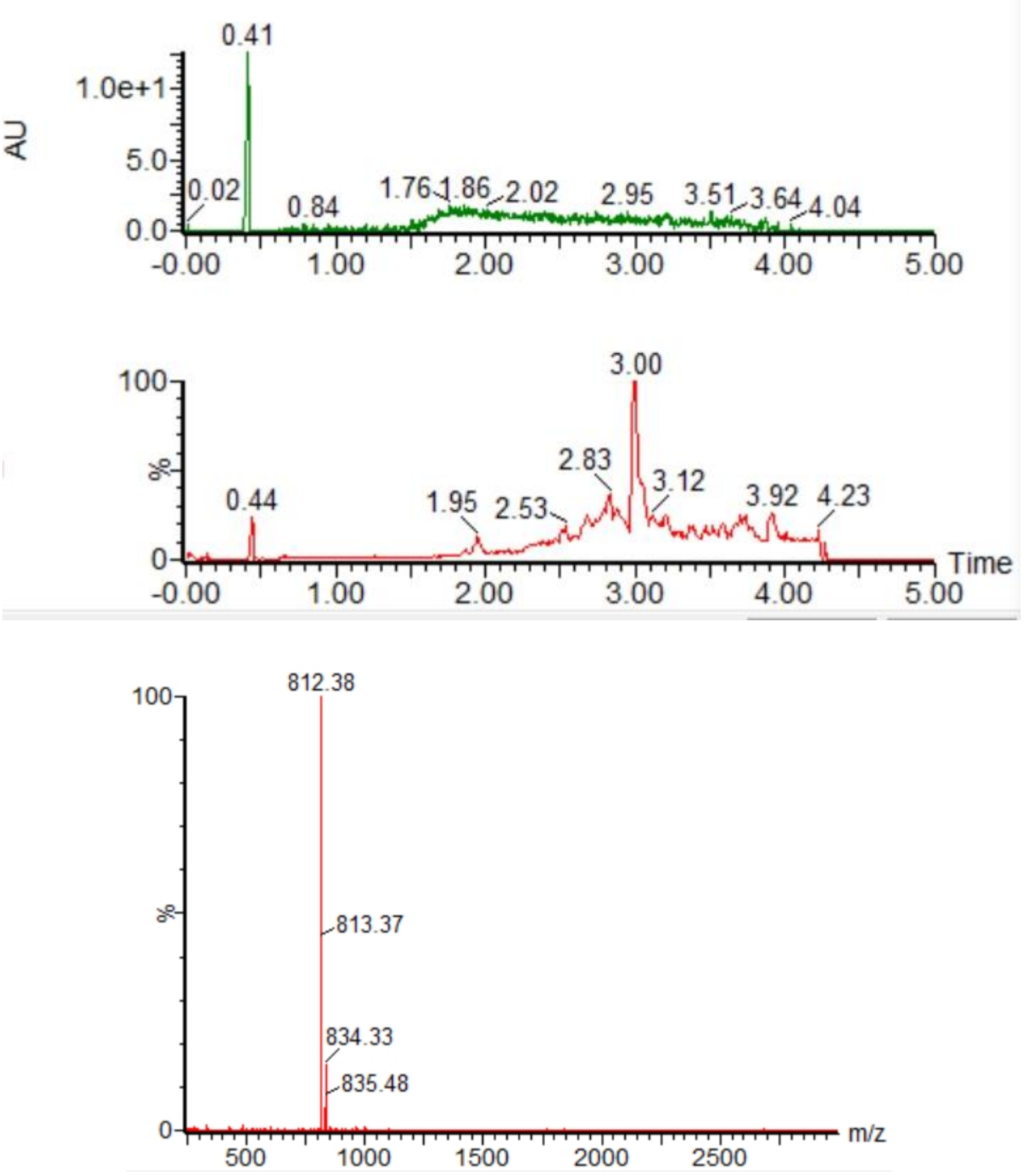

Figure S25. UV trace and corresponding MS trace from LC-MS analysis of compound S24. Gradient: 5-95% ACN/H_2O with 0.1% TFA over 5 min at a flow rate of 0.4 mL/min. ESI-MS calcd. for $C_{34}H_{53}NO_{21}$=811.31; $[M+H]^+$ m/z = 812.31, found 812.38. It should be noted that compound S24 has no UV absorption, therefore the mass trace (middle) of it is given.

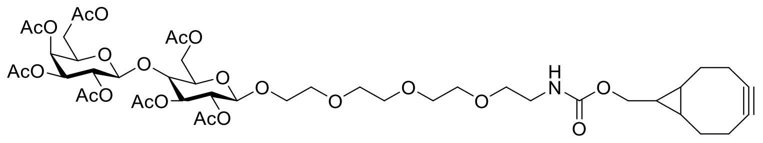

S25 Chemical Formula: $C_{45}H_{65}NO_{23}$
Exact Mass: 987.39
Molecular Weight: 988.00

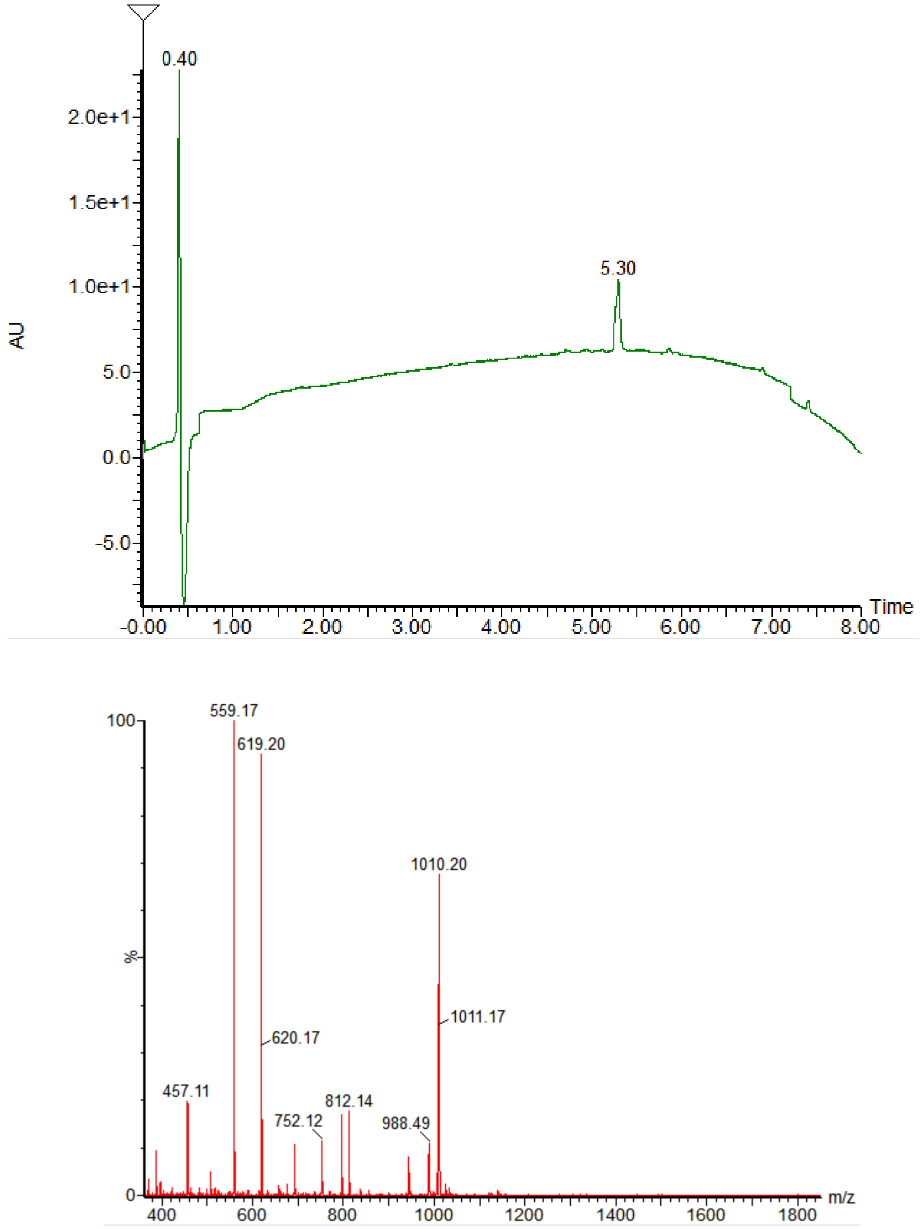

Figure S26. UV trace and corresponding MS trace from LC-MS analysis of the purified compound S25. Gradient: 5-95% ACN/H_2O with 0.1% TFA over 8 min at a flow rate of 0.4 mL/min. ESI-MS calcd. for $C_{45}H_{65}NO_{23}$=987.39; $[M+H]^+$ m/z = 988.39, $[M+Na^+]^+$ m/z = 1010.39, found 988.49, 1010.20.

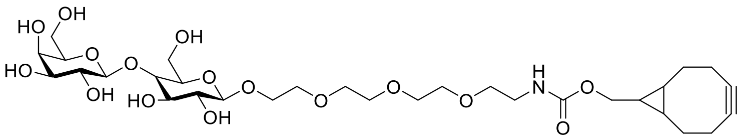

P2 Chemical Formula: $C_{31}H_{51}NO_{16}$
Exact Mass: 693.32
Molecular Weight: 693.74

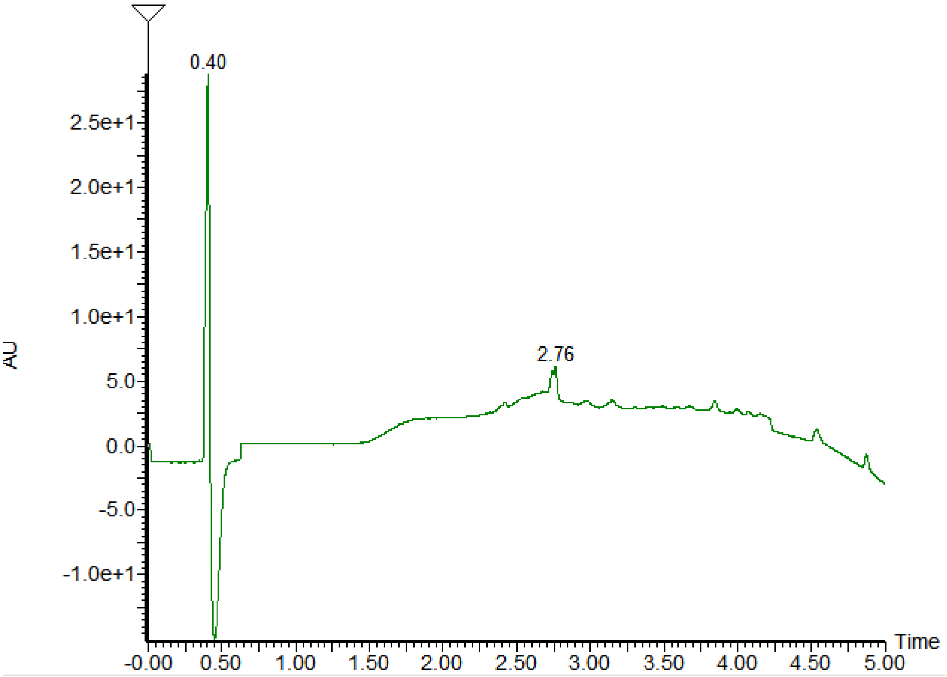

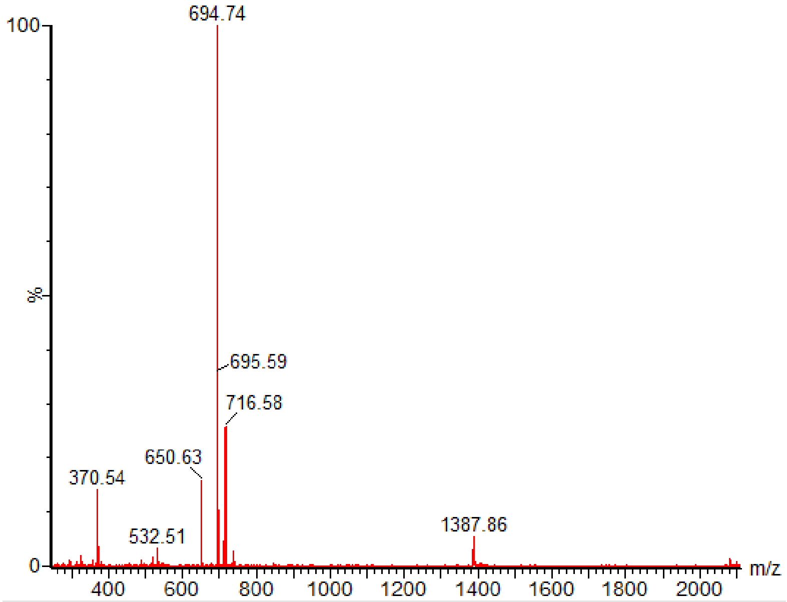

Figure S27. UV trace and corresponding MS trace from LC-MS analysis of the purified P2. Gradient: 5-95% ACN/H_2O with 0.1% TFA over 5 min at a flow rate of 0.4 mL/min. ESI-MS calcd. for $C_{31}H_{51}NO_{16}$=693.32; $[M+H]^+$ m/z = 694.32, $[M+Na^+]^+$ m/z = 716.32, found 694.74, 716.58.

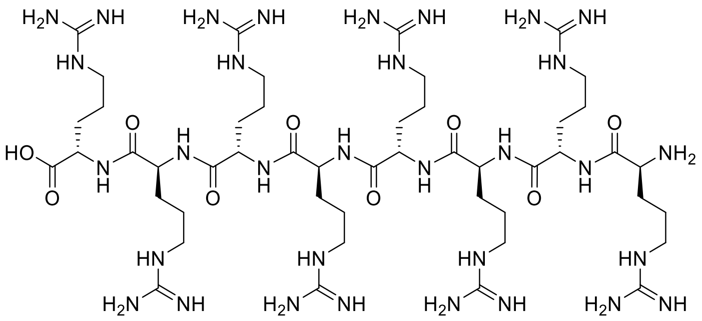

Oligoarginine octapeptide (Arg_8)

Chemical Formula: $C_{48}H_{98}N_{32}O_9$
Exact Mass: 1266.82
Molecular Weight: 1267.53

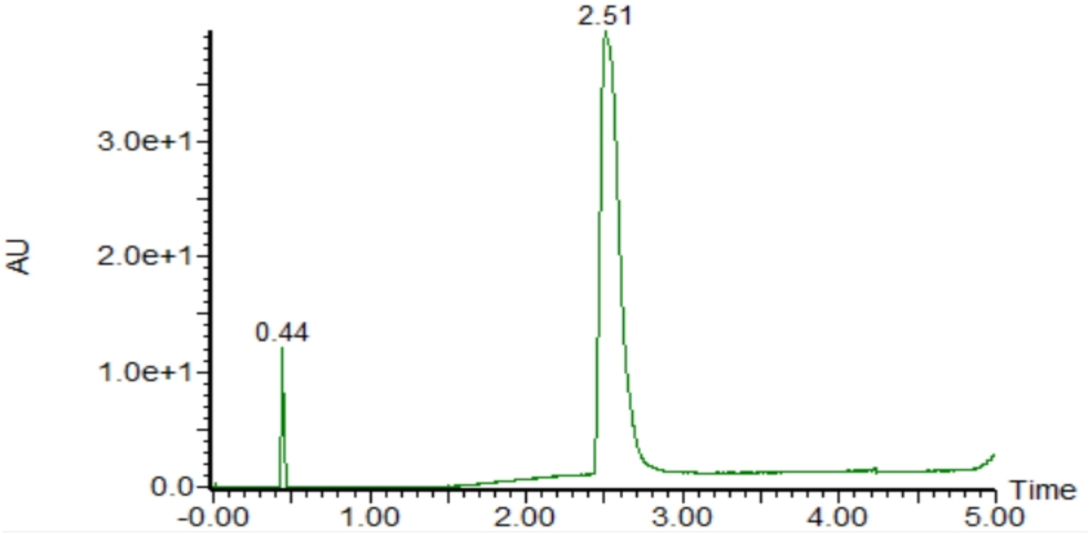

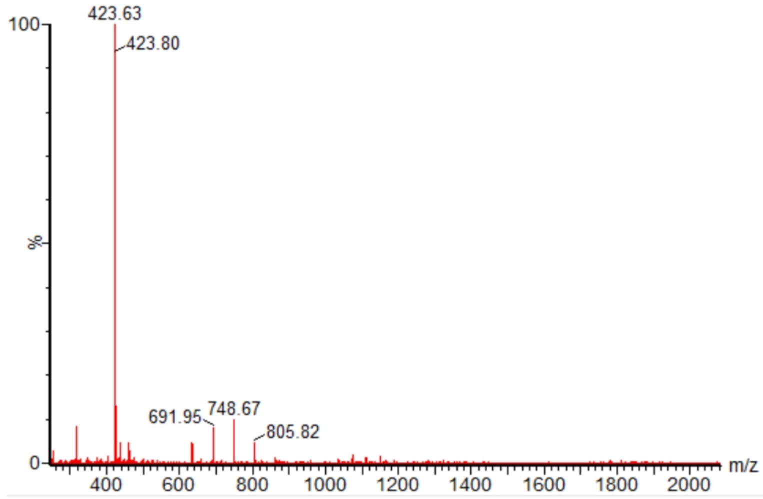

Figure S28. UV trace and corresponding MS trace from LC-MS analysis of the purified $(Arg)_8$. Gradient: 2-30% ACN/H_2O with 0.1% TFA over 5 min at a flow rate of 0.4 mL/min. ESI-MS calcd. for $C_{48}H_{98}N_{32}O_9$ =1266.82; $[M+3H]^{3+}$ m/z = 423.27, found 423.63.

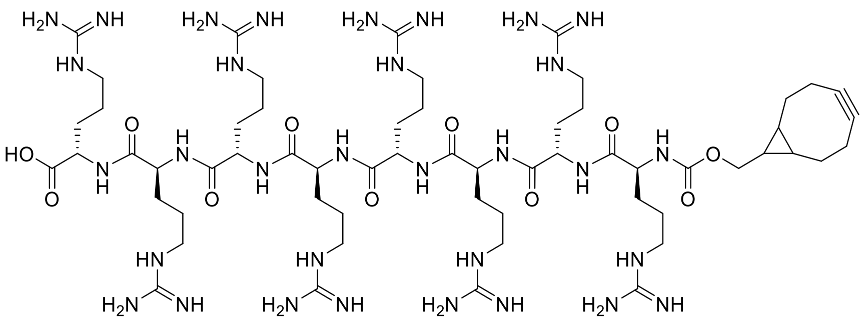

P3 Chemical Formula: $C_{59}H_{110}N_{32}O_{11}$
Exact Mass: 1442.90
Molecular Weight: 1443.74

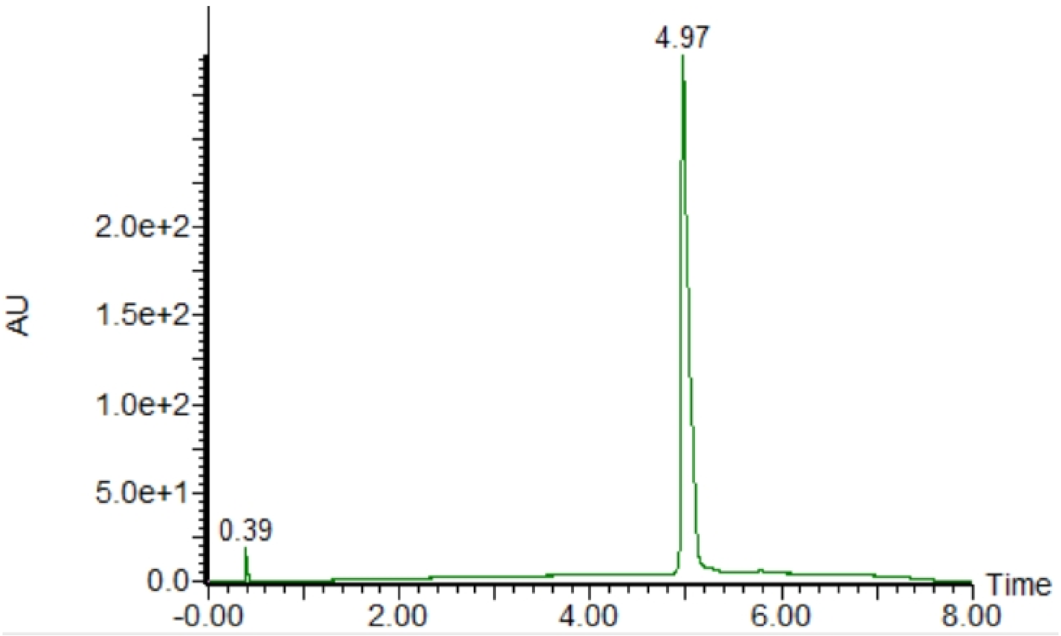

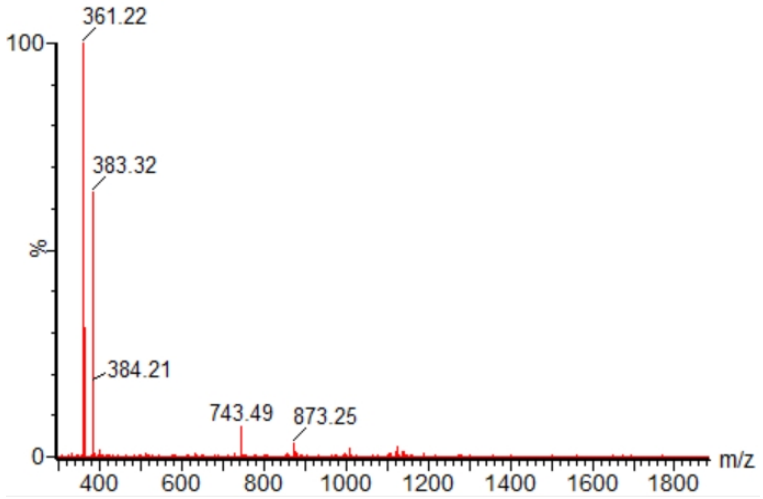

Figure S29. UV trace and corresponding MS trace from LC-MS analysis of P3. Gradient: 2-30% ACN/H_2O with 0.1% TFA over 8 min at a flow rate of 0.4 mL/min. ESI-MS calcd. for $C_{59}H_{110}N_{32}O_{11}$=1442.90; $[M+4H]^{4+}$ m/z = 361.72, found 361.22.

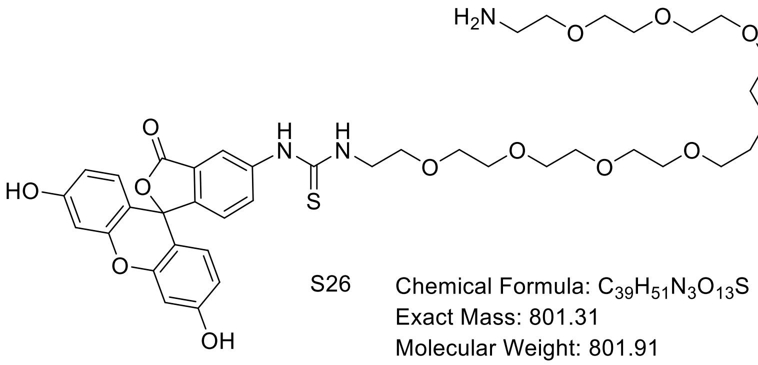

S26 Chemical Formula: $C_{39}H_{51}N_3O_{13}S$
Exact Mass: 801.31
Molecular Weight: 801.91

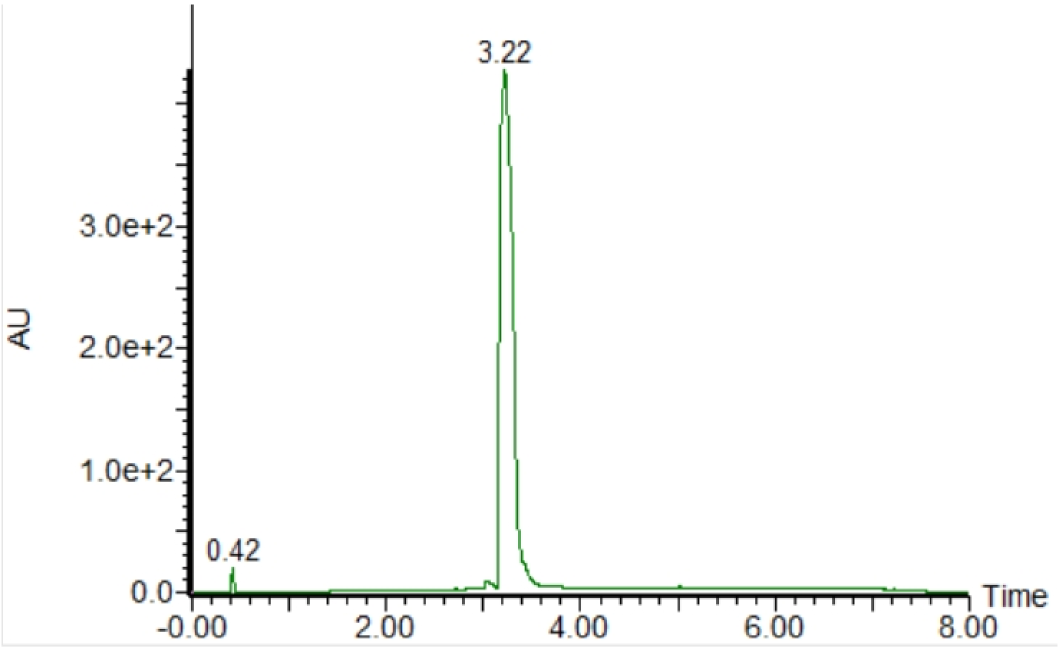

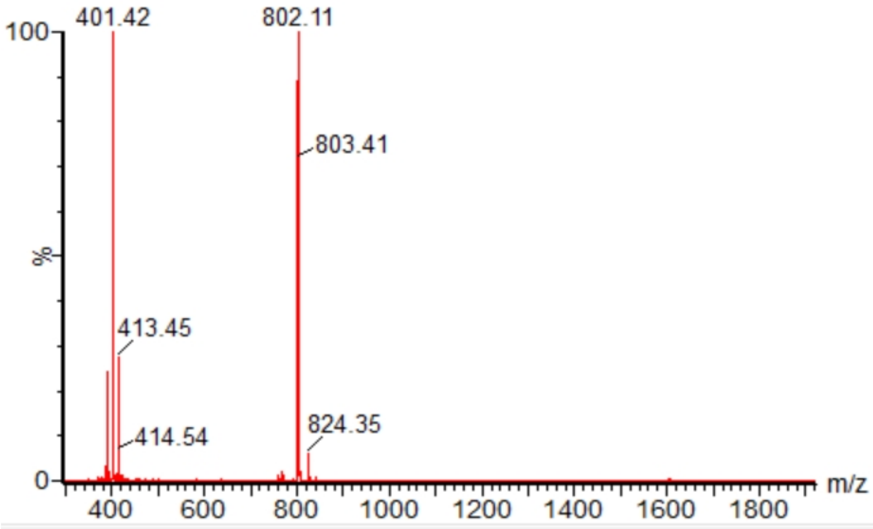

Figure S30. UV trace and corresponding MS trace from LC-MS analysis of compound S26. Gradient: 5-95% ACN/H_2O with 0.1% TFA over 8 min at a flow rate of 0.4 mL/min. ESI-MS calcd. for $C_{39}H_{51}N_3O_{13}S$ =801.31; $[M+H]^+$ m/z = 802.31, $[M+2H]^{2+}$ m/z = 401.65, found 802.11, 401.42.

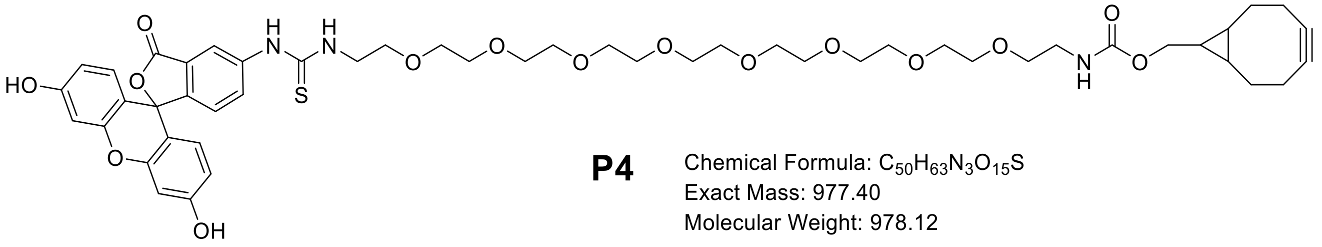

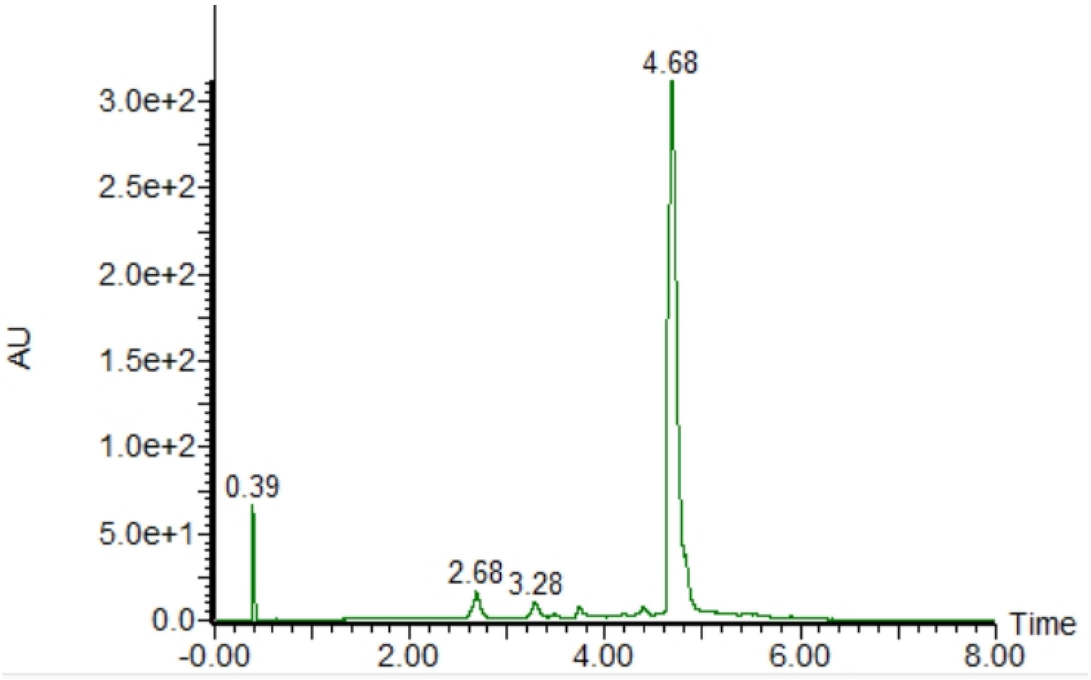

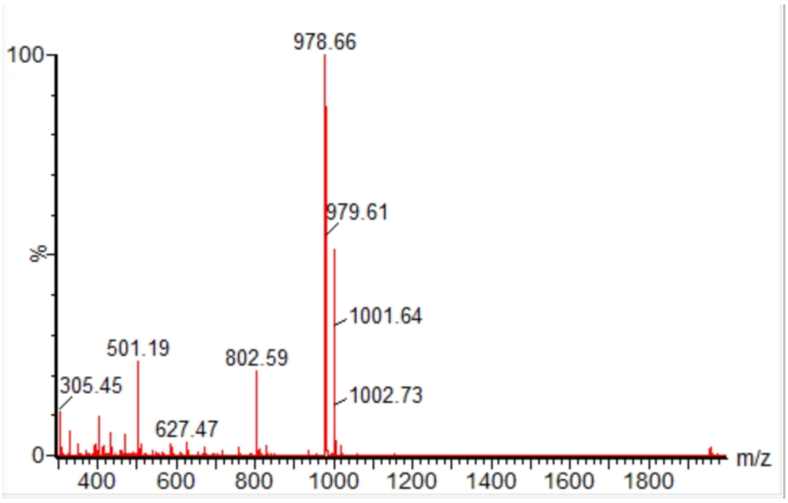

Figure S31. UV trace and corresponding MS trace from LC-MS analysis of P4. Gradient: 5-95% ACN/H_2O with 0.1% TFA over 8 min at a flow rate of 0.4 mL/min. ESI-MS calcd. for $C_{50}H_{63}N_3O_{15}S$ =977.40; $[M+H]^+$ m/z = 978.40, found 978.66.

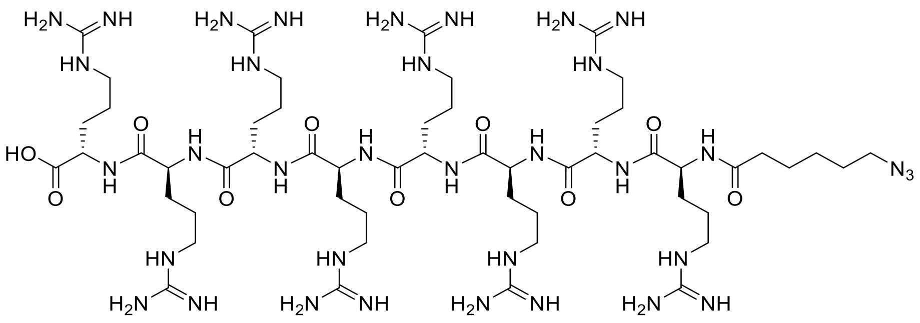

1 ((Arg)$_8$-N$_3$)

Chemical Formula: $C_{54}H_{107}N_{35}O_{10}$
Exact Mass: 1405.89
Molecular Weight: 1406.69

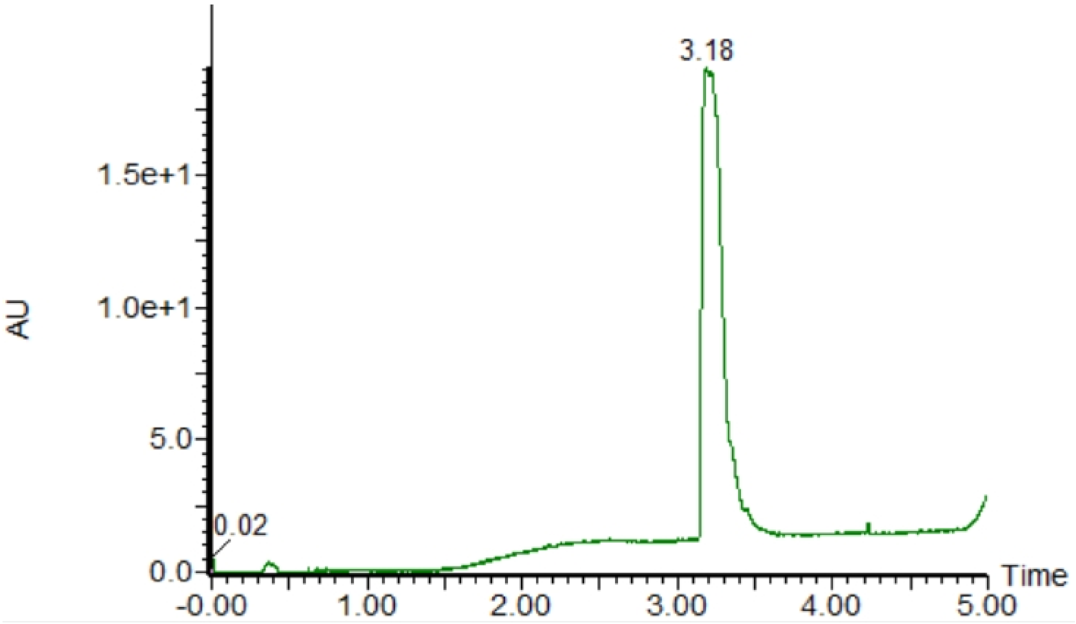

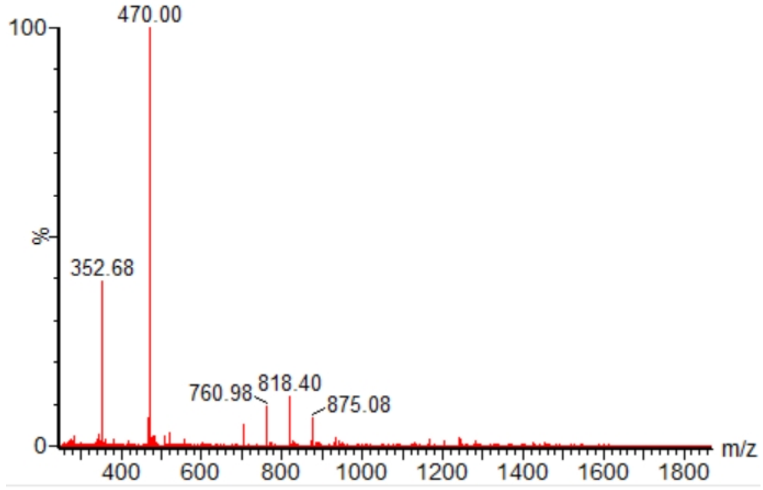

Figure S32. UV trace and corresponding MS trace from LC-MS analysis of **1**. Gradient: 2-30% ACN/H_2O with 0.1% TFA over 5 min at a flow rate of 0.4 mL/min. ESI-MS calcd. for $C_{54}H_{107}N_{35}O_{10}$=1405.89; $[M+3H]^{3+}$ m/z = 469.63, $[M+4H]^{4+}$ m/z = 352.47, found 470.00, 352.68.

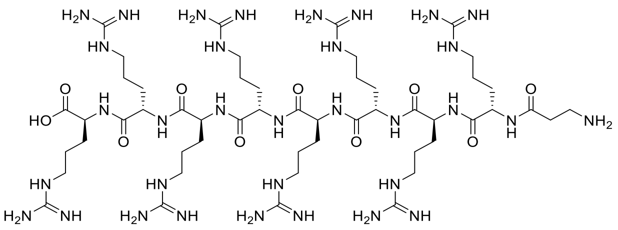

Arg$_8$-βAla Chemical Formula: $C_{51}H_{103}N_{33}O_{10}$
Exact Mass: 1337.86
Molecular Weight: 1338.61

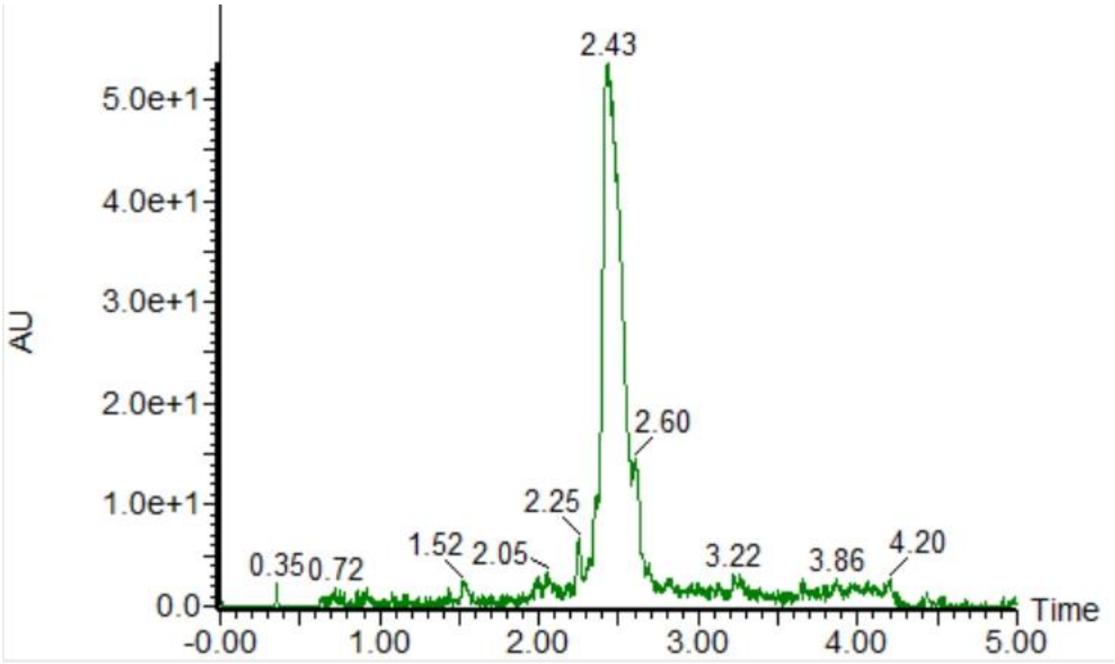

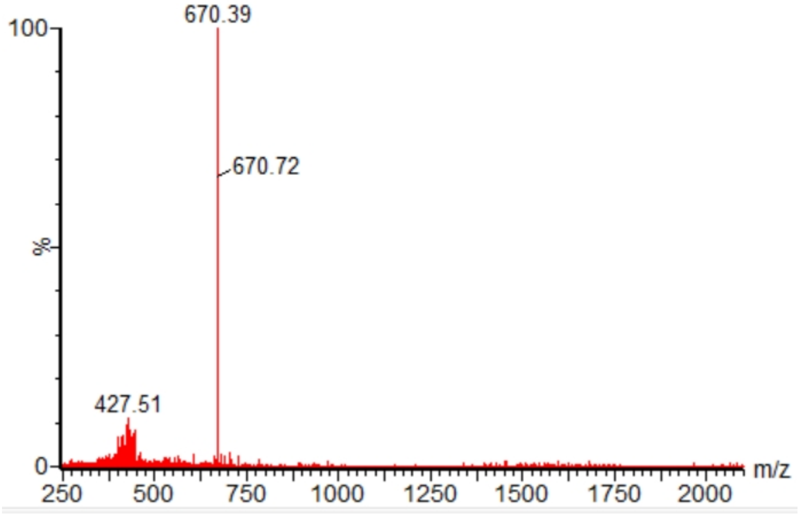

Figure S33. UV trace and corresponding MS trace from LC-MS analysis of (Arg)$_8$-βAla. Gradient: 2-30% ACN/H_2O with 0.1% TFA over 5 min at a flow rate of 0.4 mL/min. ESI-MS calcd. for $C_{51}H_{103}N_{33}O_{10}$=1337.86; $[M+2H]^{2+}$ m/z = 669.93, found 670.39.

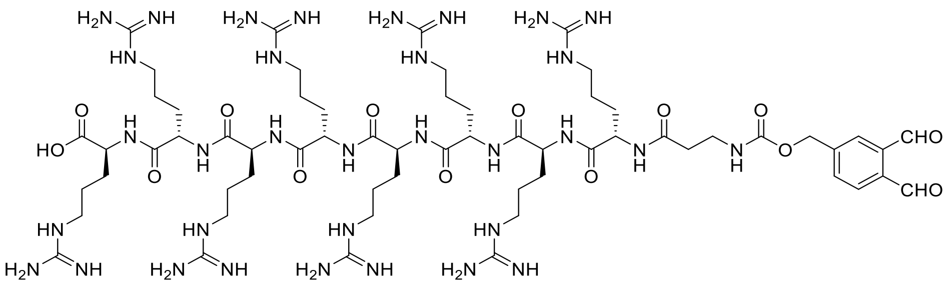

2 (Arg$_8$-OPA)

Chemical Formula: $C_{61}H_{109}N_{33}O_{14}$
Exact Mass: 1527.88
Molecular Weight: 1528.76

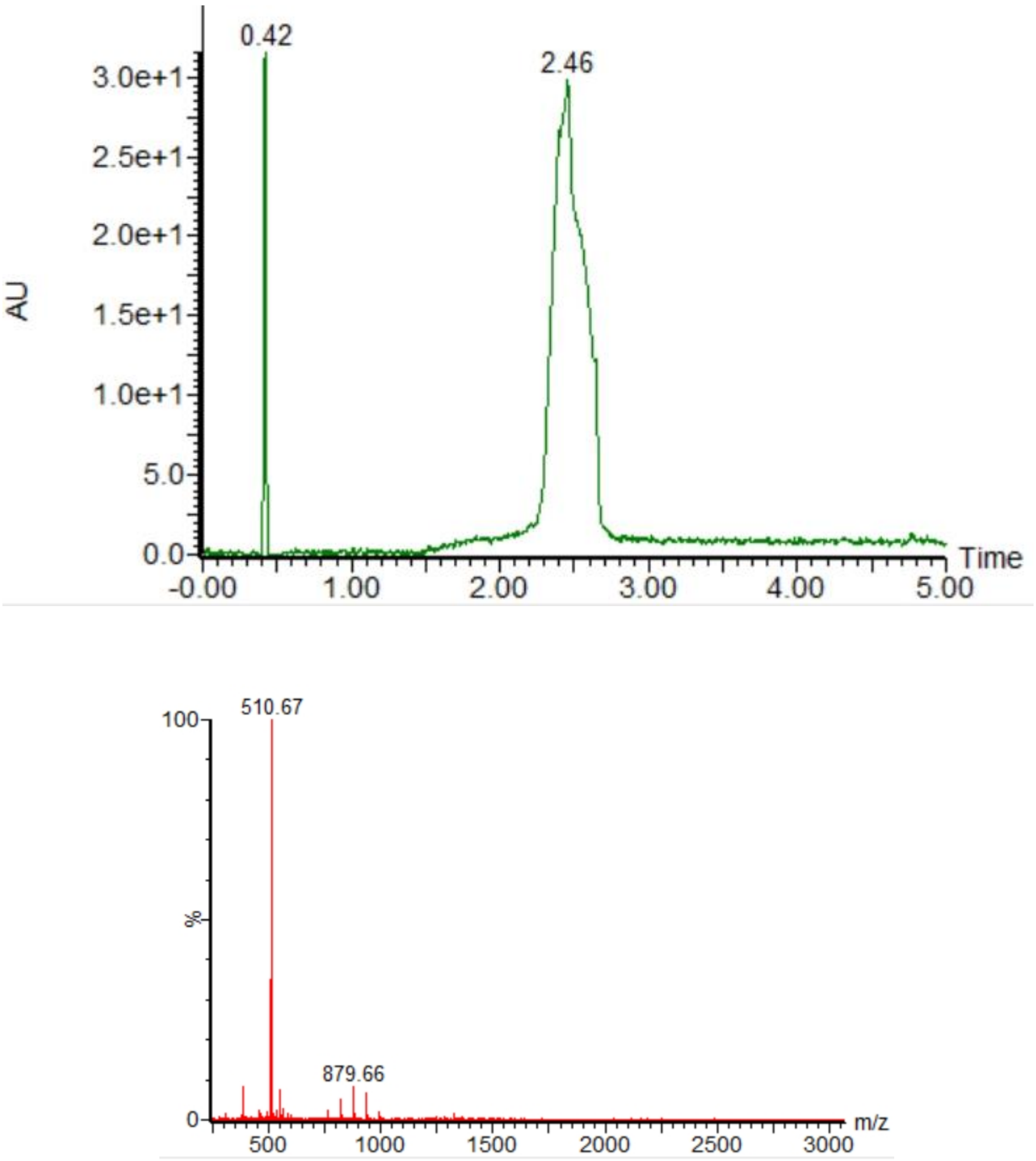

Figure S34. UV trace and corresponding MS trace from LC-MS analysis of purified **2**. Gradient: 2-30% ACN/H_2O with 0.1% TFA over 5 min at a flow rate of 0.4 mL/min. ESI-MS calcd. for $C_{61}H_{109}N_{33}O_{14}$=1527.88; $[M+3H]^{3+}$ m/z = 510.29, found 510.67.

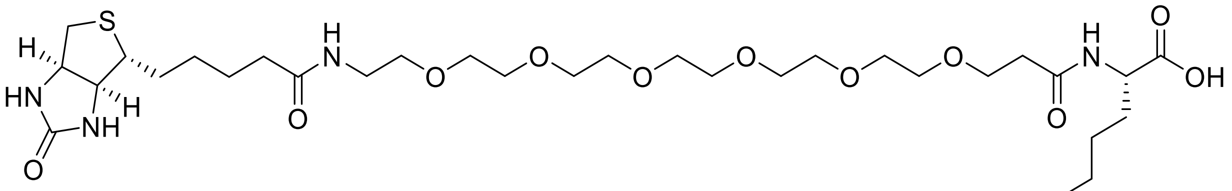

S27 Chemical Formula: $C_{31}H_{57}N_5O_{11}S$
Exact Mass: 707.38
Molecular Weight: 707.88

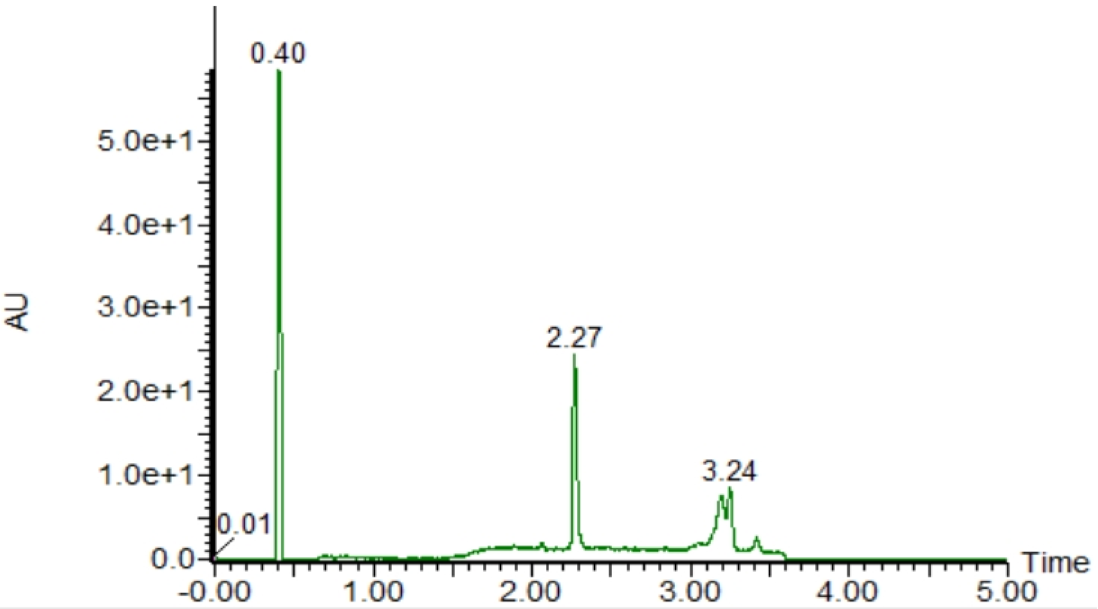

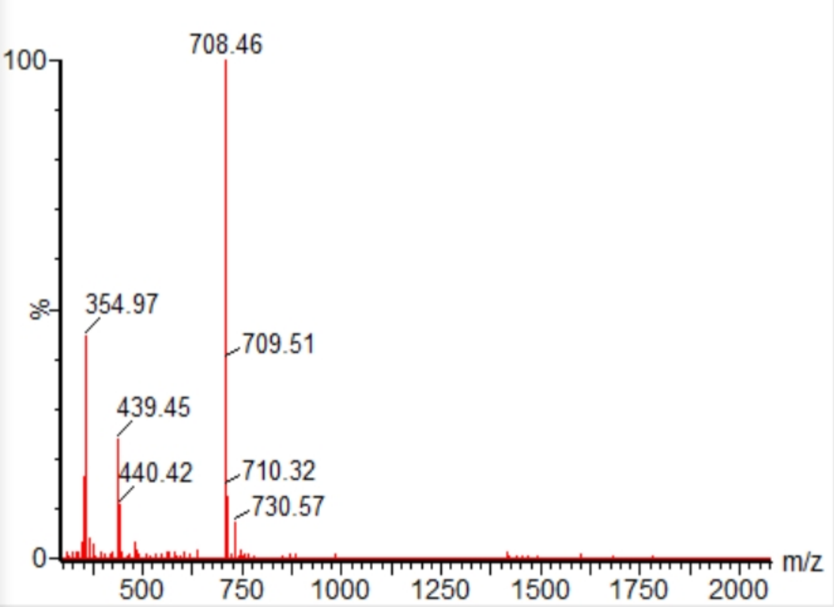

Figure S35. UV trace and corresponding MS trace from LC-MS analysis of compound S27. Gradient: 5-95% ACN/H_2O with 0.1% TFA over 5 min at a flow rate of 0.4 mL/min. ESI-MS calcd. for $C_{31}H_{57}N_5O_{11}S$ =707.38; $[M+H]^+$ m/z = 708.38, found 708.46.

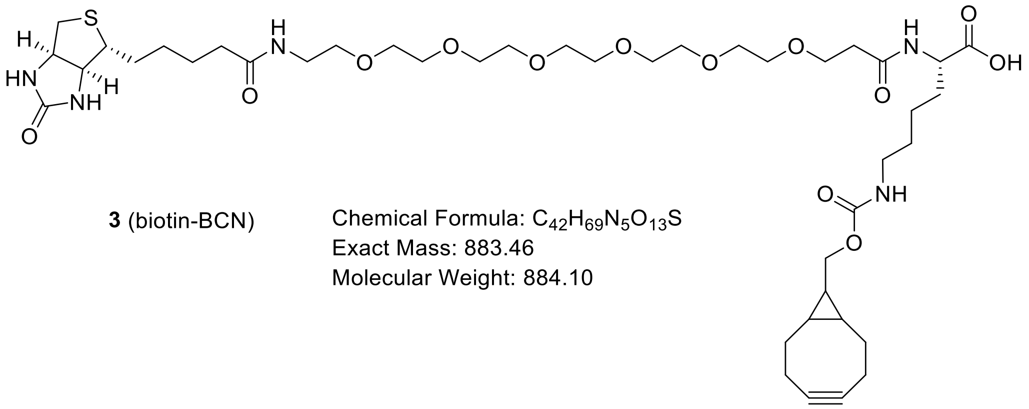

3 (biotin-BCN)

Chemical Formula: $C_{42}H_{69}N_5O_{13}S$
Exact Mass: 883.46
Molecular Weight: 884.10

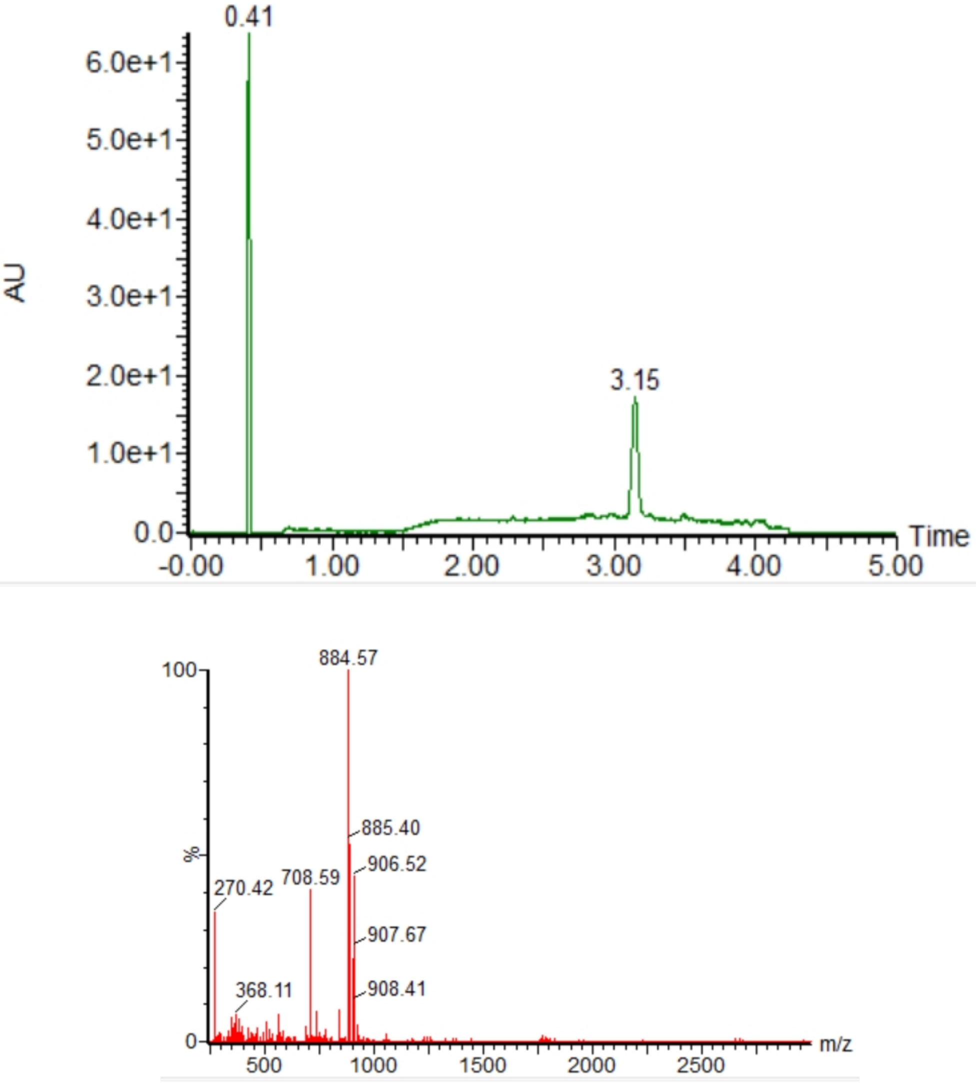

Figure S36. UV trace and corresponding MS trace from LC-MS analysis of the purified **3**. Gradient: 5-95% ACN/H_2O with 0.1% TFA over 5 min at a flow rate of 0.4 mL/min. ESI-MS calcd. for $C_{42}H_{69}N_5O_{13}S$ =883.46; $[M+H]^+$ m/z = 884.46, found 884.57.

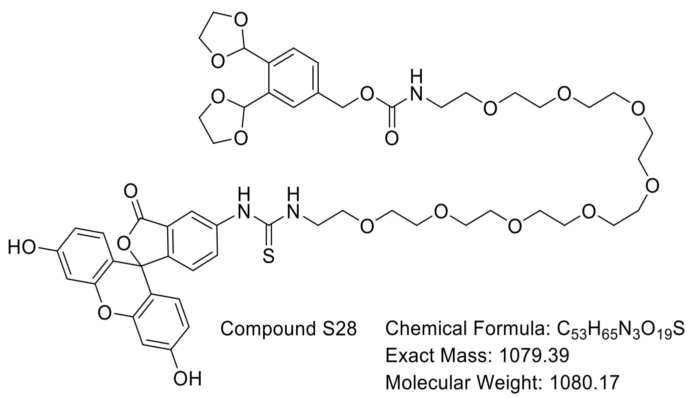

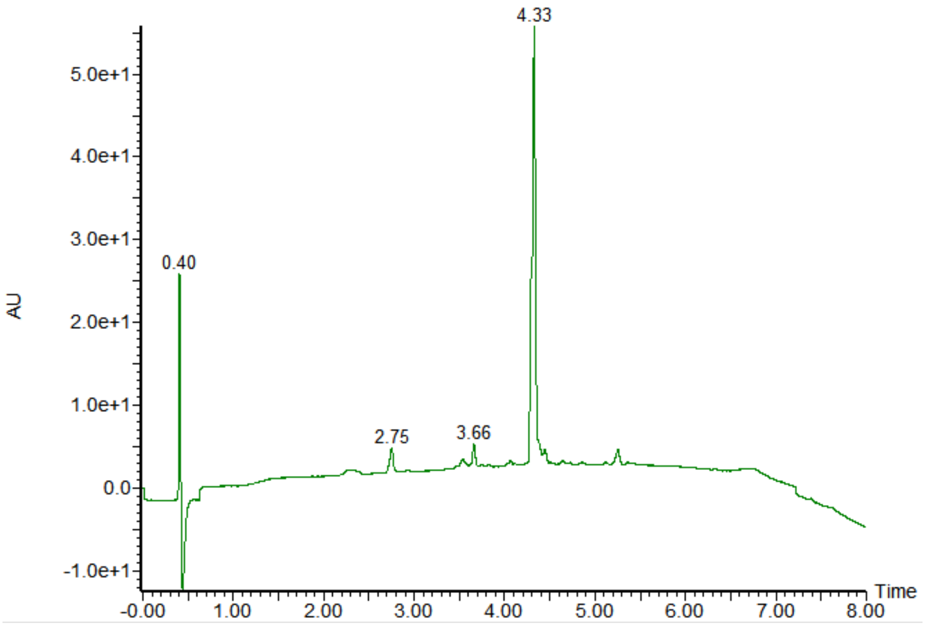

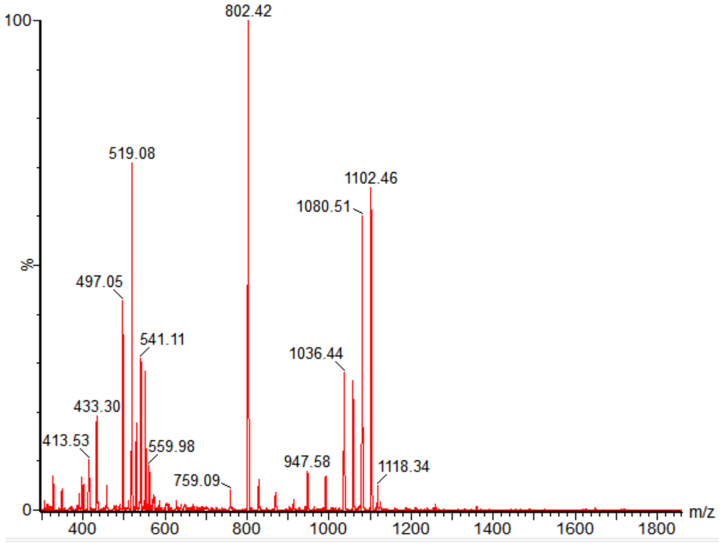

Figure S37. UV trace and corresponding MS trace from LC-MS analysis of the Compound S28. Gradient: 5-95% ACN/H_2O with 0.1% TFA over 8 min at a flow rate of 0.4 mL/min. ESI-MS calcd. for $C_{53}H_{65}N_3O_{19}S$ =1079.39; $[M+H]^+$ m/z = 1080.39, $[M+Na^+]^+$ m/z = 1102.39, found 1080.51, 1102.46.

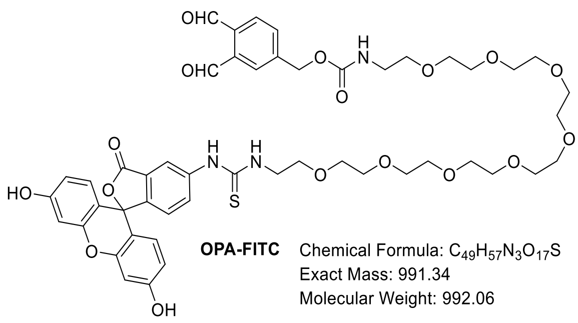

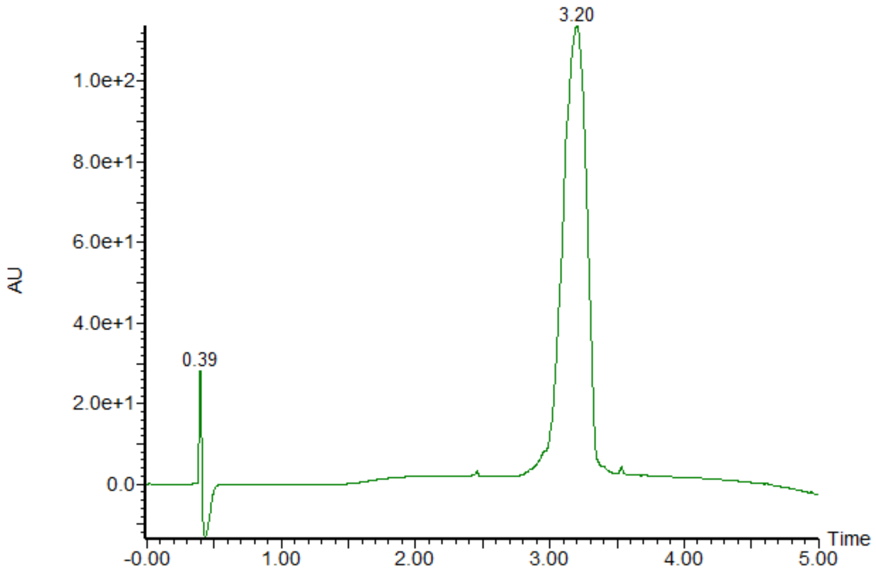

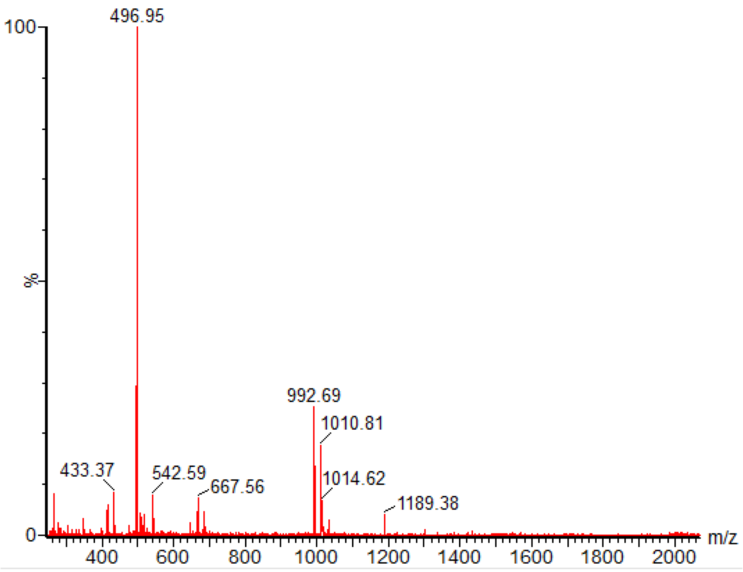

Figure S38. UV trace and corresponding MS trace from LC-MS analysis of the purified OPA-FITC. Gradient: 5-95% ACN/H_2O with 0.1% TFA over 5 min at a flow rate of 0.4 mL/min. ESI-MS calcd. for $C_{49}H_{57}N_3O_{17}S$ = 991.34; $[M+H]^+$ m/z = 992.34, $[M+2H]^{2+}$ m/z = 496.67, $[M+Na^+]^+$ m/z = 1014.34 found 992.69, 496.95, 1014.62.

9. ¹H- and ¹³C-NMR Spectra of Small Molecular

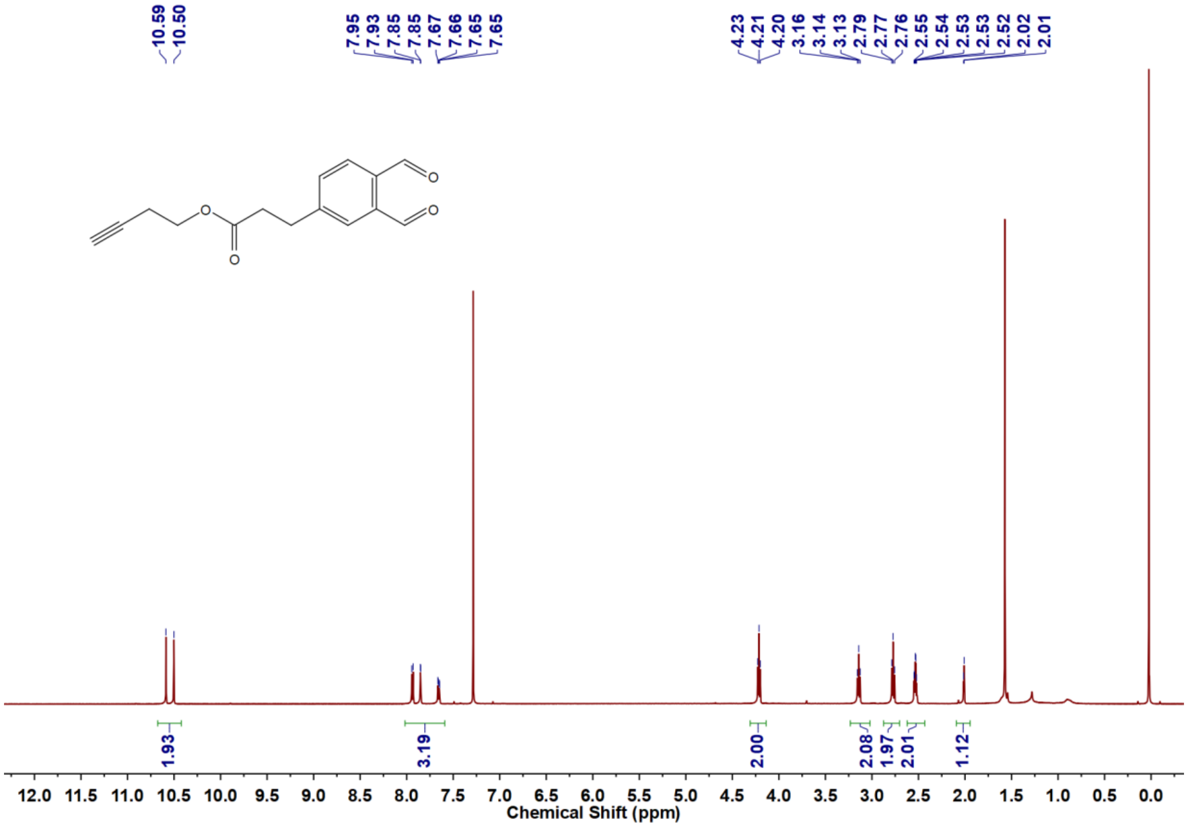

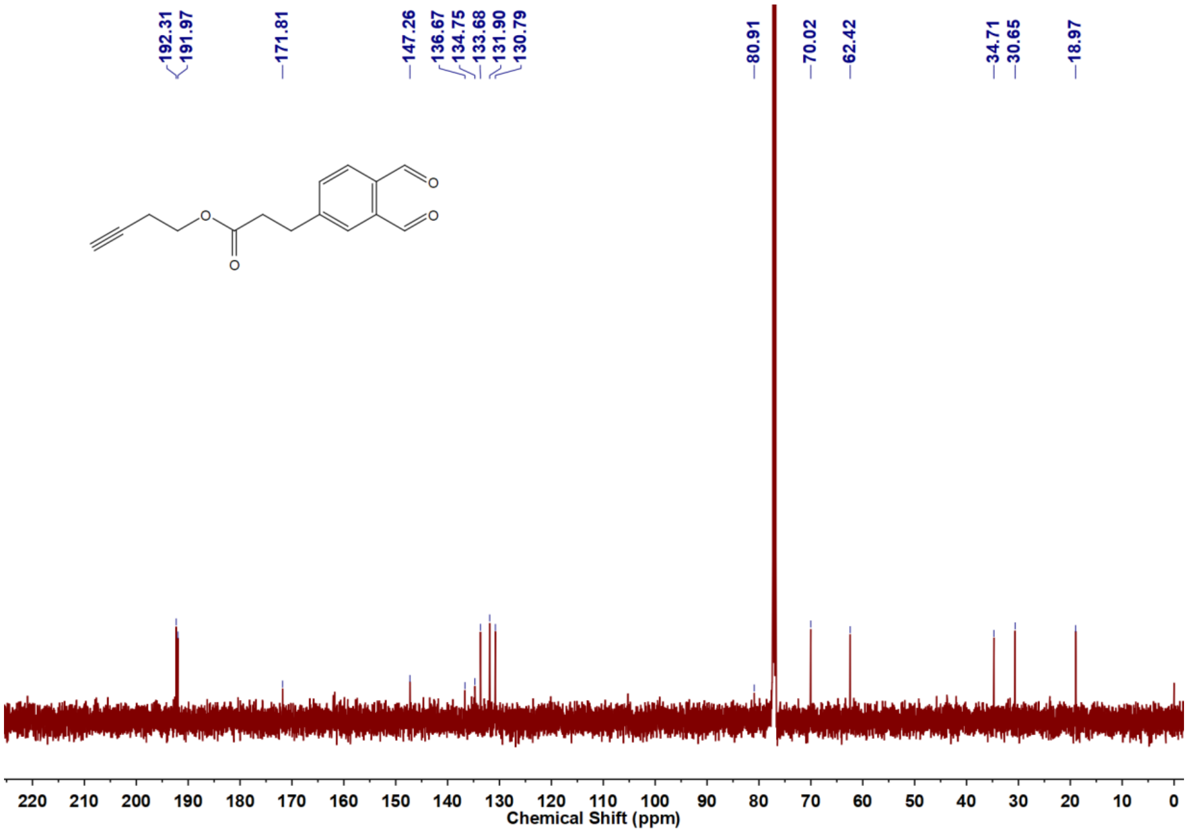

Figure S39. ¹H and ¹³C NMR spectra of M1 in $CDCl_3$.

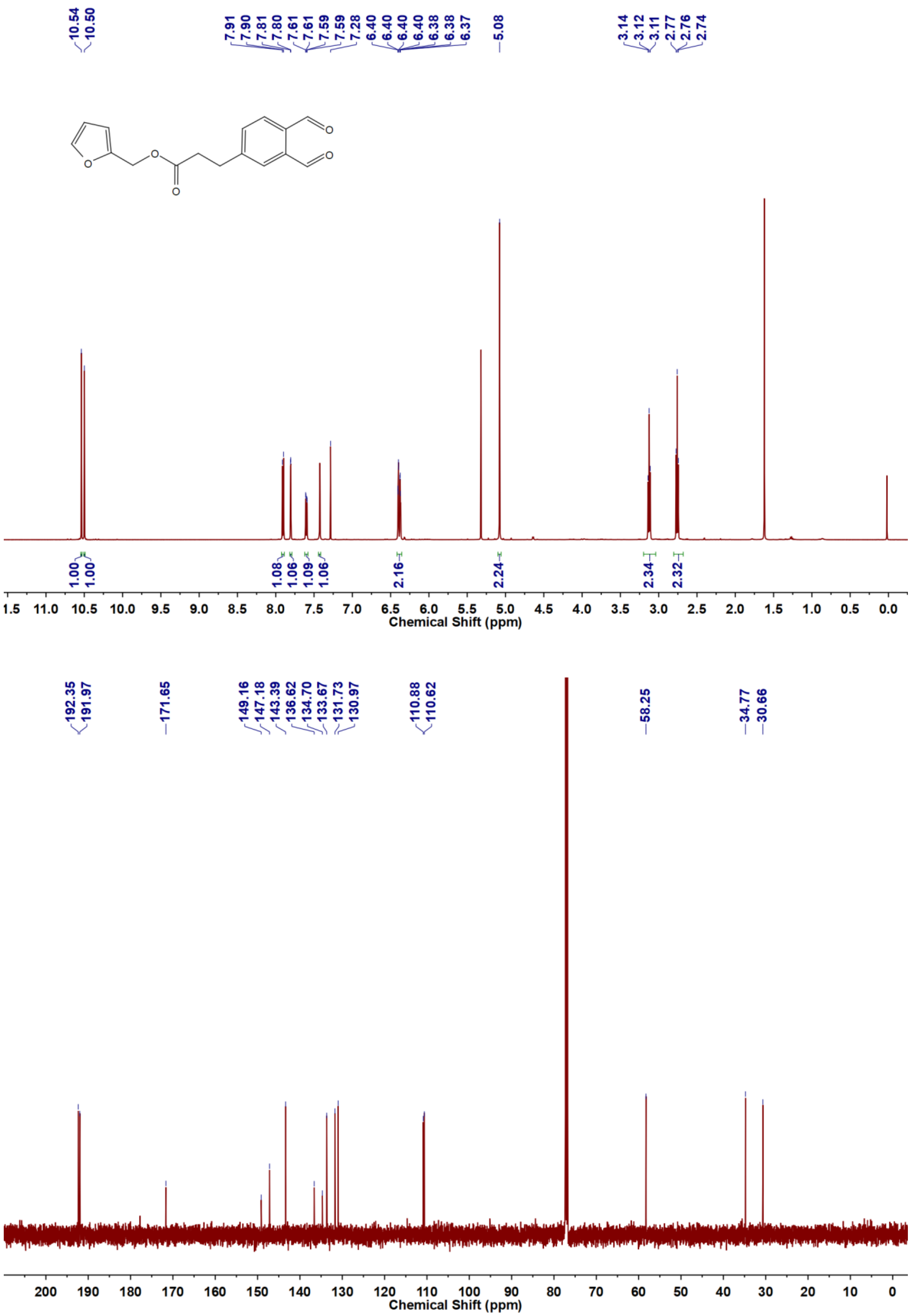

Figure S40. ^{1}H and ^{13}C NMR spectra of M2 in $CDCl_3$.

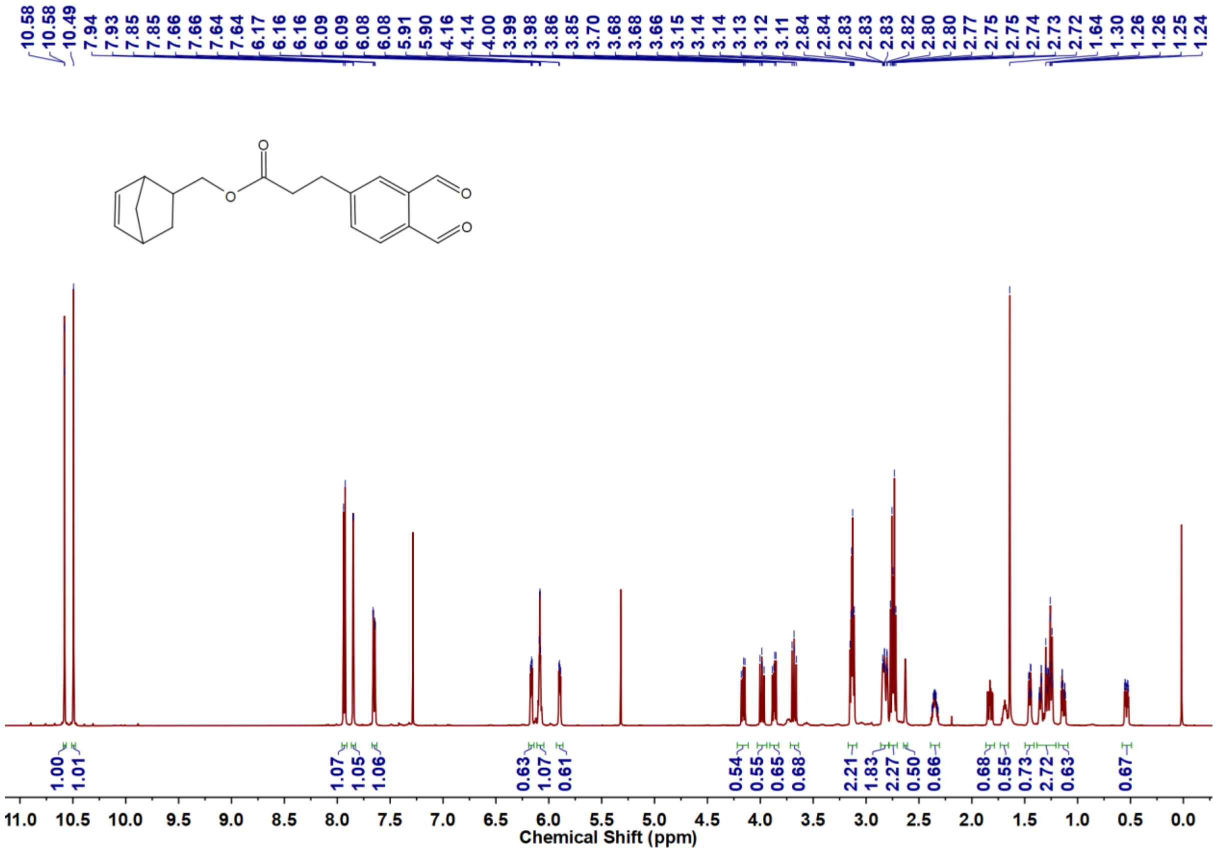

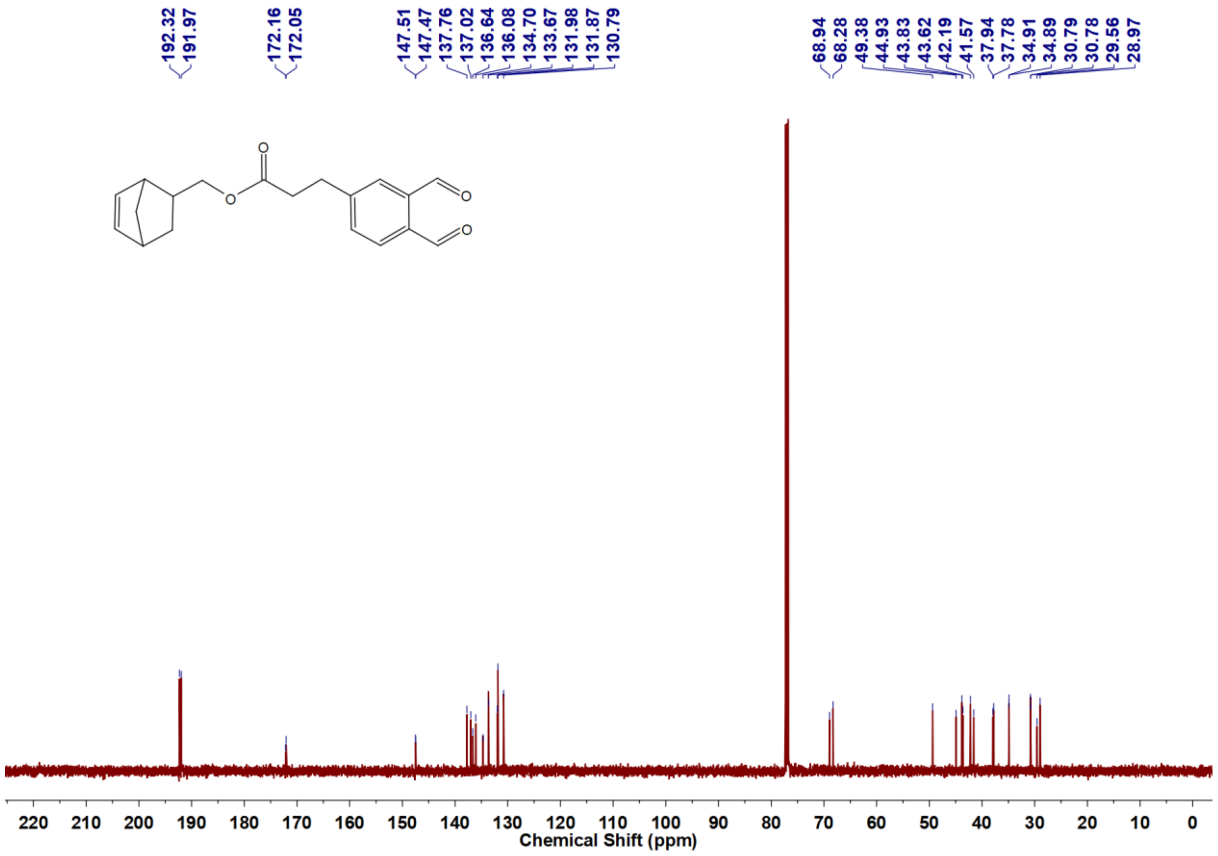

Figure S41. ^{1}H and ^{13}C NMR spectra of M3 in $CDCl_3$.

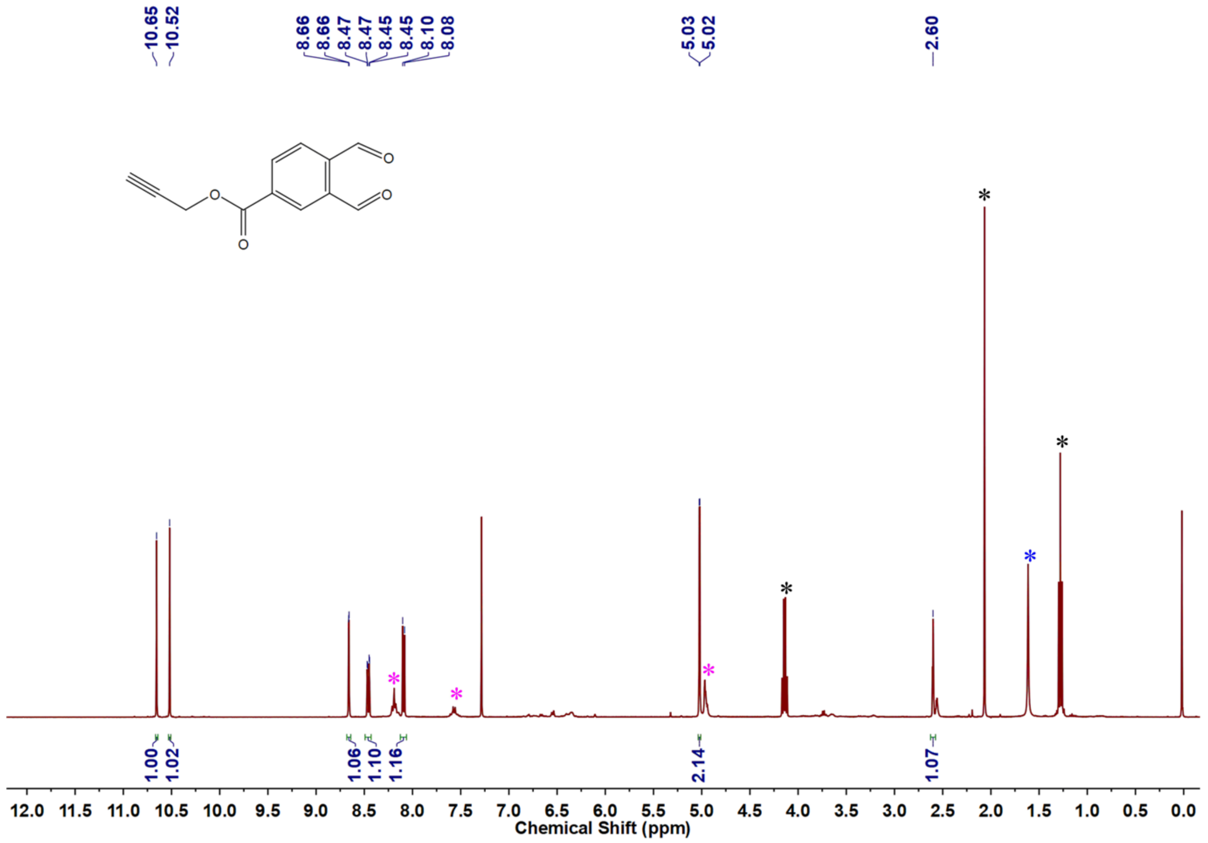

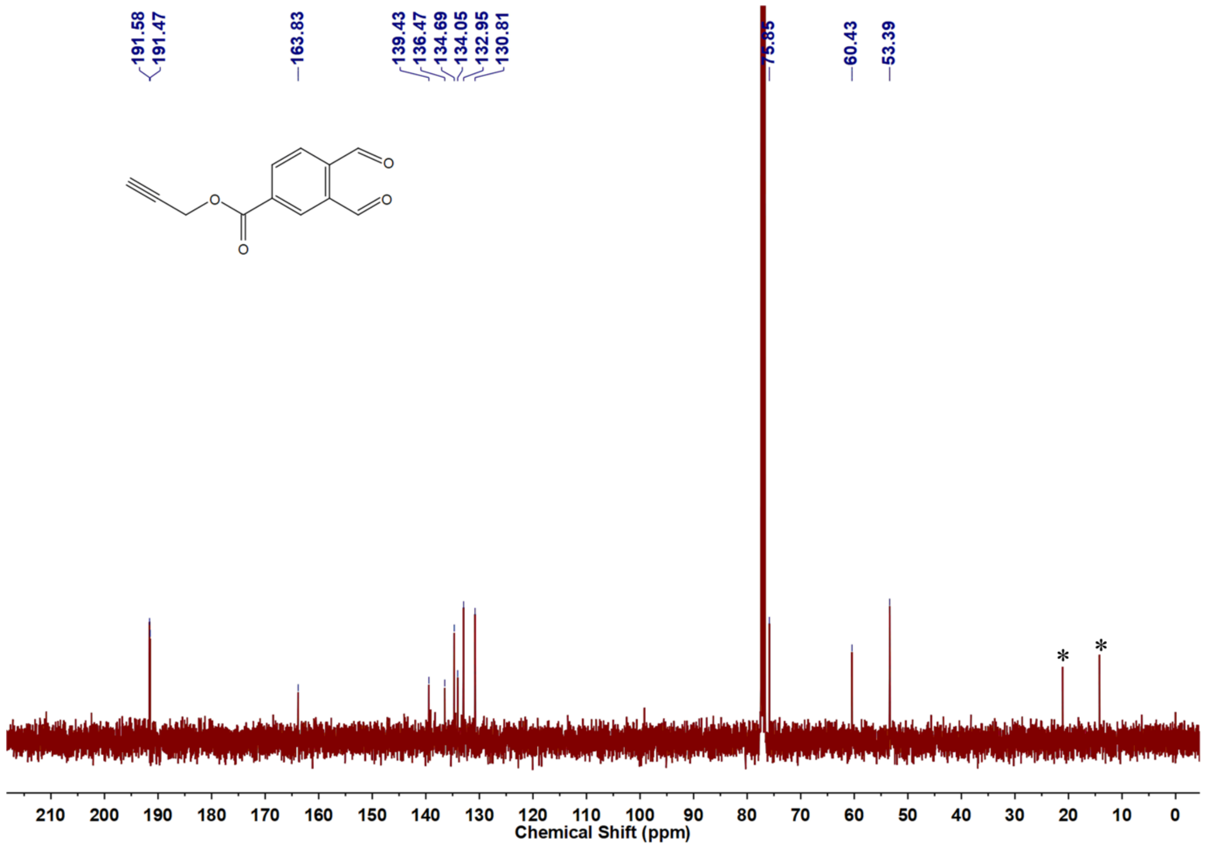

Figure S42. ^{1}H and ^{13}C NMR spectra of M4 in $CDCl_3$. The asterisks represent ethyl acetate or some unknown impurities.

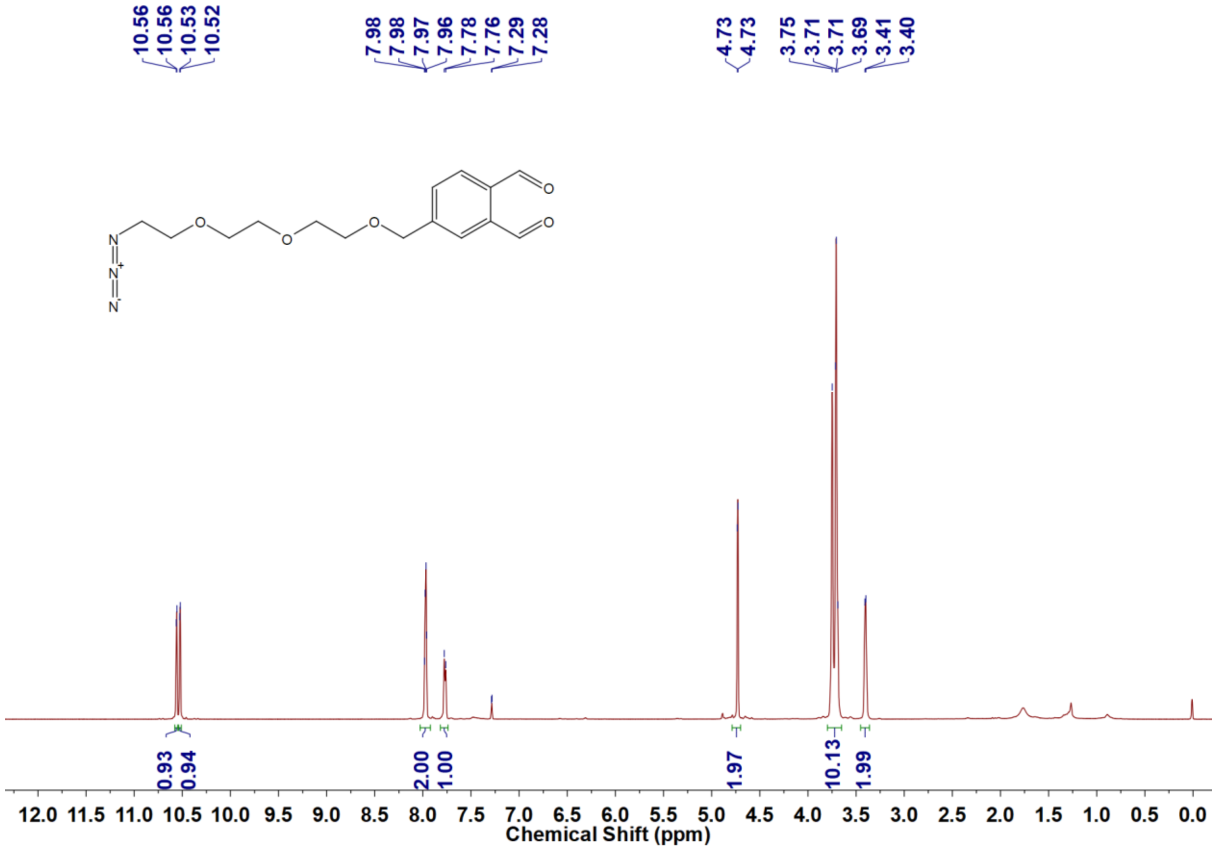

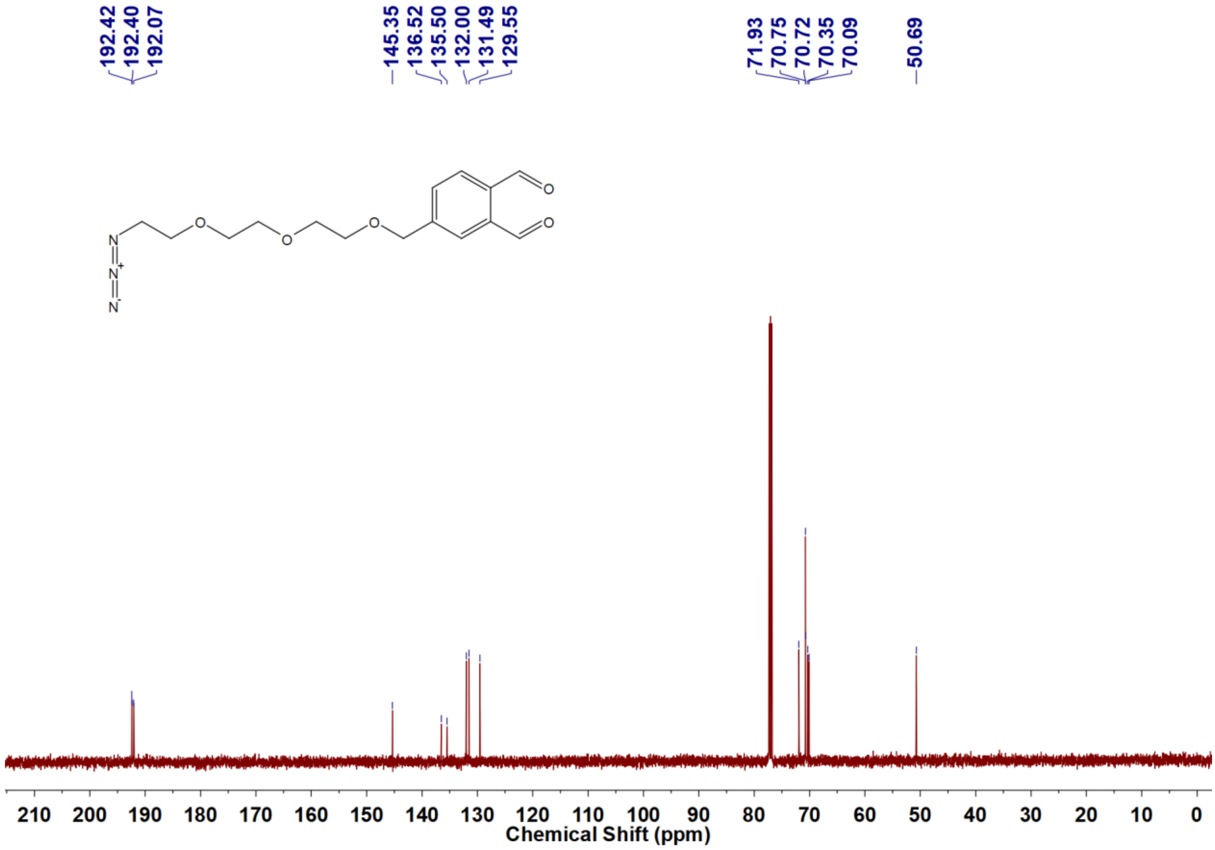

Figure S43. ^{1}H and ^{13}C NMR spectra of M5 in $CDCl_3$.

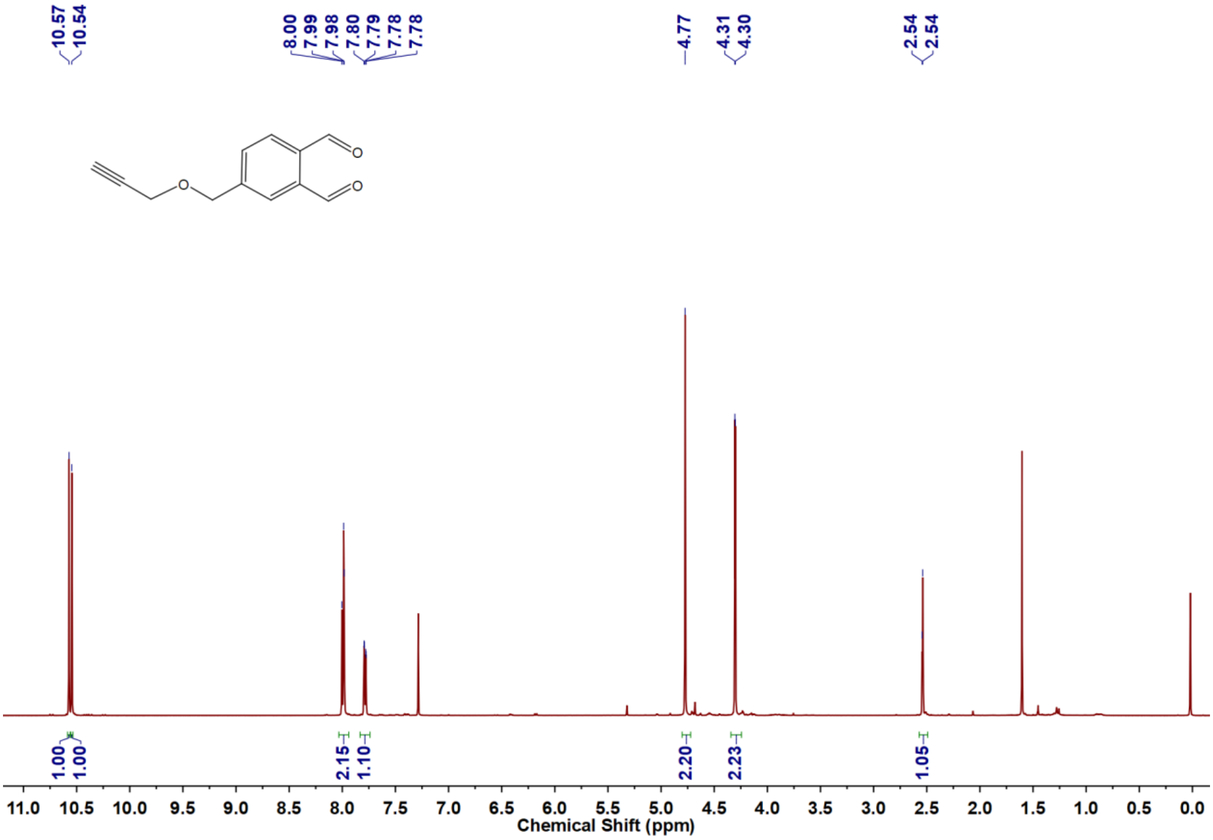

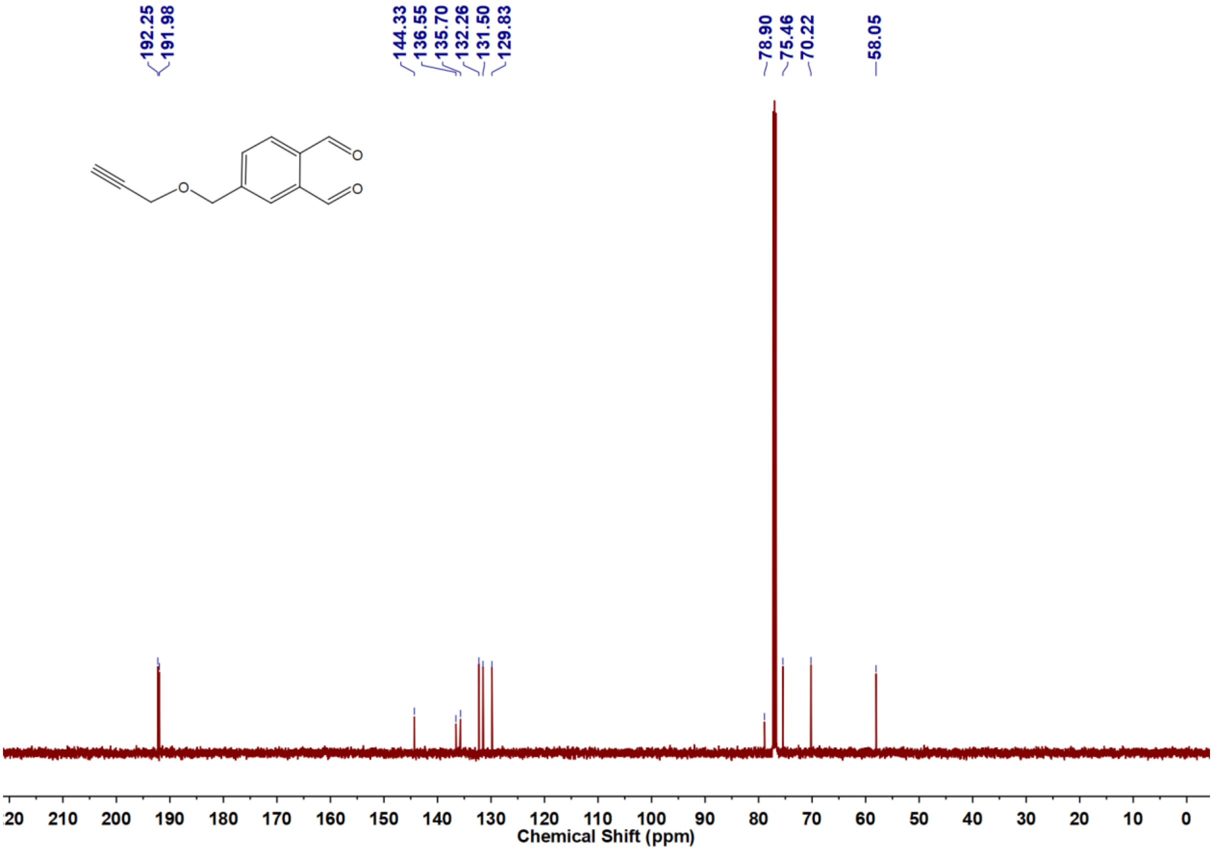

Figure S44. ^{1}H and ^{13}C NMR spectra of M6 in $CDCl_3$.

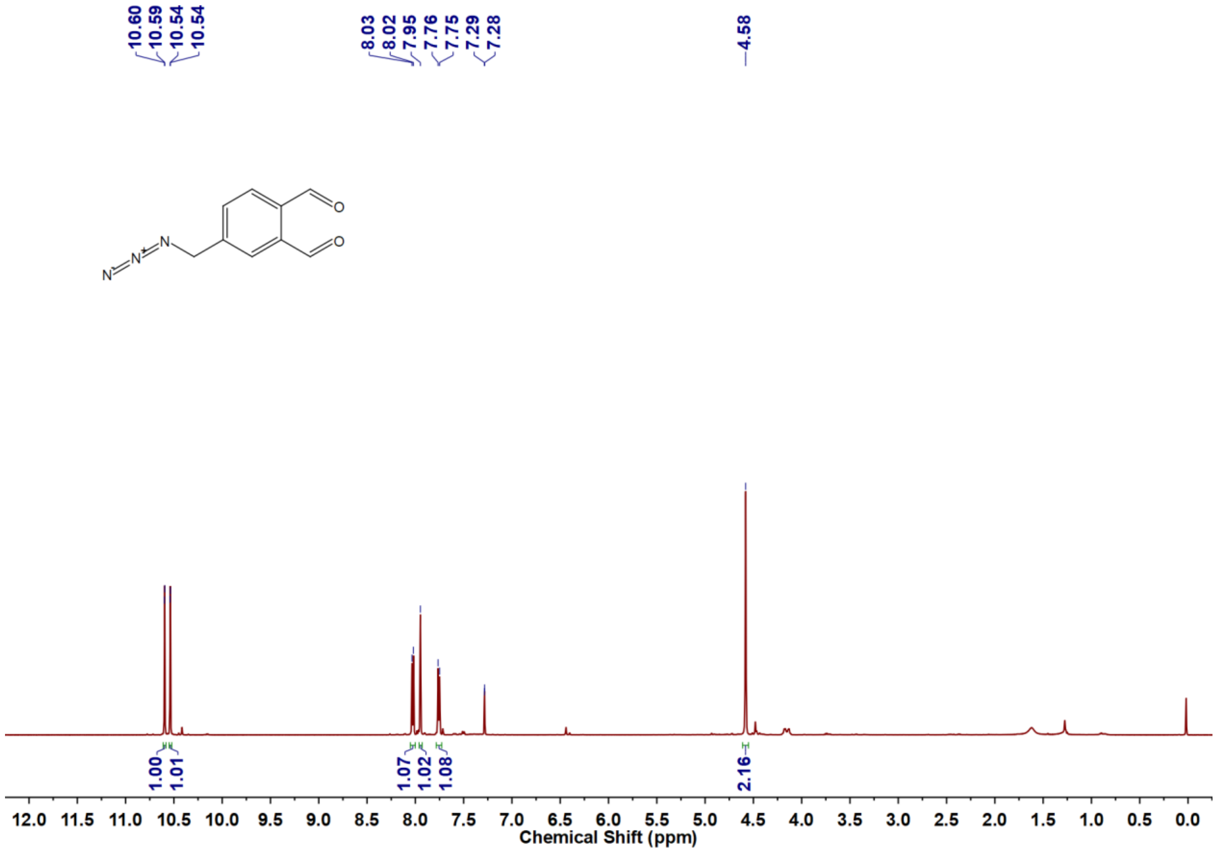

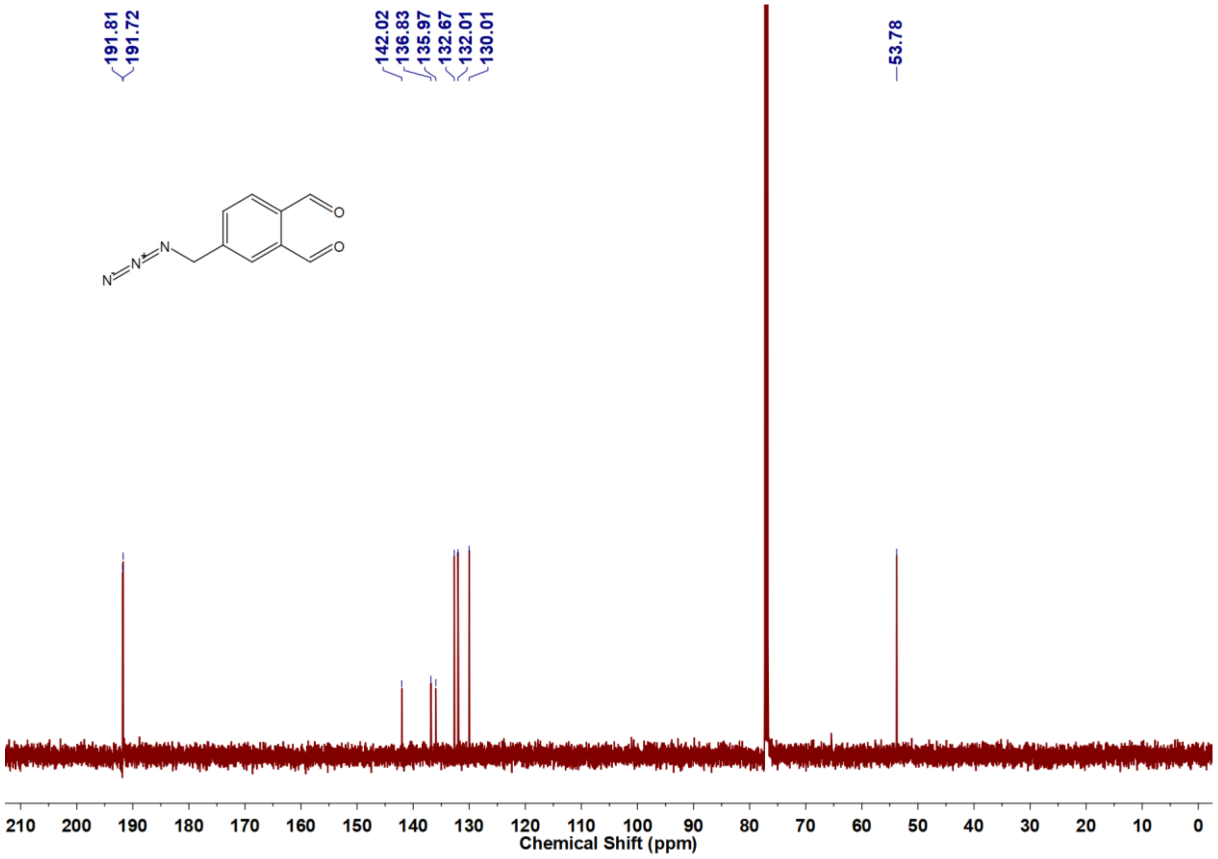

Figure S45. ^{1}H and ^{13}C NMR spectra of M7 in $CDCl_3$.

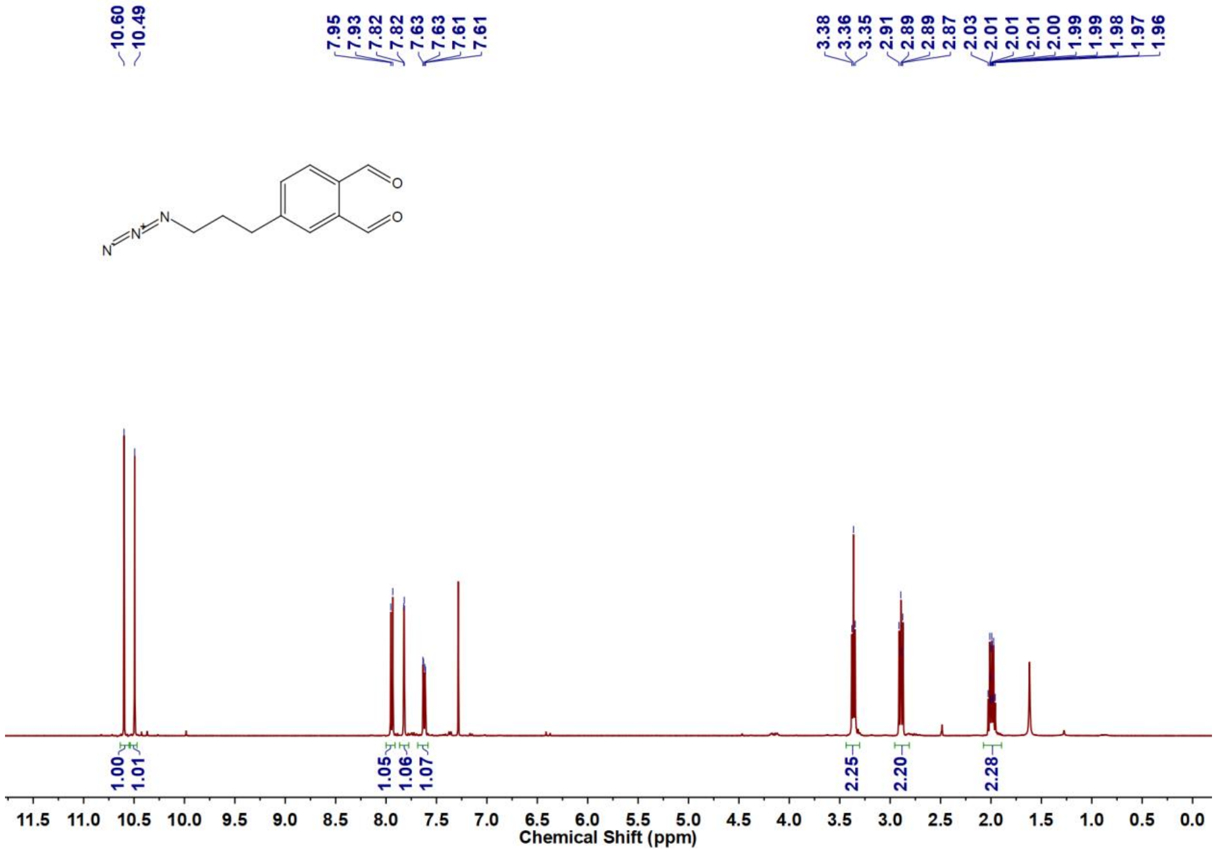

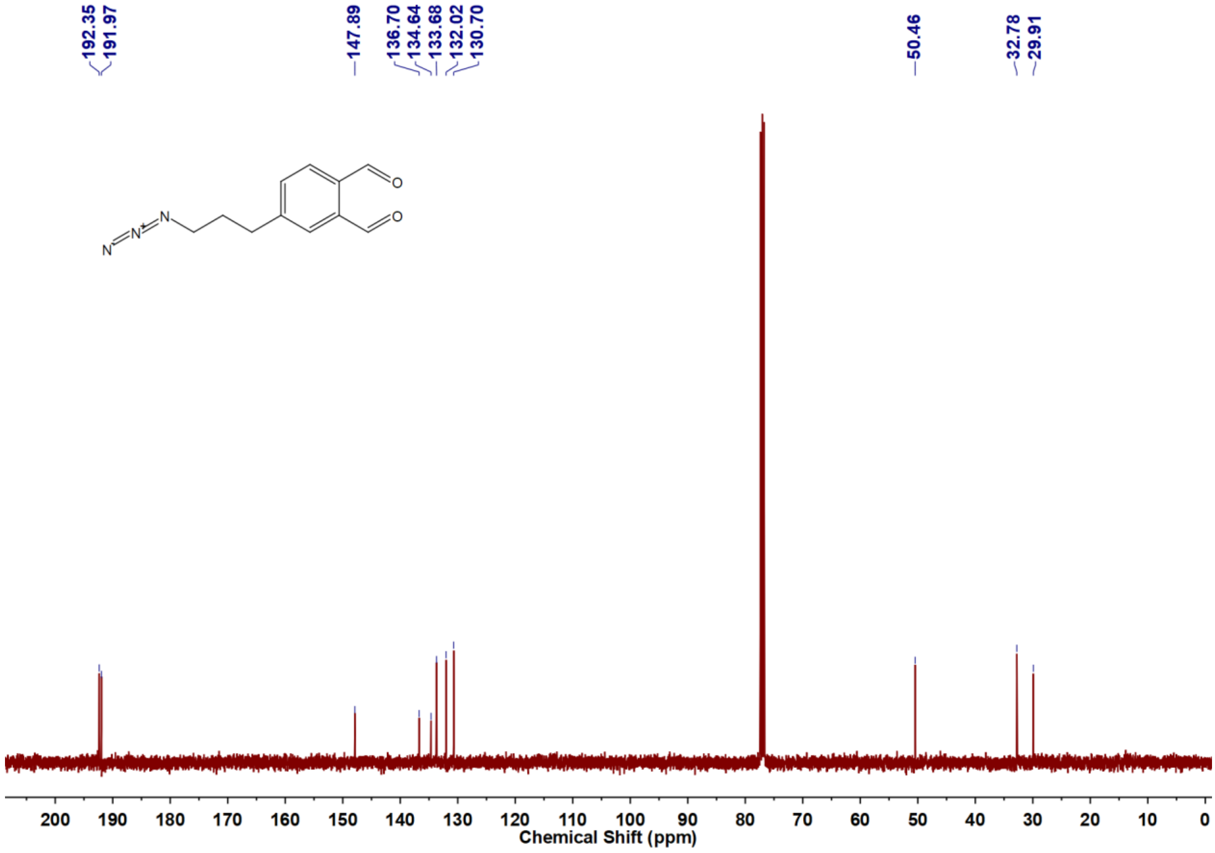

Figure S46. ^{1}H and ^{13}C NMR spectra of M8 in $CDCl_3$.

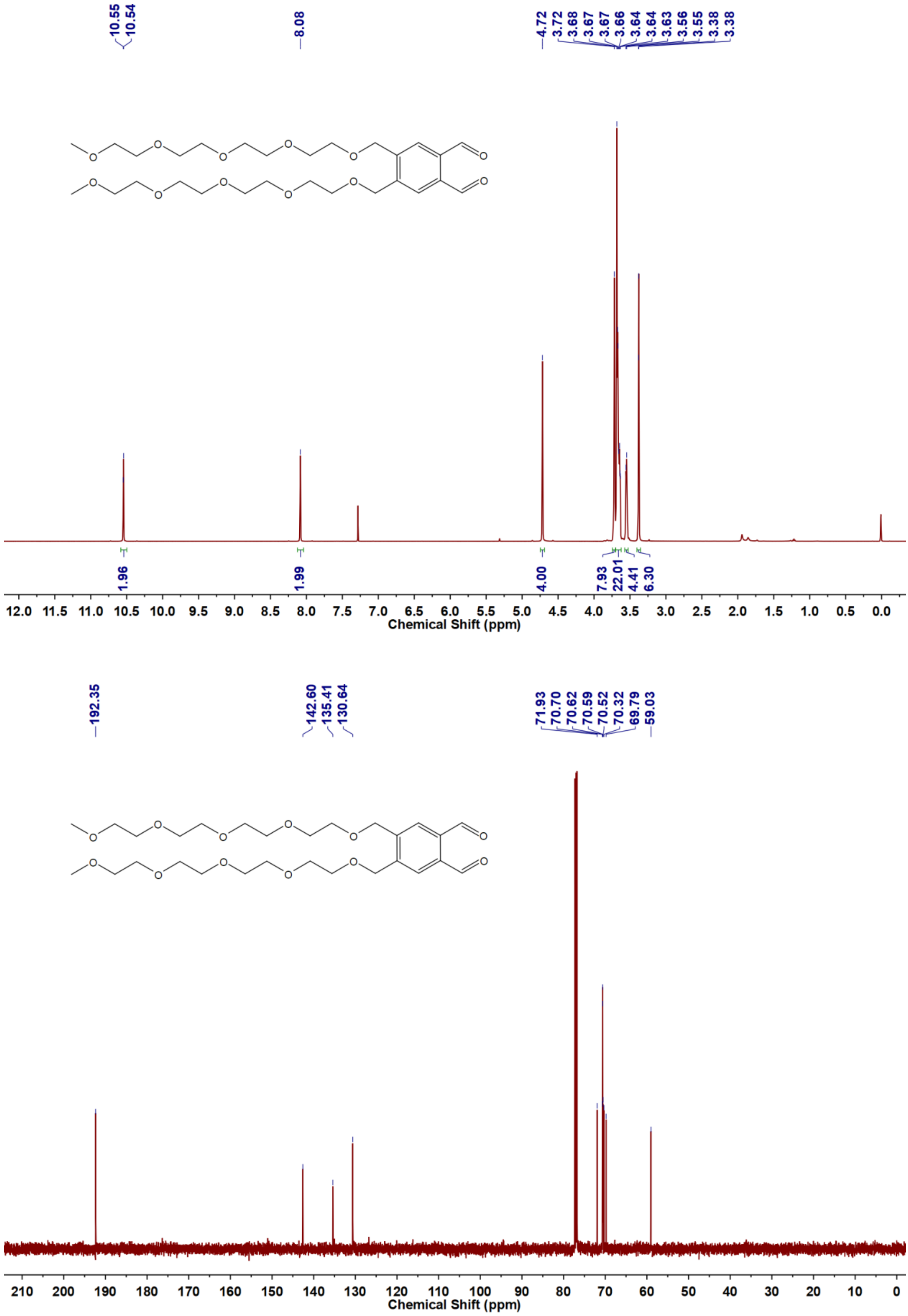

Figure S47. ^{1}H and ^{13}C NMR spectra of M9 in $CDCl_3$.

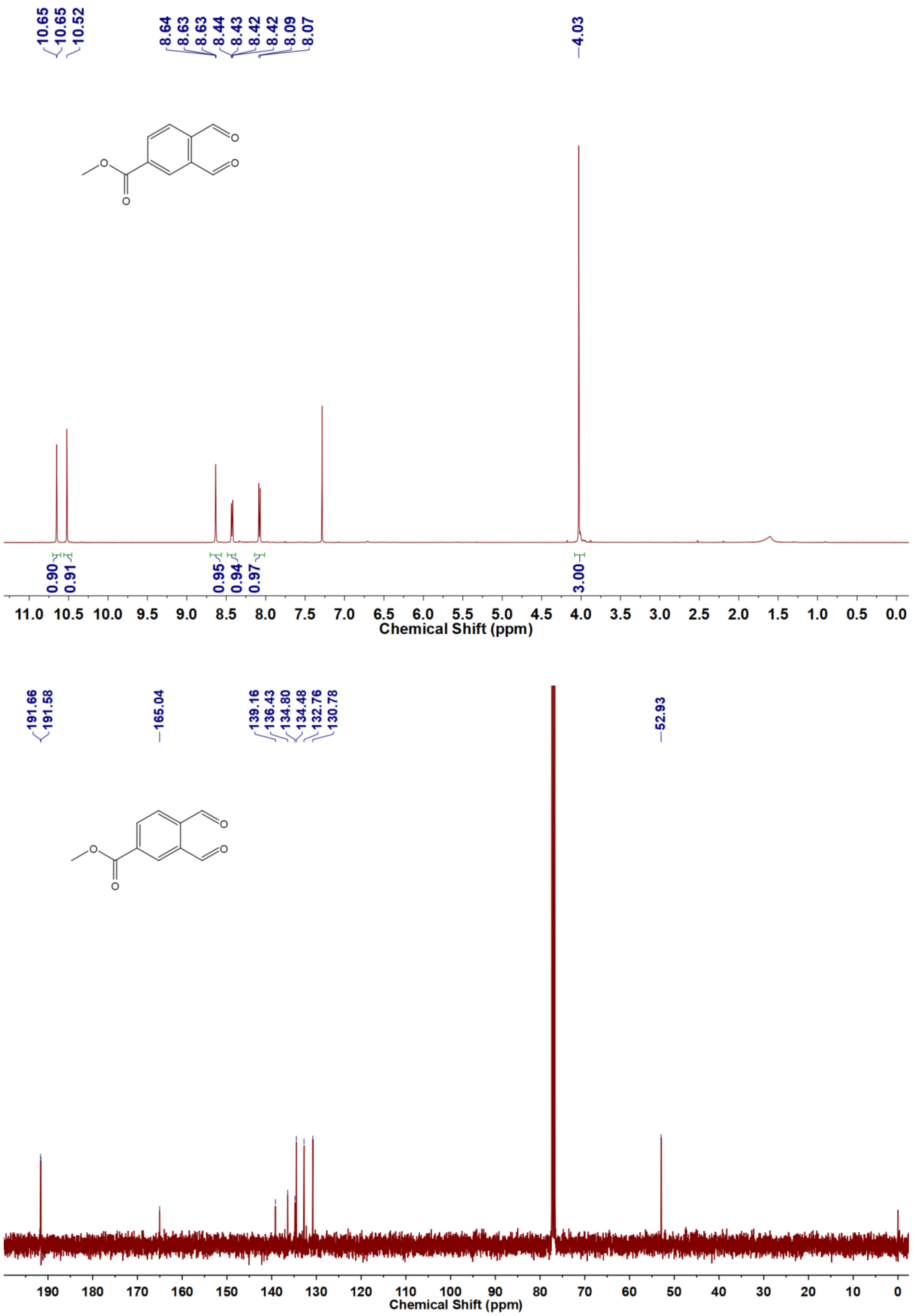

Figure S48. ^{1}H and ^{13}C NMR spectra of compound S3 in $CDCl_3$.

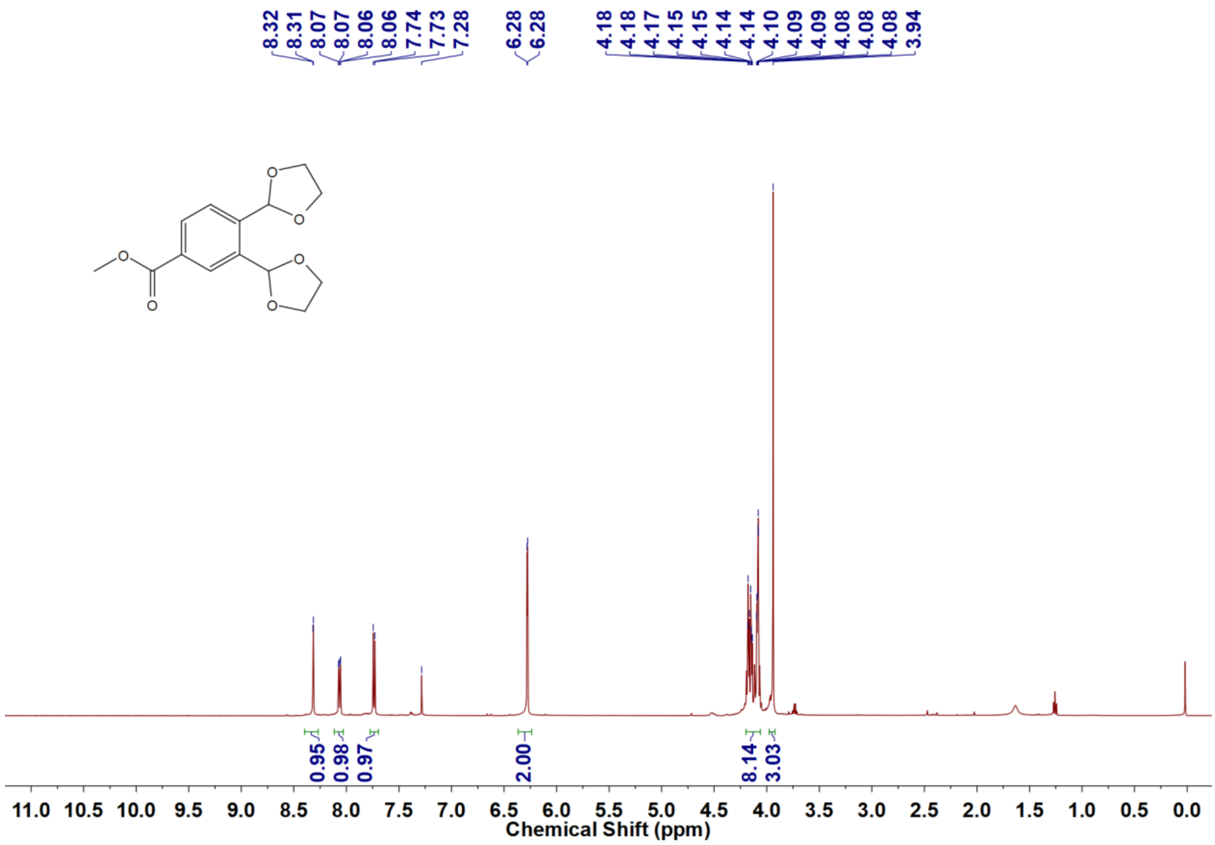

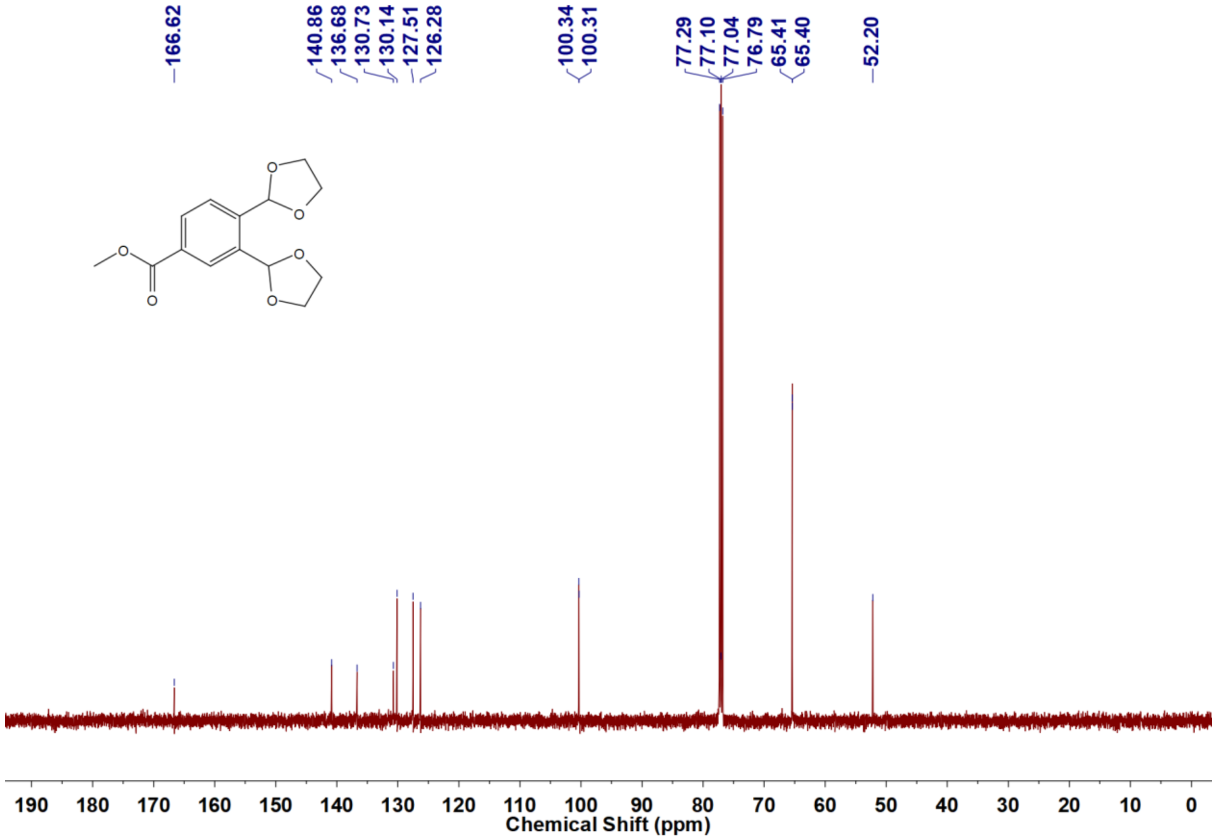

Figure S49. ^{1}H and ^{13}C NMR spectra of compound S4 in $CDCl_3$.

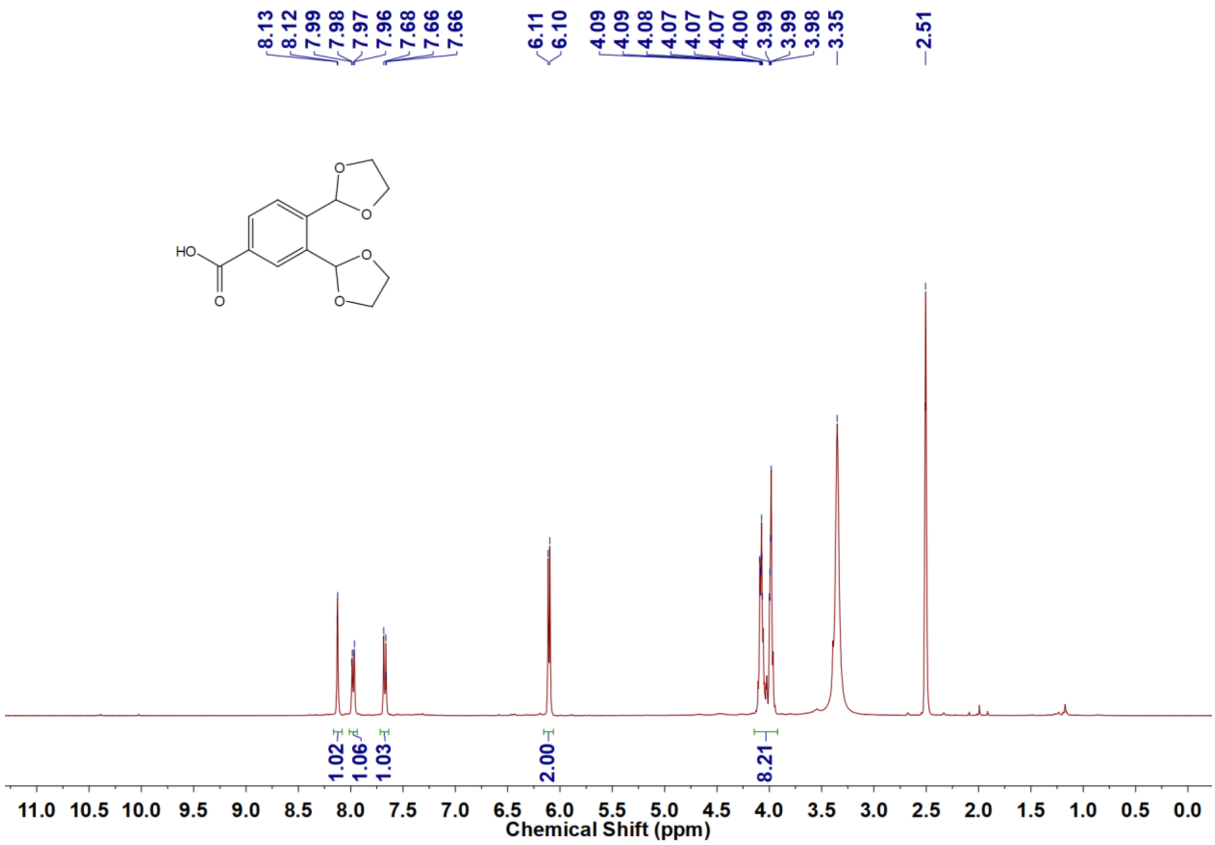

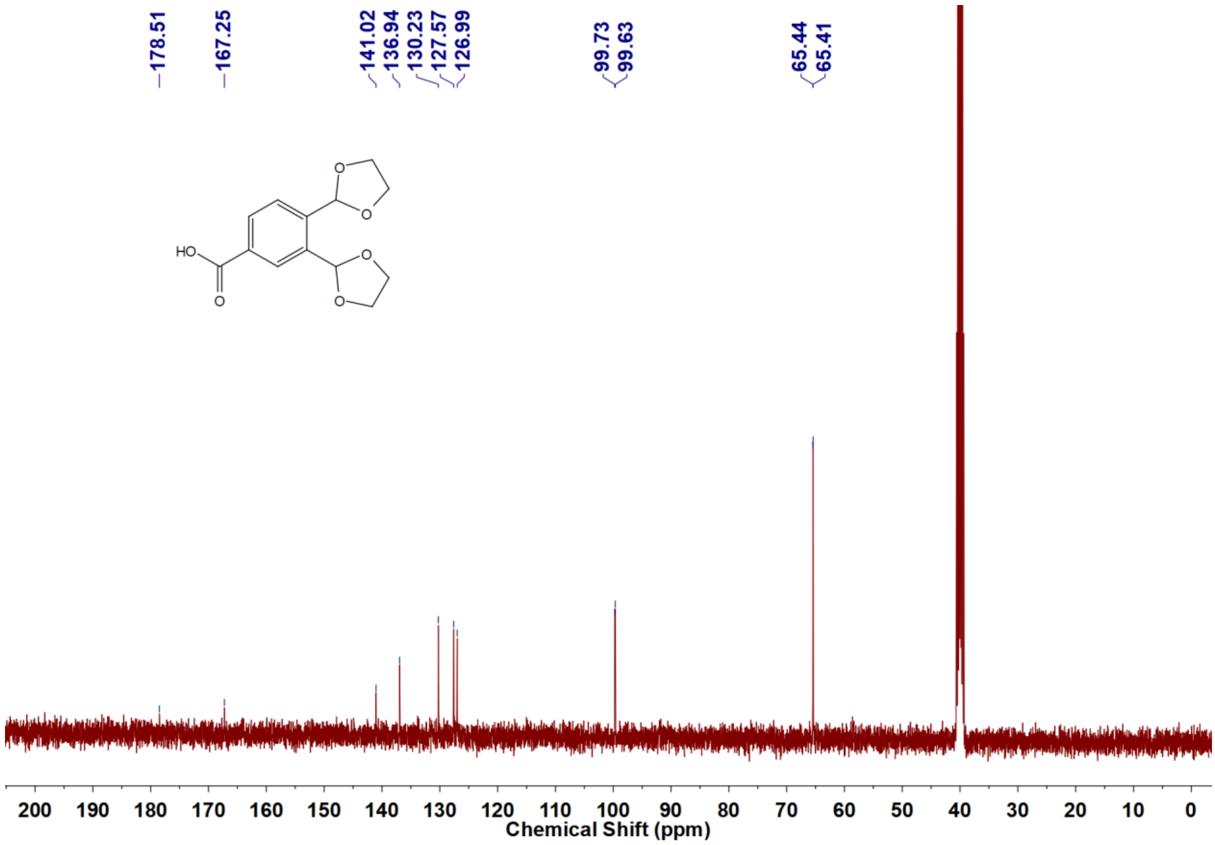

Figure S50. ^{1}H and ^{13}C NMR spectra of compound S5 in DMSO.

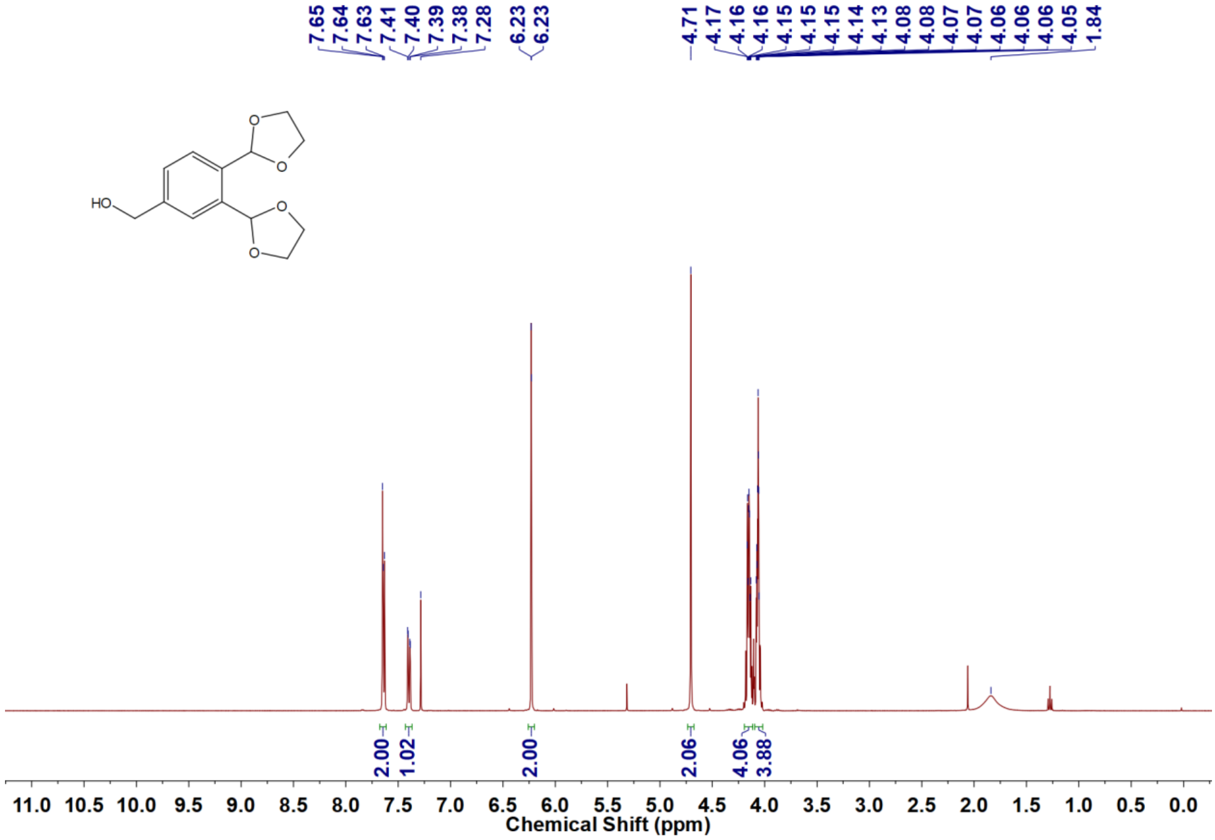

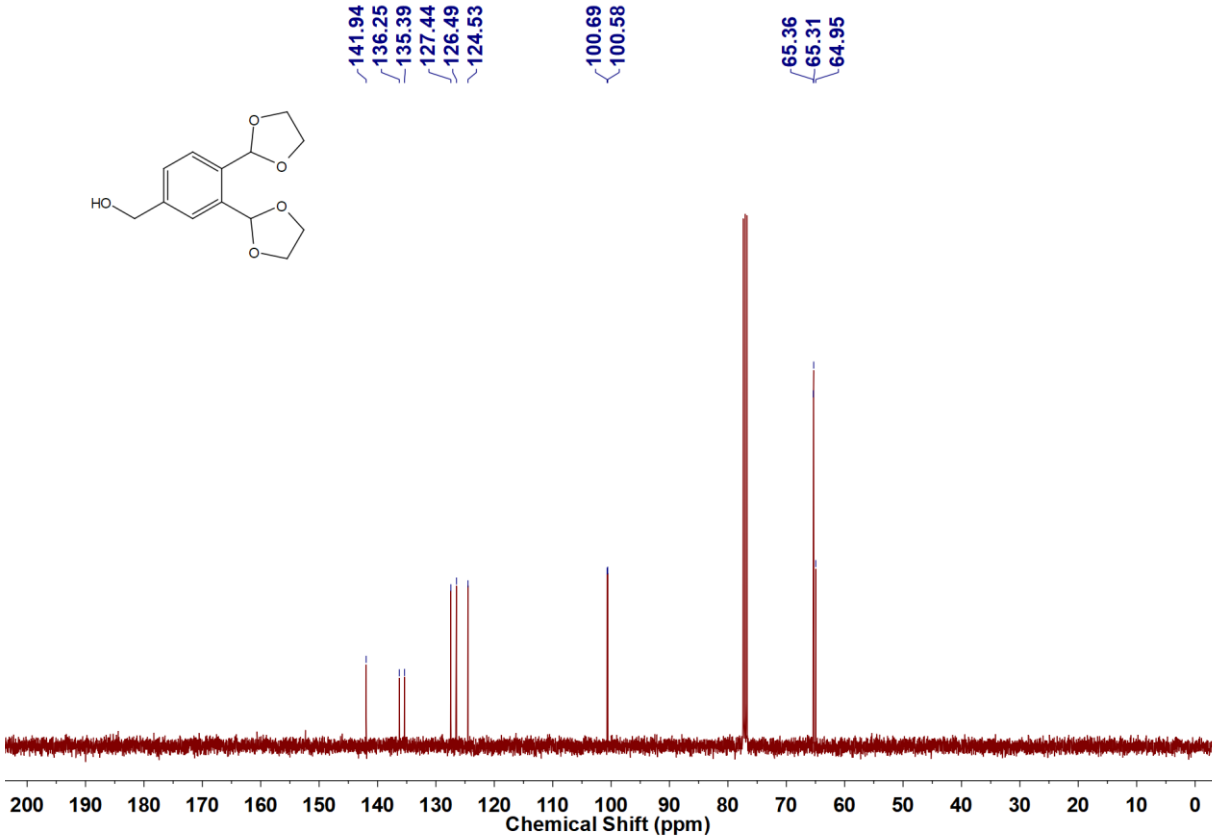

Figure S51. ^{1}H and ^{13}C NMR spectra of compound S6 in $CDCl_3$.

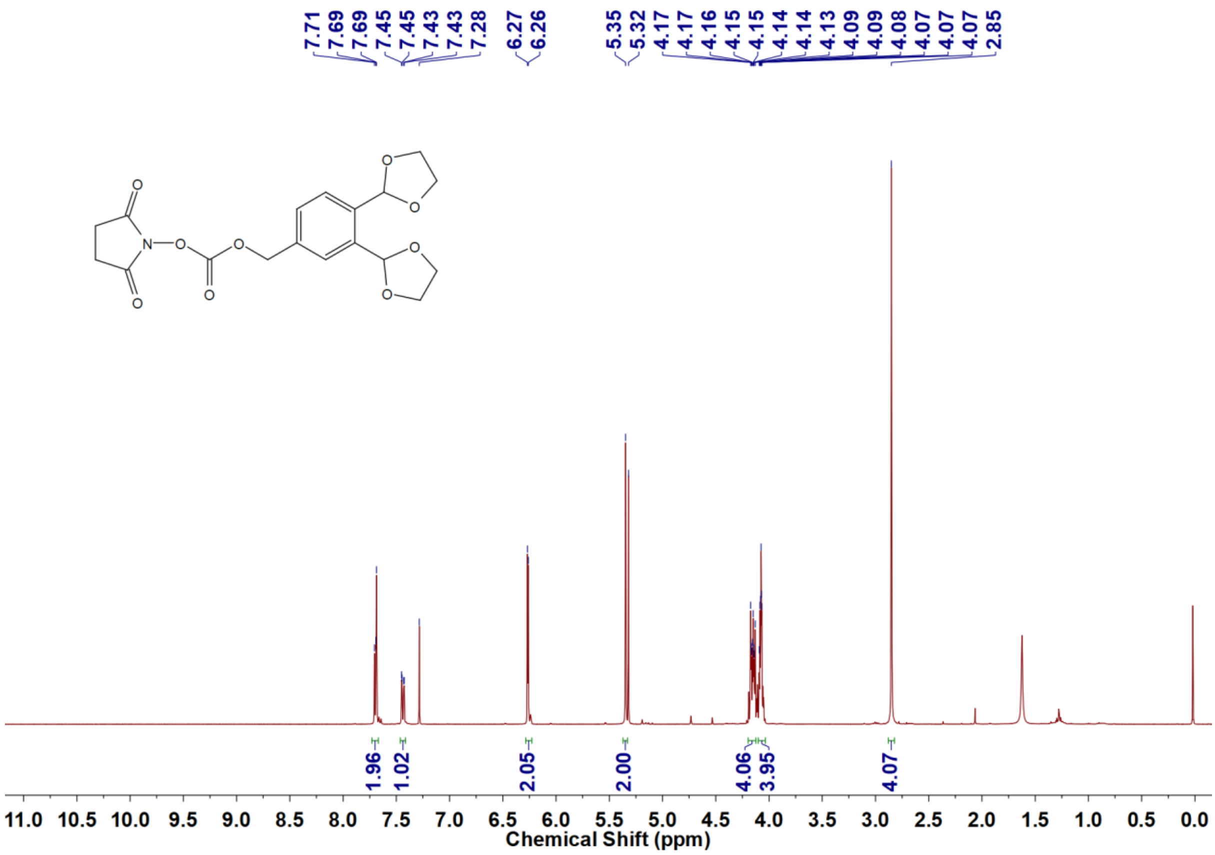

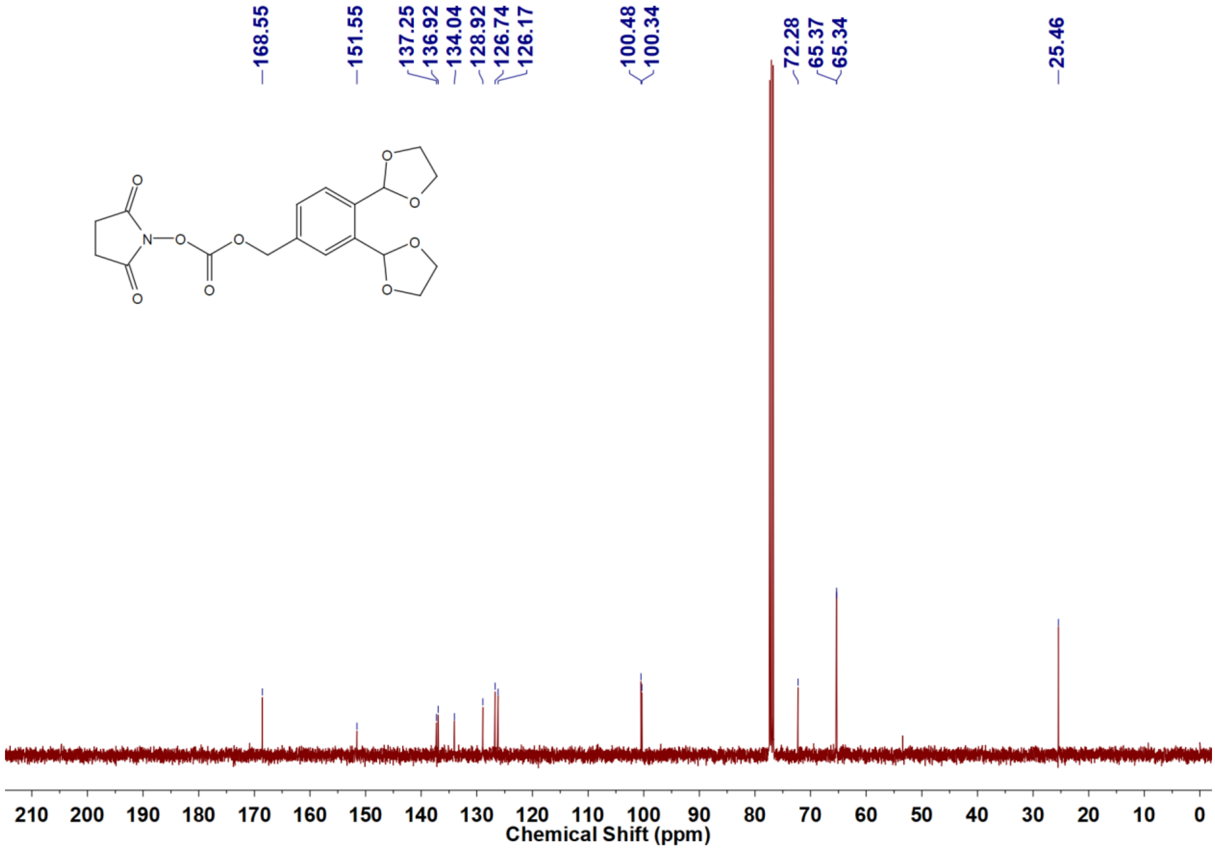

Figure S52. ^{1}H and ^{13}C NMR spectra of compound S7 in $CDCl_3$.

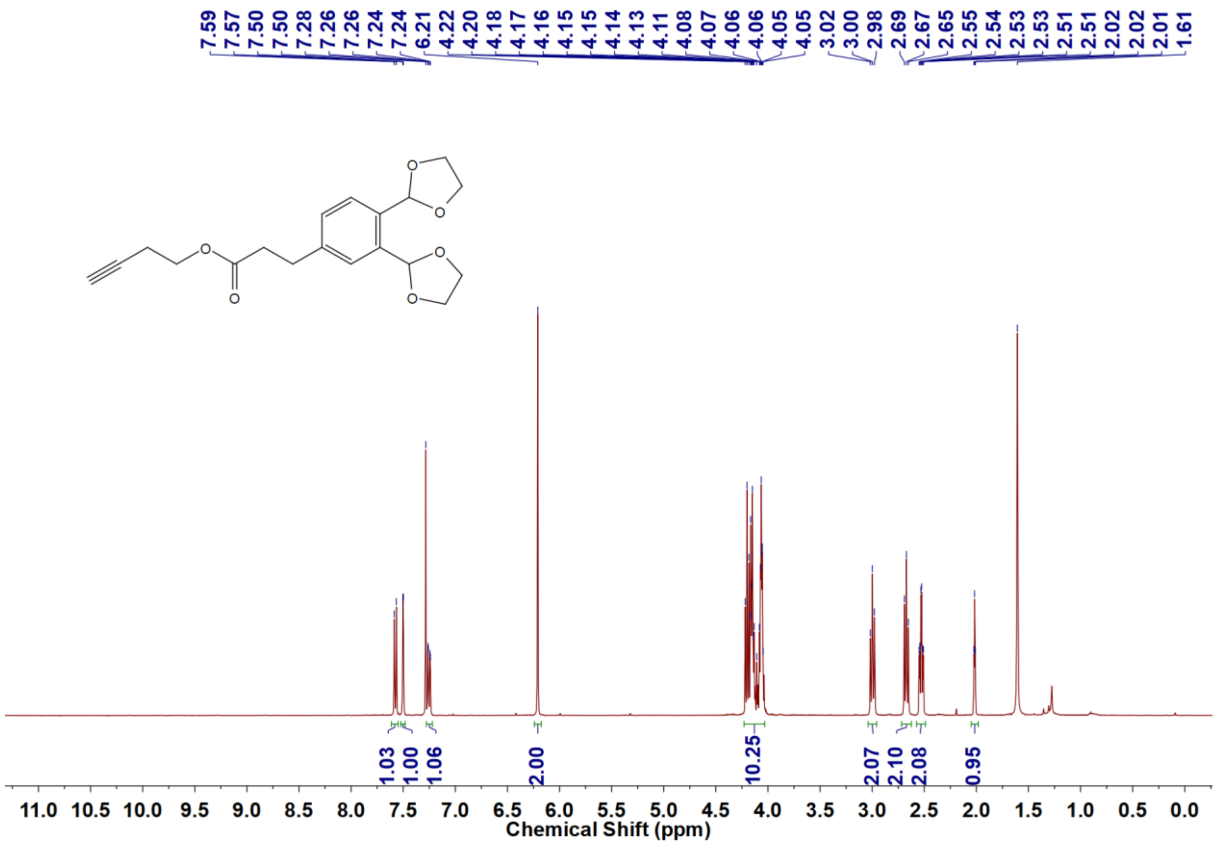

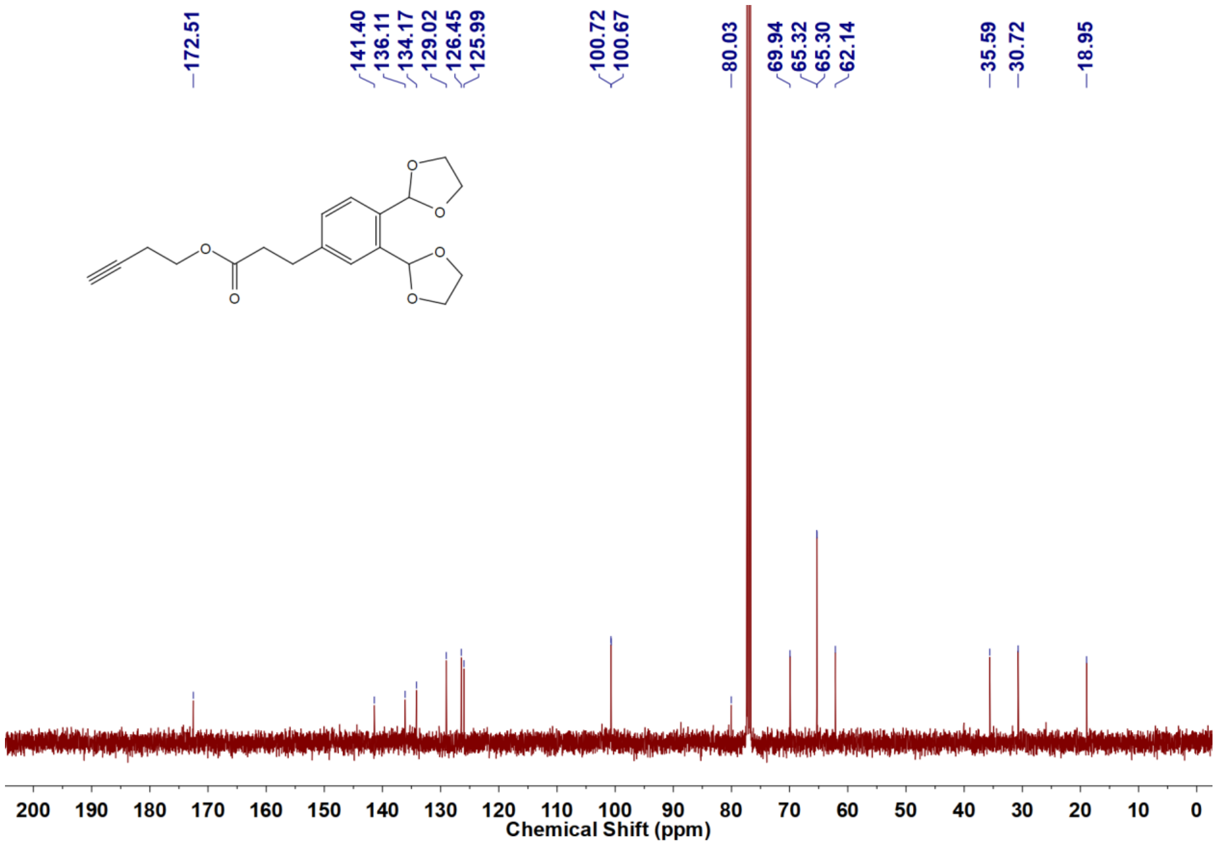

Figure S53. ^{1}H and ^{13}C NMR spectra of compound S9 in $CDCl_3$.

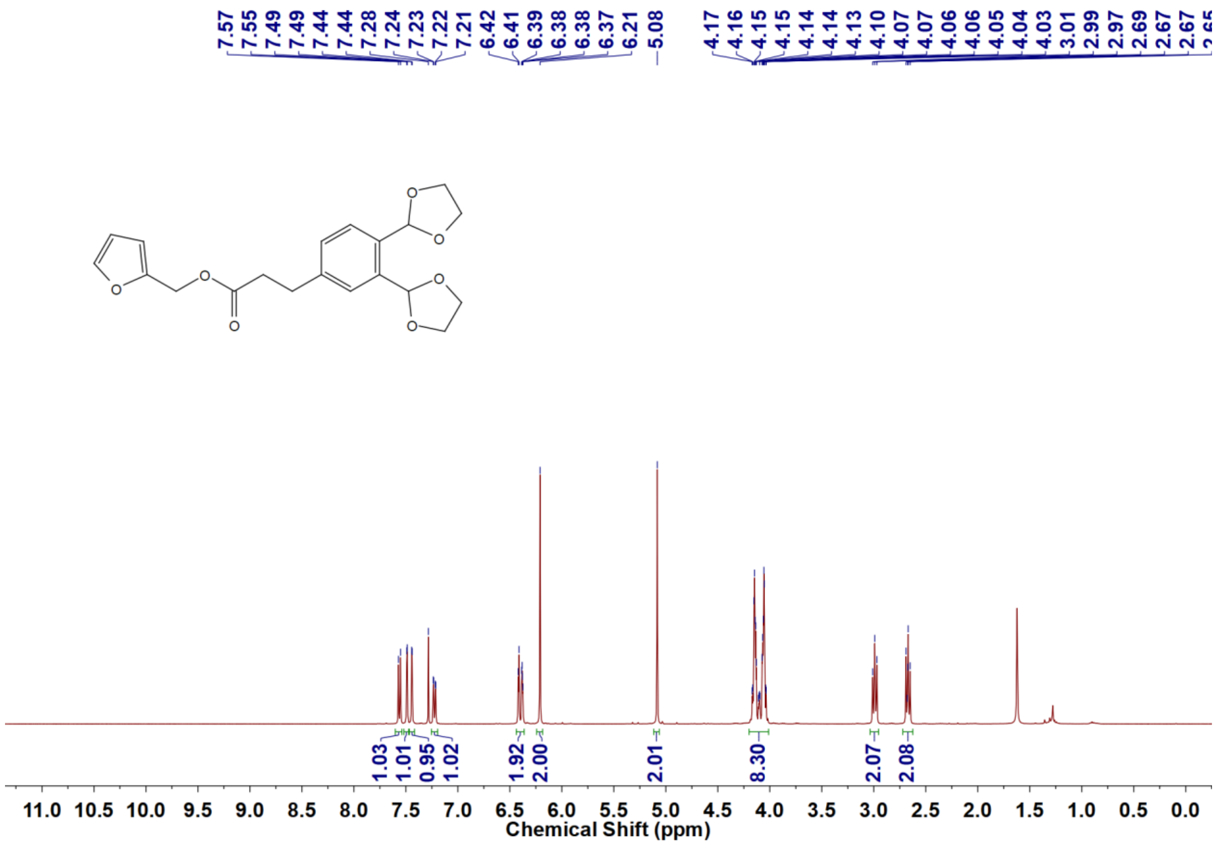

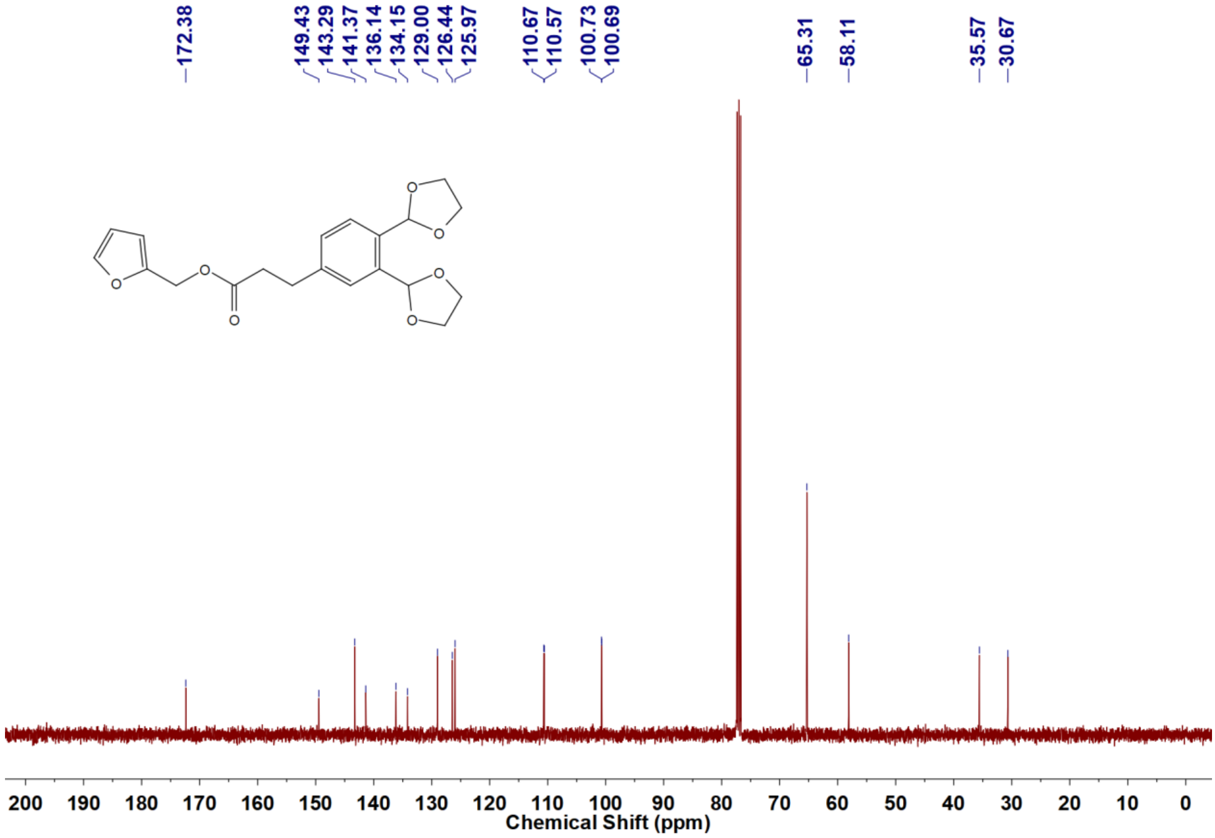

Figure S54. ^{1}H and ^{13}C NMR spectra of compound S10 in $CDCl_3$.

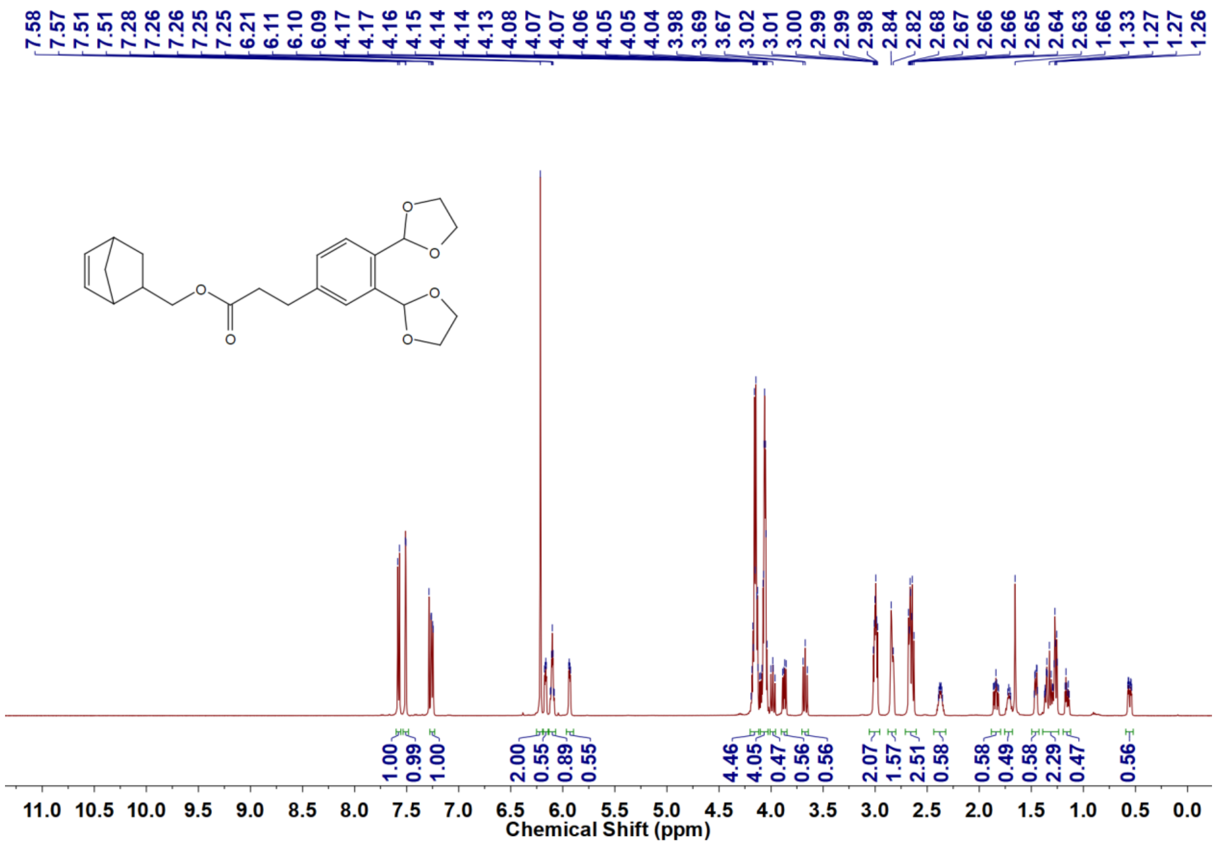

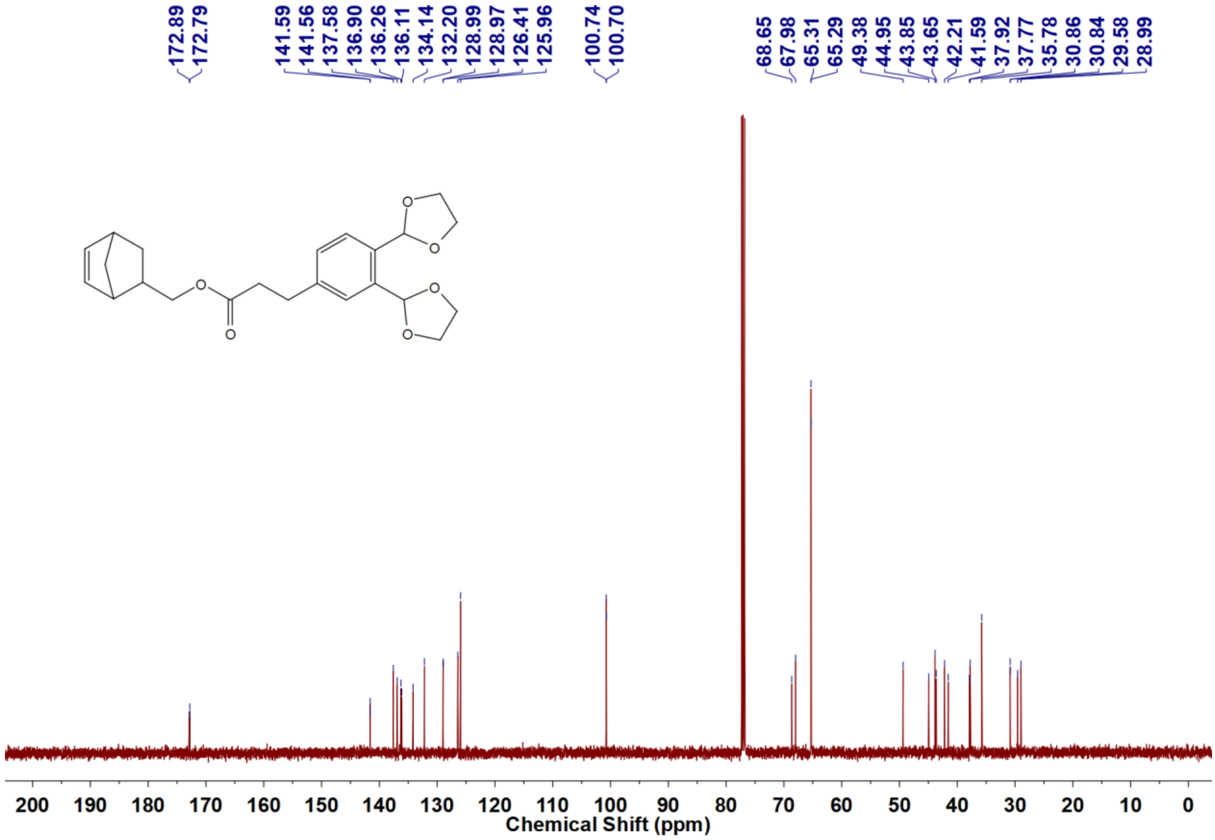

Figure S55. ^{1}H and ^{13}C NMR spectra of compound S11 in $CDCl_3$.

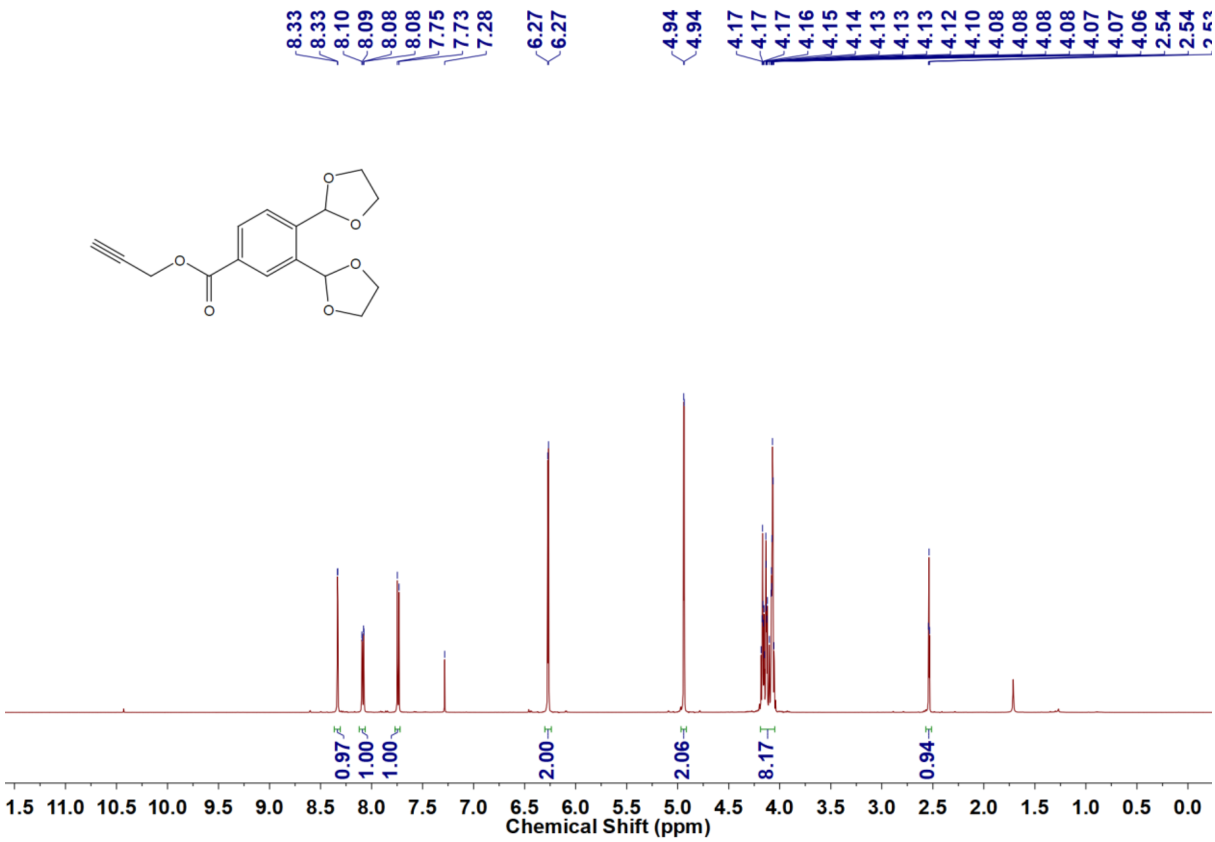

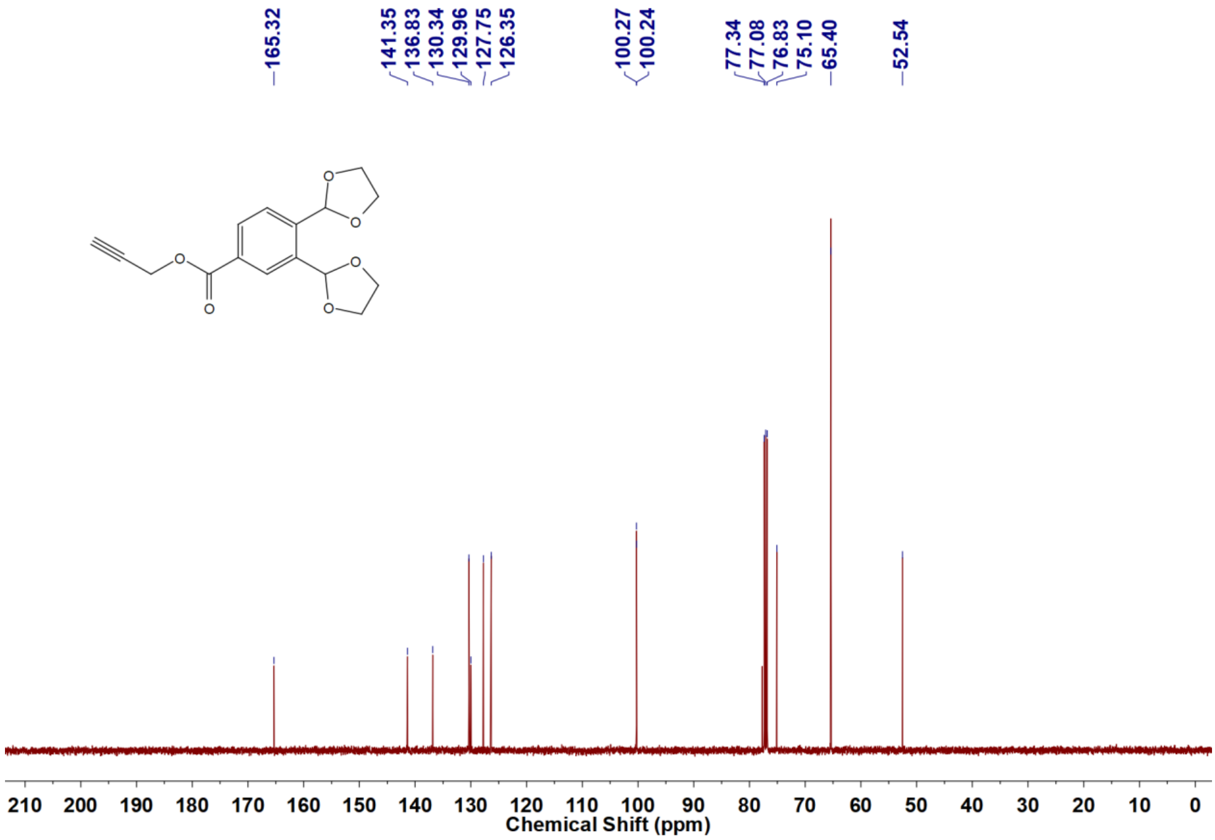

Figure S56. ^{1}H and ^{13}C NMR spectra of compound S12 in $CDCl_3$.

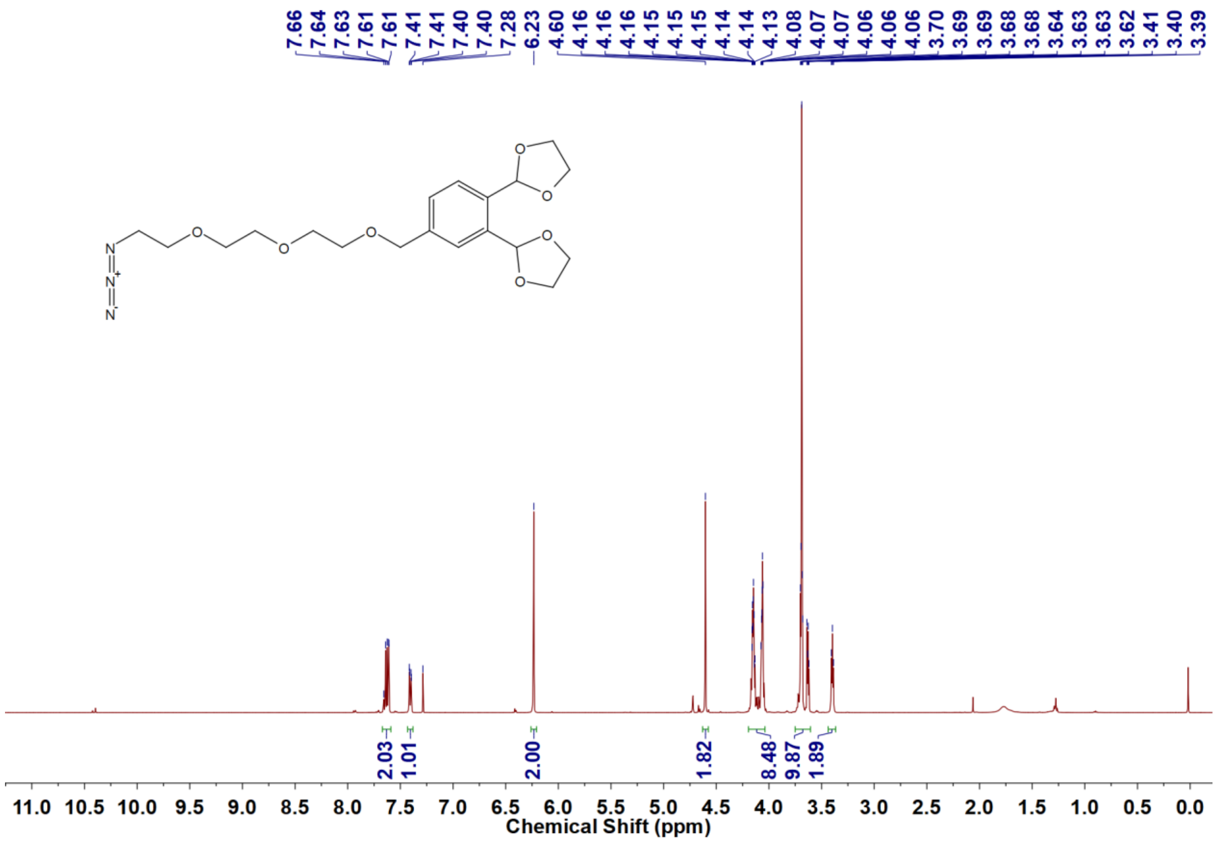

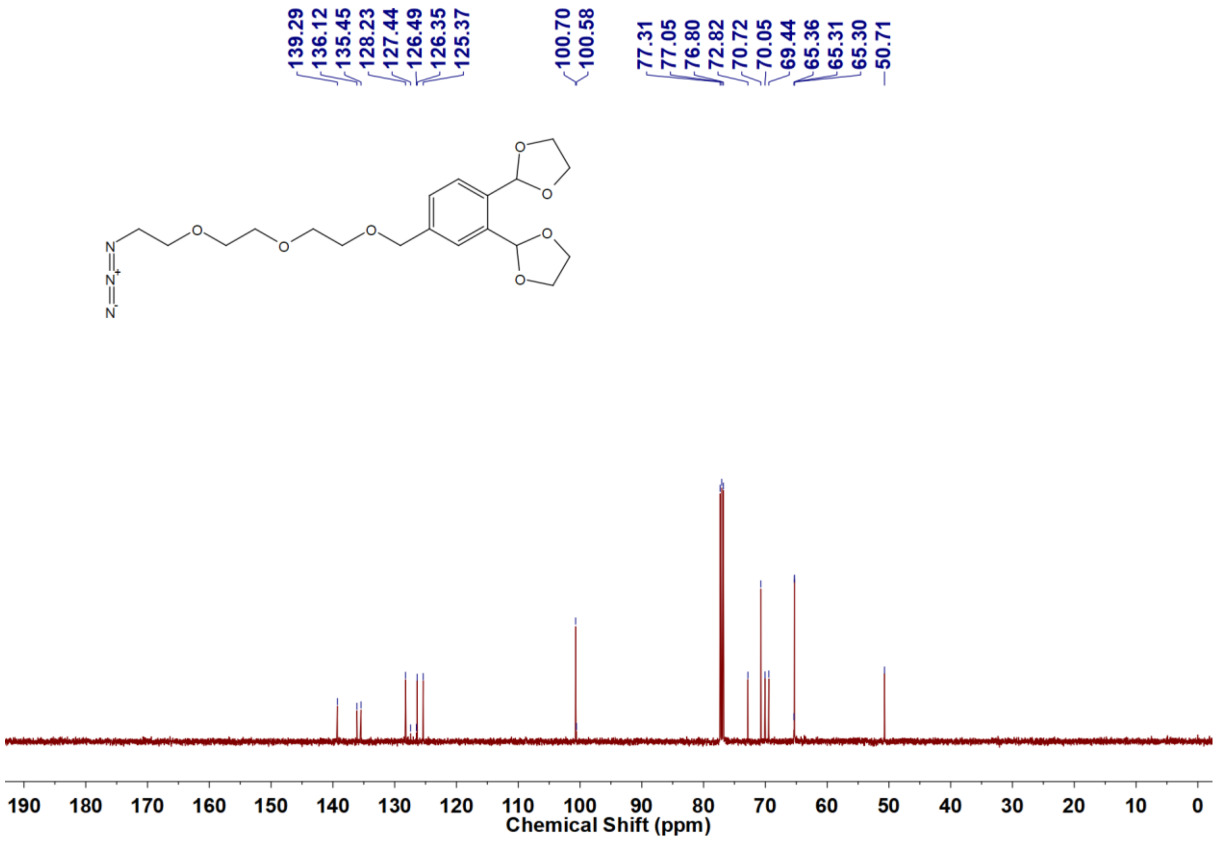

Figure S57. ^{1}H and ^{13}C NMR spectra of compound S13 in $CDCl_3$.

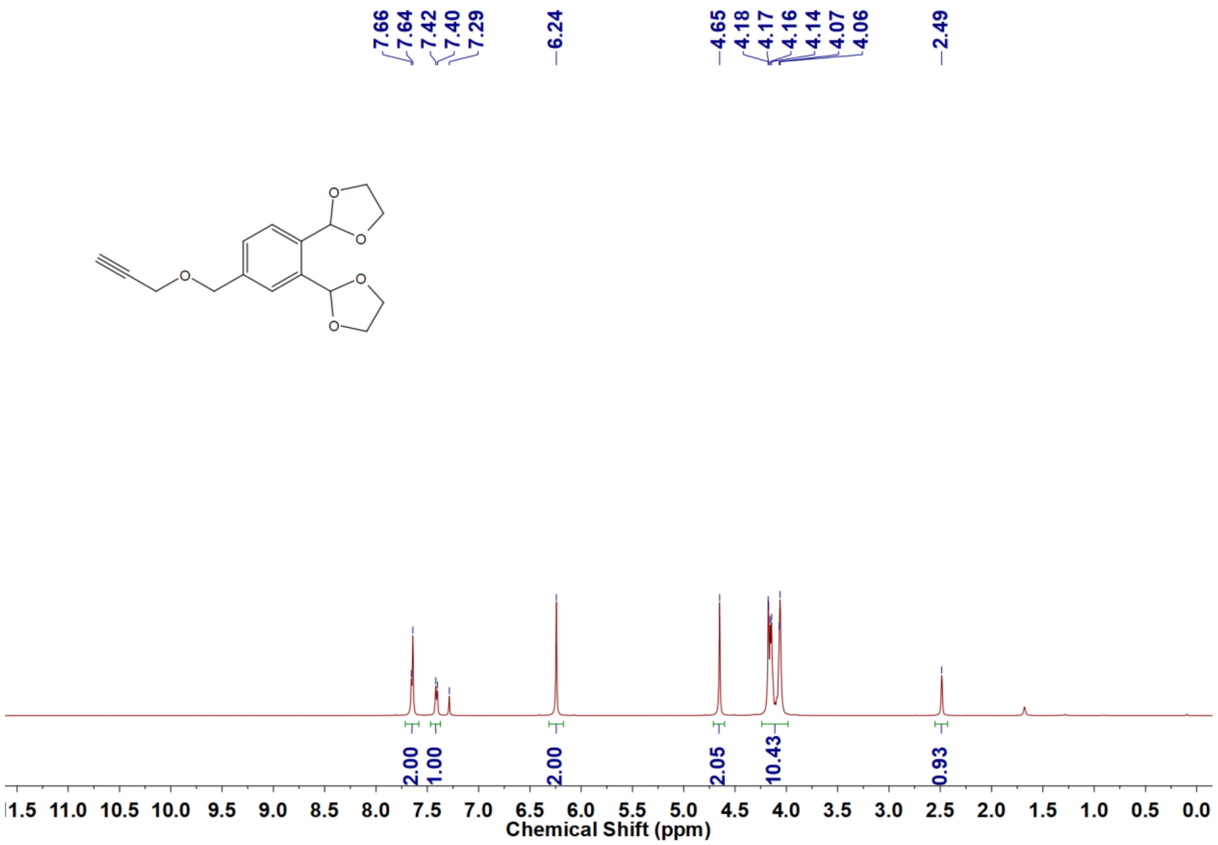

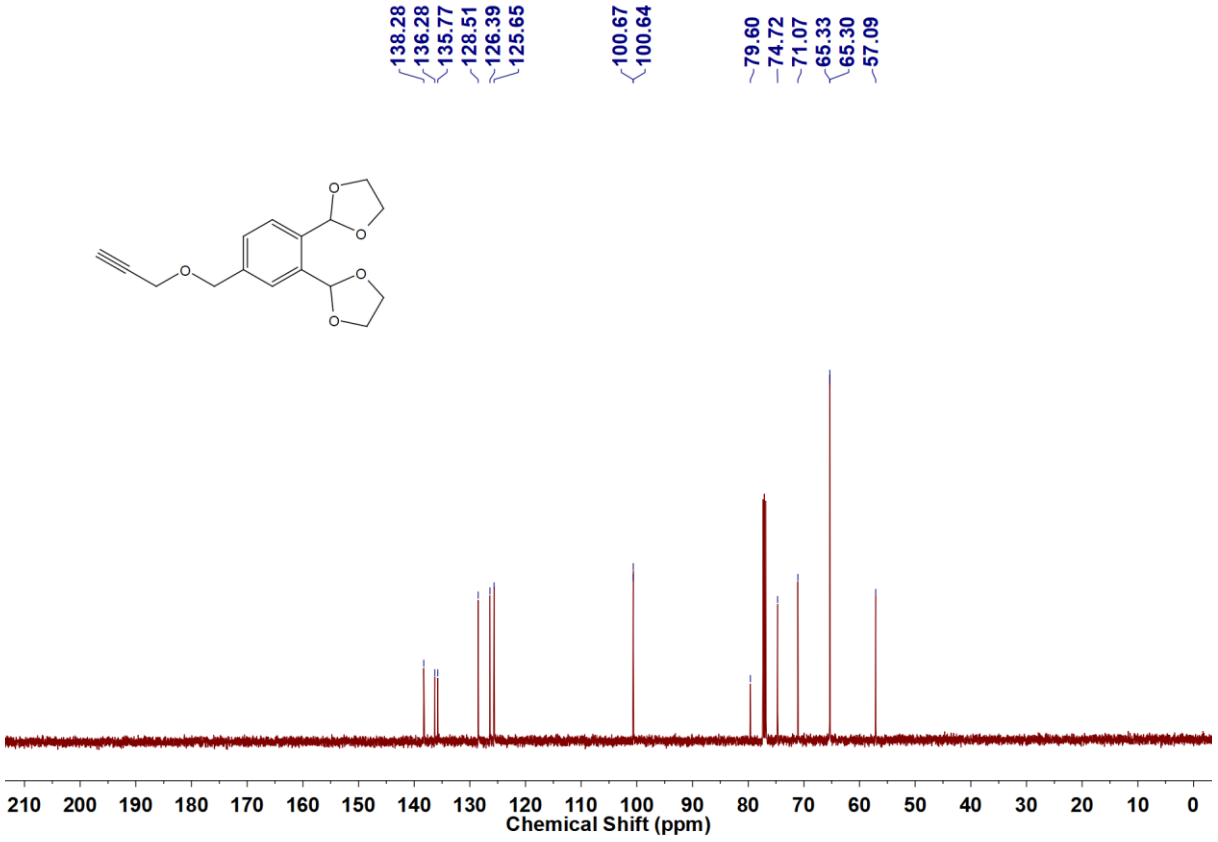

Figure S58. ^{1}H and ^{13}C NMR spectra of compound S14 in $CDCl_3$.

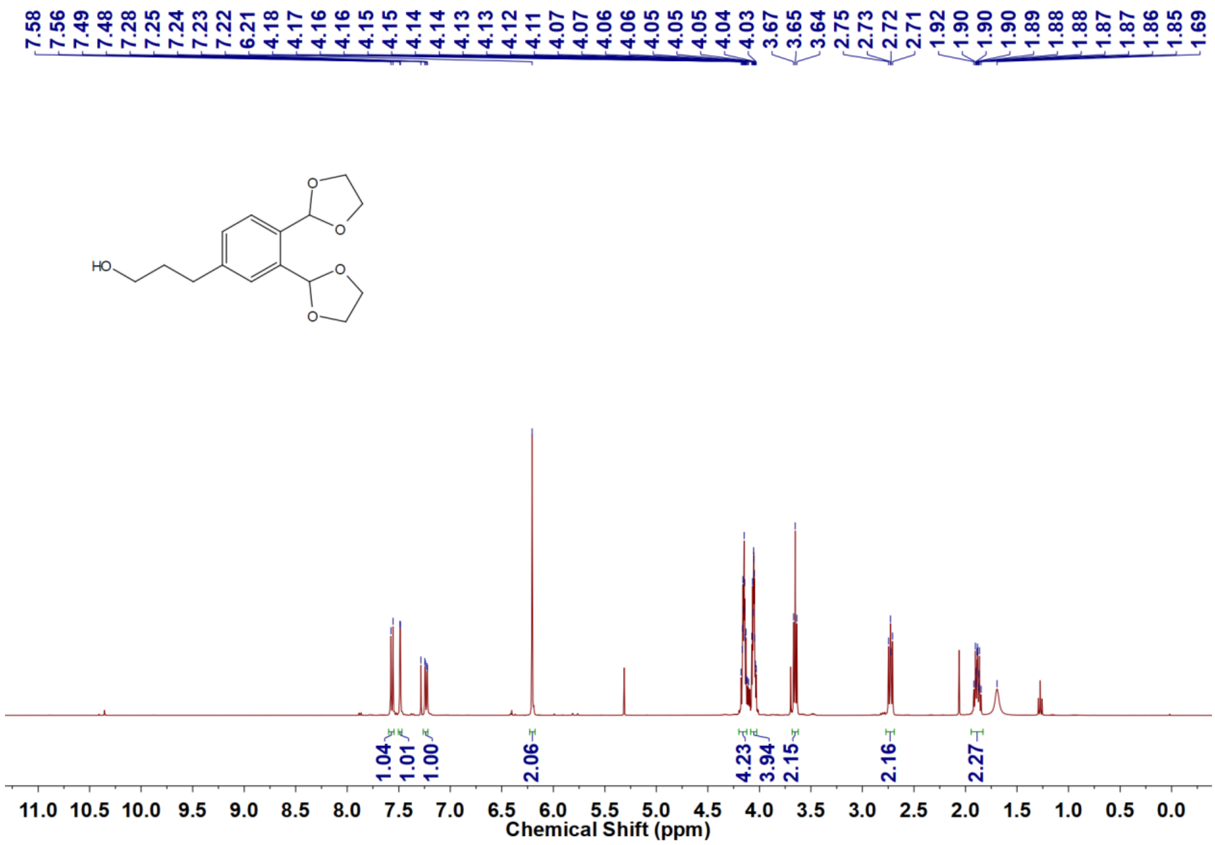

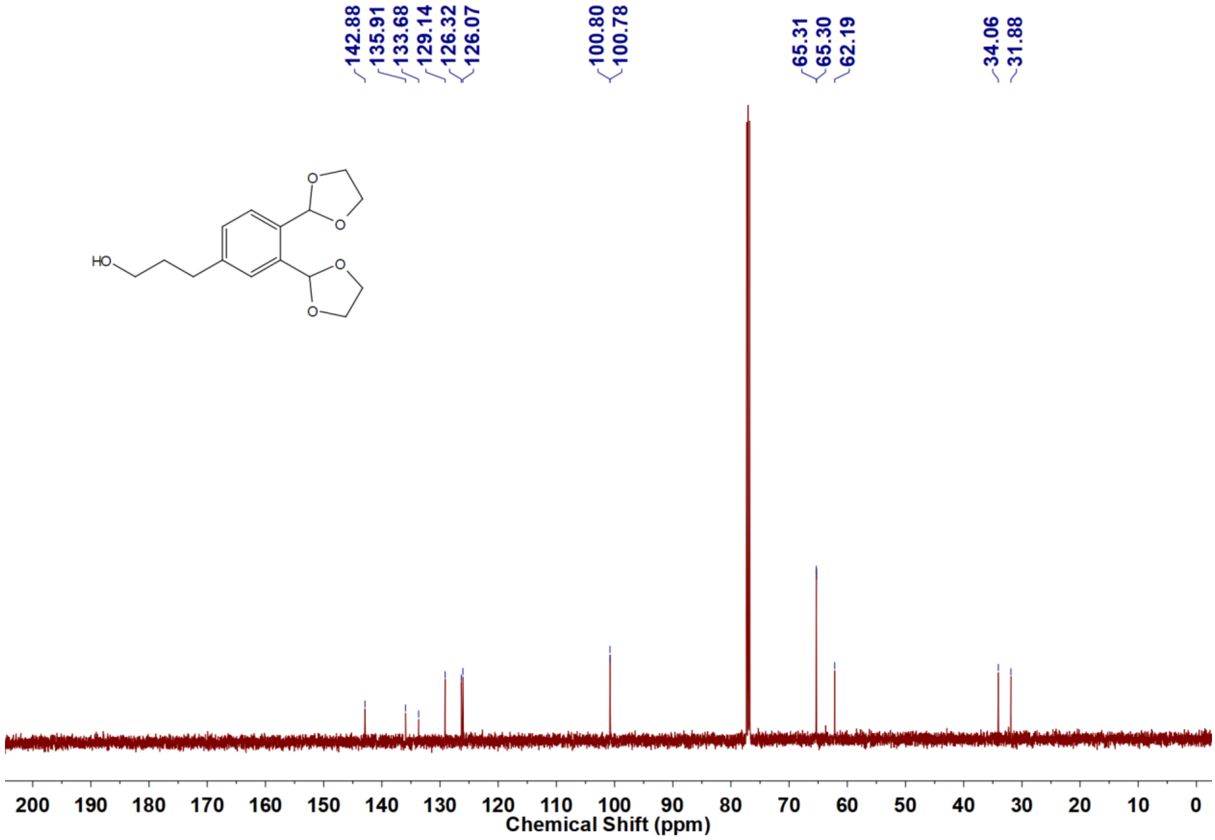

Figure S59. ^{1}H and ^{13}C NMR spectra of compound S16 in $CDCl_3$.

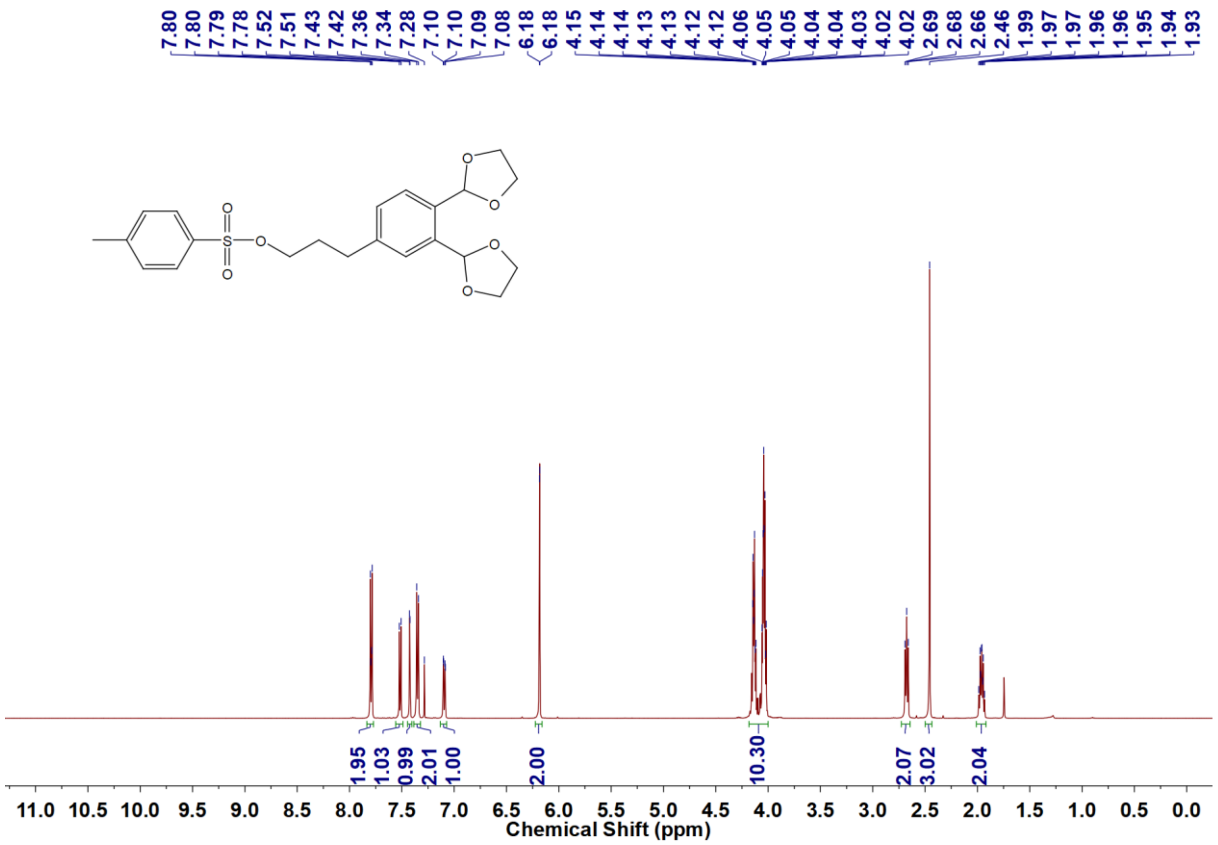

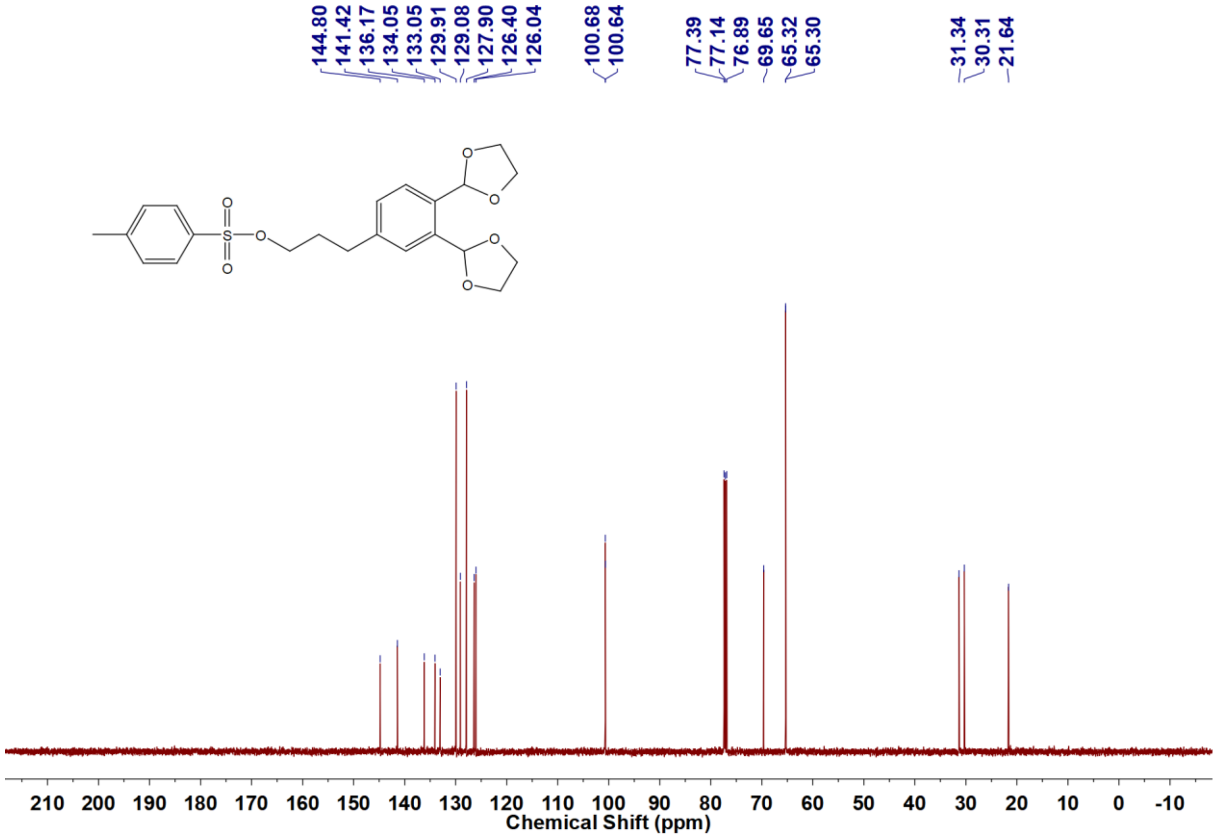

Figure S60. ^{1}H and ^{13}C NMR spectra of compound S17 in $CDCl_3$.

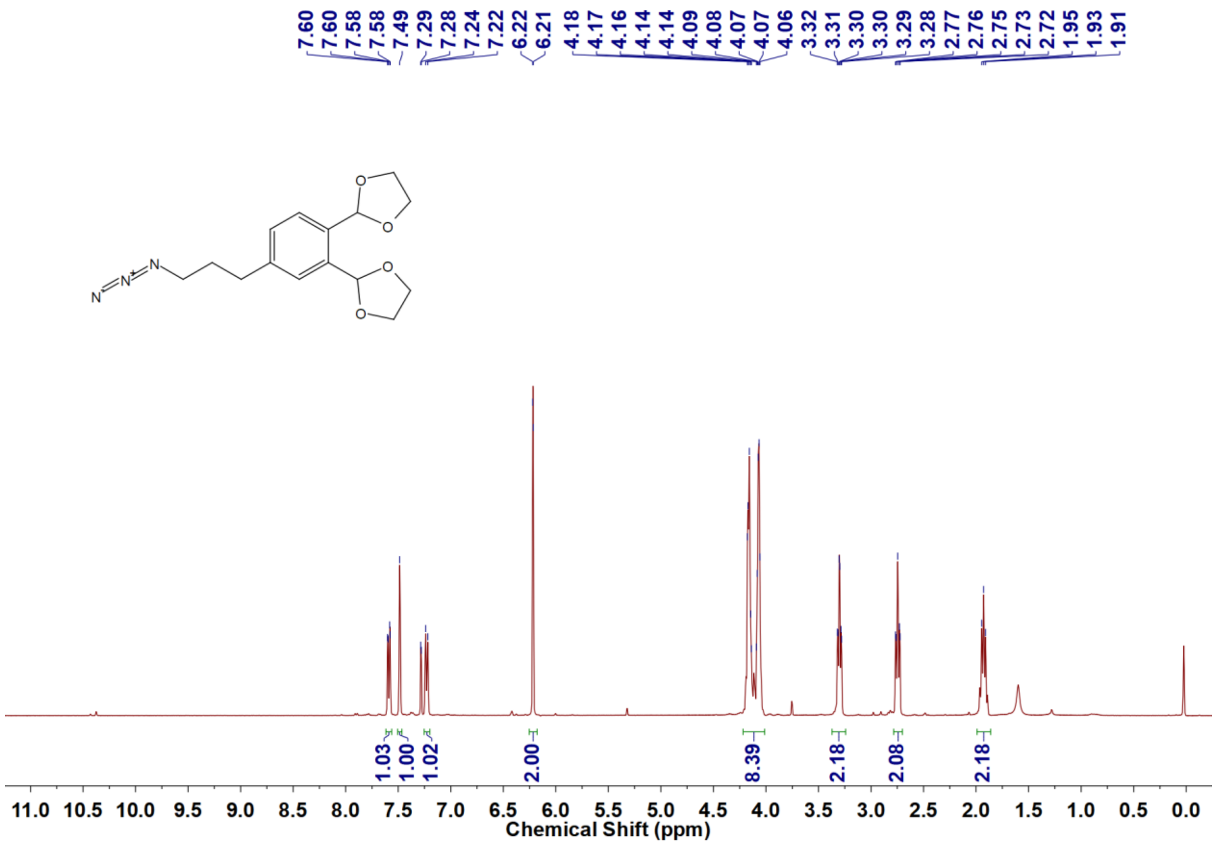

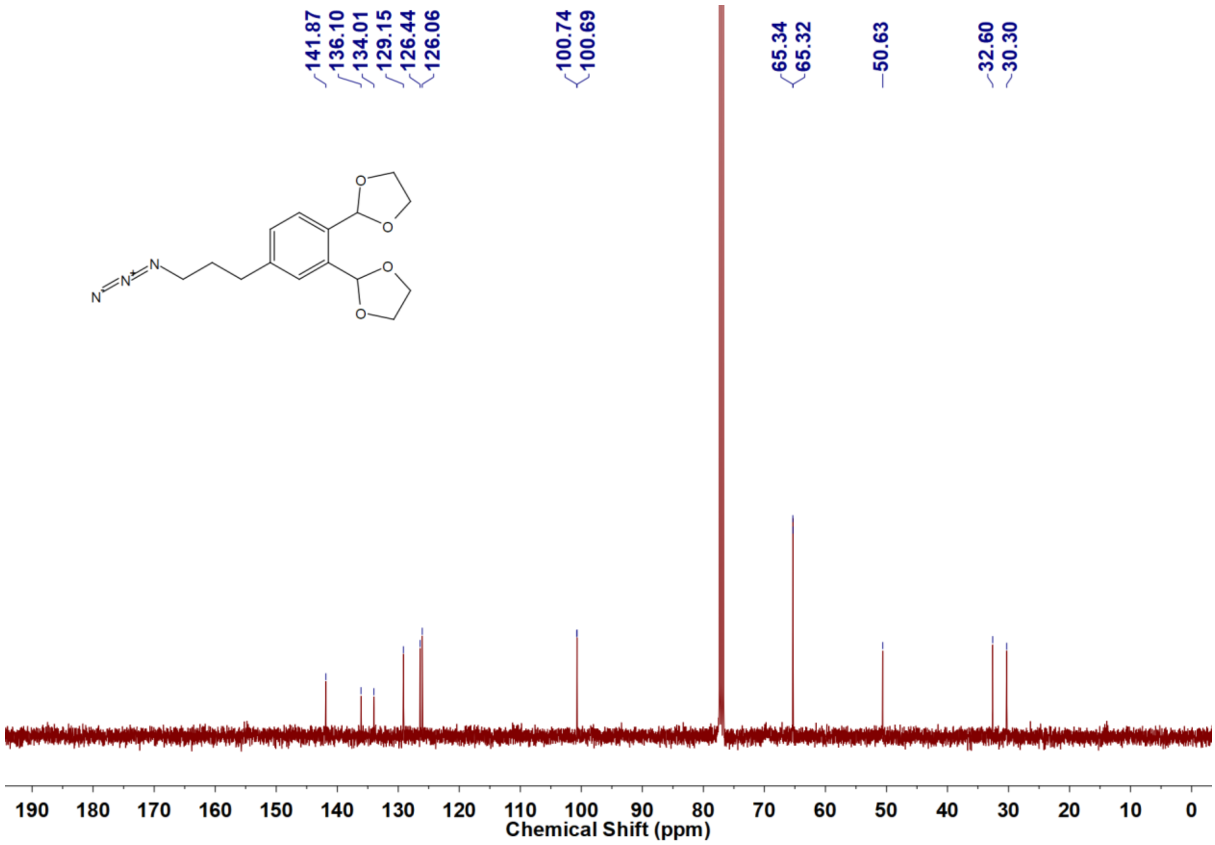

Figure S61. ^{1}H and ^{13}C NMR spectra of-compound S18 in $CDCl_3$.

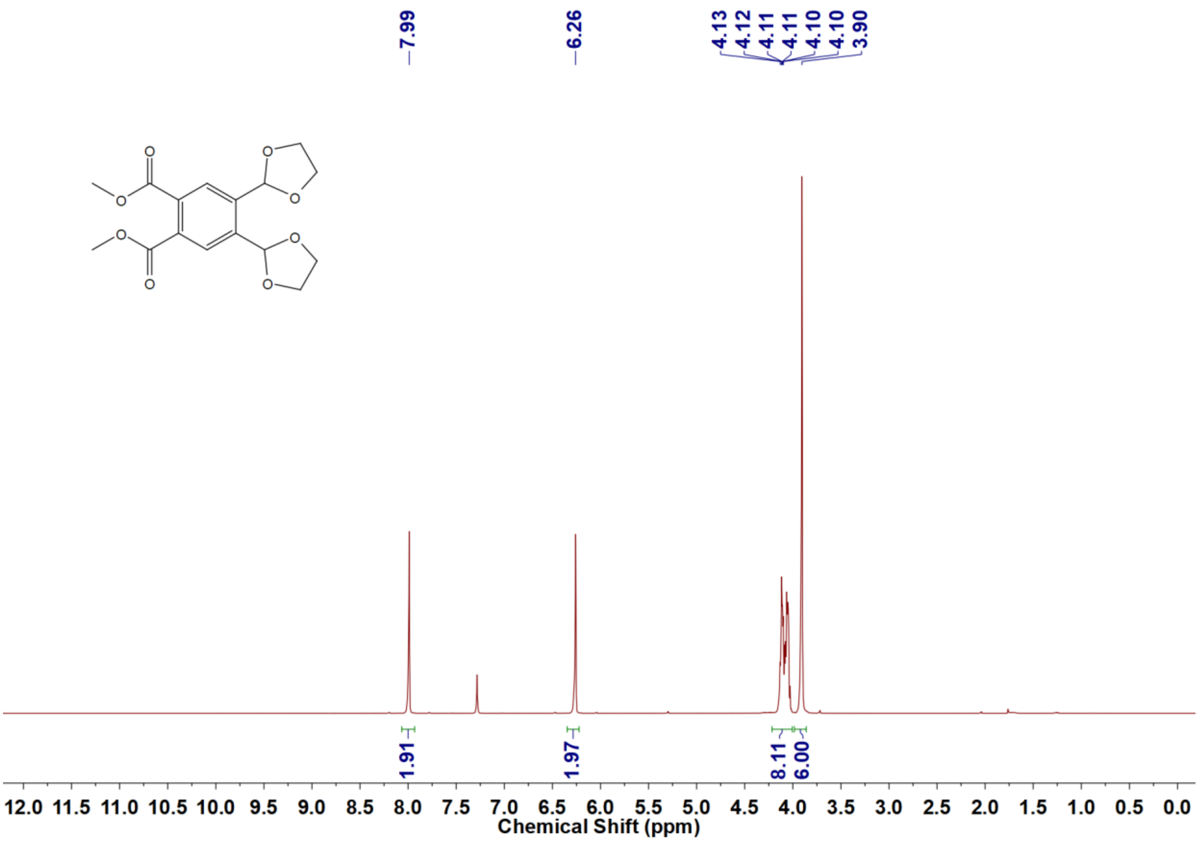

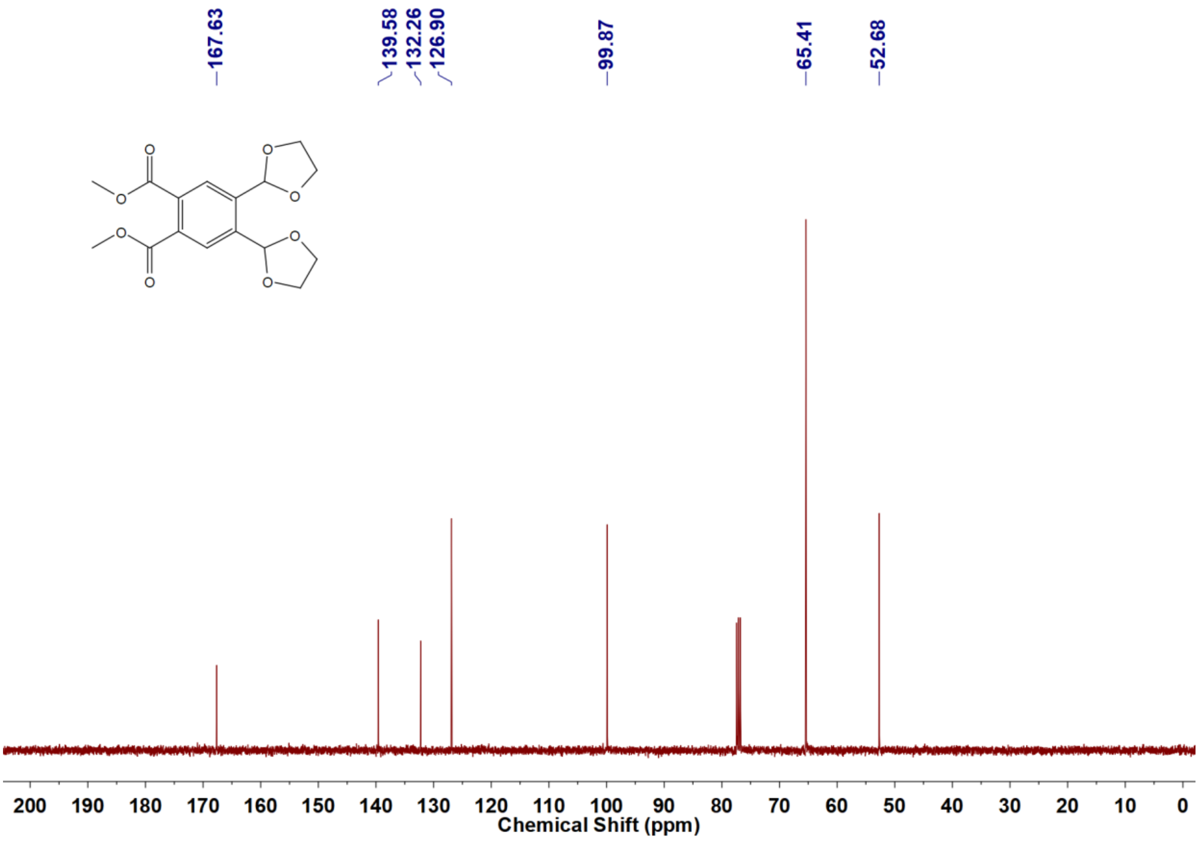

Figure S62. ^{1}H and ^{13}C NMR spectra of Compound S20 in $CDCl_3$.

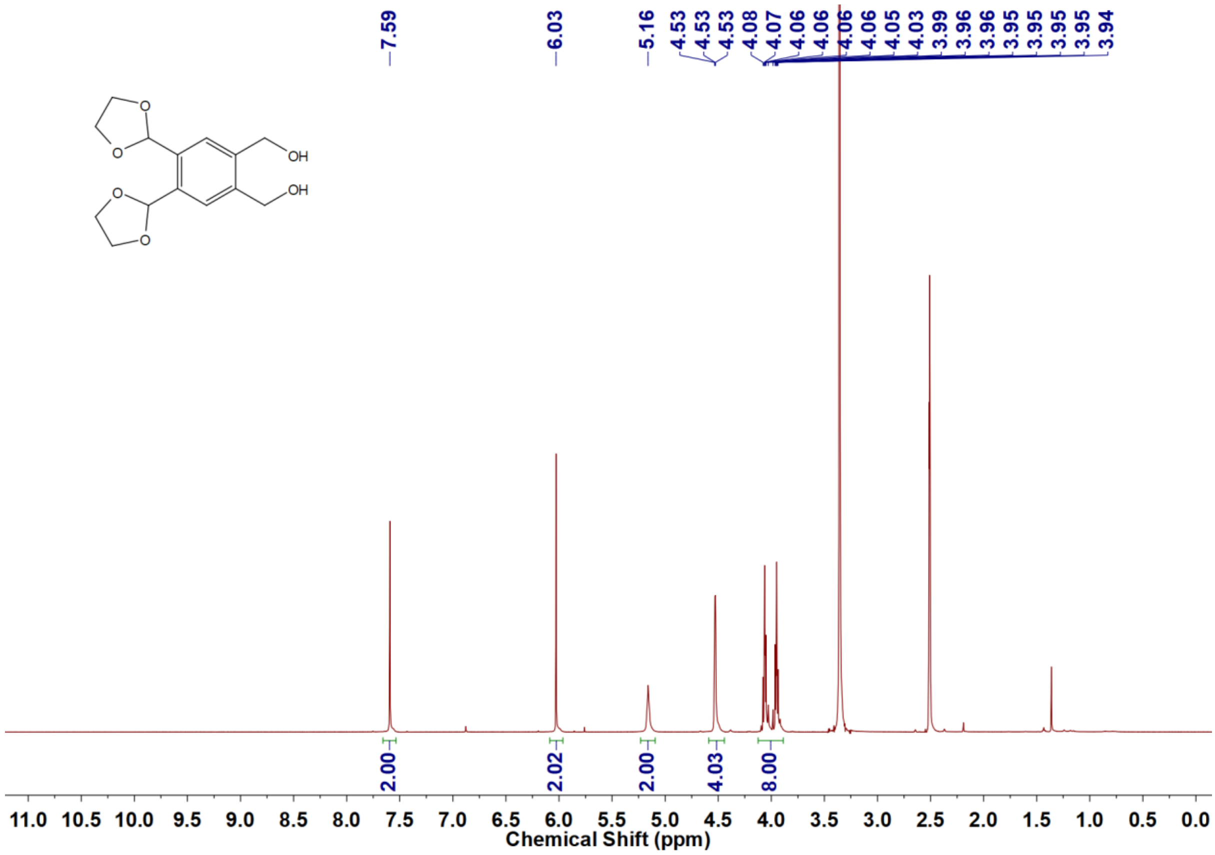

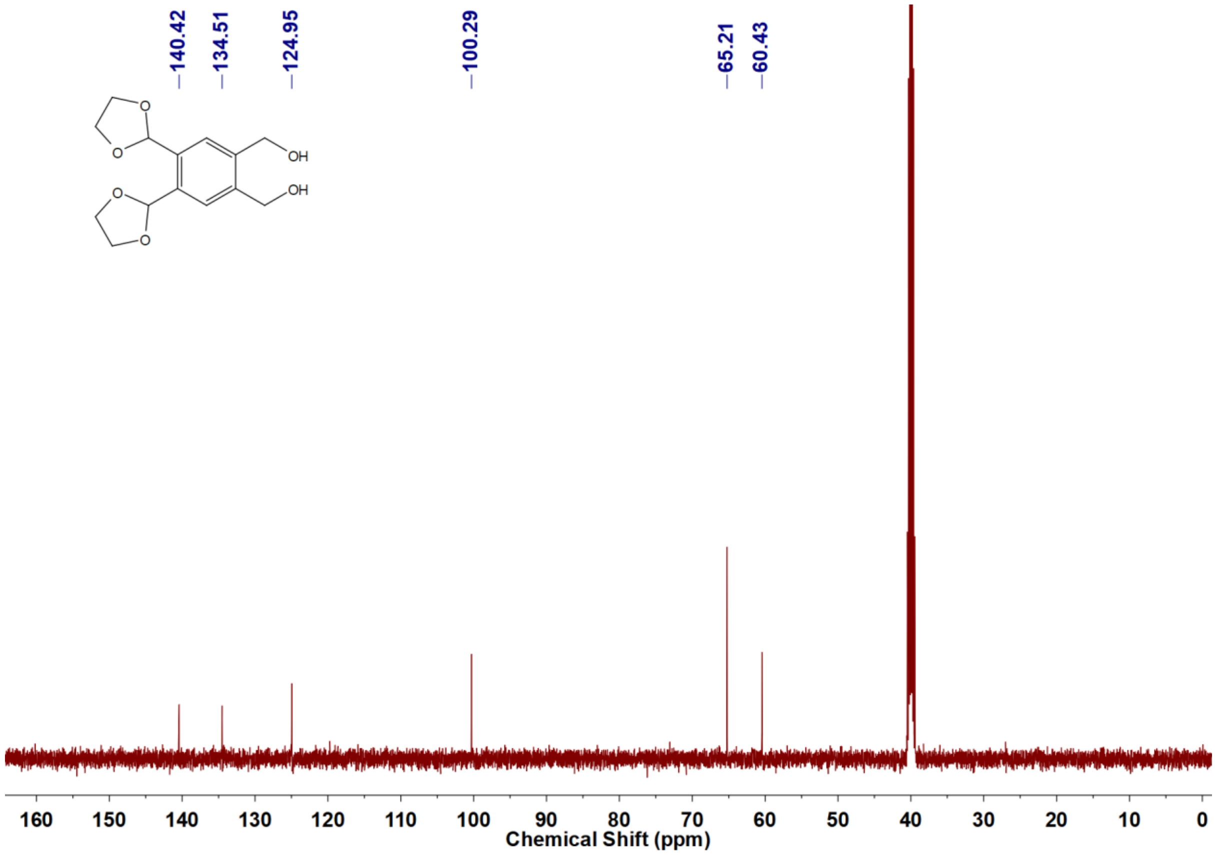

Figure S63. ^{1}H and ^{13}C NMR spectra of Compound S21 in $CDCl_3$.

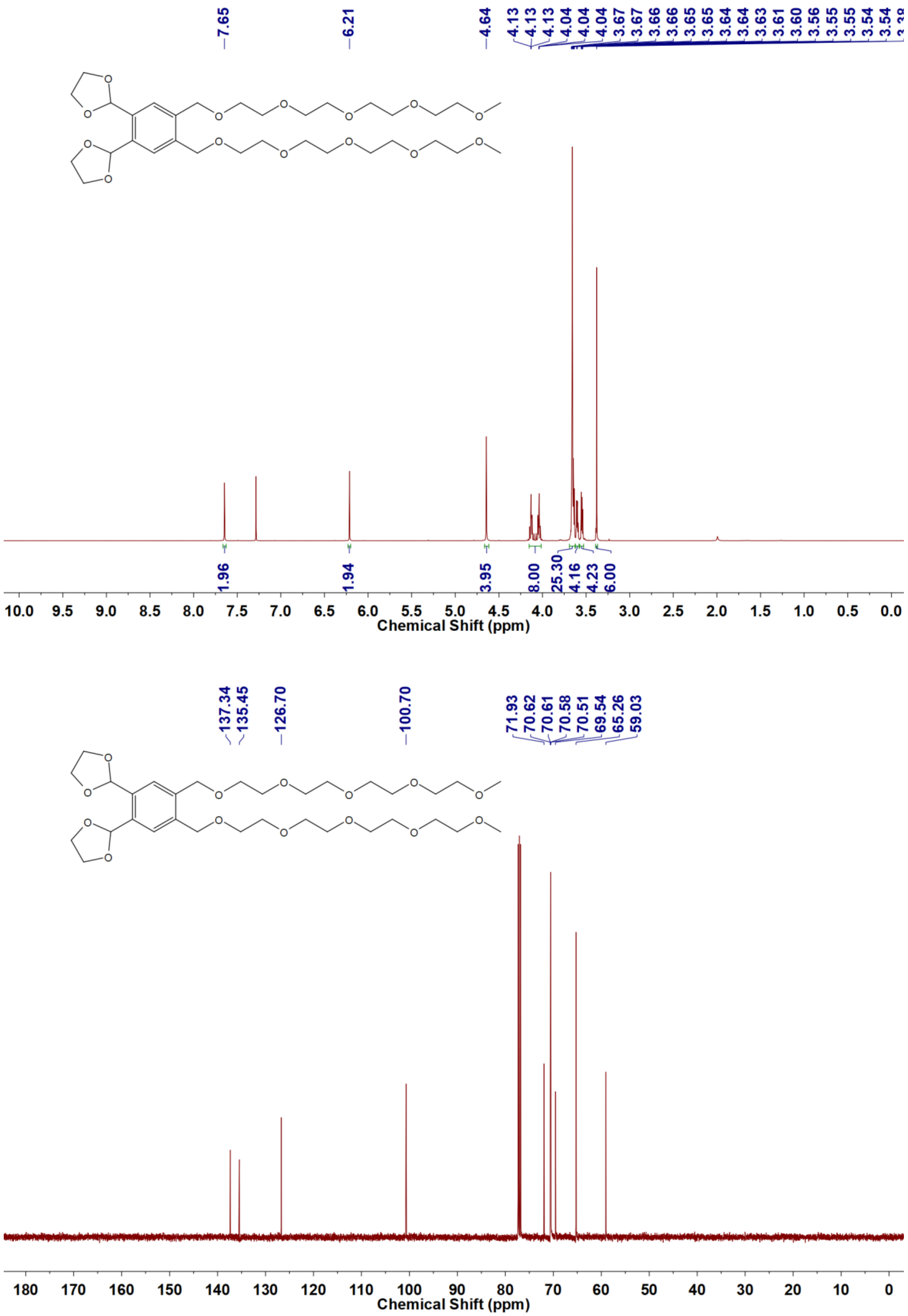

Figure S64. ^{1}H and ^{13}C NMR spectra of Compound S22 in $CDCl_3$.

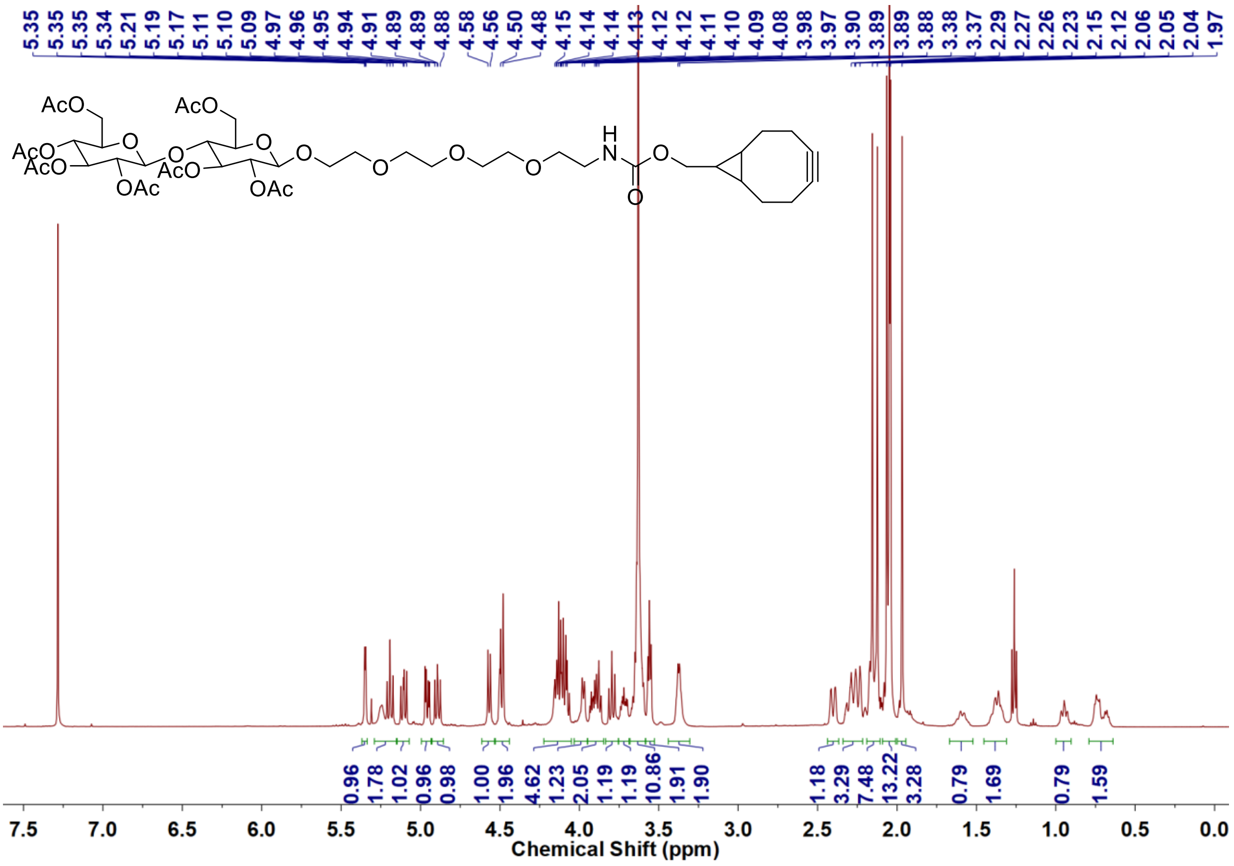

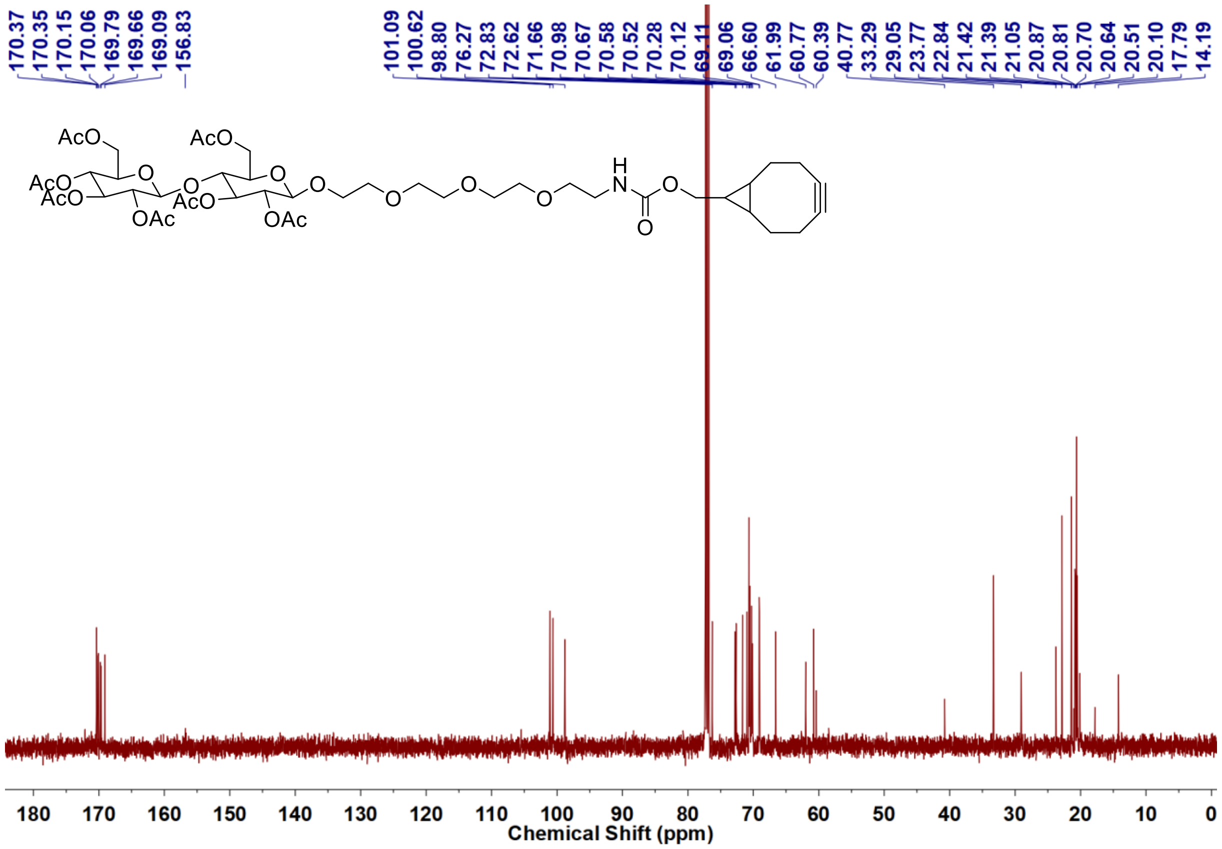

Figure S65. ^{1}H and ^{13}C NMR spectra of Compound S25 in $CDCl_3$.

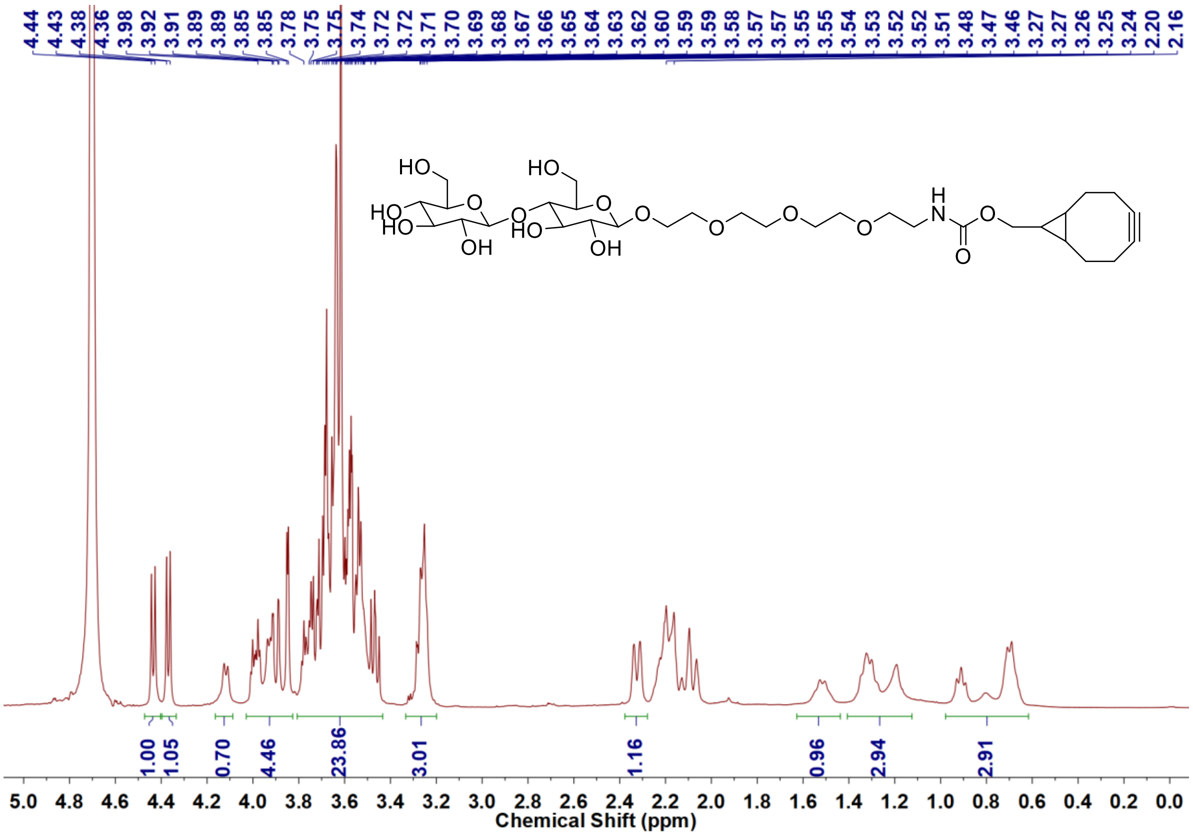

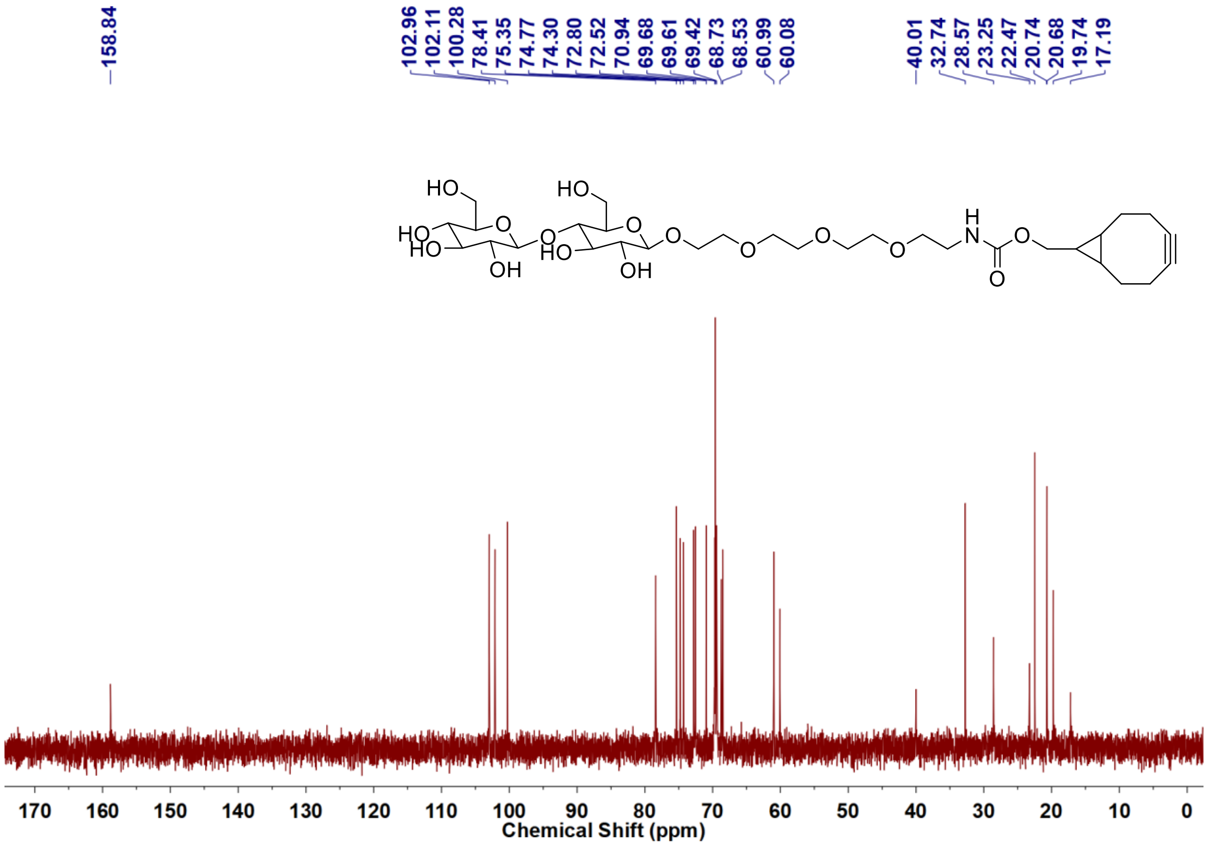

Figure S66. ^{1}H and ^{13}C NMR spectra of P2 in D_2O.

REFERENCES

[1] C. L. Tung, C. T. T. Wong, E. Y. M. Fung, X. Li, Traceless and chemoselective amine bioconjugation via phthalimidine formation in native protein modification. *Org. Lett.* **2016**, *18*, 2600-2603.

[2] P. C. Zhu, D. H. Wang, K. Lu, M. An efficient synthesis of substituted benzene-1,2-dicarboxaldehydes. *Sci. China, Ser. B* **2007**, *50*, 249-252.

[3] M. E. Bartlett, S. A. Shuler, D. J. Rose, L. M. Gilbert, R. A. Hegab, T. J. Lawton, R. E. Messersmith, Paintable proteins: biofunctional coatings via covalent incorporation of proteins into a polymer network. *New J. Chem.* **2021**, *45*, 22084-22092.

[4] Y. Zhang, Q. Zhang, C. T. T. Wong, X. Li, Chemoselective peptide cyclization and bicyclization directly on unprotected peptides. *J. Am. Chem. Soc.* **2019**, *141*, 12274-12279.

[5] J. P. Lutz, O. Davydovich, M. D. Hannigan, J. S. Moore, P. M. Zimmerman, A. J. McNeil, Functionalized and degradable polyphthalaldehyde derivatives. *J. Am. Chem. Soc.* **2019**, *141*, 14544-14548.

[6] V. Percec, P. Leowanawat, H. J. Sun, O. Kulikov, C. D. Nusbaum, T. M. Tran, A. Bertin, D. A. Wilson, M. Peterca, S. Zhang, N. P. Kamat, K. Vargo, D. Moock, E. D. Johnston, D. A. Hammer, D. J. Pochan, Y. Chen, Y. M. Chabre, T. C. Shiao, M. Bergeron-Brlek, S. Andre, R. Roy, H. J. Gabius, P. A. Heiney, Modular Synthesis of Amphiphilic Janus Glycodendrimers and Their Self-Assembly into Glycodendrimersomes and Other Complex Architectures with Bioactivity to Biomedically Relevant Lectins. *J. Am. Chem. Soc.* **2013**, *135*, 9055-9077.

[7] J. A. Kaitz, C. E. Diesendruck, J. S. Moore, End group characterization of poly(phthalaldehyde): surprising discovery of a reversible, cationic macrocyclization mechanism. *J. Am. Chem. Soc.* **2013**, *135*, 12755-12761.

[8] Q. Chen, P. C. Pang, M. E. Cohen, M. S. Longtine, D. J. Schust, S. M. Haslam, S. M. Blois, A. Dell, G. F. Clark, Evidence for differential glycosylation of trophoblast cell types. *Mol Cell Proteomics* **2016,** *15* (6), 1857-1866.

[9] A. Shevchenko, H. Tomas, J. Havlis, J. V. Olsen, M. Mann, In-gel digestion for mass spectrometric characterization of proteins and proteomes. *Nat Protoc* **2006,** *1* (6), 2856-2860.

[10] M. Wang, A. Shajahan, L. E. Pepi, P. Azadi, J. Zaia, Glycoproteomic sample processing, LC-MS, and data analysis using GlycReSoft. *Curr Protoc* **2021,** *1* (3), e84.

[11] Y. Liu, K. Yu, K. Zhang, M. Niu, Q. Chen, Y. Liu, L. Wang, N. Zhang, W. Li, X. Zhong, G. Li, S. Wu, J. Zhang, Y. Liu, O-GlcNAcylation promotes topoisomerase IIalpha catalytic activity in breast cancer chemoresistance. *EMBO Rep* **2023,** *24* (7), e56458.

[12] C. Chen, Y. Wu, J. Li, X. Wang, Z. Zeng, J. Xu, Y. Liu, J. Feng, H. Chen, Y. He, R. Xia, TBtools-II: A “one for all, all for one” bioinformatics platform for biological big-data mining. *Mol. Plant* **2023**, *16*, 1733-1742.

[13] T. Wang, W. Hong, RILP interacts with VPS22 and VPS36 of ESCRT-II and regulates their membrane recruitment. *Biochem. Biophys. Res. Commun.* **2006**, *350*, 413-423.

[14] D. X. Luo, M. C. Huang, J. Ma, Z. Gao, D. F. Liao, D. Cao, Aldo-keto reductase family 1, member B10 is secreted through a lysosome-mediated non-classical pathway. *Biochem. J.* **2011**, *438*, 71-80.

[15] P. Nair, B. E. Schaub, J. Rohrer, Characterization of the endosomal sorting signal of the cation-dependent mannose 6-phosphate receptor. *J. Biol. Chem.* **2003**, *278*, 24753-24758.

[16] G. Bjorkoy, T. Lamark, A. Brech, H. Outzen, M. Perander, A. Overvatn, H. Stenmark, T. Johansen, p62/SQSTM1 forms protein aggregates degraded by autophagy and has a protective effect on huntingtin-induced cell death. *J. Cell. Biol.* **2005**, *171*, 603-614.

[17] A. C. Gonzalez, M. Schweizer, S. Jagdmann, C. Bernreuther, T. Reinheckel, P. Saftig, M. Damme, Unconventional trafficking of mammalian phospholipase D3 to lysosomes. *Cell Rep.* **2018**, *22*, 1040-1053.

[18] S. K. Kachhap, D. Faith, D. Z. Qian, S. Shabbeer, N. L. Galloway, R. Pili, S. R. Denmeade, A. M. DeMarzo, M. A. Carducci, The N-Myc down regulated Gene1 (NDRG1) Is a Rab4a effector involved in vesicular recycling of E-cadherin. *PLoS One* **2007**, *2*, e844.

[19] F. Schimmoller, E. Diaz, B. Muhlbauer, S. R. Pfeffer, Characterization of a 76 kDa endosomal, multispanning membrane protein that is highly conserved throughout evolution. *Gene* **1998**, *216*, 311-318.

[20] A. Shiels, T. M. Bennett, H. L. Knopf, K. Yamada, K. Yoshiura, N. Niikawa, S. Shim, P. I. Hanson, CHMP4B, a novel gene for autosomal dominant cataracts linked to chromosome 20q. *Am. J. Hum. Genet.* **2007**, *81*, 596-606.

[21] M. D. Stuchell, J. E. Garrus, B. Muller, K. M. Stray, S. Ghaffarian, R. McKinnon, H. G. Krausslich, S. G. Morham, W. I. Sundquist, The human endosomal sorting complex required for transport (ESCRT-I) and its role in HIV-1 budding. *J. Biol. Chem.* **2004**, *279*, 36059-36071.

[22] S. M. Ortega-Campos, J. M. Garcia-Heredia, The multitasker protein: a look at the multiple capabilities of NUMB. *Cells* **2023,** *12* (2), 333.

[23] S. Scheuring, R. A. Rohricht, B. Schoning-Burkhardt, A. Beyer, S. Muller, H. F. Abts, K. Kohrer, Mammalian cells express two VPS4 proteins both of which are involved in intracellular protein trafficking. *Journal of molecular biology* **2001,** *312* (3), 469-480.

[24] K. L. Zulkefli, F. J. Houghton, P. Gosavi, P. A. Gleeson, A role for Rab11 in the homeostasis of the endosome-lysosomal pathway. *Exp Cell Res* **2019,** *380* (1), 55-68.

[25] S. Lucken-Ardjomande Hasler, Y. Vallis, M. Pasche, H. T. McMahon, GRAF2, WDR44, and MICAL1 mediate Rab8/10/11-dependent export of E-cadherin, MMP14, and CFTR DeltaF508. *J Cell Biol* **2020,** *219* (5).

[26] L. Fagerberg, C. Stadler, M. Skogs, M. Hjelmare, K.Jonasson, M. Wiking, A. Abergh, M. Uhlen, E. Lundberg, Mapping the subcellular protein distribution in three human cell lines. *J Proteome Res* **2011,** *10* (8), 3766-77.
